# Comparative Proteomic and Phosphoproteomic Analyses Reveal Molecular Signatures of Myocardial Infarction and Transverse Aortic Constriction in Aged Mouse Models

**DOI:** 10.1155/2024/9395213

**Published:** 2024-10-28

**Authors:** Fang Lin, Yue Ding, Xiaoting Liang

**Affiliations:** ^1^Institute for Regenerative Medicine, Shanghai East Hospital, School of Life Sciences and Technology, Tongji University, Shanghai, China; ^2^Research Center for Translational Medicine, Shanghai East Hospital, Tongji University School of Medicine, Tongji University, Shanghai, China; ^3^Shanghai Heart Failure Research Center, Shanghai East Hospital, Tongji University School of Medicine, Shanghai, China; ^4^Department of Organ Transplantation, Changzheng Hospital, Second Military Medical University, Shanghai, China

**Keywords:** aging, hypertension, myocardial infarction, phosphoproteome, proteome

## Abstract

In the elderly population, coronary heart disease (CHD) often coexists with hypertension. However, excessive blood pressure reduction can paradoxically increase the incidence of adverse events. Understanding the molecular mechanisms underlying hypertension and CHD in aged populations is crucial for developing targeted therapies and improving clinical outcomes. In this study, we constructed myocardial infarction (MI) and transverse aortic constriction (TAC) modelsY in aged mice to simulate the disease states of CHD and hypertension, respectively. Using integrated proteomic and phosphoproteomic analyses, we investigated the molecular signatures associated with MI and TAC in these models. Our aim was to identify key molecules involved in these conditions and to understand their unique and shared characteristics. Through our comprehensive proteomic and phosphoproteomic analysis, we identified a total of 1583 proteins and 232 phosphorylated proteins. We observed significant upregulation of heart disease markers such as Myh7, Xirp2, and Acta1, indicating the successful establishment of the MI and TAC models. The overlapped differentially expressed proteins (DEPs) and differentially phosphorylated proteins (DPPs) in MI and TAC were involved in heart failure-related processes including cardiac muscle contraction and hypertrophic cardiomyopathy, further supporting the validity of the models. Among the DEPs, Ppme1 was upregulated in the TAC model but downregulated in the MI model, while Sec31a and Gm56451 displayed the opposite expression patterns. Among the DPPs, Ablim1 and Atp2a2 were found to be significantly upregulated in the TAC model, whereas their expression was markedly reduced in the MI model. In addition, five other DPPs, including REV_Q3TAY5, Cbx3, PITPNB, Eif4b, and A0A1Y7VP73, were elevated in the MI model but decreased in the TAC model. In conclusion, these findings suggest that MI and TAC not only share certain molecular features but also retain their unique characteristics, providing potential biomarkers and therapeutic targets.

## 1. Introduction

Hypertension significantly increases the risk of various cardiovascular events, such as ischemic and hemorrhagic stroke, myocardial infarction (MI), heart failure, and premature death. The risk of cardiovascular disease (CVD) increased consistently as baseline systolic blood pressure (SBP) and diastolic blood pressure (DBP) rose above the typical levels of 115 mm·Hg for SBP and 75 mm·Hg for DBP [1]. In hypertensive patients, the risk of complications and adverse outcomes both immediately following and during the long-term course of acute MI is significantly higher [2, 3]. This elevated risk arises from persistent high blood pressure, which damages coronary arteries and accelerates atherosclerosis. Plaque buildup increases the likelihood of rupture and thrombosis, key mechanisms that block blood flow and trigger MI, the hallmark of coronary heart disease (CHD). However, despite the well-established benefits of antihypertensive medications in managing hypertension, their effectiveness in patients with CHD is still a subject of debate, especially in older people [4, 5]. Overly aggressive blood pressure reduction in these patients may lead to adverse outcomes, including increased risk of cardiovascular events and mortality [6]. Therefore, understanding the molecular mechanisms linking hypertension and MI is crucial for developing targeted therapies and improving clinical outcomes.

The relationship between hypertension and MI becomes even more complex when considering the effects of aging, as age is inevitably a critical factor closely associated with both conditions [7]. Mechanically, as individuals age, the prevalence and severity of hypertension increase, leading to gradual damage to blood vessels and impairment of cardiac function over time. On the other hand, age-related changes in the heart, such as increased stiffness and reduced ability to adapt to stress, exacerbate the impact of hypertension and heighten susceptibility to MI. Therefore, understanding the distinct and overlapping molecular mechanisms of hypertension and MI in elderly populations is crucial. From this perspective, the proteomic analysis provides valuable insights by identifying differentially expressed proteins (DEPs) that contribute to disease progression, allowing researchers to map complex molecular changes linked to aging and CVD. Phosphoproteomic analysis, in contrast, adds further depth by focusing on protein phosphorylation, a key posttranslational modification that affects about one-third of proteins in eukaryotes at any given time [8]. Phosphorylation regulates protein activity, stability, and interactions, particularly in signal transduction pathways. By combining proteomic and phosphoproteomic analyses, researchers can gain a more comprehensive understanding of both the expression and functional modification of proteins, offering deeper insights into the molecular pathways that underlie disease mechanisms in aging populations.

In this study, we conducted integrated proteomic and phosphoproteomic analyses to identify key molecules involved in MI and transverse aortic constriction (TAC) in aged mouse models. MI represents the acute damage to heart tissue due to the blockage of coronary arteries, often resulting from hypertension-induced atherosclerosis. On the other hand, TAC mimics increased left ventricular afterload, a condition commonly caused by aortic stenosis or systemic hypertension in clinical settings. This approach aims to elucidate the molecular mechanisms underlying hypertension and CHD in aged populations, potentially informing targeted therapies and improving outcomes for elderly patients with similar conditions.

## 2. Materials and Methods

### 2.1. Animal Models

Our animal study protocol strictly adheres to the “Guide for the Care and Use of Laboratory Animals” published by the US National Institutes of Health (Publication no. 85–23, revised in 1996) and has received approval from the Institutional Animal Care and Use Committee of Tongji University for Laboratory Animal Medicine (Permit no. TJBB04723102). We obtained 11-month-old male C57/B6J mice from Vital River Laboratories in Zhejiang, China. The mice were housed in a controlled environment with a temperature range of 20–25 degrees Celsius, relative humidity maintained at 50%–60%, and a 12-h light-dark cycle. After acclimatizing for 1 week in specific pathogen-free (SPF) conditions, the mice were randomly allocated into three groups: sham, MI, and TAC models. MI was induced by occluding the left anterior descending (LAD) artery according to established procedures (*n* = 3) [9]. In brief, mice were anesthetized with an intraperitoneal injection of pentobarbital (50 mg/kg) and connected to a mouse ventilator via tracheal intubation. The middle of the LAD artery was ligated using 8-0 sutures via a left thoracotomy. The TAC model was established by ligating the transverse aorta, following previously described methods (*n* = 3) [10, 11]. In brief, mice were anesthetized and then the TAC pressure overload model was accomplished by ligation of the transverse aorta between the right innominate and left common carotid arteries against a blunted 27‐gauge needle with a 7‐0 suture. The needle was then gently removed. For the sham group, the mice underwent thoracotomy without ligation of the LAD or transverse aorta, followed by suturing to close the chest. After 28 days post-MI or TAC induction, the mice were anesthetized using isoflurane (5 vol% isoflurane in oxygen) for cardiac function assessment via echocardiography. Subsequently, euthanasia was performed by administering CO_2_ inhalation, and the hearts were harvested for further analysis.

### 2.2. Echocardiographic Assessment of Cardiac Function

Cardiac function was accessed by transthoracic echocardiography at 4 weeks after MI or TAC surgery (Ultramark 9; Soma Technology, Bloomfield, CT, United States). The echocardiogram was employed to compute dimensions, including the left ventricular end-diastolic diameter (LVEDD), left ventricular end-systolic diameter (LVESD), left ventricular shortening rate (LVFS), and left ventricular ejection fraction (LVEF).

### 2.3. Tissue Collection

At 4 weeks after MI or TAC, cardiac function was evaluated and then the mice were euthanized. After removing the atrial tissue, the ventricular tissue was cut horizontally and divided into two parts. Half of the tissue near the cardiac apex was allocated for downstream protein extraction, while the other half near the cardiac base was designated for paraffin embedding and tissue sectioning. For protein extraction, heart tissues were collected and lysed in RIPA buffer (cell signaling, cat#9806) with a protease inhibitor cocktail. Samples were sonicated with a Bioruptor sonicator (Diagenode) at high intensity with 15-s pulses and intervals for 5 min and centrifuged at 14, 000*g* for 15 min. Proteins were quantified by BCA assay (Thermo, cat#231227) as previously described [12]. In each experimental group, the most representative individuals were selected based on echocardiography and histological results. These selected mice were then used for further mass spectrometry (MS) analysis to investigate their proteomic or phosphoproteomic profiles.

### 2.4. Wheat germ agglutinin (WGA) Staining

After cardiac function measurements, the mouse cardiac tissues were collected and fixed with 4% paraformaldehyde, embedded in paraffin, and sectioned. To measure the cardiomyocyte area, cardiac sections were stained with WGA (Invitrogen, USA) as previously described [13]. Fluorescent signals were observed and recorded using a Leica DM6000B microscope. Five high-power fields were chosen at random for measuring myocyte cross-sectional area (CSA) through ImageJ software (Version 1.53k, National Institutes of Health, Bethesda, Maryland, USA). Each experimental group involved the quantification of over 200 myocytes from three distinct animals.

### 2.5. Protein Extraction and Tryptic Digestion

Tissue proteins were lysed in RIPA buffer supplemented with a protease inhibitor cocktail as described above. A total of 100 *μ*g protein was diluted to a concentration of 1 mg/mL using RIPA buffer and precipitated using excess acetone. Subsequently, the acetone-precipitated proteins were washed three times with cooled acetone and then pumped out using the Concentrator plus (Eppendorf, Germany). Filter-aided sample preparation (FASP) procedure was used for protein digestion [100]. The proteins were resuspended in 200 *μ*L 8 M urea (pH 8.0) and loaded twice in 30 kD Microcon filter tubes (Sartorius) and centrifuged at 12, 000*g* for 20 min. The precipitate in the filter was washed twice by adding 200 *μ*L of 50 mM NH_4_HCO_3_. The precipitate was resuspended in 50 *μ*L of 50 mM NH_4_HCO_3_. Protein samples underwent trypsin digestion (enzyme-to-substrate ratio of 1:50 at 37°C for 18–20 h) in the filter and then were collected by centrifugation at 12, 000*g* for 15 min. Additional washing, twice with 200 *μ*L of MS water, was essential to obtain greater yields. Finally, the centrifugate was dried by using the Concentrator plus (Eppendorf, Germany). The dried peptides were redissolved with 100 *μ*L of 10 mM NH_4_HCO_3_ and loaded into a homemade Durashell reverse-phase column (2 mg packing [3 *μ*m, 150 Å, Agela] and two C18 layers [12 *μ*m, Empore] in a 200-*μ*L tip) and then eluted sequentially with nine gradient elution buffer that contains 6%, 9%, 12%, 15%, 18%, 21%, 25%, 30%, and 35% ACN (diluted with 10 mM of NH_4_HCO_3_ and adjusted pH to 10.0 using NH_3_·H_2_O). The nine fractions then were combined into three groups (6% + 15% + 25%, 9% + 18% + 30%, 12% + 21% + 35%) and dried under Concentrator plus (Eppendorf, Germany) for subsequential MS analysis.

### 2.6. Phosphopeptide Enrichment

For phosphopeptide enrichment, TiO_2_ beads were prepared at a ratio of 6:1 (beads: protein). Each sample's corresponding beads were resuspended in 200 *μ*L of loading buffer (80% ACN; 6% TFA) and sonicated at 4°C for 1 min to ensure uniform distribution. The TiO_2_ beads were then mixed with the peptide samples and incubated at 40°C and 2000 rpm for 5 min using a thermo mixer. The mixture was transferred to C8 stage tips and centrifuged at low speed until no liquid remained. The beads were washed five times with pho-W buffer (four times with 200 *μ*L each, and the final wash with 100 *μ*L). Phosphopeptides were eluted by washing twice with 50 *μ*L of pho-E1 buffer and once with 50 *μ*L of pho-E2 buffer. The three eluents were combined, dried under vacuum at room temperature, and reconstituted in 0.1% FA for subsequent LC–MS/MS analysis.

### 2.7. LC–MS/MS Analysis

For proteome profiling, samples were analyzed using an EASY-nLC 1200 nano-UPLC system coupled to a Q Exactive mass spectrometer (Thermo Scientific) with a total analysis time of 240 min per sample. The analysis was performed in positive ion detection mode with a scan range of 350–1600 m/z. Data-dependent acquisition (DDA) was employed, with each full scan followed by 20 MS2 scans (HCD). The resolution for MS1 at m/z 200 was set to 70,000 and for MS2 at m/z 200, it was set to 17,500. The AGC targets were 3e^6^ for MS1 and 1e^5^ for MS2, with maximum ion injection times of 50 and 45 ms, respectively. The normalized collision energy (NCE) was set to 28%, with an isolation window of 2 m/z and a dynamic exclusion time of 40 s.

For the phosphoproteomic analysis, half of the enriched phosphopeptides from each sample were separated using an EASY-nLC 1200 nano-UPLC system and analyzed online with a Q Exactive mass spectrometer (Thermo Scientific). The separation was performed on a 100 *μ*m ID × 15 cm reverse-phase column (ReproSil-Pur 120 C18-AQ, 1.9 *μ*m, Dr. Maisch) with a flow rate of 300 nL/min over a 240-min gradient. Mobile Phase A consisted of 0.1% formic acid in water with 2% acetonitrile, and Mobile Phase B consisted of 0.1% formic acid in 80% acetonitrile. The gradient was as follows: 8%–35% B for 184 min, 35%–45% B for 40 min, 45%–100% B for 4 min, 100% B for 4 min, 100%–2% B for 4 min, and 2% B for 4 min.

### 2.8. Peptide Identification and Protein Quantification

For proteome profiling, the LC–MS/MS raw files were processed with MaxQuant v1.5.6.0 (Max Planck Institute, Munich, Germany). Protein sequences were sourced from the UniProt database (UniProt-mouse-20200526). Both the protein sequences and their reversed decoy sequences were utilized for database searching in MaxQuant. Trypsin was set as the specific protease with a maximum of 3 missed cleavage sites. Oxidation (M) and acetyl (protein N-term) were set as variable modifications. Carbamidomethyl (C) was set as a fixed modification with a maximum of 3 variable modifications per peptide. The false discovery rate (FDR) was set to 0.01 at both the peptide and protein levels. Only unique peptides without variable modifications were used for quantification. Proteins with a fold change ratio of > 1.5 and at least 2 unique peptides were defined as significantly DEPs.

For the phosphoproteomic data, the LC–MS/MS raw files were processed with MaxQuant (version 1.6.1.0). The protein database utilized was from UniProt (UniProt-mouse-20200526), with label-free quantification (LFQ) based on MS1 intensity measurements. The specific protease used was trypsin/P, allowing up to four missed cleavages. Variable modifications included oxidation (M), acetyl (protein N-term), and phosphorylation (STY), while carbamidomethyl (C) was set as a fixed modification. The maximum number of modifications per peptide was set to five, with a minimum peptide length of seven amino acids and a maximum peptide mass of 4600 Da. The FDR was controlled at 0.01 for both peptide and protein levels. Only unique peptides were used for quantification, and phosphorylation (STY) was the only modification included in the quantification analysis; other variable modifications were excluded from quantification. Proteins with a fold change ratio of > 1.5 were defined as significantly differentially phosphorylated proteins (DPPs).

### 2.9. Bioinformatic Analysis and Data Visualization

DEPs and DPPs were subjected to KEGG pathway enrichment analysis in DAVID (https://david.ncifcrf.gov/) with the *p* value of < 0.05. The significance of the pathway enrichment analysis was determined by Fisher's exact test on the basis of KEGG pathways. A scatter plot of nine-quadrant association analyses, illustrating protein or phosphoprotein levels in MI and TAC based on log2-fold changes, was generated using the online platform provided by OmicShare (https://www.omicshare.com/).

### 2.10. Statistics

Continuous variables are expressed as mean ± SEM. Comparisons of variables between multiple groups were performed using one-way analysis of variance (ANOVA) followed by Bonferroni post hoc tests to identify significant differences between groups. Data analyses were conducted using SPSS (Version 25.0). Statistical significance was set at *p* < 0.05.

## 3. Results

### 3.1. Successful Establishment of MI and TAC in Aged Mice

**Figure 1:**
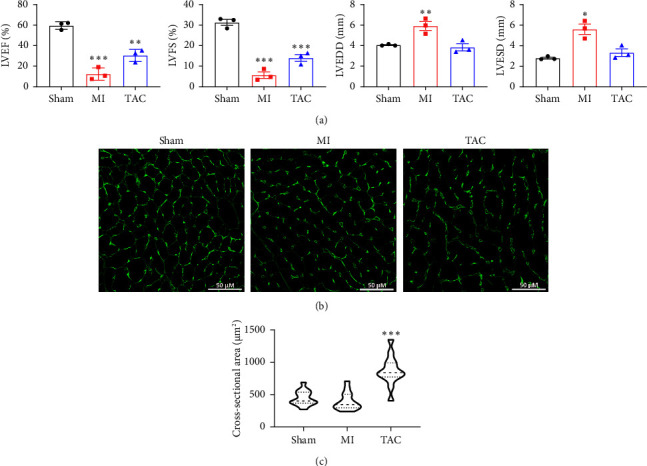
Cardiac function and WGA staining in aged mice with MI and TAC. (a) Quantitative analysis of LVEF, LVFS, LVESD, and LVEDD in sham, MI, and TAC mice at 28 days postsurgery. *n* = 3 for each group. Values are shown as mean ± SEM. ⁣^∗^*p* < 0.05 vs sham, ⁣^∗∗^*p* < 0.01 vs sham, and ⁣^∗∗∗^*p* < 0.001 vs sham. (b) Representative images of WGA staining. (c) Quantitative analysis of cardiomyocyte CSA. *n* = 3 for each group. Values are shown as mean ± SEM. ⁣^∗∗∗^*p* < 0.001 vs sham. Abbreviations: CSA: cross-sectional area; LVEF: left ventricular ejection fraction; LVEDD: left ventricular end-diastolic dimension; LVESD: left ventricular end-systolic dimension; LVFS: left ventricular fraction shortening.

After 28 days following the induction of MI or TAC, cardiac function was evaluated using echocardiography. Mice in the MI group exhibited significantly lower ejection fraction (EF) and fractional shortening (FS) values, along with increased LVESD and LVEDD compared to both the sham and TAC groups (Figure 1(a)). This indicates the development of significant systolic dysfunction and ventricular dilation, which are characteristic of MI. In contrast, mice in the TAC group also showed significantly lower EF and FS values compared to the sham group, indicating systolic dysfunction. However, there were no significant changes in LVESD and LVEDD in the TAC group compared to the sham group (Figure 1(a)), which is consistent with the development of pressure overload without ventricular dilation. Cardiac hypertrophy was assessed by measuring the CSA of the left ventricle using WGA staining (Figures 1(b) and 1(c)). The CSA was not significantly different between the MI group and the sham group, suggesting that hypertrophy was not a major feature in the MI model. However, the TAC group showed a dramatic increase in CSA (approximately 2-fold) compared to the sham group, indicating significant cardiomyocyte hypertrophy (Figures 1(b) and 1(c)), which is typical of pressure overload induced by TAC. These results confirm the successful establishment of MI and TAC models in aged mice, with MI characterized by significant reductions in EF and FS along with ventricular dilation, and TAC characterized by marked cardiac hypertrophy and systolic dysfunction without significant ventricular dilation.

### 3.2. Proteomic Analysis of MI and TAC Heart Tissue

**Figure 2:**
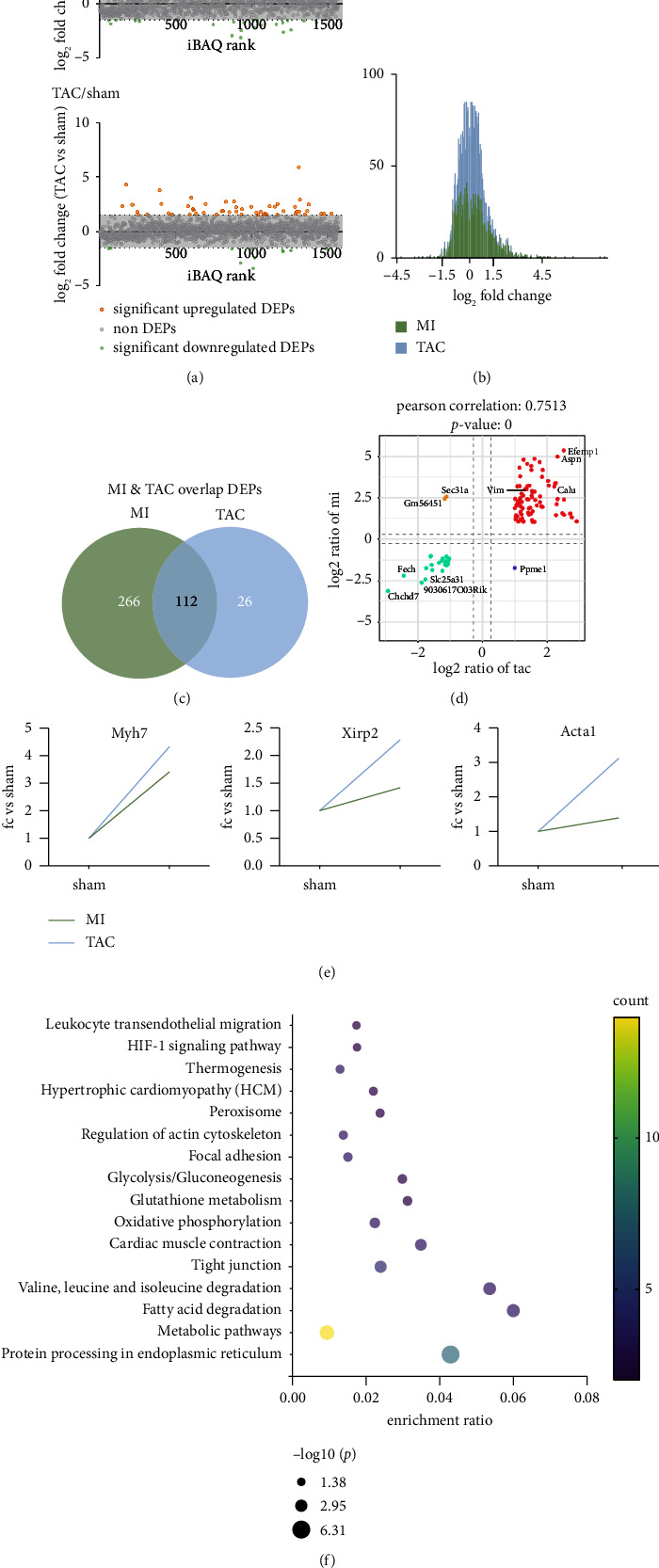
Results of proteomic profiling of heart tissues. (a) Volcano plots showing different group comparisons. Orange dots represent log2 (fold change) ≥ 1.5, while green dots represent log2 (fold change) ≤ −1.5. (b) Histogram depicting the distribution of log2-fold change comparing proteomics of MI and TAC. (c) Venn diagram illustrating the overlap of differentially expressed proteins (DEPs) identified in MI and TAC. (d) Scatter plot of nine-quadrant association analyses of protein levels in MI and TAC based on log2-fold change. (e) Expression levels of cardiac disease markers in MI and TAC. (f) KEGG enrichment analysis of coexpressed DEPs in MI and TAC.

Moving forward, a proteomic analysis was performed on heart tissue samples from aged mice with MI and TAC. In total, 1583 proteins were identified, of which 1554 had at least two unique peptides (Table S1). Comparative proteomic analysis revealed that 378 proteins were differentially expressed in MI mice, with 280 proteins upregulated and 98 proteins downregulated (fold change ≥ 1.5, Figure 2(a) and Table S2). In the case of TAC, we identified 138 DEPs, with 104 upregulated and 34 downregulated (fold change ≥ 1.5, Figure 2(a) and Table S3). Frequency distributions showed that the majority of proteins were not significantly affected by either MI or TAC injury, as the average log2-fold changes centered around zero, representing the different sets of proteins detected in each model (Figure 2(b)). Interestingly, MI and TAC shared 112 protein signatures, including 86 upregulated proteins and 26 downregulated proteins (Pearson correlation 0.7506, Figures 2(c) and 2(d) and Table S4). Several CVD markers, such as Myh7, Xirp2, and Acta1, were upregulated in both MI and TAC (Figure 2(e)). KEGG analysis indicated that the overlapping proteins in MI and TAC were associated with oxidative stress response, glucose/glutathione/fatty acid metabolism, hypertrophic cardiomyopathy, and cardiac muscle contraction (Figure 2(f)). Notably, Ppme1, Gm56451, and Sec31a showed contradictory expression patterns between MI and TAC (Figure 2(d)), suggesting their potential as biomarkers for MI- and TAC-induced heart failure, respectively. The differential expression of these proteins indicates distinct regulatory mechanisms and potentially different therapeutic targets for ischemic versus pressure overload–induced cardiac pathology.

### 3.3. Phosphoproteomic Analysis of MI and TAC Heart Tissue

**Figure 3:**
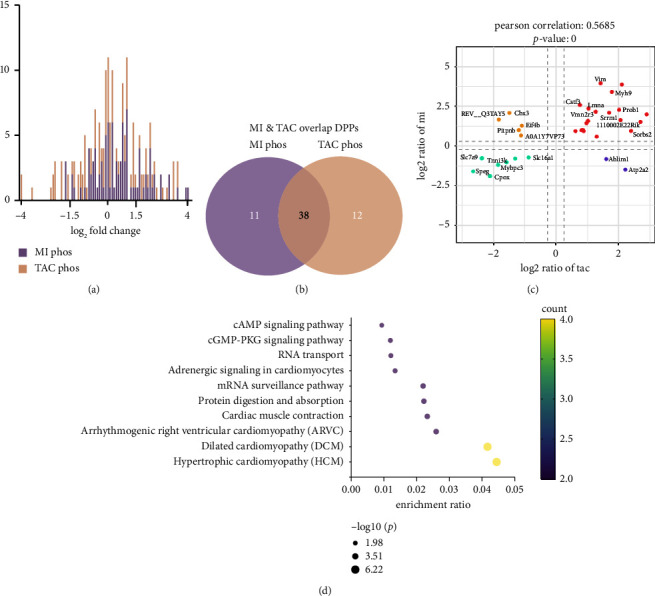
Results of phosphoproteomic profiling of heart tissues. (a) Histogram displaying the distribution of log2-fold change comparing phosphoproteomics of MI and TAC. (b) Venn diagram indicating the overlap of differentially phosphorylated proteins (DPPs) identified in MI and TAC. (c) Scatter plot of nine-quadrant association analyses of phosphoprotein levels in MI and TAC based on log2-fold change. (d) KEGG enrichment analysis of coexpressed DPPs in MI and TAC.

For the phosphopeptide datasets, the phosphorylation analysis identified 232 phosphoproteins with 356 phosphorylation sites (Table S5). Among these, 63 phosphopeptides corresponding to 49 proteins and 66 phosphopeptides corresponding to 50 proteins showed significant differential expression after MI and TAC, respectively (fold change ≥ 1.5, Tables S6 and S7). Frequency distributions showed that the majority of phosphoproteins were not significantly affected by MI or TAC injury, as the average log2-fold changes centered around zero (Figure 3(a)). When comparing the DPPs after MI and TAC challenge (fold change ≥ 1.5), we found 38 overlapping phosphoproteins (Pearson correlation 0.5685, Figures 3(b) and 3(c) and Table S8). Importantly, Ablim1 and Atp2a2 were upregulated in the TAC model but downregulated in the MI model (Figure 3(c)). Conversely, REV_Q3TAY5, Cbx3, PITPNB, Eif4b, and A0A1Y7VP73 were upregulated in MI (Figure 3(c)). These distinct expression patterns highlight potential biomarkers for differentiating between MI- and TAC-induced heart failure, reflecting their unique pathological processes. KEGG analysis indicated that the overlapping phosphoproteins in MI and TAC were associated with hypertrophic/dilated cardiomyopathy, arrhythmogenic right ventricular cardiomyopathy, cardiac muscle contraction, and adrenergic/cGMP-PKG/cAMP signaling (Figure 3(d)). These pathways are crucial for cardiac function and integrity, underscoring the role of posttranslational modifications in regulating cardiac physiology and injury responses.

### 3.4. Comparative Analysis of Proteomic and Phosphoproteomic Data

**Figure 4:**
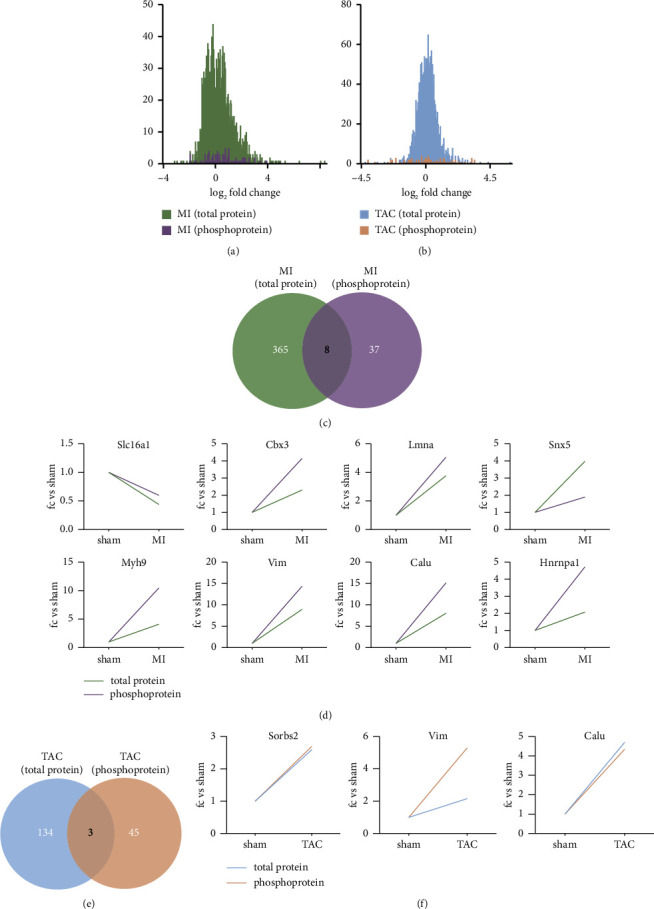
Comparison of proteomic–phosphoproteomic data. (a) Histogram showing the distribution of log2-fold change comparing total proteins and phosphoproteins in MI. (b) Histogram illustrating the distribution of log2-fold change comparing total proteins and phosphoproteins in TAC. (c) Venn diagram depicting the overlap of proteins and phosphoproteins identified in MI. (d) Expression levels of the overlapped proteins and phosphoproteins in MI. (e) Venn diagram indicating the overlap of proteins and phosphoproteins identified in TAC. (f) Visualization of the overlapped proteins and phosphoproteins in TAC.

Frequency distributions showed that the majority of phosphoproteins were not significantly affected in either MI or TAC, as the average log2-fold changes centered around zero (Figures 4(a) and 4(b)). This suggests that the phosphorylation states of most proteins remain relatively stable despite the cardiac injury, indicating specific regulatory phosphorylation events rather than widespread changes. In the MI model, eight proteins overlapped between proteomic and phosphoproteomic data, including Slc16a1, Cbx3, LMNA, Snx5, Myh9, Vim, Calu, and Hnrnpn1 (Figures 4(c) and 4(d)). Except for Slc16a1, which showed a downregulated expression after MI, the other proteins exhibited an upregulated expression pattern in both datasets (Figure 4(d)). This overlap implies that these proteins undergo both expression changes and posttranslational modifications, highlighting their potential importance in the MI response. In the TAC model, three proteins overlapped between proteomic and phosphoproteomic data, including Sorbs2, Vim, and Calu, all of which were upregulated after TAC in both proteomic and phosphoproteomic data (Figures 4(e) and 4(f)). The consistent upregulation of these proteins in both proteomic and phosphoproteomic analyses indicates their significant role in the heart's response to pressure overload.

## 4. Discussion

In this study, we conducted comprehensive proteomic and phosphoproteomic analyses of heart tissue in an aged mouse model of MI and TAC, with healthy aged mice serving as controls. We observed significant upregulation of heart disease markers Myh7, Xirp2, and Acta1, as well as alterations in proteins associated with hypertrophic cardiomyopathy and cardiac muscle contraction, indicating the successful establishment of the MI and TAC models. A total of 404 DEPs were identified, with 378 DEPs in the MI sample and 138 DEPs in the TAC sample. These findings underscore the distinct molecular changes induced by MI and TAC. The higher number of DEPs in MI suggests a more extensive remodeling of the cardiac proteome, likely due to the acute ischemic injury and subsequent infarct healing process. Conversely, the fewer changes observed in TAC may reflect the chronic nature of pressure overload and the resultant hypertrophic adaptation of the myocardium. In both proteomic and phosphoproteomic datasets, MI and TAC shared some protein signatures associated with cardiac structure and integrity. This suggests that despite the different underlying causes, both MI and TAC trigger similar fundamental stress responses in the heart. However, there were still distinct DEPs between MI and TAC. For example, Ppme1 was upregulated in TAC but downregulated in MI, while Sec31a and Gm56451 exhibited opposite patterns between the two models. In the phosphoproteomic data, we observed that Ablim1 and Atp2a2 were specifically upregulated in TAC but downregulated in MI, whereas REV_Q3TAY5, Cbx3, PITPNB, Eif4b, and A0A1Y7VP73 were upregulated in MI. These distinct expression patterns highlight potential biomarkers for differentiating between MI- and TAC-induced heart failure, reflecting their unique pathological processes.

The interactions between advanced age, hypertension, and MI are complex. Advanced age amplifies the detrimental effects of hypertension on the cardiovascular system, increasing the risk of developing MI [14]. Hypertension accelerates age-related cardiovascular changes, such as arterial stiffness and structural remodeling, which contribute to the pathogenesis of MI [15]. Furthermore, MI in older individuals with hypertension may lead to more severe complications and poorer outcomes due to reduced cardiac reserve and impaired adaptive capacity [16]. Understanding the intricate molecular signatures between advanced age, hypertension, and MI is crucial for the effective prevention, diagnosis, and management of CVDs in the elderly population.

The TAC model involves surgically narrowing the aorta to induce increased workload on the heart, leading to elevated blood pressure and cardiac remodeling [10]. This experimental model allows researchers to investigate the pathophysiological mechanisms underlying cardiac remodeling and hypertrophy in the context of elevated blood pressure. The mouse MI model induced by LAD ligation closely mimics the features of human MI, where the coronary artery is occluded, resulting in ischemic injury and necrosis of cardiac tissue. Using these experimental models, researchers can simulate the pathological processes and disease progression observed in humans, providing valuable insights into the molecular mechanisms and pathophysiology.

Protein expression and function are essential for understanding biological processes and disease pathogenesis. The emergence of proteomics and its application in protein profiling has revolutionized our understanding of cellular mechanisms. Proteomics enables the identification and quantification of posttranslational modifications, such as phosphorylation. The combination of proteomics and phosphoproteomics provides insights into the functional implications of phosphorylation events and their dynamic regulation in cellular processes and disease. For instance, Wang et al. analyzed protein and phosphoprotein changes in rats 4 weeks post-LAD ligation (MI model) and identified 129 DEPs and 33 DPPs [17]. Similarly, Wang et al. utilized proteomics and phosphoproteomics in a swine MI model, identifying 2099 DEPs and 1847 DPPs [18]. However, to date, no studies have compared the proteomic and phosphoproteomic changes in aged mice with MI and TAC. This gap in research highlights the novel aspect of our study, which aims to elucidate these differences and contribute to a deeper understanding of cardiac injury mechanisms in aging.

In this study, we successfully established MI and TAC models in aged mice and performed proteomic and phosphoproteomic analyses on heart tissues. Among the 1583 proteins detected in the proteomics analysis, 112 proteins were found to be coexpressed in both MI and TAC, indicating shared molecular characteristics and pathological alterations. This overlap highlights common pathways and mechanisms activated in response to cardiac stress, suggesting that certain molecular responses are conserved regardless of the type of cardiac injury. Among the overlapping proteins, three proteins, Ppme1, Sec31a, and Gm56451, exhibited differential expression between MI and TAC, highlighting their potential as biomarkers for distinguishing between these conditions. Ppme1, known to inactivate PP2A, a tumor suppressor, has been implicated in various cancers through the promotion of oncogenic MAP kinase and Akt pathways [19, 20]. Although Ppme1 has been detected in human cardiac muscles, its specific function in the heart has yet to be characterized. The differential expression of Ppme1 in MI and TAC suggests a role in cardiac stress response, potentially through the modulation of signaling pathways involved in cell survival and apoptosis. Sec31a is a crucial component of the COPII complex, which facilitates the transport of proteins from the endoplasmic reticulum (ER) to the Golgi apparatus. This protein transport is essential for the proper sorting and distribution of proteins within the cell, ensuring that proteins reach their correct destinations [21]. The differential expression of Sec31a between MI and TAC may reflect differences in cellular stress responses, with potential implications for ER stress and protein trafficking disorders in these conditions. Gm56415, also known as polypeptide N-acetylgalactosaminyltransferase 1 (GALNT1), is abnormally expressed in multiple cancers and affects tumor progression by initiating GalNAc-type O-glycosylation of various proteins [22]. GALNT1 has been reported to be required for heart valve development and maintenance of cardiac function [23]. The differential expression of Gm56451 in MI and TAC suggests that glycosylation pathways might be differentially regulated in ischemic versus pressure-overload cardiac injury, potentially influencing extracellular matrix remodeling and cell–cell interactions. The distinct expression patterns of Ppme1, Sec31a, and Gm56451 in MI and TAC highlight their potential as biomarkers for different types of heart failure. These genes' roles in CVDs, particularly in MI and TAC, remain largely unexplored. A comprehensive investigation into their functions could provide valuable insights and aid in developing targeted therapies. These findings emphasize the importance of molecular characterization in understanding the unique and shared mechanisms of cardiac injuries, ultimately improving diagnostic and therapeutic strategies for heart failure.

Phosphoproteomics enables the analysis of dynamic changes in protein phosphorylation levels under different conditions, providing insights into cellular responses, signaling cascades, and disease mechanisms. By comparing the phosphoproteomics data in MI and TAC, we identified 38 coexpressed phosphoproteins. Several CVD markers, such as Vim, Calu, Myh9, LMNA, and Tnni3k, exhibited significant alterations in both MI and TAC, highlighting common pathways involved in cardiac stress responses and structural integrity. Phospho-Atp2a2, one of the SERCA Ca^(2+)^-ATPases located in the sarcoplasmic or endoplasmic reticula of muscle cells, was specifically upregulated in TAC but not in MI. It has been reported that the promoter of Atp2a2 was remarkably decreased in both 1-week and 8-week TAC hearts [24]. Our data suggest that, in addition to experiencing epigenetic alterations, Atp2a2 may undergo posttranslational modifications in the pathological progression of TAC. In addition, we identified several phosphoproteins with distinct expression patterns between MI and TAC, including Ablim1, REV_Q3TAY5, Cbx3, PITPNB, Eif4b, and A0A1Y7VP73. Although the literature on their roles in CVDs is scarce, we can hypothesize their potential involvement based on known functions. For example, Ablim1 is a novel ubiquitin E3 ligase [25], which suggests that in TAC, Ablim1 might regulate protein turnover and stress responses. This regulation could help the heart adapt to increased mechanical load and prevent pathological remodeling. Similarly, Eif4b phosphorylation, which induces ATPase activity [26], might reflect alterations in energy metabolism. These changes could influence protein synthesis and energy homeostasis differently in MI and TAC contexts, potentially affecting how the heart manages energy demands under stress. Our approach does not provide conclusive evidence of the involvement of these proteins in MI or TAC; it rather offers a starting point to prioritize candidate proteins as potential contributors to the observed phenotypes. Further investigation is necessary to elucidate the molecular mechanisms and validate the involvement of these proteins in cardiac injury and repair processes.

The combination of proteomics and phosphoproteomics data allows the identification of protein abundance and modifications at the phosphorylation level, providing valuable insights into cellular processes and potential implications for disease mechanisms. In the MI model, eight proteins exhibited concordant expression changes in both proteomics and phosphoproteomics. These proteins are closely related to dilated cardiomyopathy (LMNA and MYH9) [27], fibrosis (Vim), monocarboxylate transport (Slc16a1) [28], vascular function (Cbx3 and Hnrnpn1) [29, 30], and endocytic trafficking (Snx5) [31]. In the TAC model, three proteins overlapped between proteomics and phosphoproteomics. Sorbs2, enriched in cardiomyocytes is essential for maintaining cytoskeletal structural integrity. Previous research reported that myocardial Sorbs2 expression was consistently upregulated in humans with ischemic and idiopathic cardiomyopathies, as well as in experimental animal models of heart failure [32]. The expression trend of Sorbs2 in our study aligns with these findings, suggesting its role in cardiac stress responses. Notably, Vim and Calu are two proteins that were upregulated in both the MI and TAC models, at both the total protein and phosphorylated protein levels. The established role of Vim in fibrosis is well documented, indicating its involvement in fibrotic processes across different cardiac stress models. Calu, a Ca^2+^ binding protein localized in the ER, regulates Ca^2+^ uptake during the excitation-contraction coupling process, essential for maintaining normal heart function [33, 34]. In addition, Calu has recently been identified as a potential biomarker associated with immune infiltration in heart failure, indicating its broader role in the cardiac immune response [35].

The current study has several limitations. First, the small sample size (*n* = 3/group) reduces the statistical power of the analysis and may limit the precision of our findings. Increasing the sample size would provide a more robust estimate of the observed effects and strengthen the validity of the results. In addition, there are inherent differences in the pathogenesis of MI and TAC between mice and humans. While mouse models offer valuable insights into the underlying mechanisms, they do not fully recapitulate the complexity of human disease. Therefore, further validation of our findings using human cell cultures or tissue samples is necessary to ensure the translational relevance of the data. Future studies will incorporate these approaches to strengthen the applicability of the results to human cardiovascular conditions.

In conclusion, by utilizing state-of-the-art proteome and phosphoproteome profiling techniques, we systematically investigated the molecular landscape of heart failure induced by MI and TAC in aged mouse models. Our findings are in line with those of Liu et al., who investigated protein and phosphoprotein expression differences in human ICM and DCM samples [36]. They observed that while the differentiation between ischemic cardiomyopathy and dilated cardiomyopathy based on protein expression was relatively weak, there were still distinct protein expression patterns unique to each condition. This suggests that, like in our study, each type of heart disease has its specific proteomic profile. These similarities reinforce the notion that common molecular mechanisms may be at play across different cardiac pathologies, while also underscoring the importance of identifying unique protein markers for better diagnostic and therapeutic approaches. Our comprehensive analysis revealed intricate protein networks and signaling pathways underlying the biological and metabolic changes in response to MI and TAC. Through this integrated approach, we identified numerous protein and phosphoprotein markers that play crucial functional roles in these pathological processes. Our detailed findings not only enhance our understanding of MI- and TAC-induced heart failure in the aged population but also provide an initial framework for further validation and mechanistic investigations.

## Data Availability

The mass spectrometry proteomics data have been deposited to the ProteomeXchange Consortium via the PRIDE partner repository with the dataset identifier PXD044253. The mass spectrometry Phosphoproteomic data have been deposited to the ProteomeXchange Consortium via the PRIDE partner repository with the dataset identifier PXD044254. The data that support the findings of this study are available from the corresponding author upon reasonable request.

## Supporting Information

Supporting Information Table S1: List of the identified proteins in global proteomic analysis.

Supporting Information Table S2: List of significant differentially expressed proteins (DEPs) in MI vs sham.

Supporting Information Table S3: List of significant differentially expressed proteins (DEPs) in TAC vs sham.

Supporting Information Table S4: List of coexpressed proteins in MI and TAC.

Supporting Information Table S5: List of the identified phosphoproteins in global phosphoproteomic analysis.

Supporting Information Table S6: List of significant differentially expressed phosphoproteins (DPPs) in MI vs sham.

Supporting Information Table S7: List of significant differentially expressed phosphoproteins (DPPs) in TAC vs sham.

Supporting Information Table S8: List of coexpressed phosphoproteins in MI and TAC.
