# Supplementary material for: Comparative Proteomic and Phosphoproteomic Analyses Reveal Molecular Signatures of Myocardial Infarction and Transverse Aortic Constriction in Aged Mouse Models
Source: Cardiol Res Pract. 2024 Oct 28;2024:9395213. doi: 10.1155/2024/9395213 (PMC11535427; doi:10.1155/2024/9395213)
Supplement: Supporting Information — Table S1: List of the identified proteins in global proteomic analysis. [file 9395213.f1.pdf]

Majority.protein.IDs	Gene.names	Number.of.proteins	Unique.peptides	Sequence.coverage....	MS.MS.count	Q.value	Score	LFQ.intensity.MI	LFQ.intensity.sham	LFQ.intensity.TAC	iBAQ	Symbol	Rank	fold change(MI/sham)	fold change (TAC/MI)	fold change(TAC/sham)	ENTREZID	Alias
A0A075B5P3;A0A0A6YVP0;P01867-2;P01867	Ighg2b;Igh-3	6	7	39	17	0	21.934	74530000	47345000	30009000	14334000	Ighg2b	462	1.57418946	0.402643231	0.63383673		Ighg2b
A0A075B5P4;A0A0A6YWR2;P01868;P01869	Ighg1	4	2	6.8	1	0.0006028	1.9768	13717000	5121100	5180700	1953600	Ighg1	1077	2.678526098	0.377684625	1.011638125		Ighg1
A0A075B5P6;A0A075B6A0;P01872;P01872-2	Ighm	4	13	29.9	23	0	28.986	97212000	60357000	57008000	11081000	Ighm	526	1.61061683	0.586429659	0.944513478		Ighm
A6YWK5;A0A075B5V2; A0A075B5V0; A0A075B5V7; A0A075B5V6;A0A075B5U4;A0A075B5U6;A0A075B5U7;A0A0A6YY38;A0A0A6YXN5;A0A0A6YX66; A0A0A6YXN4;P06330	Ighv1-31;Ighv1-34;Ighv1-26;Ighv1-43;Ighv1-42;Ighv1-18;Ighv1-20;Ighv1-22	35	1	31.6	6	0	3.6879	39448000	24072000	23214000	15349000	Ighv1-31	449	1.638750415	0.588470898	0.964356929		Ighv1-18;Ighv1-20;Ighv1-31;Ighv1-42;Ighv1-43;Ighv1-22;Ighv1-26;Ighv1-34
A0A087WNP6;Q4VAA2-2;Q4VAA2	Cdv3	3	2	12.8	3	0	5.4383	10998000	2582100	12760000	1714500	Cdv3	1120	4.259323806	1.160210947	4.941714109	321022	Cdv3
A0A087WP24;A0A087WRJ2;A0A087WPF8;A0A087WSR2;E9QN99;Q8VCR7	Abhd14b	6	4	29.6	5	0	3.7559	31103000	5949200	13352000	6625400	Abhd14b	673	5.228097896	0.429283349	2.244335373	76491	Abhd14b
A0A087WPE4;A0A087WQE6;A0A087WNT1;P83940	Tceb1	4	2	43.1	5	0	2.6168	13602000	17446000	14622000	12802000	Tceb1	493	0.77966296	1.074988972	0.838129084	67923	Eloc
087WP48;A0A087WS04;A0A087WS29;A0A087WQG4;A0A087WQD0;A0A087WNW3;A0A087WQI3;A0A087WPW5;A0A087WS23;A0A087WQ25;A0A087WRS1;A0A087WQQ5;A0A087WP85;A0A087WP14;A0A087WPX0;F8VQC7;Q61595-2;Q61595-12;Q61595-16;Q61595-3;Q61595-14;Q61595-6;Q61595-4;Q61595-9;Q61595-8;Q61595-13;Q61595-5;Q61595-15;Q61595-10;Q61595-7;Q61595-11;Q61595	Ktn1	35	8	6.8	12	0	12.973	78840000	75020000	90194000	3603900	Ktn1	864	1.050919755	1.144013191	1.202266062	16709	Ktn1
A0A087WR97;A0A087WRZ5;Q8R0B4; Q8BLD4; Q6VYI5;Q6VYI4;Q921F2; A0A087WRP4;H3BJV1;A0A087WSE4;A0A087WSH7;A0A087WQA5	Tardbp	12	2	8.6	3	0	4.5314	11621000	3399700	3938300	1958300	Tardbp	1075	3.418242786	0.338895104	1.158425743	230908	Tardbp
A0A087WRE7;Q8R0N6-2;Q8R0N6	Adhfe1	3	2	4.6	2	0	4.1471	6048800	10976000	6366200	1368400	Adhfe1	1200	0.551093294	1.052473218	0.580010933	76187	Adhfe1
A0A087WRP7;A0A087WNZ5;P0CW02	Ly6c1	3	1	16.2	2	0.0017544	1.7784	1934700	3727200	2344600	1584600	Ly6c1	1146	0.519075982	1.211867473	0.629051299	17067	Ly6c1
A0A087WRU0;A0A087WQ94;A0A087WQM0;A0A087WQS0; A0A1D5RM59;E9Q0S6;Q9DBT6	Tns1	18	7	4	12	0	9.502	44011000	30494000	35897000	1565200	Tns1	1148	1.443267528	0.815637	1.177182397	21961	Tns1
A0A087WS46;O70251	Eef1b2;Eef1b	6	3	19	7	0	32.259	57650000	36505000	37049000	11699000	Eef1b2	513	1.579235721	0.642653946	1.014902068	55949	Eef1b;Eef1b2
A0A087WS56;Q3UHL6; A0A087WR50;P11276;B7ZNJ1;B9EHT6;A0A087WSN6	Fn1	11	21	12	20	0	54.244	132200000	17490000	22981000	1597600	Fn1	1143	7.558604917	0.173835098	1.313950829	14268	Fn1
A0A0A0MQ90;P97352	S100a13	2	4	25	4	0	9.8342	16443000	6883500	11555000	3941900	S100a13	833	2.38875572	0.702730645	1.678651849	20196	S100a13
A0A0A0MQA5;P68368	Tuba4a	8	4	47.2	17	0	61.002	161170000	190980000	213550000	28202000	Tuba4a	326	0.843910357	1.324998449	1.118179914	22145	Tuba4a
A0A0A0MQF6;P16858;S4R1W1;A0A1D5RLD8; S4R257	Gapdh;Gm3839	13	17	48.2	136	0	117.91	5489500000	5828800000	6489400000	1.122E+09	Gapdh	20	0.941789047	1.182147737	1.113333791	14433	Gapdh;Gm10358;Gm3839
A0A0A0MQM0;P63242;Q8BGY2	Eif5a;Eif5a2	4	5	33.6	14	0	8.5199	94190000	118150000	70591000	38045000	Eif5a	284	0.79720694	0.749453233	0.597469319	276770	Eif5a;Eif5a2
A0A0A6YVU8;Q9JKV1	Gm9774;Adrm1	2	1	3.9	1	0.0059556	1.3883	1307400	1379300	1410000	406630	Gm9774	1480	0.947872109	1.078476365	1.022257667	56436	Adrm1;Adrmlb
A0A0A6YWP4;D6RGQ0;E9Q8H9; E9Q8I0;P06909; A0A0A6YVP8	Cfh	9	4	4.4	5	0	5.6285	18476000	9834800	18321000	1405800	Cfh	1191	1.878635051	0.991610738	1.86287469	12628	Cfh
A0A0A6YY53;F6TQW2;P01864	Ighg2c	3	4	26.6	14	0	25.512	27706000	19414000	12801000	6662000	Ighg2c	669	1.427114453	0.462029885	0.659369527		Ighg2c
A0A0A6YY87;Q8BH58	Tiprl	2	1	23.4	3	0.0033426	1.5613	1786800	1357000	1701900	1285100	Tiprl	1218	1.316728077	0.952484889	1.254163596	226591	Tiprl
[illegible]6	Abcd3	2	2	4.2	3	0	4.5136	2545000	4876400	2561900	425610	Abcd3	1471	0.521901403	1.006640472	0.525367074	19299	Abcd3
A0A0G2JDL9;A0A0G2JE52;P62835;Q99JI6	Rap1a;Rap1b	5	4	41.5	10	0	7.8338	122770000	50846000	94410000	32705000	Rap1a	306	2.414545884	0.768998941	1.856783228	109905	Rap1a;Rap1b
A0A0G2JDV8;Q9DAW9	Cnn3	2	1	7	2	0	2.7758	10750000	3357800	4333600	1335500	Cnn3	1204	3.201500983	0.403125581	1.290606945	71994	Cnn3
G2JGL0;P61079;A0A0G2JF40;Q05CK8;A0A0G2JGZ3;A0A494BAN1; A0A494BA56; Q6ZWY6;P62838	Ube2d3;Ube2d2b; Ube2d2	10	2	13.6	4	0.0006196	2.1606	23619000	19672000	34427000	19457000	Ube2d3	391	1.200640504	1.457597697	1.750050834	66105	Ube2d3;Ube2d2a;Ube2d2;Ube2d2b

A0A0G2JEK2;P63254	Crip1	2	2	14.8	7	0	5.0055	56190000	12011000	20963000	17834000	Crip1	408	4.678211639	0.373073501	1.745316793	12925	Crip1
A0A0G2JER9;G3X8S5;O54946-2;O54946;A0A0G2JGN9;A0A0G2JEI3;Q9QYI8	Dnajb6;Dnajb7	7	2	9.9	4	0	2.9137	4788300	2324200	5029400	1135500	Dnajb6	1263	2.060192755	1.050351899	2.163927373	23950	Dnajb6;Dnajb7
A0A0G2JEX1;Q7TPW1;A0A0G2JG59;A0A0G2JFD8;A0A0G2JGI1;A0A0G2JDY6	Nexn	10	8	11.2	11	0	10.02	59267000	37654000	65638000	5295700	Nexn	738	1.573989483	1.107496583	1.743187975	68810	Nexn
A0A0G2JFB4;A0A0G2JGT5;A0A0G2JH04;P63073	Eif4e	4	3	19.7	5	0	4.4438	8987200	6708100	9528400	3747300	Eif4e	852	1.339753432	1.060218978	1.420432015	13684	Eif4e
A0A0G2JGJ1;Q8BVI4;D3Z099;D3Z1A1;D3YWR7	Qdpr	6	3	16.9	7	0	6.2988	33560000	33311000	36428000	7970300	Qdpr	625	1.007475008	1.08545888	1.093572694	110391	Qdpr
A0A0H2UH17;A0A0G2JDV6;Q80X50-2;Q80X50-3;Q80X50-4;Q80X50;Q80X50-5	Ubap2l	7	1	0.9	2	0.0080257	1.3437	3942200	1519900	2736400	295790	Ubap2l	1516	2.593723271	0.694130181	1.800381604	74383	Ubap2l
A0A0H2UH27;A0A0G2JEP0;Q61584-2;Q61584-5;Q61584-6;Q61584-4;Q61584-7;Q61584-3;Q61584	Fxr1	10	3	7.9	3	0	3.1816	8443200	13622000	15748000	2054100	Fxr1	1054	0.619820878	1.865169604	1.156071062	14359	Fxr1
A0A0J9YKD4		1	1	59.9	3	0	3.2477	4108700	10327000	6331500	1469100	A0A0J9YKD4	1175	0.397859979	1.540998369	0.613101578		Ckm
J9YUL3;Q8C1B7-3;Q8C1B7-2;Q8C1B7	Sept11	14	3	6.8	4	0	4.8077	22603000	4471700	6554000	1526500	Sept11	1160	5.054677192	0.28996151	1.465661829	52398	Septin11
A0A0J9YU70;A0A0J9YV64;Q75WC0;P54099	Polg	4	1	15.9	1	1	-2	5952600	2764100	4525400	3201100	Polg	907	2.153540031	0.760239223	1.6372056	18975	Polg
A0A0J9YUD8;A0A0J9YUZ4;P63158;D3YVC6;D3YZ18	Hmgb1	5	4	27.5	7	0	5.8083	47057000	11266000	22235000	12665000	Hmgb1	497	4.176903959	0.47251206	1.973637493	15289	Hmgb1
A0A0J9YUI1;P61087;F8WIC2;A0A0J9YUR9;Q3V3R8;A0A0J9YU07;D3Z4U3	Ube2k	7	2	21.6	3	0	6.6641	8095600	9060600	7391700	3143700	Ube2k	913	0.893494912	0.913051534	0.8158069	53323	Ube2k
A0A0J9YVC7;D3YWI4;E9Q1I6;F8WHT5;G3UVW1;P19258	Mpv17	6	2	19	4	0	2.8478	7406100	9031100	6585200	7335000	Mpv17	641	0.820066216	0.889158937	0.729169204	17527	Mpv17
0N4SUN8;A0A0N4SWH2;A0A0N4SV32;Q3UMP4;Q9CY58-4;Q9CY58-3;Q9CY58-2;Q9CY58	Serbp1	11	4	37.4	9	0	22.504	25129000	12038000	23513000	8671800	Serbp1	598	2.087473002	0.93569183	1.953231434	66870	Serbp1
A0A0N4SUX5;P17183;D3Z6E4	Eno2	3	1	16.1	3	0	2.9613	21899000	46337000	29095000	6471300	Eno2	678	0.472602888	1.328599479	0.62789995	13807	Eno2
A0A0N4SV66;Q8R1M2;Q64523;Q6GSS7;Q8CGP4;C0HKE9;C0HKE8;C0HKE7;C0HKE6;C0HKE5;C0HKE4;C0HKE3;C0HKE2;C0HKE1;Q8CGP6;Q8CGP7;Q8CGP5;Q8BFU2;Q64522	H2afj;Hist2h2ac;Hist2h2aa1;Hist1h2aa;Hist1h2ah;Hist1h2ak;Hist1h2af;Hist3h2a;Hist2h2ab	20	2	38.9	28	0	13.625	4261900000	1564300000	1831400000	1.629E+09	H2afj	16	2.724477402	0.429714447	1.170747299	232440	H2aj;H2ac1;H2ac11;H2ac12;H2ac13;H2ac15;H2ac20;H2ac21;H2ac25;H2ac4;H2ac6;H2ac7;H2ac8;Hist1h2af;Hist1h2an;Hist1h2ao;Hist1h2ap;Hist2h2aa1;Hist2h2aa2
A0A0N4SVB8;Q9CQW2;F6QKK2;Q8VEH3	Arl8b;Arl8a	4	2	13.8	5	0	2.6874	8583100	7360700	8696500	2937300	Arl8b	939	1.166071162	1.01321201	1.181477305	67166	Arl8a;Arl8b
A0A0N4SVK8;Q8BGK2;E9Q4C0	Adprhl1	4	11	6.5	31	0	81.85	124480000	163490000	198980000	6066800	Adprhl1	689	0.761392134	1.598489717	1.217077497	234072	Adprhl1
A0A0N4SVQ1;Q62425	Ndufa4	2	5	79.6	51	0	32.274	1435600000	2504500000	1941700000	1.712E+09	Ndufa4	14	0.573208225	1.352535525	0.775284488	17992	Ndufa4
A0A0N4SVT3;A0A0N4SW28;Q9DAS9	Gng12	3	2	38.5	5	0	5.06	13984000	6138400	7389100	7129200	Gng12	650	2.278118076	0.528396739	1.203750163	14701	Gng12
A0A0N4SW73;Q8R361	Rab11fip5	2	1	0.8	2	0.0033576	1.5778	1463400	948440	1258400	72751	Rab11fip5	1565	1.542954747	0.859915266	1.326810341	52055	Rab11fip5
A0A0N4SW89;P97429;D3Z0S1;A0A0N4SV57;S4R1F2;F7ANV6	Anxa4	6	7	30.4	12	0	23.324	52510000	14737000	40109000	6018900	Anxa4	693	3.563140395	0.76383546	2.721652982	11746	Anxa4
A0A0N4SW94;O35682	Myadm	2	2	33.3	5	0	4.8597	9683400	6929200	6709600	8440200	Myadm	611	1.397477342	0.692897123	0.96830803	50918	Myadm
A0A0R3P9C8;Q9DC69	Ndufa9	2	16	38.1	56	0	45.005	583320000	960850000	694540000	104660000	Ndufa9	177	0.607087475	1.190667215	0.722839153	66108	Ndufa9
A0A0R4J005;A0A5F8MPJ3;E9QP62;Q99JW4	Lims1	6	3	8.9	5	0	5.915	15341000	8330400	14299000	2350600	Lims1	1018	1.841568232	0.93207744	1.716484202	110829	Lims1
A0A0R4J023;Q9JLZ3;F6RT60	Auh	9	7	22.3	16	0	12.674	73116000	136540000	105740000	20202000	Auh	380	0.535491431	1.446195087	0.774425077	11992	Auh
[illegible]4	Acadl	2	22	47.2	144	0	150.2	2214600000	3842000000	3063600000	528670000	Acadl	49	0.576418532	1.383364942	0.797397189	11363	Acadl
A0A0R4J093;Q9DBP5	Cmpk1	2	2	7.9	3	0	2.3629	7415800	5778400	4998100	1383100	Cmpk1	1196	1.283365638	0.673979881	0.864962619	66588	Cmpk1
A0A0R4J094;Q3TC72	Fahd2	2	3	10.5	5	0	6.6001	15250000	24438000	19667000	5587400	Fahd2	722	0.624028153	1.289639344	0.804771258	68126	Fahd2;Fahd2a
A0A0R4J0I1;P07759	Serpina3k	8	14	40.9	53	0	219.56	347860000	306150000	177500000	50339000	Serpina3k	259	1.136240405	0.510262749	0.579781153	20714	Serpina3k
A0A0R4J0I6;Q80XL6;A0A087WSI8;D3YTQ5	Acad11	5	4	5.4	7	0	8.3232	7350700	13935000	7959700	726600	Acad11	1370	0.527499103	1.082849252	0.571202009	102632	Acad11
A0A0R4J0I9;Q91ZX7	Lrp1	3	5	1.3	4	0	5.8813	20451000	13749000	13311000	211250	Lrp1	1538	1.487453633	0.650872818	0.968143138	16971	Lrp1
A0A0R4J0P1;Q9D7B6;D3YTT4	Acad8	4	10	29.8	13	0	17.503	39790000	63427000	46866000	6795100	Acad8	664	0.627335362	1.177833627	0.738896684	66948	Acad8
A0A0R4J0Q5;P21619;P21619-2	Lmnb2	3	8	15.3	12	0	11.136	13819000	12054000	10583000	1112500	Lmnb2	1268	1.146424423	0.765829655	0.87796582	16907	Lmnb2
A0A0R4J0S3;Q924D0	Rtn4ip1	2	3	8.3	4	0	5.0507	10939000	22079000	7334100	2537500	Rtn4ip1	994	0.495448163	0.670454338	0.33217537	170728	Rtn4ip1
A0A0R4J0T0;Q8K3A0;F6UL52	Hscb	3	2	7.7	2	0.0006242	2.2299	3993700	4570800	4137500	960160	Hscb	1301	0.873742015	1.036006711	0.90520259	100900	Hscb

A0A 0R4J0X5; A0A0A0MQA3;P07758;Q00896	Serpina1a;Serpina1c	5	2	22.3	9	0	5.726	203460000	193290000	156860000	33376000	Serpina1a	303	1.052615241	0.770962351	0.811526722	20702	Serpina1a;Serpina1c
A0A 0R4J0Z1;P08003	Pdia4	2	7	13.3	9	0	10.766	40198000	8712000	18540000	1912600	Pdia4	1081	4.6140955	0.461216976	2.128099174	12304	Pdia4
A0A 0R4J140;Z4YLI8;Q5SW19;Q5SW19-3;Q5SW19-2	Cluh	5	8	6	12	0	12.336	11308000	16825000	13314000	662980	Cluh	1394	0.672095097	1.177396533	0.791322437	74148	Cluh
A0A 0R4J166;Q9D783-2;Q9D783	Klhl40	3	1	1.8	1	0.0080214	1.343	3031600	3424100	2996200	368610	Klhl40	1489	0.885371338	0.988322998	0.875032855	72330	Klhl40
A0A 0R4J1B9;G3X9M0;Q9ER88-2;Q9ER88;G3UWJ4;G3UZC0; G3UWZ6	Dap3	10	3	9.3	3	0	2.7904	4376100	4920900	3479100	724420	Dap3	1371	0.889288545	0.795022966	0.707004816	65111	Dap3
A0A 0R4J1C2;A0A 0R4IZW8; O88456	Capns1	3	2	14	3	0	5.9244	6835300	3378400	4430800	1467400	Capns1	1178	2.023235851	0.648223194	1.311508406	12336	Capns1
A0A 0R4J1C5;Q91YY4	Atpaf2	2	3	10.7	7	0	6.8196	6861300	11693000	7893900	2070200	Atpaf2	1049	0.586786967	1.150496262	0.675096211	246782	Atpaf2
A0A 0R4J1N8; Q8CEE7;D3YVJ8	Rdh13	3	3	12.4	3	0	4.8976	2920300	3062100	3490200	634220	Rdh13	1399	0.953691911	1.195151183	1.139806015	108841	Rdh13
A0A 0R4J1N9; P40630-2;P40630	Tfam	3	2	9.5	3	0	6.9431	8270700	15872000	12114000	3269000	Tfam	892	0.52108745	1.4646886	0.763230847	21780	Tfam
A0A 0R4J1P2; P21107	Tpm3	3	0	40.8	16	0	16.372	623290000	601390000	893740000	141100000	A0A 0R4J1P2	147	1.036415637	1.43390717	1.486123813	59069	Tpm3
A0A 0R4J1R7;Q9CZL5	Pcbd2	2	1	9.7	1	0.0044469	1.5343	3532800	3451200	3545700	1439500	Pcbd2	1183	1.02364395	1.003651495	1.02738178	72562	Pcbd2
A0A 0R4J1Z3;Q9CR67;Q9CZM3	Tmem33	3	2	8.9	2	0	3.8693	7816900	5273900	6059800	1798000	Tmem33	1100	1.482185859	0.775217797	1.149016857	67878	Tmem33
A0A 0R4J275;Q7TMF3	Ndufa12	3	9	75.8	43	0	39.433	190820000	399400000	215190000	109780000	Ndufa12	173	0.47776665	1.12771198	0.538783175	66414	Ndufa12
A0A 0U1RNT6;A0A 0U1RNK6;Q3THS6;A0A 0U1RQ95	Mat2a	4	2	6.1	2	0.010026	1.2934	4206700	3298700	3936500	690750	Mat2a	1383	1.275259951	0.93576913	1.193348895	232087	Mat2a
A0A 0U1RPC6;A0A 0M3HEP9;J3QMN4;A0A 0U1RPV8;A0A 0U1RQA4;J3QMQ8;A0A 0M3HEQ0;Q9JLT4-3;Q9JLT4-2;Q9JLT4-4;Q9JLT4;Q9D8I4; A0A0U1RPS1;D3Z0K8	Txnrd2	14	3	30.9	5	0	5.1101	5083800	10470000	6656500	2695300	Txnrd2	964	0.485558739	1.309355207	0.635768863	26462	Txnrd2
A0A 0U1RPS0;P62743	Ap2s1	2	1	11.7	3	0.0033501	1.5683	9674000	4729600	6518600	5443800	Ap2s1	726	2.045416103	0.673826752	1.378256089	232910	Ap2s1
A0A 140LIF0;Q3TYT1;P97478	Coq7	3	4	18	4	0	11.322	33893000	58838000	29969000	11300000	Coq7	520	0.576039294	0.884223881	0.5093477	12850	Coq7
[illegible]2	Cox7a1	2	2	25.8	16	0	8.442	510360000	1073500000	554530000	538030000	Cox7a1	47	0.475416861	1.086546751	0.516562646	12865	Cox7a1
[illegible]5	Psmc4	2	3	8.3	4	0	4.4602	17500000	19774000	18039000	2521900	Psmc4	1000	0.885000506	1.0308	0.912258521	23996	Psmc4
A0A 140LJB7; Q3MIA8;B1ATU4;G3UXW9;Q99LD4;Q99LD4-2	Gps1	6	2	3.5	2	0	2.4217	7129800	7651300	7197100	1331800	Gps1	1206	0.931841648	1.009439255	0.940637539	209318	Gps1
A0A 1B0GQU8;P35980;A 0A1B0GSF7;A0A1B0GSS8;A 0A1B0GSA8	Rpl18	7	4	28.9	9	0	29.102	76043000	54100000	70338000	35426000	Rpl18	297	1.405600739	0.924976658	1.300147874	19899	Rpl18
A0A 1B0GR11;Q93092	Taldo1	2	4	9.9	8	0	7.3222	65198000	36180000	48696000	9803200	Taldo1	561	1.802045329	0.746894077	1.345936982	21351	Taldo1
A0A 1B0GRV0;Q9Z0S1;D3Z0E6	Bpnt1	4	5	14.6	7	0	6.3821	22187000	20748000	17899000	3207100	Bpnt1	906	1.069356083	0.806733673	0.86268556	23827	Bpnt1
A0A 1B0GSG5;Q91VI7	Rnh1	4	9	22.8	17	0	22.581	52725000	32280000	44596000	5299200	Rnh1	736	1.633364312	0.845822665	1.381536555	107702	Rnh1
A0A 1B0GSK5;A0A 1B0GR19;A0A 1B0GR86; Q8BH97;A 0A1B0GS22	Rcn3	6	4	23.7	4	0	19.372	37400000	439910	1590700	4291500	Rcn3	804	85.01738992	0.042532086	3.615966902	52377	Rcn3
A0A 1B0GSX0;P06151;A 0A1B0GSR9;D3YZQ9;A 0A1B0GT41;A0A1B0GQX5;A0A1B0GSL7	Ldha	16	13	39.1	46	0	36.65	1371300000	1123600000	1460300000	216500000	Ldha	101	1.220452118	1.064901918	1.299661801	16828	Ldha
A0A 1B0GT04;A0A 1B0GS58; Q9CQM9	Glrx3	3	2	16	3	0	3.4255	7822200	7037600	7239000	3113700	Glrx3	916	1.111486871	0.92544297	1.028617711	30926	Glrx3
A0A 1B0GT63;G3X9F4;Q8VD26-2;Q8VD26;A0A1B0GR29;Q8VD26-3	Tmem143	7	5	17	8	0	19.614	15071000	24616000	17206000	3281300	Tmem143	891	0.612244069	1.141662796	0.698976276	70209	Tmem143
A0A 1B0GT92;Q9Z1E4	Gys1	3	5	8	8	0	9.6402	18658000	36206000	22307000	2903500	Gys1	943	0.515328951	1.195572945	0.616113351	14936	Gys1
A0A 1C7CYV0;B1AX58;Q99K51	Pls3	4	10	24.3	12	0	37.061	68257000	11221000	32403000	2983200	Pls3	935	6.082969432	0.474720541	2.887710543	102866	Pls3
A0A 1C7ZMZ5;P46412;A 0A1C7ZMZ7	Gpx3	4	6	32.7	7	0	8.6635	86249000	23441000	27842000	17633000	Gpx3	414	3.679407875	0.32280954	1.187747963	14778	Gpx3
A0A 1D5RLW5;A0A1D5RM85;P62717;A0A 1D5RM79;A0A1D5RME4	Rpl18a	6	3	20.4	8	0	7.125	36805000	23529000	29178000	11828000	Rpl18a	510	1.564239874	0.792772721	1.240086702	76808	Rpl18a
A0A 1D5RMC8;Q3URE1	Acsf3	3	3	7.1	2	0	3.9765	2419400	5558700	2953100	388650	Acsf3	1486	0.435245651	1.220591882	0.531257308	257633	Acsf3
A0A 1L1SQA8;P62852	Rps25	2	3	30.1	9	0	6.098	49681000	25543000	36134000	23250000	Rps25	356	1.944994715	0.727320304	1.414634146	75617	Rps25
A0A 1L1SSA8;Q91XE8	Tmem205	2	2	13.9	2	0	3.0089	6462100	5953300	4775400	1975600	Tmem205	1072	1.085465204	0.738985779	0.802143349	235043	Tmem205
A0A 1L1SSW1;Q8VDC0	Lars2	3	3	6.3	5	0	6.68	4689000	7433700	6746900	848540	Lars2	1338	0.63077606	1.438878226	0.907609939	102436	Lars2
A0A 1L1ST61; A0A1L1SV00;Q8R472;Q9JLR9;A0A 1L1SR69	Higd1a	5	2	35.5	6	0	4.9797	10081000	1948100	11269000	5961700	Higd1a	697	5.174785689	1.117845452	5.784610646	56295	Higd1a
A0A 1L1STC0;Q61081	Cdc37	2	3	8.2	5	0	3.7811	7542900	7019200	14165000	2178000	Cdc37	1035	1.074609642	1.877924936	2.018036243	12539	Cdc37
A0A 1L1STE6;Q9D6R2;Q9D6R2-2	Idh3a	3	17	43.8	84	0	75.952	2012800000	2910000000	2540700000	378550000	Idh3a	71	0.691683849	1.262271463	0.873092784	67834	Idh3a
A0A 1L1SUX8;P01831	Thy1	2	2	19.2	3	0	2.9362	7298500	3261100	3688000	2057900	Thy1	1053	2.238048511	0.50530931	1.130906749	21838	Thy1
A0A 1L1SV25; P57780;E9Q2W9	Actn4	6	14	36.3	21	0	26.241	123460000	29845000	62330000	3789300	Actn4	848	4.136706316	0.504859874	2.088457028	60595	Actn4
A0A 1L1SVG6;Q8BY89-2; Q8BY89	Slc44a2	3	2	3.4	5	0	4.569	10304000	3693300	4802700	766890	Slc44a2	1359	2.789916877	0.466100543	1.300381772	68682	Slc44a2

A0A1L1SVK0;Q61206;A0A1L1SQ76;A0A1L1SRD0;A0A1L1SQV7	Pafah1b2	5	2	14.2	4	0	5.6618	8090200	4548000	6683100	3328100	Pafah1b2	884	1.778847845	0.826073521	1.469459103	18475	Pafah1b2
A0A1W2P6F6;A0A1W2P7Q9;Q60605-2;Q60605;A0A1W2P6G5	Myl6	6	6	39.5	13	0	14.377	150910000	27303000	61867000	26456000	Myl6	340	5.52723144	0.409959579	2.265941472	17904	Myl6
A0A1W2P6P1;Q8BJU0-2;Q8BJU0;A0A1W2P7I5	Sgta	4	2	12.8	2	0	2.5973	2259800	1765900	1846400	575910	Sgta	1419	1.279687412	0.817063457	1.04558582	52551	Sgta
A0A1W2P6Y4;Q8R4U0	Stab2	2	1	1	1	1	-2	58070000	218390	215080	1543400	Stab2	1157	265.9004533	0.003703806	0.984843628	192188	Stab2
A0A1W2P729;Q9QXT0	Cnpy2	2	2	19.1	3	0	3.7742	6567200	2903200	3219600	1370700	Cnpy2	1198	2.262055663	0.490254599	1.108983191	56530	Cnpy2
A0A1W2P7G2;Q9D1J3	Sarnp	3	3	30.7	2	0	3.1951	5673200	3529900	3983100	1887200	Sarnp	1085	1.60718434	0.702090531	1.128388906	66118	Sarnp
A0A1W2P7G5;A0A1W2P7J1;E9Q3H6	Coq10a	7	6	27.6	12	0	12.983	27095000	77108000	37074000	11929000	Coq10a	506	0.351390258	1.368296734	0.480806142	210582	Coq10a
A0A1W2P7X3;Q561M1;Q9D358-2;Q9D358	Acp1	4	2	14	2	0	2.4379	9034600	10027000	7828600	6137100	Acp1	687	0.901027226	0.866513183	0.78075197	11431	Acp1
A0A1Y7VJN8;A0A338P6J9;Q921U7;Q8C281;Q8CE04;Q8CE80;P51125-5;P51125-4;P51125-7;P51125-3;P51125-2;P51125-6;P51125	Cast	13	1	4	2	0	4.1331	3602000	1579000	2830100	476570	Cast	1454	2.281190627	0.785702388	1.792336922	12380	Cast
A0A1Y7VJN9;Q8CFR5;A0A1Y7VL34;Q9D2N4-7;Q9D2N4-4;Q9D2N4-3;Q9D2N4-2;Q9D2N4;Q9D2N4-6;Q9D2N4-5	Dtna	10	2	5.8	2	0	7.4243	3836200	2989900	3749000	295730	Dtna	1517	1.283052945	0.977269173	1.25388809	13527	Dtna
A0A1Y7VJW9;Q9WVH9	Fbln5	2	11	25.6	18	0	49.189	240480000	12174000	27039000	15410000	Fbln5	444	19.75357319	0.112437625	2.22104485	23876	Fbln5
A0A1Y7VJY8;A0A1Y7VM56;Q8K2C6;A0A1Y7VK33	Sirt5	5	7	28	16	0	15.058	24823000	41543000	31840000	6507500	Sirt5	677	0.597525456	1.282681384	0.766434778	68346	Sirt5
A0A1Y7VJZ2;Q9WVL0;A0A1Y7VNC1	Gstz1	3	5	27.4	6	0	9.028	17337000	24952000	24969000	5704000	Gstz1	714	0.694814043	1.44021457	1.000681308	14874	Gstz1
A0A1Y7VK19;Q9DCB8	Isca2	2	4	30.1	7	0	16.76	20102000	35018000	21817000	8899800	Isca2	591	0.574047633	1.085314894	0.623022446	74316	Isca2
A0A1Y7VKT5;Q8BVV6;P59325	Eif5	3	1	27.8	1	0.0028918	1.7156	3724400	2003400	2409800	4218800	Eif5	811	1.859039633	0.647030394	1.202855146	217869	Eif5
A0A1Y7VM65;Q9QUH0	Glrx	2	3	23.8	5	0	5.4459	7411400	13902000	6905400	5362900	Glrx	731	0.533117537	0.931726799	0.496719896	93692	Glrx
A0A286YCI8;Q62417;E9PYX6;Q62417-3	Sorbs1	5	0	19.8	5	0	8.5142	24038000	27129000	27651000	1196700	A0A286YCI8	1250	0.886062885	1.150303686	1.019241402	20411	Sorbs1
A0A286YD34		1	1	37.3	2	0.0054201	1.4009	4307700	4217100	5515300	2127000	A0A286YD34	1042	1.021483958	1.280335214	1.307841882		Sorbs1
A0A286YDC5;A6H611	Mipep	2	2	3.9	2	0	2.5934	2331000	3967300	2367800	283600	Mipep	1522	0.587553248	1.015787216	0.596829078	70478	Mipep
A0A286YDS5;Q99KF1	Tmed9	2	2	9.1	2	0	6.4447	18592000	8697200	9404300	3097800	Tmed9	918	2.137699489	0.505825086	1.081302028	67511	Tmed9
A0A286YE33;A0A286YE75;Q59J78;A0A286YDL9	Ndufaf2	4	2	19.5	3	0.0011723	1.7995	14895000	11335000	14293000	7256700	Ndufaf2	644	1.31407146	0.959583753	1.260961623	75597	Ndufaf2
A0A286YEB7;P62849-2;P62849-3;P62849	Rps24	4	2	22	2	0	3.7372	15580000	10850000	14000000	8414800	Rps24	613	1.4359447	0.898587933	1.290322581	20088	Rps24
A0A2C9F2D2;Q07076	Anxa7	3	13	28.5	23	0	39.818	104800000	54522000	91031000	12408000	Anxa7	500	1.922159862	0.868616412	1.669619603	11750	Anxa7
A0A2I3BPG0;A0A2I3BS14;Q9WVJ3	Cpq	3	2	18.3	2	0	3.7716	25848000	4054700	4012300	6949400	Cpq	657	6.374824278	0.15522671	0.989542999	54381	Cpq
A0A2I3BPG9;P83882	Rpl36a	3	3	23.6	5	0	2.9553	21832000	15418000	18240000	13943000	Rpl36a	470	1.416007264	0.835470868	1.183032819	19982	Rpl36a;Rpl36a-ps1
A0A2I3BR94;A0A2I3BPS1;Q9CQI3;D3Z2F6;D3YY16;Q9ERL7	Gmfb;Gmfg	6	2	14.6	2	0	2.6512	16408000	5446800	7202400	3589700	Gmfb	866	3.012410957	0.438956607	1.322317691	63985	Gmfb;Gmfg
A0A2I3BRL8;Q91VM5;Q9WV02;A2AFI4;A2AFI3;Q9WV02-2	Rbmxl1;Rbmx	8	5	13.1	6	0	7.2963	25017000	9745100	8159600	1771200	Rbmxl1	1106	2.567136304	0.32616221	0.83730285	19656	Rbmx;Gm7324;Rbmxl1
A0A2K6EDJ7;E9Q5L2;E9PVD2;A6X935-2;A6X935	Itih4	5	7	8.4	10	0	10.453	22609000	11448000	26014000	1243000	Itih4	1232	1.974930119	1.150603742	2.272361985	16427	Itih4
A0A2R8VHF9;E9QPE7;A0A338P6K2;O08638-2;O08638	Myh11	5	15	15.4	20	0	26.852	60088000	24582000	40313000	1274000	Myh11	1222	2.444390204	0.670899348	1.639939793	17880	Myh11
A0A2R8VI25;Q3TT81;A0A2R8VHP9;B2M1R7;Q61990-2;Q61990-3;Q61990;A0A2R8VHN3;A0A2R8VHG2;A0A2R8VKF0;A0A2R8VJY1;A0A2R8W6L5;A0A2R8VHZ0;A0A2R8VI71;A0A2R8VHY9;A0A2R8VKN0;A0A2R8VI73;A0A2R8VI12;A0A2R8VHL8;A0A2R8W6U6	Pcbp2	27	4	20.6	10	0	16.359	36758000	24449000	30536000	5605800	Pcbp2	720	1.503456174	0.830730725	1.248967238	18521	Pcbp2
A0A2R8VJV3;Q8R3M1;P61924	Copz1	3	2	25	2	0.0049532	1.4811	1938400	2325200	1819800	1427200	Copz1	1186	0.833648718	0.938815518	0.782642353	56447	Copz1
A0A2R8VJW0		1	1	59.7	3	0	2.4751	3141000	5713200	6317900	5554800	A0A2R8VJW0	723	0.549779458	2.011429481	1.10584261		Aco2
A0A2R8VK70;Q61735;A0A2R8VJU9;A0A2R8VI30;A0A2R8W6P0;Q61735-2	Cd47	8	3	9.3	3	0	2.6171	7474300	2891300	3300100	1198500	Cd47	1248	2.585100128	0.441526297	1.141389686	16423	Cd47

A0A 2R8VKH6;A0A 2R8VHV1;A0A5S8D HC4;Q8CCJ9-5;Q8CCJ9-2;Q8CCJ9-3;Q8CCJ9-1;Q8CCJ9-4;Q8CCJ9	Phf20l1	9	1	1.8	3	0.0011737	1.8044	19527000	13572000	20877000	1640600	Phf20l1	1135	1.438770999	1.069135044	1.538240495	239510	Phf20l1
A0A 338P6I9		1	2	39	2	0	3.4208	938970	2250000	1044300	1284300	A0A 338 P6I9	1219.5	0.41732	1.112176108	0.464133333		Gm56451
A0A 338P7C0;A0A 338P7F1;O09061	Psmb1	3	1	10	3	0.004894	1.439	8371800	4003200	4955700	2759000	Psmb1	957	2.091276978	0.591951552	1.237934652	19170	Psmb1
A0A 338P7E5; P68037;A0A338P786;A0A 338P702	Ube2l3	4	4	32.8	9	0	5.8535	13027000	35478000	28109000	11906000	Ube2l3	507	0.367185298	2.15774929	0.792293816	22195	Ube2l3
A0A 3B2W450;A0A 3B2WCR6;Q62241	Snrpc	3	1	5.7	2	0.0033389	1.5524	5697500	3986600	13607000	3735200	Snrpc	854	1.429162695	2.388240456	3.413184167	20630	Snrpc
W83;A0A 338P7K4; P63325;A 0A338P731	Rps10	11	4	14.6	7	0	5.9464	14203000	10557000	14521000	2812000	Rps10	951	1.345363266	1.022389636	1.37548546	67097	Rps10;Gm49804
A0A 3B2WB82;Q9JK23	Psmg1	2	1	4.9	3	0.0028969	1.7244	1202300	1205000	953840	270590	Psmg1	1525	0.997759336	0.793346087	0.791568465	56088	Psmg1
A0A 3B2WCD8;Q3U0V1	Khsrp	3	3	7.9	5	0	3.8302	23383000	6351900	17804000	1445200	Khsrp	1181	3.681260725	0.76140786	2.802940852	16549	Khsrp
A3B2W7E0;Q8K0D7	Wrb	3	1	11.5	2	0.0033445	1.5621	4230700	5834900	4341300	2069700	Wrb	1050	0.725068125	1.026142246	0.744023034	71446	Get1
A0A 3B2WDB3;Q3UN02;B0V2Q9	Lclat1	3	2	6.5	4	0	3.8852	4449000	4747500	5237700	865070	Lclat1	1332	0.937124803	1.177275792	1.103254344	225010	Lclat1
A3B2WBL1;Q5XJF6;P53026; A0A3B2W820;A0A3B2W824	Rpl10a	6	5	26.8	10	0	7.2127	36144000	28619000	21145000	10670000	Rpl10a	538	1.26293721	0.585021027	0.738844823	19896	Rpl10a
A0A 3Q4EBU5;Q68FL0;G5E8Y0;G3X9J1; P70414;A 0A3Q4L326	Slc8a1	6	6	6.7	12	0	12.896	34009000	30777000	46974000	3475000	Slc8a1	873	1.105013484	1.381222618	1.526269617	20541	Slc8a1
A0A 3Q4EBV4;Q3TJZ6	Fam98a	2	1	4.9	3	0	2.6737	1603100	1247900	1451700	287960	Fam98a	1521	1.284638192	0.905557981	1.163314368	72722	Fam98a
A0A 3Q4EGL6;P56213;A 0A3Q4EII8	Gfer	3	2	12.8	2	0.0006057	2.0116	2400700	2821500	2279300	665000	Gfer	1393	0.850859472	0.949431416	0.807832713	11692	Gfer
A0A 3Q4EH04;A0A 3Q4L335;A0A 3Q4L393;Q8BL97-3;Q8BL97-4;Q8BL97-2;Q8BL97	Srsf7	7	3	32.1	6	0	5.3422	21045000	9030200	15757000	6316800	Srsf7	681	2.330513167	0.748728914	1.744922593	225027	Srsf7
A0A 3Q4EI12; P35293;A0A452J8C1	Rab18	4	6	33	10	0	20.666	26292000	18171000	23918000	5702700	Rab18	715	1.446920918	0.909706375	1.316273183	19330	Rab18
A0A 494B945; Q9D1G1	Rab1b	2	1	22.8	3	0	3.1796	11559000	10030000	12592000	2423700	Rab1b	1010	1.152442672	1.089367592	1.255433699	76308	Rab1b
A0A 494B952; Q91V64	Isoc1	2	2	11.3	4	0	4.2681	6440200	8730300	5401700	1855000	Isoc1	1087	0.737683699	0.838747244	0.61873017	66307	Isoc1
A0A 494BAH5;Q9CQF7; Q9CWM4;A0A494BBK3	Pfdn1	4	2	17.3	1	0	2.9033	5290700	4305000	4413700	2799800	Pfdn1	952	1.228966318	0.834237436	1.02524971	67199	Pfdn1
A0A 494BAJ6;Q9WUL7	Arl3	2	1	6.1	1	0.0099685	1.2806	4055500	3172400	2884200	939490	Arl3	1310	1.278369689	0.711182345	0.909153953	56350	Arl3
A0A 494BAX5;A0A 498WGK2;Q8BP47; A0A 494BB89	Nars	5	3	5.6	8	0	6.114	7359700	6266000	7531600	1293400	Nars	1215	1.174545164	1.02335693	1.201978934	70223	Nars;NARS1
A0A 494BB86; P61164;E0CZD4;E0CYB4; Q8R5C5	Actr1a;Actr1b	5	2	4.8	2	0.000591	1.8429	8998900	5075200	8573800	1230800	Actr1a	1240	1.77311239	0.952760893	1.689352144	54130	Actr1b;Actr1a
A0A 494BBB0;O35350	Capn1	4	5	8.3	4	0	5.9708	7138200	4061900	3416000	348390	Capn1	1499	1.757354932	0.478552016	0.840985746	12333	Capn1
A0A 494BBI1; A0A494B955;A0A498WGD8;Q8CDN6;A 0A494BB31	Txnl1	5	4	20.6	9	0	6.5817	20881000	13955000	17206000	4500400	Txnl1	785.5	1.496309566	0.824002682	1.232963096	53382	Txnl1
A0A 494BBN2;Q9D1B9	Mrpl28	2	1	3.6	1	0.0006035	1.983	2414400	3465400	2312000	862070	Mrpl28	1334	0.696716108	0.957587806	0.667166849	68611	Mrpl28
A0A 571BDS0;E9QQ93;O70373	Xirp1	3	17	12	28	0	37.686	55411000	20722000	101060000	1547200	Xirp1	1156	2.674017952	1.823825594	4.87694238	22437	Xirp1
A0A 571BEC9;O88492;O88492-2	Plin4	3	17	42.6	34	0	40.193	145840000	163500000	161050000	4470100	Plin4	789	0.891987768	1.104292375	0.985015291	57435	Plin4
A0A 571BEL9; P80317	Cct6a	4	7	12.2	14	0	10.859	41718000	39300000	44024000	5912700	Cct6a	702	1.061526718	1.0552759	1.120203562	12466	Cct6a
A0A 571BG24; D3YU59;D3Z589;A0A5F8MPN3;D3YU22;Q3UH68-3;Q3UH68-2;Q3UH68	Limch1	9	5	4	8	0	7.0732	23194000	20587000	27760000	1185500	Limch1	1252	1.126633312	1.196861257	1.348423763	77569	Limch1
A0A 5F8MPB9;P26043	Rdx	2	3	18	7	0	6.475	26315000	16136000	18568000	2086100	Rdx	1047	1.630825483	0.705605168	1.150718889	19684	Rdx
A0A 5F8MPJ2;D3Z5J3;A0A286YCQ0;A0A286YDN0;E9Q6A3;Q62417-5;Q62417-6;Q62417-4	Sorbs1	9	3	23.5	49	0	61.244	112310000	131860000	153880000	7871200	A0A 5F8 MPJ2	629	0.85173669	1.37013623	1.166995298	20411	Sorbs1
A0A 5F8MPK9;Q9EP69	Sacm1l	2	6	9.4	7	0	7.1104	21451000	10507000	25329000	1518700	Sacm1l	1161	2.04159132	1.180784113	2.410678595	83493	Sacm1l
A0A 5F8MPR4;Q4U4S6-2;Q4U4S6	Xirp2	7	32	10.3	40	0	58.737	21624000	3753700	225400000	932600	Xirp2	1313	5.760716093	10.4236034	60.04741988	241431	Xirp2
A0A 5F8MPU6;G3UWE1;A0A5F8MQC8;Q52L67;Q9CY27	Tecr	8	3	15	10	0	5.6575	9793500	10670000	9862100	2623800	Tecr	978	0.917853796	1.007004646	0.924283037	106529	Tecr
A0A 5H1ZRK8;P01837		2	2	23.4	4	0	4.5266	34176000	5829200	7686200	11730000	A0A 5H 1ZRK8	512	5.862897139	0.224900515	1.318568586		Igkc
A0A 5K1VVQ9		9	0	37	2947	0	323.31	1.7374E+10	2.6309E+10	2.6285E+10	40535000	A0A 5K 1VVQ9	277	0.660382379	1.512892828	0.999087765		Ttn
A1BN54; Q7TPR4	Actn1	2	15	40.4	29	0	80.779	285180000	76530000	131880000	9543600	Actn1	573	3.726381811	0.462444772	1.723245786	109711	Actn1
A2A 547;P84099	Rpl19	2	5	25.8	7	0	7.1426	56778000	39373000	63732000	28235000	Rpl19	325	1.4420542	1.122477016	1.618672694	19921	Rpl19
A2A 6G6;A2A 6G7; A2A 6G8;A2A 6G9; A2A 6H0; Q61792;A2A6H1	Lasp1	9	3	36	3	0	7.2671	22402000	10210000	12400000	9092400	Lasp1	587	2.194123408	0.553522007	1.214495593	16796	Lasp1
A2A 6T4;Q8R035-2;Q8R035	Ict1	3	2	11.8	2	0	2.4301	1050300	1985500	985720	537550	Ict1	1435	0.528985142	0.938512806	0.49645933	68572	Mrpl58
A2A 6U5; A2A6U3; Q80UG5-2;Q80UG5-3;Q80UG5	Sept9	5	2	8.3	2	0	3.2402	4160800	2877400	4092900	760860	Sept9	1361	1.446027664	0.983681023	1.422429972	53860	Septin9

A2A7S7;Q91WQ3;F6VXZ2	Yars	3	2	3	3	0	2.489	6768200	5549000	7172200	486320	Yars	1450	1.219715264	1.059690907	1.292521175	107271	Yars;Yars1
A2A8U2-2;A2A8U2-3;A2A8U2	Tmem201	3	1	2.3	2	0.0028868	1.7094	1642400	1361000	1841000	392320	Tmem201	1485	1.206759735	1.120920604	1.352681852	230917	Tmem201
A2ACG7;Q9DBG6	Rpn2	2	7	15.1	8	0	21.668	31337000	8578600	16088000	2179900	Rpn2	1034	3.652927051	0.513386731	1.875364279	20014	Rpn2
A2ACV6;E1U8D0	Soga1	2	1	0.6	3	0.0090234	1.3111	27003000	57607000	43277000	1585100	Soga1	1145	0.468745118	1.602673777	0.751245508	320706	Soga1
A2AEW9;A2AEW8	Gripap1	2	1	1.6	2	0.003337	1.5486	9318500	11789000	8225300	752020	Gripap1	1362	0.790440241	0.882684981	0.697709729	54645	Gripap1
A2AEX8;A2AEX6;P97447;A2AEY1;A2AEY2;A2AEX7;P97447-2	Fhl1	10	10	38.5	29	0	31.346	104060000	38213000	222500000	18510000	Fhl1	405	2.723157041	2.138189506	5.822625808	14199	Fhl1
A2AG39;F6WI02;Q99JB2	Stoml2	3	2	9.4	2	0.0033689	1.5948	6222600	5888800	6386000	1448200	Stoml2	1179	1.056683874	1.02625912	1.084431463	66592	Stoml2
A2AGN7;B7ZCF1;O88685;F6Q2E3;A0A087WPH7	Psmc3	5	2	4.8	5	0	4.3856	17639000	13738000	19526000	1963300	Psmc3	1074	1.283956908	1.106978854	1.421313146	19182	Psmc3
A2AH25;Q5FWK3	Arhgap1	2	4	9.6	3	0	6.1266	15037000	9744800	10286000	1232700	Arhgap1	1238	1.543079386	0.68404602	1.055537312	228359	Arhgap1
A2AJY2;O35206;A2AJY7;A2AJY5	Col15a1	4	13	10.9	22	0	22.014	132480000	49363000	80237000	5975000	Col15a1	695	2.683791504	0.605653684	1.62544821	12819	Col15a1
A2AKD7;Q61234	Snta1	2	2	6.6	3	0	6.349	8668200	7544700	11058000	1253900	Snta1	1228	1.148912482	1.275697377	1.465664639	20648	Snta1
A2AMM0	Murc	1	7	25.4	16	0	26.677	36042000	52301000	47680000	11217000	Murc	522	0.689126403	1.32290106	0.911646049	68016	Cavin4
A2AP32;Q3UIU2	Ndufb6	2	5	43.3	21	0	11.951	168530000	212500000	153680000	89743000	Ndufb6	196	0.793082353	0.911885124	0.7232	230075	Ndufb6
A2APX3;P21460	Cst3	2	2	26.5	2	0	3.0497	7190800	1088100	2522200	1719300	Cst3	1117	6.60858377	0.350753741	2.317985479	13010	Cst3
A2AQR0;Q64521	Gpd2	2	4	6.3	4	0	4.2776	6241100	7746600	8212700	581730	Gpd2	1416	0.805656675	1.31590585	1.060168332	14571	Gpd2
A2ASS6-3	Ttn	1	8	24	9	0	11.26	17006000	17244000	18137000	206160	A2ASS6-3	1539	0.986198098	1.066505939	1.051786129		
A2ASS6;E9Q8K5;E9Q8N1	Ttn	4	0	37.2	4	0	3.1197	40245000	58739000	56770000	89425	A2ASS6	1564	0.68514956	1.410610014	0.96647883	22138	Ttn
A2AST1;E9Q8Q6	Ccdc141	2	6	4.4	5	0	6.1511	6760500	4881500	6522800	244040	Ccdc141	1532	1.384922667	0.964839879	1.336228618	545428	Ccdc141
A2AUC9	Klhl41	1	7	12.9	15	0	23.079	33120000	35325000	64963000	4500200	Klhl41	787	0.937579618	1.961443237	1.8390092	228003	Klhl41
A2AVJ7;Q99PL5	Rrbp1	13	16	11.6	21	0	35.989	91045000	19994000	34264000	1390700	Rrbp1	1194	4.553616085	0.37634137	1.713714114	81910	Rrbp1
A2BE92;A2BE93;Q9EQU5-2;Q9EQU5	Set	5	3	23.2	4	0	7.9981	19859000	8375400	11016000	6775400	Set	665	2.371110634	0.554710711	1.315280464	56086	Set
A2BFF8;Q3TPJ8;A2BFF5;A2BFF9;O88487	Dync1i2	5	2	3.1	4	0	3.4521	25626000	10906000	25618000	3047600	Dync1i2	925	2.349715753	0.999687817	2.348982212	13427	Dync1i2
A2CG35;P35283	Rab12	2	5	17.5	7	0	10.888	8172000	9924100	10270000	1612900	Rab12	1140	0.823449985	1.256730299	1.034854546	19328	Rab12
A3KFM7-2;A3KFM7	Chd6	2	1	0.4	4	0.0090329	1.3177	93387000	183550000	161220000	3864300	Chd6	840	0.508782348	1.726364483	0.878343776	71389	Chd6
A3KGU9;A3KGU7;E9Q447;A3KGU5	Sptan1	6	3	37.2	172	0	291.15	803270000	622060000	679100000	15717000	Sptan1	437	1.291306305	0.845419348	1.091695335	20740	Sptan1
A3KMP2-2;A3KMP2	Ttc38	2	2	7.2	3	0	4.1187	5693700	8263700	5197700	1502200	Ttc38	1163	0.689001295	0.912886172	0.628979755	239570	Ttc38
A6H644;Q8BG95	Ppp1r12b	4	9	12.6	17	0	20.603	30373000	33176000	36481000	2014800	Ppp1r12b	1063	0.915511213	1.201099661	1.099620207	329251	Ppp1r12b
A6X940	Fermt2	1	1	12	3	0	2.3582	5286000	2759600	5330700	1118400	A6X940	1266	1.915494999	1.0084563	1.931692999		Fermt2
A8DUK4;P02088;E9Q223	Hbbt1;Hbb-b1;Hbb-bs	6	8	95.2	358	0	296.23	1.3721E+10	1.4717E+10	1.3876E+10	4.324E+09	Hbbt1	3	0.932323164	1.011296553	0.942855201	100503605	Hbb-bs;Hbb-b1
B0QZL1	Eno1	1	1	48.9	3	0	5.1061	24223000	27377000	31158000	8964600	B0QZL1	590	0.884793805	1.286298146	1.138108631		Eno1
B0R1E3;P70349	Hint1	2	3	34.5	7	0	9.6172	80923000	111630000	101300000	46623000	Hint1	268	0.724921616	1.251807274	0.907462152	15254	Hint1
B1AQF4;Q9D7X3;H3BKL8	Dusp3	5	3	17.6	5	0	8.192	17245000	12288000	22483000	5259800	Dusp3	739	1.403401693	1.303740215	1.829671224	72349	Dusp3
B1ASZ3;Q64516-1;Q64516-2;Q64516	Gyk;Gk	6	9	18.5	12	0	13.313	36667000	31312000	46218000	4131700	Gyk	817	1.171020695	1.26047945	1.476047522	14933	Gk
B1AT36;Q9D8W5;Q3TRH2	Psmd12	3	5	11	6	0	7.2473	12042000	8645100	13501000	1500100	Psmd12	1165	1.392927786	1.121159276	1.561693908	66997	Psmd12
B1AU25;Q9Z0X1	Aifm1	3	17	35	40	0	73.123	209470000	361310000	243150000	31673000	Aifm1	309	0.57975146	1.160786748	0.672967812	26926	Aifm1
B1AV77;B1ATI0;P47740	Aldh3a2	3	1	1.6	2	0.0006039	1.9981	4058200	1741100	3377000	460010	Aldh3a2	1461	2.33082534	0.832142329	1.939578427	11671	Aldh3a2
B1AXC8;Q8K2Q5;Q3TFT3;B1AXC7;B1AXC5	Chchd7	5	2	34.2	2	0	6.2439	940010	8245200	1100800	3026100	Chchd7	929	0.114006937	1.171051372	0.13350798	66433	Chchd7
B1AXP6-2;B1AXP6;B1AXP6-3;B1AXP6-4	Tomm5	4	3	51.2	3	0.0006109	2.0665	20465000	25382000	26191000	20414000	Tomm5	378	0.806280041	1.279794772	1.031872981	68512	Tomm5
B1AZ26;B1AZ15;B1AZ14;Q3UMF0-4;Q3UMF0-2;Q3UMF0-3;Q3UMF0	Cobll1	7	2	5.8	2	0.0049207	1.4531	1068900	1400800	1427100	203950	Cobll1	1542	0.763063963	1.335110862	1.018774986	319876	Cobll1
B1AZS9;O08807	Prdx4	2	1	8.3	1	0	2.5356	41582000	40527000	38816000	9545900	Prdx4	572	1.026032028	0.933480833	0.957781232	53381	Prdx4
B1B1A8;Q6PDN3-2;Q6PDN3-3;Q6PDN3	Mylk	4	2	1	4	0	3.1188	7890400	9216800	7332000	510340	Mylk	1443	0.856088881	0.929230457	0.795503863	107589	Mylk
B2KGR2;O89112	Lancl1	2	2	11.6	2	0.0006131	2.0786	5895200	2519700	3663000	1244100	Lancl1	1231	2.339643608	0.621352965	1.453744493	14768	Lancl1
PVD5;A0A0G2JGM2;A0A0N4SVY1;C0H5X4;A0A0G2JE25;A0A0G2JGP4;Q9D091;Q61411-2;P32883-2;Q61411;P32883;P08556	Kras;Nras;Hras	14	1	32.4	1	0.0017513	1.7606	7174100	4454500	5180100	8800200	Kras	593	1.610528679	0.722055728	1.162891458	18176	Nras;Kras;Hras
B2M1R6;P61979-3;P61979;P61979-2;H3BLL4;A0A286YDM3;H3BK96;H3BKD0;H3BKI8	Hnrnpk	17	13	36.4	31	0	46.943	250970000	111780000	148150000	21126000	Hnrnpk	373	2.245213813	0.590309599	1.325371265	15387	Hnrnpk
B2RQK7;Q8BWB1;D3YU08	Synpo2l	3	14	19.3	22	0	31.756	52600000	18392000	121660000	3997300	Synpo2l	831	2.859939104	2.312927757	6.614832536	68760	Synpo2l
B2RXT3;E9Q7L0	Ogdhl	2	20	29.9	43	0	58.787	151500000	322700000	165560000	16163000	Ogdhl	429	0.469476294	1.092805281	0.513046173	239017	Ogdhl
B2Z893;B2Z892;Q6GTL7;A0A087WRG0;A0A087WRM7;P70265;B2Z894	Pfkfb2	24	3	5.9	2	0	2.9383	3472400	2854000	3205000	292190	Pfkfb2	1519	1.216678346	0.922992743	1.122985284	18640	Pfkfb2;Gm29427
B7FAV1;B7FAU9;Q8BTM8	Flna	7	31	17.8	38	0	76.518	226100000	22324000	107510000	2522700	Flna	999	10.12811324	0.475497567	4.815893209	192176	Flna
B7ZBY7;E9PY39;Q9D2M8;Q9CZY3;A6X925;B7ZBY6;Q9D2M8-2;Q9CZY3-2;B2KF55	Ube2v1;Gm20431;Ube2v2	9	4	20.8	8	0	6.3615	41928000	44004000	48699000	12103000	Ube2v1	503	0.952822471	1.161491128	1.106694846	66589	Ube2v1;Ube2v2;Gm20431
B7ZCA9;Q5SSK3	Tefm	2	1	5.9	1	0.0005879	1.8213	1236400	1416700	1344600	345500	Tefm	1501	0.872732406	1.087512132	0.94910708	68550	Tefm

B7ZCI2;B7ZCI3;A0A1B0GXL1;Q0II04	Nebl	7	36	44.4	85	0	136.89	494530000	672090000	665400000	29742000	Nebl	318	0.735809192	1.345519989	0.990045976		Nebl
B7ZNL2;Q78ZA7	Nap1l4	4	4	17.6	9	0	7.171	28693000	5461300	14260000	2771500	Nap1l4	954	5.253877282	0.496985327	2.611099921	17955	Nap1l4
B7ZNU9;Q61235	Sntb2	2	2	4.1	2	0.0006098	2.0567	4268500	1844400	2873000	345230	Sntb2	1502	2.314302754	0.673070165	1.557688137	20650	Sntb2
B8JJG9;B8JJG7;B8JJG8;Q9Z1A1	Tfg	4	1	11.2	2	0.0028802	1.6976	9333100	5302000	8313700	4721200	Tfg	771	1.760298001	0.890775841	1.568030932	21787	Tfg
B8JK33;B8JK32;Q9D0E1-2;Q9D0E1	Hnrnpm	7	7	12.8	9	0	10.451	43981000	13935000	14607000	1663300	Hnrnpm	1130	3.15615357	0.332120688	1.048223897	76936	Hnrnpm
D3YU60;E9QJW0;Q91VS7	Mgst1	3	1	12.7	2	0.0011751	1.8121	6758800	3073000	6953600	3306500	Mgst1	888	2.199414253	1.028821684	2.262805076	56615	Mgst1
D3YUM1;Q91YT0;D3Z1U9	Ndufv1	8	23	59.8	88	0	136.78	868120000	1349200000	917700000	173220000	Ndufv1	126	0.643433146	1.05711192	0.680180848	17995	Ndufv1
D3YVS7;Q8K353	Cystm1	2	1	11.9	1	0.007983	1.3302	2222200	1391600	1748000	5752300	Cystm1	711	1.596866916	0.786607866	1.256108077		Cystm1
D3YVV9; E9Q1U2;Q91YE8;A0A498WGE8;Q91YE8-2	Synpo2	5	3	3.5	3	0	3.8101	7470900	10098000	8142600	503510	Synpo2	1446	0.739839572	1.089908846	0.806357695	118449	Synpo2
D3YVZ9; P58059	Mrps21	2	1	29.5	2	0.0006068	2.0172	2361400	4181800	3230200	5639900	Mrps21	717	0.564685064	1.367917337	0.772442489	66292	Mrps21
D3YWD6;D3YVQ5;A0A1B0GRF7;D3YTK6;A0A1B0GRY5;Q8R104-2;Q8R104	Sirt3	7	1	11	2	0	2.6077	2534300	3210300	3686600	1704900	Sirt3	1121	0.789427779	1.454681766	1.148366196	64384	Sirt3
D3YWF6;Q7TQI3; D3Z7K0	Otub1	3	6	29.9	15	0	12.1	30821000	23919000	31281000	6939600	Otub1	658	1.288557214	1.014924889	1.307788787	107260	Otub1
D3YWY6;D3Z5S0;E9Q0V4;P63030;D3Z786	Mpc1	5	3	20	8	0	4.4509	180360000	267090000	232560000	156820000	Mpc1	135	0.675277996	1.289421158	0.870717736	55951	Mpc1
D3YX34;E9Q586;E9Q3M3;O08788-2;O08788	Dctn1	5	3	3	4	0	5.9484	5807800	4880500	6394400	349820	Dctn1	1498	1.190001024	1.101002101	1.310193628	13191	Dctn1
D3YX62;O70252;D3YXN4	Hmox2	3	2	8.8	1	0.0028885	1.7128	5260700	4262100	5715200	1068100	Hmox2	1277	1.234297647	1.086395347	1.34093522	15369	Hmox2
D3YY36;Q9DBD0;F6W4D3	1300017J02Rik;Ica	3	1	3.1	1	0.0006258	2.2518	2920000	1950100	1953800	205720	1300017J02Rik	1540	1.49735911	0.669109589	1.001897339	71775	Inhca;Ica
D3YYD5;D3Z645;Q9QZ88;Q9QZ88-2	Vps29	5	3	25	4	0	3.5979	12822000	7877800	20091000	3714100	Vps29	855	1.62761177	1.566916238	2.550331311	56433	Vps29
D3YYE1;D3Z7M9; O35381;F6UFG6	Anp32a	5	5	37.9	13	0	47.259	57374000	41598000	31226000	15840000	Anp32a	435	1.379249002	0.544253495	0.750661089	11737	Anp32a
D3YYK8;E9Q6X0;Q8R001-2;Q8R001;Q3TG90	Mapre2	5	2	6.5	3	0.0006046	2.0086	12002000	10918000	20275000	2624200	Mapre2	977	1.099285583	1.689301783	1.857025096	212307	Mapre2
D3YYN7;Q6PIE5;A0A0G2JGX4;Q8VCE0;Q6PIC6	Atp1a2;Atp1a3	6	11	32.2	18	0	28.647	44593000	51119000	46494000	3393800	Atp1a2	880	0.872337096	1.042630009	0.909524834	98660	Atp1a3;Atp1a2
D3YYT0; P15116	Cdh2	2	6	10.2	9	0	23.277	9723400	28225000	24632000	2159700	Cdh2	1037	0.344496014	2.533270255	0.872701506	12558	Cdh2
D3Z041;P41216	Acsl1	7	34	52.9	114	0	147.44	1203000000	2296900000	1484400000	158240000	Acsl1	132	0.523749401	1.233915212	0.646262354	14081	Acsl1
D3Z0I3	Myl2	1	1	79.6	4	1	-2	39086000	122140000	168380000	50233000	D3Z0I3	260	0.320009825	4.307936345	1.378581955		Myl2
D3Z0S0;D3YVI6;D3Z440;Q9CZ04;Q9CZ04-2	Cops7a	5	1	17.7	2	0.001173	1.7999	1539500	1723300	1209100	1282200	Cops7a	1221	0.893344165	0.785384865	0.701618987	26894	Cops7a
D3Z0T8;D3Z032;E9Q1L7;Q8VCE1	Dnajc28	4	1	16.7	1	0	2.6184	1021000	1482000	755620	777080	Dnajc28	1356	0.688933873	0.740078355	0.509865047	246738	Dnajc28
D3Z0U5;D6RGM7;D3Z4J2;A0A0G2JFY0;A0A0A0MQ80;Q9D3R6-3;Q9D3R6;Q3UMC0	Katnal2;Spata5	8	1	4.3	3	0	2.5014	18717000	17991000	18208000	4294100	Katnal2	803	1.04035351	0.972805471	1.012061586	57815	Katnal2;Spata5
D3Z158;Q8BML9	Qars	10	5	6.3	7	0	6.7204	12699000	12650000	10374000	921400	Qars	1318	1.003873518	0.816914718	0.820079051	97541	Qars;Qars1
D3Z2Z1;F8WIA1;Q922J3;D3Z3M7;Q922J3-2;Q8C0S5;F6RCU2	Clip1	10	10	7.4	12	0	18.593	22663000	13374000	27448000	689100	Clip1	1384	1.694556602	1.211137096	2.052340362	56430	Clip1
D3Z368;Q91YS8	Camk1	2	1	6.2	3	0.0006165	2.1216	3751500	2340200	2859800	1185300	Camk1	1253	1.603068114	0.76230841	1.222032305	52163	Camk1
D3Z390;Q921C5;Q921C5-2;Q921C5-3	Bicd2	4	2	2.8	2	0	2.3657	3824300	4515800	3481200	335820	Bicd2	1506	0.846870986	0.910284235	0.770893308	76895	Bicd2
D3Z4C9;Q9CQY6;D3Z4D6	Uqcc2	3	3	28.3	5	0	7.1404	8277000	9067300	5243400	4453000	Uqcc2	791	0.912840647	0.633490395	0.578275782	67267	Uqcc2
D3Z598;Q8K4G1-2;E9Q6B8;E9QPS5;Q8K4G1-3;Q8K4G1	Ltbp4	7	6	5.1	6	0	9.5114	20566000	4547100	4987500	404280	Ltbp4	1483	4.522882716	0.242511913	1.096852939	108075	Ltbp4
D3Z5B1;B2RPU8;Q9D1L0	Zbed5;Chchd2	3	3	25.7	6	0	8.4157	18900000	21349000	18632000	8454400	Zbed5	610	0.885287367	0.985820106	0.872734086	71970	Chchd2l;Chchd2
D3Z636;Q91VM9;G8JL76;Q91VM9-2	Ppa2	4	8	30.7	19	0	49.166	39191000	71066000	48424000	9796900	Ppa2	563	0.551473278	1.235589804	0.68139476	74776	Ppa2
D3Z637;D3Z125;E9PUA7;D3Z7X7;F8WHQ1;Q62393-2;Q62393-3;Q62393	Tpd52	8	1	6.8	1	0	3.1612	4387000	825430	1659200	1083300	Tpd52	1273	5.314805616	0.378208343	2.010103825	21985	Tpd52
D3Z6I8;E9Q7Q3	Tpm3	2	0	46.6	5	0	17.234	28179000	5526500	10816000	2626100	D3Z6I8	976	5.09888718	0.383831932	1.957115715	59069	Tpm3
D3Z6Z0;Q78ZM0;O70492;D3Z789	Snx3	4	3	22.9	5	0	5.135	9687200	5532300	6851600	2896500	Snx3	945	1.751025794	0.707283838	1.238472245	54198	Snx3
D3Z7C6;Q9R0Q7	Ptges3	2	4	34.6	5	0	8.1588	30260000	21609000	24102000	10204000	Ptges3	548	1.40034245	0.796497026	1.115368596	56351	Ptges3
D3Z7E5;Q2NL51;E9QAQ5;Q9WV60	Gsk3a;Gsk3b	4	2	4.5	3	0	3.12	5883900	4199400	5558600	822900	Gsk3a	1342	1.401128733	0.94471354	1.323665286	606496	Gsk3a;Gsk3b
3;Q8BFS6-4;Q8BFS6-2;Q8BFS6	Cpped1	5	1	4.6	1	0	2.5566	2534500	2023800	4022400	949540	Cpped1	1304	1.25234707	1.587058591	1.987548177	223978	Cpped1
D3Z7P4;D3Z7P3-2;D3Z7P3;F6U529	Gls	4	3	6.7	3	0	4.2521	6386300	3373100	5034000	611870	Gls	1406	1.893302896	0.788249847	1.492395719	14660	Gls
D3Z7X0;D3Z2B3	Acad12	3	7	31.4	40	0	31.94	298470000	274850000	216330000	27557000	Acad12	331	1.085937784	0.724796462	0.787083864	338350	Acad12
D6REG4;Q91Z53	Grhpr	3	4	24.4	9	0	10.114	37354000	39755000	54588000	14295000	Grhpr	463	0.939605081	1.461369599	1.373110301	76238	Grhpr
D6RFA9;D6RHQ2;Q8CDV7;P55772	Entpd1	4	1	30.2	1	0	2.8322	1684700	852740	903720	915690	Entpd1	1319	1.975631494	0.536427851	1.059783756	12495	Entpd1
BC3-2;Q05BC3;Q05BC3-3	Eml1	5	3	4.9	4	0	4.6798	2578600	2454600	2607800	260260	Eml1	1526	1.050517396	1.011323974	1.062413428	68519	Eml1
D6RJ83;A6PWX5; F6V305;A6PWX8;A6PWX9;Q9CWU6	Uqcc1	6	1	10	1	0	3.1105	2464600	3006800	2936300	1555400	Uqcc1	1153.5	0.819675402	1.191390084	0.976553146	56046	Uqcc1
D9J301;D9J302;D9J2Z9; D9J300	Pdlim5	6	1	24.9	30	0	56.089	340660000	373040000	300850000	36346000	D9J301	291.5	0.913199657	0.883138613	0.806481879	56376	Pdlim5
D9J303;F8WJI6;E9Q8P5;Q8CI51-3;Q9CRA2	Pdlim5	5	0	45.3	5	0	4.745	5685100	13183000	4765300	1755200	D9J303	1109	0.431244785	0.838208651	0.361473109	56376	Pdlim5
E0CXB9;Q61301-2;Q61301-3;Q61301	Ctnna2	5	1	9.1	5	0	5.0155	5246200	6065900	7046500	414530	Ctnna2	1474	0.864867538	1.34316267	1.161657792	12386	Ctnna2
E0CXN5; P13707	Gpd1	2	5	19.9	6	0	5.5685	27579000	45419000	36161000	3992300	Gpd1	832	0.60721284	1.311178795	0.796164601	14555	Gpd1

E0CZ27;F8WI35;A0A1W2P768;P84244;P84228;P68433;P02301	H3f3a;Hist1h3b;Hist1h3a;H3f3c	9	3	19.3	16	0	3.3918	1914400000	866470000	1171100000	1.062E+09	H3f3a	24	2.209424446	0.611732135	1.351575935	15078	H3f3a;H3-3a; H3-3b;H3-5;H3c1; H3c8; H3c10; H3c11;H3c14; H3c2; H3c3; H3c4; H3c6; H3c7; H3c13; H3c14; H3c15
E9PUD2;Q8K1M6-4;Q8K1M6-3;Q8K1M6-2;Q8K1M6;A0A2U3TZ67	Dnml1	7	11	20.1	20	0	43.481	32610000	46562000	30303000	2667900	Dnml1	971	0.700356514	0.92925483	0.650809673	74006	Dnml1
E9PUE8;P70170-3;P70170-2;P70170	Abcc9	4	2	1.6	1	0.0048886	1.4266	2188200	4947100	2125500	156440	Abcc9	1553	0.442319743	0.971346312	0.429645651	20928	Abcc9
E9PUM4; A0A1L1SQ51;Q71LX4;Q8CDM9;A0A1L1SRI1	Tln2	6	17	12	27	0	24.891	95260000	52004000	67450000	1615800	Tln2	1139	1.831782171	0.708062146	1.297015614	70549	Tln2
E9PUT1;Q91YM4-2;Q91YM4	Tbrg4	3	1	2.7	2	0.0005992	1.9322	2140900	3540300	3392000	461510	Tbrg4	1460	0.604722764	1.584380401	0.958110895	21379	Tbrg4
E9PV22;Q505F5	Lrrc47	2	1	2	1	0.0028604	1.6725	1249400	1654200	1133000	153160	Lrrc47	1554	0.755289566	0.906835281	0.684923226	72946	Lrrc47
E9PV24-2;E9PV24	Fga	2	11	23.3	27	0	49.628	125500000	61036000	98488000	10675000	Fga	537	2.056163576	0.78476494	1.613605086	14161	Fga
E9PV44;O35143	Atpif1	2	3	24.3	5	0	4.1767	47441000	89324000	47736000	53578000	Atpif1	256	0.531111459	1.00621825	0.534414043	11983	Atp5if1;Atpifl
E9PVM7;P48774;E9Q024	Gstm5	5	4	23.7	7	0	7.8159	18180000	14910000	14891000	3114500	Gstm5	915	1.219315895	0.819086909	0.998725687	14866	Gstm5
E9PW43; Q9CQS8	Gm10320; Sec61b	2	2	18.6	5	0	4.2471	18291000	6659100	10873000	5255100	Gm10320	740	2.746767581	0.594445356	1.632803232	66212	Gm10320; Sec61b
E9PWE8;Q3TT92;Q62188	Dpysl3	3	11	28.8	13	0	24.167	77543000	8512700	18993000	2589300	Dpysl3	987	9.109095822	0.244935068	2.231137007	22240	Dpysl3
E9PWG4; P05977-2;P05977; E0CZ30	Myl1	4	3	37.3	5	0	7.9061	9569500	3034600	8971100	1889400	Myl1	1084	3.153463389	0.937467997	2.956271008	17901	Myl1
E9PWK1;Q9D379;F6YTS6	Ephx1	3	5	10	8	0	9.1202	42205000	9954600	17676000	2568200	Ephx1	990	4.239748458	0.418812937	1.775661503	13849	Ephx1
E9PXC0;P14576	Srp54c;Srp54	2	1	1.8	1	0.0033937	1.6203	1467600	989680	1288900	131750	Srp54c	1557	1.482903565	0.878236577	1.30234015	100101806	Srp54;Srp54c
E9PY26;Q8R016	Blmh	2	1	6.1	1	0.0064725	1.3665	551240000	513760000	514060000	157250000	Blmh	134	1.072952351	0.932552064	1.00058393	104184	Blmh
E9PYF1;Q8VCT3	Rnpep	2	2	3.3	3	0	4.9064	4238800	2703900	3898000	392910	Rnpep	1484	1.567661526	0.919599887	1.441621362	215615	Rnpep
E9PYH2;Q91V12-2;Q91V12-3;Q91V12-4;Q91V12	Acot7	5	1	2.9	3	0	3.0057	8256600	5751800	6822600	1057000	Acot7	1280	1.435481067	0.826320762	1.186167808	70025	Acot7
E9PYI8;Q9JMA1	Usp14	2	6	14.4	10	0	7.3231	14491000	14221000	19760000	1978100	Usp14	1071	1.018986007	1.363604996	1.38949441	59025	Usp14
E9PZ00;Q8BFQ1;K3W4L3;J3QPG5;Q61207	Psap	5	8	14.2	11	0	14.216	85925000	33191000	32809000	5052200	Psap	746	2.588804194	0.381832994	0.988490856	19156	Psap
E9PZ16;B1B0C7;Q05793	Hspg2	4	55	15.7	108	0	137.15	564410000	384870000	371800000	8017900	Hspg2	623	1.46649518	0.658740986	0.966040481	15530	Hspg2
E9PZ72;Q9DAK3;E9PZ17;E9QAZ7;E9Q922;E9PYH9;E9Q1X7;Q91V93	Rhobtb1;Rhobtb2	8	2	3.2	4	0.002849	1.6481	25496000	12144000	29387000	1987400	Rhobtb1	1069	2.099472991	1.152612174	2.419878129	69288	Rhobtb1;Rhobtb2
E9PZF0;Q01768;Q5NC80;P15532	Gm20390; Nme2;Nme1	5	12	59.6	52	0	26.726	985230000	1340400000	1262800000	209150000	Gm20390	106	0.735026858	1.281731169	0.942106834	18103	Nme1;Gm20390;Nme2
E9PZS8;Q61387	Cox7a2l	2	3	21.6	4	0	4.9545	12640000	16197000	14933000	5414800	Cox7a2l	728	0.780391431	1.181408228	0.921960857	20463	Cox7a2l
6A7; Q9Z2Y3-3;Q9Z2Y3;Q9Z2Y3-2	Homer1	6	1	4.4	1	0.0095339	1.3061	1817900	2220700	2262100	576210	Homer1	1418	0.818615752	1.244347874	1.01864277	26556	Homer1
E9Q0U7;Q61699-2;Q61699	Hsph1	4	2	4.3	2	0	2.3042	3947000	3761400	3062700	251910	Hsph1	1529	1.04934333	0.775956423	0.814244696	15505	Hsph1
E9Q133;P80318;Q3U0I3	Cct3	5	11	28.4	21	0	30.01	75444000	48994000	64162000	6823600	Cct3	662	1.539862024	0.850458618	1.309588929	12462	Cct3
E9Q1V3;O35435;F6TCT7	Dhodh	3	2	8.7	5	0	5.238	6468600	9727300	7184200	2153300	Dhodh	1038	0.664994397	1.11062672	0.738560546	56749	Dhodh
E9Q1W0; E9Q1T1;A0A0G2JGS4;Q6PHZ2-2;Q6PHZ2-4;Q6PHZ2;Q6PHZ2-5;E9Q1V9;Q8CCM0	Camk2d	24	4	13.3	14	0	27.492	1066600000	571400000	1072100000	86395000	Camk2d	200	1.866643332	1.005156572	1.876268813	108058	Camk2d
E9Q242;P54822;A0A140LHU1;E9Q3T7	Adsl	4	4	11.1	8	0	9.9166	16945000	16733000	16375000	2353700	Adsl	1017	1.012669575	0.966361759	0.978605151	11564	Adsl
E9Q2H8;E9PYA3;A0A0R4J052;G5E8T9;Q99KB8-2;Q99KB8;D3YWI0	Hagh	7	3	14.3	6	0	8.2333	15116000	10487000	15607000	4029700	Hagh	827	1.441403643	1.032482138	1.488223515	14651	Hagh
E9Q2Q9;D3Z3Z9;Q9WUZ7;A0A3B2WCC8;F6X737;A0A3B2WB97	Sh3bgr	8	3	34.8	7	0	7.9024	9484500	16359000	15071000	5728600	Sh3bgr	712	0.579772602	1.589013654	0.921266581	50795	Sh3bgr
E9Q2T3;O35452;A0A571BG59;A0A5F8MPH8	Tnxb	10	3	0.9	4	0.0006158	2.1214	2372100	2738400	3448500	63117	Tnxb	1569	0.866235758	1.453775136	1.259312007	81877	Tnxb
E9Q397;Q3UGX2;P15508	Sptb	3	5	4.3	7	0	8.096	4578100	5289100	5885500	124570	Sptb	1560	0.865572593	1.285576986	1.112760205	20741	Sptb
E9Q3B9;D3YYS6;O35678;Q3UFN1	Mgll	4	2	6.9	3	0	3.3367	6178500	7197200	9234100	1269000	Mgll	1223	0.858458845	1.494553694	1.283012838	23945	Mgll
3;Q8CC35-2;Q8CC35	Synpo	4	4	3.9	5	0	4.6398	23074000	14908000	29860000	1116600	Synpo	1267	1.547759592	1.294097252	2.002951435	104027	Synpo
E9Q3V6;P42208;F6WYM0;D3YYB1;D3Z3C0	Sept2	10	7	26.5	12	0	16.941	88029000	14395000	34662000	5879500	Sept2	704	6.11524835	0.393756603	2.407919416	18000	Septin2
E9Q3X0;Q9EQK5	Mvp	3	20	24.9	31	0	33.158	108880000	38921000	89859000	5044700	Mvp	748	2.797461525	0.825303086	2.308753629	78388	Mvp
E9Q401;F6U7V1	Ryr2	10	71	16.9	140	0	162.98	352080000	561010000	433100000	5940400	Ryr2	699	0.627582396	1.230118155	0.772000499	20191	Ryr2
E9Q4B9;E9Q827;P56212-2;P56212; P60840-2;P60840	Arpp19;Ensa	6	1	15.5	3	0	3.1618	5508700	3891100	4040500	2906900	Arpp19	942	1.41571792	0.733476138	1.038395312	59046	Arpp19;Ensa
E9Q4M4;Q91VN4	Chchd6	2	2	12.2	4	0	4.7678	3031100	3239200	2618200	750550	Chchd6	1363	0.935755742	0.863778826	0.808285997	66098	Chchd6
E9Q4Z2	Acacb	1	3	1.5	5	0	4.7083	6476100	9208200	6662300	233420	Acacb	1533	0.703297061	1.028751872	0.723518169	100705	Acacb
E9Q557;E9PZW0	Dsp	2	81	28.3	159	0	221.56	618470000	699720000	756250000	13581000	Dsp	478	0.883882124	1.222775559	1.080789459	109620	Dsp
E9Q616	Ahnak	4	68	32	108	0	160.6	631030000	187070000	297670000	3074700	Ahnak	921	3.373229272	0.471720837	1.591222537	66395	Ahnak
E9Q6P2;G3X8U7;E0CZ78;P63328-2;P63328; P48453-2;P48453; A0A2I3BPC5;P48455	Ppp3cb;Ppp3ca;Ppp3cc	9	3	6.2	5	0	6.1472	14433000	10479000	11107000	1800500	Ppp3cb	1097	1.377326081	0.769555879	1.059929383	19056	Ppp3cb;Ppp3cc;Ppp3ca

E9Q7H6;Q922A3;Q3UQ84;A0A0G2JGZ2;A0A0G2JFR0;Q3TP97	Tars2	6	2	2.8	3	0	2.335	2387400	2851900	2660900	204570	Tars2	1541	0.837126126	1.114559772	0.933027105	71807	Tars2
E9Q7N5;Q8R2Q4-3;Q8R2Q4-2;Q8R2Q4;Q8C1C2	Gfm2	6	3	4.6	5	0	6.0905	2848100	4502800	4812700	350270	Gfm2	1497	0.632517545	1.689793195	1.068823843	320806	Gfm2
E9Q7N6;Q3V1B8	Gal3st4	2	2	3.4	4	0.0028555	1.6674	12314000	29634000	16273000	3037200	Gal3st4	928	0.415536208	1.321503979	0.549132753	330217	Gal3st4
E9Q800;Q8CAQ8-2;Q8CAQ8;Q8CAQ8-5;Q8CAQ8-3;E9QAY6;A0A0U1RP81	Immt	11	41	58.3	129	0	133.95	1192900000	1924700000	1419400000	119590000	Immt	168	0.619784902	1.189873418	0.737465579	76614	Immt
E9Q8Z4;E9Q8Z9;D3Z2H2;E9Q903;E9Q901;E9Q904;G3X9V2;E9Q8Z5;E9Q8Z6;E9Q8Z8;P30999-2;P30999-3;P30999;D3Z2H7;E9Q986;D3Z7H6;E9Q906;E9Q907;E9Q905	Ctnnd1	19	4	4.7	3	0	3.2727	7595800	5226200	6001200	414060	Ctnnd1	1475	1.45340783	0.790068196	1.148291302	12388	Ctnnd1
E9Q933;Q8BK08;Z4YN74	Tmem11	3	2	9.7	4	0.0006234	2.2179	8994700	9800200	13300000	4848500	Tmem11	766	0.9178078	1.478648537	1.357115161	216821	Tmem11
E9Q9C4;E9Q9C1;E9QK41;Q8K4G5;E9Q9C0;E9Q9C2;Q8K4G5-4;Q8K4G5-2;E9Q9C7;E9Q030;Q8K4G5-3;E9Q9D2;E9Q9D1;E9Q9Q7	Ablim1	16	20	31.5	33	0	32.278	119060000	144910000	177450000	10473000	Ablim1	540	0.821613415	1.490424996	1.224553171	226251	Ablim1
E9Q9F5;E9Q1G8;O55131	Sept7	9	8	19.2	16	0	37.199	119700000	25268000	44115000	9262600	Sept7	580	4.737217033	0.368546366	1.745884122	235072	Septin7
E9Q9T8;Q3UIK0;O70468;Q3TF37	Mybpc3	6	74	60.5	402	0	323.31	4909700000	6215900000	6352500000	263340000	Mybpc3	86	0.789861484	1.293867242	1.021975901	17868	Mybpc3
A1C7ZN10;K3W4L0;B2RRE2;E9QAX2;Q9JMH9-5;Q9JMH9-7;Q9JMH9-2;Q9JMH9-4;Q9JMH9-1;Q9JMH9;Q9JMH9-6	Myo18a	13	5	3.5	5	0	8.5464	4542700	3872500	4705800	174560	Myo18a	1549	1.173066495	1.035903758	1.21518399	360013	Myo18a
E9QAT9;Q9ERP3	Trim54	2	2	8.2	3	0	4.7819	6763000	5502400	7524400	1236900	Trim54	1234.5	1.229100029	1.112583173	1.36747601	58522	Trim54
E9QJT5;P56376	Acyp1	2	4	30.6	6	0	7.0801	13238000	17521000	13571000	5327800	Acyp1	733	0.755550482	1.025154857	0.774556247	66204	Acyp1
E9QK48;Q7TNG5;Q7TNG5-2	Eml2	4	3	3.8	6	0	5.209	6777600	6527600	5945700	519060	Eml2	1439	1.038298915	0.877257436	0.910855445	72205	Eml2
E9QKK8;F6Q8D3;Q9QZW0-2;Q9QZW0	Atp11c	4	1	1.2	3	0.0005981	1.9278	7858800	5795300	11361000	522060	Atp11c	1437	1.356064397	1.445640556	1.960381689	320940	Atp11c
E9QL12;E9PXU9;A0A0N4SWH3;A0A0N4SWG7;A0A0N4SUN3;A0A0N4SVX9;Q9ESD7;A0A0N4SUJ2;E9Q423;A0A0N4SV63;Q9ESD7-3;Q9ESD7-2	Dysf	12	13	7.5	18	0	24.055	45238000	37950000	48258000	1318000	Dysf	1209	1.192042161	1.066758035	1.271620553	26903	Dysf
E9QMK9;Q8BH86-2;Q8BH86	9030617O03Rik	3	6	11.9	8	0	10.779	3461700	21630000	5901700	1315300	9030617O03Rik	1210	0.160041609	1.704855996	0.272847896	217830	Dglucy
E9QN08;A0A0R4J1L2;Q91VK2;Q80T06;P57776-2;P57776;D3Z7N2;D3YY68;D3YUQ9;D3YZT9;F6ZFU0;A0A0R4J1E2;P57776-3	Eef1d	13	3	13.9	6	0	20.265	27252000	16482000	21942000	4713900	Eef1d	772	1.653440116	0.805151915	1.331270477	66656	Eef1d
E9QN70;P02469	Lamb1	2	19	10.7	36	0	48.133	90669000	125080000	78983000	4568000	Lamb1	781	0.724888072	0.871113611	0.631459866	16777	Lamb1
E9QPX1;P39061-2;P39061-1;P39061	Col18a1	4	3	2.1	3	0	5.6931	4490700	3233400	2554400	150480	Col18a1	1555	1.388847653	0.568820006	0.79000433	12822	Col18a1
E9QPX3;Q9CXZ1;A0A338P7A0	Ndufs4	3	4	25.1	17	0	24.748	318130000	494430000	380680000	127220000	Ndufs4	160	0.643427786	1.196617735	0.769937099	17993	Ndufs4
E9QQ25;Q62407;A0A087WSE3;Q62407-4;Q62407-3	Speg	8	8	2.6	9	0	8.8516	8115500	15004000	12571000	279410	Speg	1524	0.540889096	1.549011152	0.837843242	11790	Speg
F2Z455;Q9R059	Fhl3	2	1	3.6	1	0.0005938	1.8863	2235400	1149300	3517400	461860	Fhl3	1459	1.945010006	1.57349915	3.060471591	14201	Fhl3
F6QTS1;O88544;D3Z1R9;D3YV99	Cops4	4	5	23.3	6	0	11.74	18562000	13549000	16776000	3407500	Cops4	879	1.369990405	0.90378192	1.238172559	26891	Cops4
F6QYE1;O09161;E9PZ67	Casq2	3	10	23.4	32	0	29.753	613110000	1046500000	865170000	193570000	Casq2	113	0.585867176	1.411117092	0.826727186	12373	Casq2
F6R5R5;P70699	Gaa	2	1	4.8	1	0	2.5141	2029200	317750	707290	476270	Gaa	1455	6.386152636	0.348556081	2.225932337	14387	Gaa
F6RER8;E9QNU1;Q8R2G4-8;Q8R2G4;A0A0R4J1M9;A0A0R4J1Q4;E9Q7Q8;A0A0R4J1N4;F6Z2C7;F6W3A0;E9Q984;E9Q9Q8;E9Q8Z0;E9Q7Z7;E9Q1U7;Q8R2G4-7	Art3	16	4	13.2	8	0	6.1786	15166000	32952000	17891000	5003300	Art3	756	0.460245205	1.179678228	0.542941248	109979	Art3
F6RPJ9;Q9JHR7	Ide	2	7	7.6	9	0	6.4472	15060000	5054400	7217200	540200	Ide	1428	2.979582146	0.479229748	1.4279044		Ide
F6SQH7;Q91VA6	Poldip2	2	2	8.5	3	0	3.8362	3146400	3496200	2175400	662860	Poldip2	1395	0.899948516	0.691393338	0.622218409	67811	Poldip2
F6T2S0;Q91W09;A0A494BAI5;Q544X6;P22315;A0A494B9Y8;A0A494BBL4	Fech	8	4	12.9	4	0	5.1602	4947800	22933000	4251700	2293400	Fech	1022	0.215750229	0.859311209	0.18539659	14151	Fech
F6TCV0;D3YX28;D3YX27;Q9JIY5	Htra2	4	1	4.4	2	0	5.8778	5012700	5147400	4356900	1161300	Htra2	1258	0.973831449	0.869172302	0.846427323	64704	Htra2
F6TFN2;E9PYI7;F6VG99;E9PYF4	Lmo7	5	10	9.8	15	0	12.703	11873000	11912000	12102000	571220	Lmo7	1420	0.996725991	1.019287459	1.015950302	380928	Lmo7
F6UFZ5;G3X9Z4	Pcf11	2	1	0.7	2	0.0048966	1.4448	19702000	12982000	19698000	685900	Pcf11	1386	1.517639809	0.999796975	1.51733169	74737	Pcf11

F6UV57;F6YCM8;F8WIH0;D3Z7V3;H7BX64;Q3URD3-4;Q3URD3-3;Q3URD3-5;Q3URD3-2;Q3URD3	Slmap	10	11	23.8	18	0	54.758	99659000	49229000	145610000	8879200	Slmap	592	2.024396189	1.461082291	2.957809421	83997	Slmap
F6V7S2;Q8BFZ1	Tecrl	2	1	4.7	3	0.0005974	1.9237	1916000	3591100	1581600	794200	Tecrl	1351	0.533541255	0.825469729	0.440422155	243078	Tecrl
F6VQ81;Q3TUJ9;Q8BKP1;Q3TAI4;A2AUD5;Q9CYZ2;V9GWU5	Tpd52l2	7	5	36.6	8	0	17.305	20875000	10636000	13679000	5527100	Tpd52l2	724	1.962673938	0.655281437	1.286103798	66314	Tpd52l2
F6VV02;Q3UMA3;Q99LI8	Hgs	3	1	3.7	2	0.0005935	1.8825	61843000	71643000	82779000	38159000	Hgs	283	0.863210642	1.338534677	1.155437377	15239	Hgs
F6YVP7;A0A1Y7VKY1;P62270;S4R1N6	Gm10260; Rps18	5	7	38.2	16	0	19.009	112940000	78788000	86100000	26963000	Gm10260	336	1.433467025	0.762351691	1.092806011	20084	ps5;Rps18-ps6
F6Z5N5;F6YTG0;Q3TLP5-3;Q3TLP5-2;Q3TLP5	Echdc2	5	1	14.3	2	0	3.863	1212500	2689000	1315700	801470	Echdc2	1348	0.450911119	1.085113402	0.489289699	52430	Echdc2
F6ZFT1;F8WJ64;Q9CR21	Ndufab1	3	4	26	23	0	14.362	294380000	454830000	419240000	268720000	Ndufab1	85	0.647230833	1.424145662	0.921750984	70316	Ndufab1
F7ARZ1; Q8VE22;Q3TI14;A7M7Q8	Mrps23	5	4	44.2	6	0	8.873	8381400	9423500	10498000	4072700	Mrps23	824	0.889414761	1.252535376	1.114023452	64656	Mrps23
F7CK55;Q63ZW6;P02463;A0A1B0GRC0;A0A1B0GSI7	Col4a5;Col4a1	5	2	3	7	0	4.3879	16383000	8345000	8453300	943050	Col4a5	1307	1.963211504	0.515979979	1.012977831	12830	Col4a1;Col4a5
F7CVJ5;F7DBB3;E9PYB0	Ahnak2	3	4	20.3	5	0	10.214	32664000	2377100	5366600	625270	Ahnak2	1402	13.74111312	0.164297085	2.257624837		
F7D432;E9PWE0;E0CYV0;P23506;P23506-2;F6V9F1	Pcmt1	7	3	15.5	5	0	10.773	5699300	6894600	9059600	1291000	Pcmt1	1216	0.826632437	1.589598723	1.314013866	18537	Pcmt1
F8VPN4;A0A0G2JGI9	Agl	6	17	12.9	27	0	43.765	62010000	98912000	64652000	2914500	Agl	941	0.626920899	1.042606031	0.653631511	77559	Agl
F8VQJ3;P02468	Lamc1	6	30	22.6	60	0	99.143	216260000	200790000	146300000	8430100	Lamc1	612	1.07704567	0.676500509	0.728621943	226519	Lamc1
F8WHL2;Q8CIE6	Copa	3	10	8.3	13	0	15.476	42709000	16473000	26806000	1238800	Copa	1233	2.592666788	0.627642886	1.627268864	12847	Copa
F8WHM5;Q61543	Glg1	3	3	3	5	0	4.8828	9239900	1829400	5701000	259640	Glg1	1527	5.050781677	0.616998019	3.116322291	20340	Glg1
F8WHU8;Q9D8T7	Slirp	4	3	30.4	5	0	3.731	32584000	36751000	21443000	22243000	Slirp	365	0.88661533	0.658083722	0.583467117	380773	Slirp
F8WID5; B7ZNL3;E9Q448	Tpm1	3	1	60.1	2	0	3.4706	11654000	3491500	4271200	1048200	F8WID5	1281	3.337820421	0.366500772	1.223313762	22003	Tpm1
F8WIR1;P18242;A0A1B0GT66;F6Y6L6	Ctsd	4	7	23.8	24	0	28.421	150500000	105340000	98603000	22015000	Ctsd	366	1.428707044	0.655169435	0.936045187	13033	Ctsd;Gm49369
F8WIS9;P11798	Camk2a	4	1	6.3	1	0.0006262	2.2518	1225300	2432100	1255600	245080	Camk2a	1531	0.503803298	1.024728638	0.516261667	12322	Camk2a
F8WIT2;P14824	Anxa6	2	31	49.3	83	0	93.72	612680000	538100000	620020000	42936000	Anxa6	273	1.138598773	1.011980153	1.152239361	11749	Anxa6
F8WIV2;Q60854;E9Q3Y1;E9Q6X2;E9Q108;E9Q0P9;K7E6F1	Serpinb6a;Serpinb6	11	10	30.6	24	0	24.639	179070000	93661000	143500000	19233000	Serpinb6a	396	1.911895026	0.801362596	1.53212116	20719	Serpinb6a;Serpinb6
F8WJ05;Q61702	Itih1	3	5	6.7	8	0	9.7564	26984000	8661100	14480000	1204200	Itih1	1247	3.115539597	0.53661429	1.671843068	16424	Itih1
F8WJI3;Q8R3Q6;E9Q4B5	Ccdc58	3	2	19.4	3	0.0006075	2.0236	7534800	11744000	8023400	4115800	Ccdc58	820	0.641587193	1.064845782	0.683191417	381045	Ccdc58;Mix23
F8WJK8;Q99L47;E9Q1V0	St13	4	6	18	8	0	10.908	54356000	28708000	39973000	12401000	St13	501	1.893409503	0.735392597	1.392399331	70356	St13
G3UVV4; P17710-3;P17710-4;P17710; P17710-2	Hk1	9	20	30.1	46	0	60.154	281780000	191440000	277330000	16302000	Hk1	426	1.4718972	0.984207538	1.448652319	15275	Hk1
G3UW30;F6ZHD8;Q9D6Y9	Gbe1	3	1	2	3	0	2.8547	5655000	4911800	4988100	514230	Gbe1	1441	1.151309092	0.882068966	1.01553402	74185	Gbe1
G3UWC2;Q9CZR2	Naalad2	2	5	7.8	7	0	10.499	22584000	1254900	4595900	729200	Naalad2	1369	17.99665312	0.20350248	3.662363535	72560	Naalad2
G3UXY0;G3UXZ5;P97371;G3X9K9;G3UWN9	Psme1	6	6	27.4	10	0	7.1659	30969000	24308000	27403000	5853100	Psme1	707	1.274025012	0.884852595	1.127324338	19186	Psme1
G3UY72;G3UYR8;Q9DBF1-2;Q9DBF1;E9Q1H3	Aldh7a1	6	4	16.5	7	0	6.9185	5902000	6514600	4474100	1290000	Aldh7a1	1217	0.905965063	0.758065063	0.686780462	110695	Aldh7a1
G3UYU4;O08917	Flot1	2	3	8.4	4	0	6.2502	7374900	4553400	3015400	785640	Flot1	1353	1.619646857	0.408873341	0.662230421	14251	Flot1
G3UYV7; P62858	Rps28	2	3	57.1	4	0	25.482	71458000	30189000	43449000	50166000	Rps28	261	2.3670211	0.608035489	1.439232833	54127	Rps28
G3UZ48;G3UZI2;A0A0R4J259;G3V018;Q7TMK9-2;Q7TMK9;G3UXJ6	Syncrip	9	4	11.2	7	0	9.2031	23018000	14783000	15053000	2370600	Syncrip	1016	1.557058784	0.653966461	1.018264222	56403	Syncrip
G3UZP7;P01899;E9PWT4;E9QJR9; P79568;Q8HWB2;P14430;P14429;P01897	H2-D1;H2-Q7;H2-Q6;H2-Q4;H2-Q8;H2-L	19	2	13.9	6	0	7.9968	30504000	8443500	9252000	2061800	H2-D1	1051	3.612719844	0.303304485	1.09575413	14964	H2-Q7;H2-D1;H2-L; H2-Q4;H2-Q6;H2-Q8
G3UZZ3; Q3T9X3;Q3TCR7;G3X9G4;F8WIV5;P39054-2;P39054	Dnm2	20	7	8.6	10	0	7.8187	21521000	11528000	19321000	1139600	Dnm2	1262	1.86684594	0.897774267	1.676006246	13430	Dnm2
G3X8R0;A0A494BBE3;Q60870	Reep5	3	6	25.9	9	0	17.831	187140000	168970000	171270000	97604000	Reep5	188	1.107533882	0.915197179	1.013611884	13476	Reep5
G3X8U3	2210016F16Rik	1	2	5.9	4	0	2.3652	6609500	7669400	8450600	1178200	2210016F16Rik	1254	0.861801445	1.278553597	1.101859337	70153	2210016F16Rik
G3X975;Q8BYM8;A0A1B0GSP2	Cars2	3	2	5.1	4	0	4.202	1033800	2362400	2266600	298580	Cars2	1514	0.437605825	2.192493713	0.959448019	71941	Cars2
G3X9Q1;Q3TZS3;Q61738-4;Q61738-2;Q61738-5;Q61738-3;Q61738-6;Q61738	Itga7	8	10	11.1	14	0	28.313	26305000	25256000	26047000	1667700	Itga7	1128	1.041534685	0.990191979	1.03131929	16404	Itga7
G3X9T8;G3X8Q5;E9PZD8;Q61147	Cp	5	4	4	3	0	4.3327	8186000	6601800	7841000	497820	Cp	1448	1.239964858	0.957854874	1.187706383	12870	Cp
G3X9U9;Q9CQ92	Fis1	3	4	26.2	8	0	12.736	14004000	16370000	12679000	8577400	Fis1	604	0.855467318	0.905384176	0.774526573	66437	Fis1
G3X9V0;P97372;E0CZ90	Psme2	3	3	14.3	4	0	5.5752	10407000	5571000	8553900	1676900	Psme2	1126	1.868066774	0.821937158	1.535433495	19188	Psme2
G3XA66; D3YVW0;F7C846;P47802;G3UYJ5;G3XA75;D3Z3F4;G3UXX9	Mtx1	8	4	16.1	4	0	3.9585	6645900	8575200	6383800	1480700	Mtx1	1172	0.775013994	0.960562151	0.744449109		Mtx1
G5E814;Q9D8B4;A0A3B2W441	Ndufa11	4	3	35	12	0	22.981	47499000	104340000	55324000	49636000	Ndufa11	262	0.455232892	1.16474031	0.5302281	69875	Ndufa11
G5E850;P56395;A0A494B9D8	Cyb5a	4	6	74.5	16	0	17.303	556550000	416600000	390310000	232740000	Cyb5a	94	1.335933749	0.701302668	0.936893903	109672	Cyb5a
G5E883;P57784	Snrpa1	2	1	5.8	1	0.0099895	1.2816	2620400	841910	1128400	926840	Snrpa1	1316	3.112446699	0.430621279	1.340285779	68981	Snrpa1
G5E895;S4R2G9;D3Z494;P21300	Akr1b10; Akr1b7	4	4	15.2	6	0	10.782	18708000	44632000	23548000	5711900	Akr1b10	713	0.41916114	1.25871285	0.527603513	67861	Akr1b10; Akr1b7
G5E8J6	Hrc	1	8	14.8	18	0	15.775	99184000	163560000	83345000	12925000	Hrc	491	0.606407435	0.840306904	0.509568354	15464	Hrc
G5E8R1;G5E8R0;E9Q455;G5E8R2; E9Q4[illegible]	[illegible]	[illegible]	[illegible]	[illegible]	[illegible]	[illegible]	[illegible]	[illegible]	[illegible]	[illegible]	[illegible]	[illegible]	[illegible]	[illegible]	[illegible]	[illegible]	[illegible]	[illegible]

G5E8R3;E9QPD7;Q05920	Pcx;Pc	6	7	7.5	9	0	14.85	13858000	17240000	10925000	789440	Pcx	1352	0.803828306	0.788353298	0.633700696	18563	Pcx;Pc
G5E902;Q8VEM8	Slc25a3	2	16	38.8	101	0	123.18	2801800000	4440700000	3142000000	637750000	Slc25a3	39	0.630936564	1.121421943	0.707546108	18674	Slc25a3
G5E924;Q8R081;G3UY38	Hnrnpl	3	4	7	7	0	8.1682	26912000	10090000	20803000	2512200	Hnrnpl	1002	2.667195243	0.773000892	2.061744301	15388	Hnrnpl
H3BJ37;Q8BIG7	Comtd1	2	2	10.5	2	0	2.4816	5515800	2856200	8874900	1854900	Comtd1	1088	1.931167285	1.608995975	3.107240389	69156	Comtd1
BJL6;H3BKH6;Q9R0P3;H3BK43;H3BJC6	Esd	7	8	32.4	17	0	15.686	83182000	79024000	87215000	21778000	Esd	367	1.052616927	1.048484047	1.103652055	13885	Esd
H3BJZ9;Q921H8;Q8VCH0;H3BKL5	Acaa1a;Acaa1b	5	3	15.1	5	0	6.6173	13863000	14829000	10718000	2717100	Acaa1a	961	0.934857374	0.773137128	0.722772945	113868	Acaa1a;Acaa1b
H7BWX9;D3Z794;P61957;G3UZX6;G3UZ60; G3UWI9; G3UWX9;G3UZA7;Q9Z172-2;Q9Z172	Sumo2;Sumo3	10	2	41.5	3	0	10.639	17347000	19181000	15032000	28615000	Sumo2	324	0.904384547	0.86654753	0.783692195	170930	Sumo3;Sumo2;Gm49325
H7BWZ1;D3YW97;P61961	Ufm1	3	1	26.3	2	0.0006031	1.9785	6237700	2349800	3996100	3195600	Ufm1	909	2.654566346	0.640636773	1.700612818	67890	Ufm1
H7BX88;P47934	Crat	5	20	35.5	53	0	60.445	371850000	771460000	484770000	74443000	Crat	216	0.48200814	1.303670835	0.628379955	12908	Crat
H7BX95;Q6PDM2	Srsf1	2	1	4	3	0.0079872	1.3326	8439600	3773500	3052500	1128600	Srsf1	1265	2.236544322	0.361687758	0.808930701	110809	Srsf1
I7HLV2;Q6ZWV3;P86048	Rpl10;Rpl10l	4	4	20.4	9	0	9.7073	32451000	28342000	22521000	12070000	Rpl10	504	1.144979183	0.694000185	0.794615765	110954	Rpl10;Rpl10l
J3QMG3;Q60931	Vdac3	2	11	38	28	0	51.056	320760000	580560000	407670000	109070000	Vdac3	174	0.552501033	1.270950243	0.702201323	22335	Vdac3
3W4M4;Q9CZ42-2;Q9CZ42-3;Q9CZ42	Carkd	6	3	13.6	6	0	4.7995	7034700	12200000	6140700	1991500	Carkd	1068	0.576614754	0.872915689	0.503336066	69225	Naxd
J3QN31;P28650;P28650-2	Adssl1	3	5	12.1	13	0	20.571	48571000	54913000	54875000	7804900	Adssl1	630	0.884508222	1.12978938	0.999307996	11565	Adss1;Adssl1
J3QNN5;Q8R127	Sccpdh	2	2	13.8	4	0	2.9202	14934000	15621000	17571000	6644400	Sccpdh	670	0.956020741	1.176576939	1.124831957	109232	Sccpdh
J3QP56;P97823-2;P97823; D3YUG4;D3Z111	Lypla1	5	2	10.7	3	0.0005949	1.9022	12221000	10822000	12762000	3260600	Lypla1	894	1.129273702	1.044268063	1.179264461	18777	Lypla1
J3QQ16;A0A087WS16	Col6a3	2	2	22.9	112	0	243.48	1234100000	405650000	425600000	14059000	Col6a3	467	3.042277826	0.344866704	1.049180328	12835	Col6a3
K3W4R2;Q6URW6-2;Q6URW6;Q6URW6-3;A0A140LI60	Myh14	5	6	5.1	8	0	9.2166	232970000	263930000	353860000	10942000	Myh14	531	0.882696169	1.518908014	1.340734286	71960	Myh14
K3W4R6;K3W4R7;Q6P3Z7;J3QQ13;P50752-2;P50752-3;P50752-4;P50752; A0A087WRX3	Tnnt2	19	18	44.7	141	0	249.44	2309900000	4021100000	3052700000	1.072E+09	Tnnt2	23	0.574444804	1.321572362	0.759170376	21956	Tnnt2
O08528;E9Q5B5	Hk2	6	23	36.1	64	0	83.362	338400000	360270000	385760000	25757000	Hk2	344	0.939295528	1.139952719	1.070752491	15277	Hk2
O08529	Capn2	1	8	12	11	0	23.802	38135000	19180000	27269000	2584000	Capn2	988	1.98826903	0.715064901	1.421741397	12334	Capn2
O08547;E9Q6R3;A0A0G2JF08	Sec22b	4	4	19.5	7	0	7.1931	18920000	9548900	10684000	3292300	Sec22b	890	1.981380054	0.564693446	1.118872331	20333	Sec22b
O08553	Dpysl2	2	13	37.8	31	0	35.523	224060000	73863000	108220000	13523000	Dpysl2	479	3.033453827	0.482995626	1.46514493	12934	Dpysl2
O08600	Endog	1	5	22.8	8	0	9.9868	15635000	23750000	22477000	4118400	Endog	818	0.658315789	1.437607931	0.9464	13804	Endog
O08677-2;D3YTY9;A0A0R4J038;O08677-3;O08677	Kng1	10	10	28.5	19	0	17.687	94180000	67950000	92481000	10969000	Kng1	529	1.386019132	0.981960076	1.361015453	16644	Kng1
O08692	Ngp	1	2	10.8	2	0.0006146	2.1005	3493900	2719400	2738600	885840	Ngp	1328	1.284805472	0.783823235	1.007060381	18054	Ngp
O08749	Dld	1	16	36.3	80	0	123.25	765410000	1620400000	1009100000	189880000	Dld	116	0.472358677	1.318378385	0.62274747	13382	Dld
O08804;F7B9A0	Serpinb6b	3	4	14.9	8	0	8.7471	19459000	15738000	23667000	3207600	Serpinb6b	905	1.236434109	1.21624955	1.503812429	20708	Serpinb6b
O08972;Q99LD8;G3UZR0	Ddah2	3	2	9	3	0	7.0945	20576000	4454500	6988200	2135000	Ddah2	1040	4.619149175	0.339628694	1.5687956	51793	Ddah2
O08997	Atox1	1	2	20.6	3	0	3.9656	4818600	3450500	4230900	3872600	Atox1	839	1.396493262	0.878035114	1.22617012	11927	Atox1
O09111	Ndufb11	1	4	45	11	0	17.767	121820000	245850000	151290000	78024000	Ndufb11	212	0.495505389	1.2419143	0.615375229	104130	Ndufb11
O09131;A0A494BAB1	Gsto1	6	11	38.3	25	0	21.759	75298000	93172000	110890000	19456000	Gsto1	392	0.80816125	1.472681877	1.190164427	14873	Gsto1
O35074;Q8BXC0	Ptgis	2	8	18.2	8	0	16.393	62610000	6261300	9613200	2760600	Ptgis	956	9.999520866	0.153540968	1.535336112	19223	Ptgis
O35114	Scarb2	1	2	4.4	2	0.000627	2.2568	10827000	7959300	7387300	1260100	Scarb2	1226	1.360295503	0.682303501	0.928134384	12492	Scarb2
O35129;F6QPR1	Phb2	3	12	43.8	26	0	33.36	388880000	574420000	444370000	70328000	Phb2	226	0.676995926	1.142691833	0.773597716	12034	Phb2
O35226;O35226-2;O35226-5;O35226-4;O35226-3	Psmd4	6	3	9.6	3	0.0006139	2.0853	7763200	6033900	8941200	1823400	Psmd4	1095	1.286597391	1.15174155	1.481827674	19185	Psmd4
O35386	Phyh	1	1	3	3	0.010011	1.2849	2303400	3140600	2450300	501020	Phyh	1447	0.733426734	1.063775289	0.780201235	16922	Phyh
O35459;F7B227	Ech1	2	13	48.9	38	0	140.53	472780000	1277200000	600380000	148550000	Ech1	140.5	0.37016912	1.269892973	0.470075164	51798	Ech1
O35566	Cd151	2	3	10.3	5	0	4.2486	18740000	21258000	23089000	5519000	Cd151	725	0.881550475	1.232070438	1.08613228	12476	Cd151
O35593	Psmd14	1	2	6.5	2	0	2.8685	13331000	5929100	12156000	1843300	Psmd14	1091	2.24840195	0.911859575	2.050226847	59029	Psmd14
O35639;Q3TET3	Anxa3	4	6	20.4	12	0	16.745	86317000	19630000	43432000	7022800	Anxa3	654	4.397198166	0.503168553	2.212531839	11745	Anxa3
O35683	Ndufa1	1	1	14.3	4	0	9.7788	25264000	46437000	42605000	67301000	Ndufa1	231	0.544048927	1.686391704	0.917479596	54405	Ndufa1
O35685	Nudc	1	3	10.8	4	0	5.4719	5764400	5075600	5514100	833030	Nudc	1341	1.135708094	0.956578308	1.086393727	18221	Nudc
O35855;O88374;A0A1B0GX27	Bcat2	5	11	35.1	19	0	44.812	134480000	189150000	152110000	27565000	Bcat2	330	0.71097013	1.131097561	0.804176579	12036	Bcat2
O35857	Timm44	1	10	21.5	15	0	20.611	36542000	43783000	40128000	5022400	Timm44	752	0.834616175	1.098133654	0.916520111	21856	Timm44
O35864;A0A087WQA8	Cops5	3	3	7.5	4	0	10.378	5368900	6501300	8826900	1226500	Cops5	1243	0.825819451	1.644079793	1.357713073	26754	Cops5
O35943	Fxn	1	3	16.9	3	0	3.6969	7205000	12424000	8214000	2540800	Fxn	993	0.57992595	1.140041638	0.66113973	14297	Fxn
O54724	Ptrf	1	12	41.1	39	0	229.53	291350000	383120000	291420000	58246000	Ptrf	244	0.760466695	1.000240261	0.760649405	19285	Cavin1
O54734	Ddost	1	3	7.7	4	0	14.707	18108000	5274300	6619500	1284300	Ddost	1219.5	3.433251806	0.36555666	1.255048063	13200	Ddost
O54950;D3YUS1;Q8BIQ9;Q91WG5-2;Q91WG5	Prkag1;Prkag2	5	2	6.4	3	0	2.444	6095100	5147100	8274800	888880	Prkag1	1325	1.184181384	1.357615133	1.607662567	19082	Prkag2;Prkag1
O55026;O55026-2	Entpd2	2	2	5.3	2	0	10.154	18273000	1661200	1906200	1008500	Entpd2	1289	10.99987961	0.104317846	1.147483747	12496	Entpd2
O55029	Copb2	1	5	5.5	7	0	7.6184	15802000	6761500	13360000	768400	Copb2	1358	2.337055387	0.8454626	1.975892923	50797	Copb2
O55111	Dsg2	3	6	7.6	7	0	13.547	5577200	10378000	7265300	512960	Dsg2	1442	0.537406051	1.302678764	0.70006745	13511	Dsg2
O55142	Rpl35a	1	5	33.6	9	0	5.428	165080000	98001000	153850000	77729000	Rpl35a	213	1.684472607	0.931972377	1.56988194	57808	Rpl35a
O55143-2	Atp2a2	1	1	46.3	269	0	323.31	5299600000	8247200000	6239100000	500030000	Atp2a2	54	0.64259385	1.17727753	0.756511301		
O55222;D3YZA5	Ilk	4	6	14.8	10	0	10.591	27398000	16765000	20785000	2970000	Ilk	937	1.634237996	0.758632017	1.239785267	16202	Ilk
O55234;Q8BTY5	Psmb5	2	5	24.2	7	0	6.6149	14731000	16580000	14965000	3130600	Psmb5	914	0.888480097	1.015884869	0.902593486	19173	Psmb5
O70250	Pgam2	1	13	57.3	73	0	66.26	1049000000	1446900000	1353300000	305360000	Pgam2	80.5	0.724998272	1.290085796	0.935309973	56012	Pgam2
O70400;S4R1V0	Pdlim1	2	11	33.9	21	0	21.347	79856000	29282000	79911000	11151000	Pdlim1	524	2.727136125	1.00068874	2.729014412	54132	Pdlim1
O70433	Fhl2	1	15	54.1	50	0	60.652	341260000	512920000	513970000	86033000	Fhl2	201	0.665327926	1.506095059	1.002047103	14200	Fhl2
O70435	Psma3	5	6	23.5	8	0	10.259	37585000	26092000	34267000	8147800	Psma3	620	1.440479841	0.911720101	1.313314426	19167	Psma3
O70456	Sfn	1	1	13.3	1	1	-2	68804000	44349000	141420000	14890000	Sfn	454	1.551421678	2.055403756	3.188797944	55948	Sfn
O70548	Tcap	1	6	37.7	13	0	22.578	25027000	24555000	33366000	6822700	Tcap	663	1.019222154	1.333200144	1.358827123	21393	Tcap

O70571	Pdk4	1	5	12.9	8	0	7.8725	4184600	23671000	37713000	2998300	Pdk4	931	0.176781716	9.012330928	1.593215327	27273	Pdk4
O70591	Pfdn2	1	1	7.8	2	0	2.5084	2430400	9644500	3255900	2005000	Pfdn2	1064	0.251998548	1.339656024	0.337591373	18637	Pfdn2
O70622-2;O70622	Rtn2	2	2	12.3	5	0	6.1027	16533000	40899000	22043000	14135000	Rtn2	465	0.404239712	1.333272848	0.538961833	20167	Rtn2
O88322;A0A286YCQ5	Nid2	2	3	3.4	4	0	5.9211	5912200	3661200	3385500	221320	Nid2	1536	1.61482574	0.572629478	0.924696821	18074	Nid2
O88342	Wdr1	2	11	20.6	23	0	25.424	121740000	72243000	138900000	11148000	Wdr1	525	1.685145966	1.140956136	1.92267763	22388	Wdr1
O88441	Mtx2	1	5	24.3	14	0	23.257	28008000	51019000	39063000	10962000	Mtx2	530	0.548971952	1.394708655	0.765655932	53375	Mtx2
O88531;B1B0P8;B1B0P9	Ppt1	3	2	10.8	4	0	3.2594	6247600	3269300	2663700	986300	Ppt1	1296	1.91099012	0.426355721	0.81476157	19063	Ppt1
O88569;O88569-3;O88569-2;A0A0N4SUM2	Hnrnpa2b1	4	12	40.5	39	0	39.892	221610000	137390000	132700000	27199000	Hnrnpa2b1	334	1.612999491	0.598799693	0.9658636	53379	Hnrnpa2b1
O88844;A0A087WPT4;A0A087WRS9	Idh1	5	9	26.8	18	0	29.477	80462000	87314000	66152000	9858600	Idh1	557	0.921524612	0.822152072	0.757633369	15926	Idh1
O89023	Tpp1	1	3	7.5	3	0	8.6247	12637000	4702700	6028300	1176100	Tpp1	1255	2.687179705	0.477035689	1.281880622	12751	Tpp1
P00329	Adh1	1	1	2.4	1	0.0017564	1.7828	3656000	4911000	5333800	883070	Adh1	1329	0.744451232	1.458916849	1.086092446	11522	Adh1
P00397	Mtco1	1	1	2.7	3	0	3.7428	30199000	57088000	44640000	34984000	Mtco1	299	0.528990331	1.478194642	0.781950673	17708	Mtco1
P00405	Mtco2	1	7	35.2	54	0	60.352	3484000000	4448100000	3307700000	1.693E+09	Mtco2	15	0.783255772	0.949397245	0.743620872	17709	Mtco2
P00416	mt-Co3	1	1	5.4	10	0	3.1808	55483000	40804000	56852000	162990000	mt-Co3	127	1.359744143	1.024674225	1.393294775	17710	mt-Co3
P00493	Hprt1	1	5	25.7	6	0	8.7877	14973000	6852800	11890000	2314500	Hprt1	1020	2.184946299	0.79409604	1.735057203	15452	Hprt1
P00848	Mtatp6	1	2	8.4	21	0	5.5235	17543000	52219000	35620000	67293000	Mtatp6	232	0.335950516	2.030439492	0.682127195	17705	Mtatp6
P00920	Ca2	2	5	26.2	8	0	9.513	44967000	48741000	52902000	12637000	Ca2	498	0.922570321	1.176462739	1.085369607	12349	Ca2
P01027	C3	5	27	19.2	49	0	72.215	156350000	92290000	140830000	3904000	C3	837	1.694116372	0.900735529	1.525950807	12266	C3
P01029	C4b	1	1	0.9	3	0.0006154	2.1149	2077600	1345100	1616000	69505	C4b	1567	1.544569177	0.777820562	1.201397666	12268	C4b
P01887	B2m	1	2	15.1	2	0	2.4736	42524000	13665000	15548000	9397300	B2m	576	3.111891694	0.365628821	1.137797292	12010	B2m
P02089	Hbb-b2	1	1	44.2	2	0.0028637	1.6734	9284400	6685400	9639200	3806000	Hbb-b2	846	1.388757591	1.03821464	1.441828462	15130	Hbb-b2
P02802;A0A1D5RLN7	Mt1	2	1	32.8	4	0	3.2269	19343000	19615000	51012000	28141000	Mt1	327	0.986133061	2.637233108	2.600662758	17748	Mt1
P03888	Mtnd1	1	3	8.2	8	0	6.9357	117630000	222090000	171480000	98800000	Mtnd1	186	0.529650142	1.45779138	0.772119411	17716	Mtnd1
P03893	Mtnd2	1	1	4.9	3	0	39.124	9485300	13799000	13650000	4101600	Mtnd2	823	0.687390391	1.439068875	0.989202116	17717	mt-Nd2
P03899	Mtnd3	1	1	13	5	0	3.39	16106000	24988000	19346000	23142000	Mtnd3	357	0.644549384	1.201167267	0.774211622	17718	mt-Nd3
P03903	Mtnd4l	1	1	18.4	1	0.010037	1.2967	5274800	4297100	5505700	8007500	Mtnd4l	624	1.22752554	1.043774172	1.281259454	17720	Mtnd4l
P03911	Mtnd4	1	7	11.3	12	0	11.207	162390000	228400000	207490000	58788000	Mtnd4	243	0.710989492	1.277726461	0.908450088	17719	Mtnd4
P03921	Mtnd5	1	6	13.7	13	0	148.81	104450000	152940000	133840000	35808000	Mtnd5	294	0.682947561	1.28137865	0.875114424	17721	Mtnd5
P03930	Mtatp8	1	2	34.3	15	0	10.16	87890000	149920000	188110000	152580000	Mtatp8	138	0.586245998	2.140288998	1.254735859	17706	Mtatp8
P04117	Fabp4	6	9	59.1	47	0	45.831	851590000	1268800000	1057500000	440420000	Fabp4	63	0.671177491	1.24179476	0.833464691	11770	Fabp4
P04247;A0A2R8VK58	Mb	3	17	71.4	294	0	274.99	9369100000	1.4113E+10	1.3608E+10	5.943E+09	Mb	1	0.663863105	1.452434065	0.964217388	17189	Mb
P04919-2; P04919	Slc4a1	2	6	7.4	9	0	13.074	19753000	16937000	18493000	1910400	Slc4a1	1082	1.166263211	0.936212221	1.091869871	20533	Slc4a1
P05064;A6ZI44;D3YWI1;Q9CPQ9; A6ZI46;D3Z510;A0A0U1RPN8	Aldoa;Aldoart1	12	26	80.2	151	0	138.54	2814500000	4059900000	3773000000	516070000	Aldoa	50	0.693243676	1.340557826	0.929333235	11674	Aldoa;Aldoart1
P05132;P05132-2;P68181-2;P68181-3;P68181; P68181-4	Prkaca;Prkacb	8	6	16.5	12	0	12.176	49404000	50596000	55400000	9435200	Prkaca	574	0.976440825	1.121366691	1.094948217	18747	Prkaca;Prkacb
P05201	Got1	2	20	52.8	92	0	127.47	1107100000	2419600000	1529000000	250250000	Got1	88	0.457554968	1.381085719	0.631922632	14718	Got1
P05202	Got2	1	24	56.3	155	0	178.83	2710600000	4685800000	3580200000	496800000	Got2	56	0.578471126	1.32081458	0.764053097	14719	Got2
P05213;A0A2R8VHF3	Tuba1b	2	0	49.9	68	0	224.35	1660500000	831050000	1327200000	186630000	Tuba1b	119	1.998074725	0.799277326	1.597015823	22143	Gm49450; Tuba1b
P06728	Apoa4	1	8	21.8	14	0	24.344	23604000	22390000	19306000	2996800	Apoa4	934	1.054220634	0.817912218	0.862259937	11808	Apoa4
P06745	Gpi	5	18	34.8	52	0	74.132	642540000	685660000	754460000	86687000	Gpi	199	0.937111688	1.174183708	1.100341277	14751	Gpi
P06801;Q3TQP6	Me1	2	7	12.2	13	0	14.442	34872000	40351000	35109000	4103200	Me1	822	0.8642165	1.006796284	0.870089961	17436	Me1
P07310	Ckm	1	4	54.9	153	0	258.64	2908100000	5895400000	4109200000	768090000	Ckm	31	0.493282899	1.41301881	0.697018014	12715	Ckm
P07356;B0V2N7;B0V2N8;B0V2N5	Anxa2	4	18	54.3	41	0	130.03	505110000	137260000	271200000	39563000	Anxa2	279	3.679950459	0.536912752	1.975812327	12306	Anxa2
P07724	Alb	1	39	74	368	0	314.02	1.0902E+10	1.1405E+10	1.0321E+10	943570000	Alb	26	0.955896537	0.946707026	0.904953968	11657	Alb
P07901	Hsp90aa1	4	13	28.9	29	0	45.661	79439000	45478000	92427000	7345100	Hsp90aa1	640	1.746756674	1.163496519	2.03234531	15519	Hsp90aa1
P08113;F7C312	Hsp90b1	2	18	24.4	31	0	50.277	319990000	104380000	160590000	15109000	Hsp90b1	452	3.065625599	0.501859433	1.538513125	22027	Hsp90b1
P08122	Col4a2	1	4	2.9	5	0	8.2024	33525000	20561000	21424000	932240	Col4a2	1314	1.63051408	0.639045488	1.041972667	12827	Col4a2
P08207	S100a10	1	2	27.8	6	0	6.5202	18580000	23531000	14295000	13001000	S100a10	490	0.789596702	0.769375673	0.607496494	20194	S100a10
P08226;A0A1B0GX15;G3UWN5	Apoe	4	6	19.3	11	0	11.272	39375000	10207000	11964000	4379900	Apoe	795	3.857646713	0.303847619	1.172136769	11816	Apoe
P08228	Sod1	1	5	39	12	0	15.62	61685000	83128000	53888000	25421000	Sod1	345	0.742048407	0.873599741	0.648253296	20655	Sod1
P08249	Mdh2	3	18	65.7	222	0	195.5	6602200000	9652500000	8971000000	1.367E+09	Mdh2	17	0.683988604	1.358789494	0.929396529	17448	Mdh2
P08752;A0A0A6YWA9	Gnai2	15	5	25.4	20	0	25.85	96228000	62735000	59484000	13963000	Gnai2	469	1.533880609	0.618156877	0.948178848	14678	Gnai2
P09055	Itgb1	1	1	10.4	18	0	42.632	88608000	39882000	62620000	5822400	Itgb1	708	2.221754175	0.706708198	1.570131889	16412	Itgb1
P09103	P4hb	2	18	37.3	37	0	66.944	355510000	98188000	197070000	19289000	P4hb	394	3.620707215	0.554330399	2.007068073	18453	P4hb
P09405	Ncl	1	12	18	22	0	26.76	87945000	38039000	45596000	4890900	Ncl	763	2.311969295	0.518460401	1.198664529	17975	Ncl
P09411;S4R2M7	Pgk1	3	21	54	74	0	75.174	888100000	999590000	1023500000	122620000	Pgk1	164	0.88846427	1.152460309	1.023919807	18655	Pgk1
P09470;P09470-2;F6QCP8	Ace	3	6	4.9	8	0	12.166	30209000	7776300	27618000	932060	Ace	1315	3.884752389	0.914230858	3.551560511	11421	Ace
P09542;A0A0G2JDW2;A0A0G2JDM3	Myl3	7	13	82.8	224	0	200.48	9492200000	1.4532E+10	1.3779E+10	3.21E+09	Myl3	6	0.653192953	1.451612903	0.94818332	17897	Myl3
P09671;A0A3B2WBF0	Sod2	2	7	30.2	26	0	75.892	533370000	993220000	755040000	232900000	Sod2	93	0.537010934	1.415602677	0.760194116	20656	Sod2
P09813	Apoa2	1	2	18.6	10	0	8.987	84230000	108150000	90624000	60069000	Apoa2	241	0.778825705	1.075911196	0.837947295	11807	Apoa2
P0DN34		1	2	31.6	8	0	4.9085	182400000	416800000	203420000	322000000	P0DN34	79	0.437619962	1.115241228	0.488051823		Ndufb1
P0DP28;P0DP27;P0DP26;Q3UKW2;A0A3Q4EHJ0;Q9D6P8	Calm1;Calml3	8	7	45.6	21	0	29.407	285200000	202170000	197480000	80992000	Calm1	207	1.41069397	0.692426367	0.976801702	12313	Calm1;Calm2; Calm3;Calml3
P10107;A0A494BBD8	Anxa1	7	9	26.6	11	0	37.857	116410000	12917000	27347000	6961100	Anxa1	656	9.012154525	0.23491968	2.117132461	16952	Anxa1
P10126	Eef1a1	3	6	30.5	16	0	23.492	211450000	64884000	101550000	16889000	Eef1a1	420	3.258892793	0.48025538	1.565100795	13627	Eef1a1
P10493	Nid1	1	19	18.6	37	0	54.98	149180000	153460000	131530000	9687500	Nid1	567	0.972109996	0.881686553	0.857096312	18073	Nid1
P10518	Alad	1	2	6.4	3	0	5.1022	5993500	9110900	6064900	1361800	Alad	1202	0.657838413	1.011912906	0.66567518	17025	Alad
P10605	Ctsb	1	4	12.1	7	0	21.37	17146000	9277200	11261000	1979300	Ctsb	1070	1.848186953	0.656771259	1.213836071	13030	Ctsb
P10639	Txn	1	4	41	16	0	7.7091	133070000	111630000	93130000	48983000	Txn	264	1.192063065	0.699857218	0.834273941	22166	Txn
P10649;A2AE89;F6WHQ7	Gstm1	5	10	56.4	36	0	45.344	435170000	558620000	375260000	99818000	Gstm1	184	0.779008986	0.862329664	0.671762558	14862	Gstm1
P10833;A0A1B0GRG1	Rras	2	3	25.7	10	0	17.245	51219000	30191000	37218000	8032500	Rras	622	1.696498957	0.726644409	1.232751482	20130	Rras
P10922	H1f0	1	6	27.3	12	0	13.285	216330000	148950000	107570000	104440000	H1f0	178	1.452366566	0.497249572	0.722188654	14958	H1-0
P11087-2; P11087	Col1a1	3	6	5.7	8	0	17.202	127850000	4630400	11205000	2275600	Col1a1	1025	27.61100553	0.087641768	2.419877332	12842	Col1a1
P11152	[illegible]	1	4	[illegible]	6	0	7.7038	12526000	27811000	19281000	3259100	[illegible]	895	[illegible]	[illegible]	[illegible]	16956	[illegible]

P11352;A 0A0A6YY34;A 0A0A6YVV2	Gpxl	3	6	39.3	11	0	12.929	27089000	11912000	14773000	3750200	Gpxl	851	2.274093351	0.545350511	1.240177972	14775	Gpxl
P11404	Fabp3	1	9	60.2	146	0	62.817	4419500000	7435100000	8260200000	2.391E+09	Fabp3	9	0.594410297	1.869034959	1.110973625	14077	Fabp3
P11438	Lamp1	1	3	8.4	6	0	11.548	21283000	12542000	10945000	2876600	Lamp1	947	1.696938287	0.514260208	0.872667836	16783	Lamp1
P11499	Hsp90ab1	6	21	44.5	102	0	128.11	1037700000	846130000	930380000	94773000	Hsp90ab1	189	1.22640729	0.896578973	1.099570988	15516	Hsp90ab1
P11531	Dmd	7	47	14.8	85	0	130.98	228410000	197100000	266390000	3835600	Dmd	841	1.158853374	1.166279935	1.351547438	13405	Dmd
P11983-2; P11983	Tcp1	2	8	20.1	11	0	23.944	34342000	24678000	32051000	3658400	Tcp1	860	1.391603858	0.933288684	1.298768134	21454	Tcp1
P12382	Pfkl	1	6	15.9	12	0	14.911	20649000	11971000	16921000	1644600	Pfkl	1134	1.724918553	0.819458569	1.41349929	18641	Pfkl
P12787	Cox5a	1	9	54.1	56	0	34.743	1456900000	2461100000	1939100000	613250000	Cox5a	41	0.59197107	1.330976731	0.78789972	12858	Cox5a
P12815-2; P12815	Pdcd6	2	3	15.3	4	0	4.778	7789600	6444800	7961000	1921900	Pdcd6	1080	1.20866435	1.022003697	1.235259434	18570	Pdcd6
P12970	Rpl7a	2	9	35.3	13	0	16.394	58329000	35075000	49985000	13436000	Rpl7a	482	1.66297933	0.856949373	1.425089095	27176	Rpl7a
P13020-2;P13020; A0A0J9YUQ8	Gsn	6	11	17.4	20	0	22.315	97407000	24869000	34328000	5007100	Gsn	754	3.916804053	0.352418204	1.38035305	227753	Gsn
P13541	Myh3	2	2	13.7	18	0	4.1274	1878900000	2180200000	1794200000	61336000	Myh3	237	0.86180167	0.954920432	0.822952023	17883	Myh3
P14069	S100a6	1	3	25.8	4	0	3.8432	142220000	27294000	73566000	48108000	S100a6	265	5.210669012	0.51726902	2.695317652	20200	S100a6
P14094;A 0A0A6YX05	Atp1b1	2	8	28.6	18	0	31.54	197140000	295970000	240480000	67596000	Atp1b1	230	0.666081022	1.219843766	0.812514782	11931	Atp1b1
P14115	Rpl27a	1	3	22.3	7	0	5.775	42270000	22817000	28476000	14081000	Rpl27a	466	1.852566069	0.673669269	1.24801683	26451	Rpl27a
P14131	Rps16	1	8	41.8	16	0	14.259	198140000	90549000	146380000	40052000	Rps16	278	2.18820749	0.738770566	1.616583286	20055	Rps16
P14142;J3QK17	Slc2a4	2	3	8.1	3	0	9.2118	11591000	13413000	10980000	4380700	Slc2a4	794	0.864161634	0.947286688	0.818608812	20528	Slc2a4
P14148;F6XI62	Rpl7	2	8	31.1	15	0	16	83718000	60094000	80693000	17077000	Rpl7	417	1.393117449	0.963866791	1.342779645	19989	Rpl7
P14152;A 0A5F8MPN8	Mdh1	3	15	43.4	93	0	152.43	2813800000	4295900000	2999300000	837340000	Mdh1	27	0.654996625	1.065925084	0.698177332	17449	Mdh1
P14206;A 0A1L1SUK3	Rpsa	3	7	22.4	14	0	63.303	89281000	60237000	73452000	17824000	Rpsa	409	1.482162126	0.822705839	1.219383435	16785	Rpsa
P14211	Calr	1	12	31.5	21	0	18.218	368000000	154160000	200400000	30405000	Calr	311	2.387130254	0.544565217	1.299948106	12317	Calr
P14602-2;P14602; P14602-3;D3YZ06	Hspb1	4	10	61.4	33	0	61.159	308360000	220550000	521900000	73125000	Hspb1	219	1.398141011	1.69250227	2.366356835	15507	Hspb1
P14685	Psmd3	2	8	16.2	12	0	23.361	27514000	24257000	24542000	2279200	Psmd3	1024	1.13427052	0.891982264	1.011749186	22123	Psmd3
P14733	Lmnb1	1	12	23	17	0	23.149	87774000	34428000	38382000	4588800	Lmnb1	779	2.549494597	0.437282111	1.114848379	16906	Lmnb1
P14869;D3YVM5;S4R1N1	Rplp0	3	10	42.3	13	0	20.651	99595000	65410000	71944000	18684000	Rplp0	402	1.52262651	0.722365581	1.099892983	11837	Rplp0
P15105;D3YVK1	Glul	2	2	6.7	2	0	3.7867	8551100	10937000	12355000	1931400	Glul	1078	0.781850599	1.444843353	1.129651641	14645	Glul
P15626;D3YX76	Gstm2	6	7	53.2	27	0	45.45	99720000	128380000	95679000	23889000	Gstm2	353	0.776756504	0.959476534	0.745279639	14863	Gstm2
P15864	Hist1h1c	1	6	45.3	37	0	37.728	1568200000	816140000	956980000	385460000	Hist1h1c	70	1.921484059	0.610241041	1.172568432	50708	H1-2
P16045	Lgals1	2	8	71.9	34	0	63.458	603740000	232520000	341630000	135090000	Lgals1	154	2.596507827	0.565856163	1.469249957	16852	Lgals1
P16125;D3Z7F0	Ldhb	3	14	48.2	70	0	107.54	1668100000	3179100000	2269900000	482840000	Ldhb	61	0.524708251	1.360769738	0.714007109	16832	Ldhb
P16332	Mut	1	9	14.6	16	0	25.707	34033000	60272000	39264000	4492300	Mut	788	0.564656889	1.153703758	0.651446775	17850	Mmut
P16460	Ass1	1	2	5.6	2	0.010042	1.298	3504300	2256000	1866200	414000	Ass1	1476	1.553324468	0.53254573	0.827216312	11898	Ass1
P17047-3;P17047; P17047-2	Lamp2	3	3	7.2	3	0	7.1118	29956000	20280000	16526000	3563200	Lamp2	869	1.477120316	0.551675791	0.814891519	16784	Lamp2
P17182;Q6PHC1	Eno1	8	9	42.9	55	0	79.676	714080000	551230000	582880000	94576000	P17182	190	1.29543022	0.816267085	1.057417049	13806	Eno1
P17426-2; P17426	Ap2a1	5	6	10.5	11	0	12.153	44797000	16619000	28797000	1716100	Ap2a1	1119	2.695529214	0.642833225	1.732775739	11771	Ap2a1
P17427	Ap2a2	1	5	10	4	0	7.9143	12880000	7546100	7979700	610520	Ap2a2	1407	1.706841945	0.619541925	1.057460145	11772	Ap2a2
P17563;Q63836;A0A0R4J135;G3UYY2	Selenbp1;Selenbp2	7	14	35.2	30	0	34.242	107660000	185600000	100810000	15949000	Selenbp1	433	0.580064655	0.936373769	0.543157328	20341	Selenbp2;Selenbp1
P17665	Cox7c	1	2	28.6	11	0	9.5441	290480000	472280000	476820000	275680000	Cox7c	84	0.615058863	1.641489948	1.009612941	12867	Cox7c
P17742;A 0A1L1SST0	Ppia	5	7	47	20	0	18.312	617920000	216470000	336010000	121050000	Ppia	166	2.854529496	0.543775893	1.552224327	268373	Ppia
P17751;H7BXC3	Tpil	2	14	49.5	98	0	83.319	1447000000	1714100000	1800700000	291800000	Tpil	83	0.844174786	1.244436766	1.05052214	21991	Tpil
P18572;K3W4Q8;P18572-2;J3QP71	Bsg	4	9	23.7	20	0	39.005	105960000	130580000	119150000	20864000	Bsg	376	0.811456578	1.124480936	0.912467453	12215	Bsg
P18760;F8WGL3;A0A494B9A7	Cfl1	3	9	59.6	20	0	45.569	376480000	160280000	268930000	82176000	Cfl1	206	2.348889443	0.714327454	1.677876217	12631	Cfl1
P19123;E9Q8P0	Tnnc1	2	6	35.4	30	0	103.32	363750000	684840000	515210000	305360000	Tnnc1	80.5	0.531145961	1.41638488	0.752307108	21924	Tnnc1
P19157;A 0A494B908;A0A494BAW2;P46425;A0A494BAS3;A0A 494BAU3	Gstp1;Gstp2	9	6	40	14	0	127.03	108540000	103530000	84560000	27380000	Gstp1	333	1.048391771	0.779067625	0.816768087	14870	Gstp1;Gstp2
P19253;A 0A1B0GSL5;A0A1B0GSC2;A 0A1B0GRH1;A0A1B0GSB2;A 0A1B0GSQ6;A0A1B0GS68	Rpl13a	12	3	16.7	5	0	7.0225	33021000	17902000	19541000	8151500	Rpl13a	619	1.844542509	0.591774931	1.091554016	22121	Rpl13a;Gm45713
P19324	Serpinh1	4	11	35.3	31	0	97.134	296940000	69581000	122280000	21399000	Serpinh1	370	4.267544301	0.411800364	1.757376295	12406	Serpinh1
P19783	Cox4il	3	13	64.5	91	0	83.072	1311100000	1870500000	1649200000	514460000	Cox4il	51	0.700935579	1.257875067	0.881689388	12857	Cox4il
P20029	Hspa5	1	24	36.8	54	0	79.259	630400000	384820000	428580000	50612000	Hspa5	258	1.638168494	0.679854061	1.113715503	14828	Hspa5
P20065-2		1	2	31.8	9	0	9.908	136280000	37746000	61292000	122460000	P20065-2	165	3.610448789	0.449750514	1.623801197		
P20108	Prdx3	1	6	26.8	22	0	18.322	267230000	635060000	375010000	87029000	Prdx3	198	0.420794886	1.40332298	0.590511133	11757	Prdx3
P20152;A 0A0A6YWC8	Vim	21	30	70.2	128	0	254.27	8662100000	1077100000	2795300000	364740000	Vim	75	8.042057376	0.322704656	2.595209358	22352	Vim
P20918	Plg	2	7	9.7	14	0	13.813	29271000	19242000	23023000	1654300	Plg	1132	1.521203617	0.786546411	1.196497246	18815	Plg
P21550;Q5SX59	Eno3	5	16	43.5	121	0	83.023	1700900000	3140800000	2044500000	403300000	Eno3	66	0.541549924	1.2020107	0.650948803	13808	Eno3
P21614	Gc	2	11	22.5	17	0	17.917	68644000	64418000	61336000	9016800	Gc	589	1.065602782	0.893537673	0.95215623	14473	Gc
P21981	Tgm2	2	14	23.8	29	0	41.231	79468000	122130000	124920000	10501000	Tgm2	539	0.650683698	1.571953491	1.02284451	21817	Tgm2
P22599	Serpina1b	1	3	27.1	28	0	68.888	473820000	381160000	286650000	71040000	Serpina1b	223	1.24310001	0.604976573	0.752046385	20701	Serpina1b
P23116	Eif3a	1	2	1.3	2	0.0005886	1.8235	10716000	6738300	6927300	450560	Eif3a	1464	1.590312097	0.646444569	1.028048618	13669	Eif3a
P23242	Gja1	3	4	12.6	6	0	5.0874	13127000	23041000	15837000	3420900	Gja1	878	0.569723536	1.206444732	0.687339959	14609	Gja1
P23927;A 0A1L1SRG5	Cryab	2	9	51.4	63	0	72.139	1283600000	1706800000	2343100000	505760000	Cryab	52	0.752050621	1.825412901	1.372802906	12955	Cryab
P23953	Ces1c	9	5	17.5	15	0	20.126	66400000	72805000	45059000	8637800	Ces1c	600	0.912025273	0.678599398	0.618899801	13884	Ces1c
P24270;A 2AL20	Cat	2	18	40	32	0	34.912	86344000	107550000	111340000	10397000	Cat	542	0.802826592	1.28949319	1.035239424	12359	Cat
P24369	Ppib	1	8	36.1	15	0	17.38	173550000	54652000	78058000	25901000	Ppib	342	3.175547098	0.4497724	1.428273439	19035	Ppib
P24472	Gsta4	1	4	21.6	14	0	9.5056	36075000	59244000	37362000	15407000	Gsta4	445	0.608922423	1.035675676	0.630646141	14860	Gsta4
P24527	Lta4h	1	7	13.9	12	0	14.821	31318000	18562000	14942000	1789400	Lta4h	1103	1.68721043	0.477105818	0.804977912	16993	Lta4h
P24549	Aldh1a1	3	6	15.4	6	0	17.178	43051000	6092700	8484200	2376000	Aldh1a1	1015	7.065997013	0.197073239	1.392518916	11668	Aldh1a1
P24668	M6pr	1	2	7.9	2	0.0005942	1.8879	9091400	5067100	5261000	1256200	M6pr	1227	1.794201812	0.578678751	1.038266464	17113	M6pr
P25444;D3YVC1;D3YWJ3	Rps2	3	6	21.5	16	0	22.638	96901000	61039000	71506000	13645000	Rps2	475	1.587526008	0.737928401	1.171480529	16898	Rps2
P26039	Tln1	4	30	18.4	73	0	112.11	275220000	111790000	173540000	4304500	Tln1	802	2.461937562	0.630550105	1.552374989	21894	Tln1
P26041	Msn	2	18	35.9	54	0	56.48	532880000	219470000	451880000	36346000	Msn	291.5	2.428031166	0.847995796	2.058960222	17698	Msn
[illegible]	[illegible]	[illegible]	[illegible]	[illegible]	[illegible]	[illegible]	[illegible]	[illegible]	[illegible]	[illegible]	[illegible]	[illegible]	[illegible]	[illegible]	[illegible]	[illegible]	[illegible]	[illegible]

P26350	Ptma	4	6	35.1	15	0	20.492	101670000	24230000	36224000	52863000	Ptma	257	4.196037969	0.356289958	1.495006191	19231	Ptma
P26443	Glud1	2	17	38.5	37	0	51.125	197060000	205340000	155390000	19162000	Glud1	397	0.959676634	0.788541561	0.756744911	14661	Glud1
P26516	Psmd7	1	4	11.8	5	0	5.6147	16321000	12946000	14453000	2934900	Psmd7	940	1.260698285	0.885546229	1.116406612	17463	Psmd7
P26883;F6X9I3	Fkbp1a	2	2	25	6	0	5.289	29752000	23453000	28991000	28712000	Fkbp1a	320	1.268579713	0.974421888	1.236131838	14225	Fkbp1a
P27048;P63163;A0A0G2JGN4	Snrpb;Snrpn	3	2	6.5	5	0.0006006	1.9392	21886000	8734700	13074000	2998100	Snrpb	932.5	2.505638431	0.597368181	1.49678867	20638	Snrpb;Snrpn
P27546-3; P27546-2;P27546	Map4	4	5	10.2	17	0	16.332	60246000	28895000	44619000	2552000	Map4	991	2.084997404	0.740613485	1.544177193	17758	Map4
P27659;A0A2R8VHN4	Rpl3	4	6	17.6	10	0	10.219	110200000	11779000	49174000	8394400	Rpl3	614	9.355632906	0.446225045	4.174717718	27367	Rpl3
P27773	Pdia3	2	19	44.6	44	0	74.845	447270000	118970000	186580000	24444000	Pdia3	351	3.759519207	0.41715295	1.568294528	14827	Pdia3
P28271	Aco1	1	4	4.7	6	0	4.8158	10884000	8279400	12204000	779850	Aco1	1355	1.314588014	1.121278942	1.474019857	11428	Aco1
P28474	Adh5	2	7	26.2	9	0	13.063	88487000	98068000	74875000	14507000	Adh5	457	0.902302484	0.846169494	0.763500836	11532	Adh5
P28653	Bgn	1	13	46.3	31	0	55.991	828820000	33143000	113560000	57297000	Bgn	246	25.00739221	0.137014068	3.426364542	12111	Bgn
P28654	Dcn	1	8	22.6	19	0	70.036	222460000	28695000	31660000	15169000	Dcn	451	7.752570134	0.14231772	1.103328106	13179	Dcn
P28665;P28666	Mug1;Mug2	3	12	9.8	18	0	24.152	77032000	61656000	58061000	3048400	Mug1	924	1.249383677	0.753725724	0.941692617	17836	Mug1;Mug2
P29341;Q62029;Q9D4E6;A0A2I3BR37	Pabpc1;Pabpc2;Pabpc6	5	2	8.3	2	0	4.1673	9034000	2439200	3151000	410270	Pabpc1	1478	3.703673336	0.348793447	1.291816989	18458	Pabpc1;Pabpc2;Pabpc6
P29699;A0A338P703;A0A338P7G1;A0A338P7H5	Ahsg	7	5	20.3	6	0	19.556	39892000	36490000	42595000	10188000	Ahsg	549	1.093231022	1.067757946	1.167306111	11625	Ahsg
P29758	Oat	1	9	23.5	12	0	34.817	69299000	84004000	73494000	12628000	Oat	499	0.824948812	1.060534784	0.87488691	18242	Oat
P29788	Vtn	1	4	9.4	7	0	7.8022	33309000	4678600	6085300	2038100	Vtn	1057	7.119437439	0.182692365	1.300666866	22370	Vtn
P30412	Ppic	1	1	5.7	1	0.0080171	1.342	6331600	2097900	3022000	1026800	Ppic	1286	3.018065685	0.477288521	1.440488107	19038	Ppic
P30416	Fkbp4	2	3	5.9	3	0	2.4487	3742800	6763700	4154700	613410	Fkbp4	1405	0.553365761	1.110051298	0.614264382	14228	Fkbp4
P31001	Des	6	30	65.2	187	0	297.51	4585900000	3943900000	6941100000	486570000	Des	60	1.162783032	1.513574217	1.759958417	13346	Des
P31428;A0A1D5RML1;A0A1D5RM32	Dpep1	3	4	12.4	6	0	17.314	25576000	10033000	7980900	2118900	Dpep1	1044	2.549187681	0.31204645	0.795464966	13479	Dpep1
P31725	S100a9	1	1	12.4	2	0	3.6937	5610800	8371600	439300	2746600	S100a9	959	0.670218357	0.07829543	0.052475035	20202	S100a9
P32020-2; P32020	Scp2	2	6	39.9	9	0	16.219	39475000	60011000	51584000	16086000	Scp2	431	0.657796071	1.306751108	0.859575744	20280	Scp2
P32067;F6SXM5	Ssb	4	3	8.4	4	0	5.8714	8138800	3656400	5909900	794950	Ssb	1350	2.225905262	0.726138989	1.616316596	20823	Ssb
P32261	Serpinc1	6	8	18.1	13	0	14.81	51103000	35172000	21336000	3919300	Serpinc1	836	1.452945525	0.417509735	0.606618901	11905	Serpinc1
P32921-2; P32921	Wars	2	2	6.1	3	0	4.2248	6110300	5183200	4201200	596570	Wars	1411	1.178866337	0.687560349	0.81054175	22375	Wars1
P34022;H7BX22	Ranbp1	2	2	10.8	4	0	5.5745	8081100	7088600	6860100	3046100	Ranbp1	926	1.140013543	0.848906708	0.967765144	19385	Ranbp1
P34884	Mif	1	3	23.5	11	0	8.4033	149680000	125540000	170700000	78152000	Mif	211	1.19228931	1.140432924	1.359725984	17319	Mif
P34914;A8JYK8;P34914-2	Ephx2	3	12	28	27	0	107	90521000	147780000	97448000	13725000	Ephx2	474	0.612538909	1.07652368	0.65941264	13850	Ephx2
P34928	Apoc1	1	1	10.2	2	0.009979	1.2816	7706400	7136800	4600900	7428500	Apoc1	637	1.07981168	0.597023253	0.644672682	11812	Apoc1
P35235;P35235-1	Ptpn11	2	4	6.7	7	0	8.3147	16970000	14441000	17341000	1418400	Ptpn11	1188	1.175126376	1.02186211	1.200817118	19247	Ptpn11
P35279-2;P35279; D3YV69;P61294	Rab6a;Rab6b	6	3	14.9	9	0	12.691	61867000	47399000	59776000	15403000	Rab6a	446	1.305238507	0.966201691	1.261123652	19346	Rab6a;Rab6b
P35282	Rab21	2	4	23.9	9	0	10.388	20829000	18280000	16443000	4500400	Rab21	785.5	1.139442013	0.789428201	0.899507659	216344	Rab21
P35385	Hspb7	1	3	26	9	0	6.5468	24999000	22405000	53812000	11185000	Hspb7	523	1.115777728	2.152566103	2.401785316	29818	Hspb7
P35486	Pdha1	1	20	56.4	75	0	126.77	1208000000	1755400000	1522200000	247130000	Pdha1	90	0.688162242	1.260099338	0.867152786	18597	Pdha1
P35564	Canx	1	13	22.3	29	0	26.055	116000000	85217000	76694000	9343600	Canx	579	1.36123074	0.661155172	0.899984745	12330	Canx
P35700;B1AXW5;B1AXW6;B1AXW4	Prdx1	4	9	50.8	29	0	20.713	664880000	638330000	552670000	136580000	Prdx1	152	1.041592906	0.831232704	0.865806088	18477	Prdx1
P35979	Rpl12	1	3	28.5	8	0	14.734	29943000	16879000	20618000	7960700	Rpl12	626	1.773979501	0.688574959	1.221517862	269261	Rpl12
P37804;A0A1L1STN8	Tagln	2	10	52.7	20	0	36.608	126570000	32763000	75041000	14392000	Tagln	461	3.863199341	0.592881409	2.29041907	21345	Tagln
P38060	Hmgcl	1	5	18.2	9	0	35.556	25026000	23068000	22181000	4606300	Hmgcl	777	1.084879487	0.886318229	0.961548465	15356	Hmgcl
P38647	Hspa9	1	32	47	97	0	126.07	972720000	1282300000	993630000	99575000	Hspa9	185	0.758574437	1.021496422	0.774881073	15526	Hspa9
P40124	Cap1	2	12	32.3	11	0	18.895	86182000	20540000	25916000	4966400	Cap1	759	4.195813048	0.300712446	1.261733204	12331	Cap1
P40142;A0A286YE28	Tkt	2	8	17.7	10	0	16.588	62435000	11041000	20942000	3638700	Tkt	861	5.65483199	0.335420838	1.896748483	21881	Tkt
P40336;P40336-2	Vps26a	2	1	6.1	1	0	2.4666	5701100	5223700	3749900	864190	Vps26a	1333	1.09139116	0.657750259	0.717862818	30930	Vps26a
P41105	Rpl28	1	2	13.9	5	0.0006021	1.9737	19704000	11911000	20609000	6628700	Rpl28	671	1.654269163	1.04592976	1.730249349	19943	Rpl28
P42125;A0A3Q4EC00;A0A452J8A5	Ecil	4	10	39.1	53	0	46.799	427760000	1113200000	507580000	145960000	Ecil	144	0.384261588	1.186599963	0.455964786	13177	Ecil
P42669	Pura	1	4	16.5	7	0	5.6791	22406000	17315000	15026000	6860200	Pura	661	1.294022524	0.67062394	0.867802483	19290	Pura
P42932;H3BL49;H3BJB6	Cct8	5	13	26.1	23	0	23.269	89141000	54860000	77674000	6551100	Cct8	675	1.624881517	0.871361102	1.415858549	12469	Cct8
P43023	Cox6a2	1	1	12.4	8	0	4.6023	176240000	273080000	177490000	146480000	Cox6a2	142	0.645378644	1.007092601	0.649956057	12862	Cox6a2
P43274	Hist1h1e	3	2	33.8	8	0	3.8405	67898000	31088000	30032000	15388000	Hist1h1e	447	2.184058157	0.442310525	0.966031909	50709	H1-4
P43276	Hist1h1b	1	6	32.7	10	0	9.031	187550000	25246000	73377000	30404000	Hist1h1b	312	7.428899628	0.391239669	2.906480234	56702	H1-5
P43277	Hist1h1d	1	1	38	5	0	4.244	386730000	90979000	198290000	69086000	Hist1h1d	228	4.250761165	0.512734983	2.179513954	14957	H1-3
P45376;D3YVJ7	Akr1b1;Akr1b3	2	14	49.4	43	0	50.285	341640000	488060000	388340000	73307000	Akr1b1	217	0.699995902	1.136693596	0.795680859	11677	Akr1b1;Akr1b3
P45591;A0A1Y7VJ71	Cfl2	2	6	42.8	10	0	73.558	82622000	72288000	140690000	25894000	Cfl2	343	1.142955954	1.702815231	1.946242807	12632	Cfl2
P45952	Acadm	2	20	53	86	0	105.1	1987900000	4025200000	2126200000	496890000	Acadm	55	0.493863659	1.069570904	0.5282222	11364	Acadm
Q3P9;F8WGS1;G3UZD3;P62492;G3UZL4	Rab11b;Rab11a	10	10	46.3	24	0	30.095	142450000	107440000	142920000	27387000	Rab11b	332	1.325856292	1.003299403	1.330230827	19326	Rab11b;Rab11a
P46935;A0A571BDN7	Nedd4	11	5	9.5	8	0	16.227	23127000	14867000	17100000	1382400	Nedd4	1197	1.555592924	0.739395512	1.150198426	17999	Nedd4
P46978	Stt3a	2	4	6.1	4	0	8.2069	15697000	10310000	6271900	1333700	Stt3a	1205	1.522502425	0.399560426	0.608331717	16430	Stt3a
P47738;A0A0G2JEU1	Aldh2	5	15	42.4	46	0	108.26	442330000	649430000	363660000	72270000	Aldh2	221	0.681104969	0.822146361	0.559967972	11669	Aldh2
P47754;A0A0N4SVM0;D6RCW7	Capza2	3	7	34.6	15	0	21.731	69058000	60784000	70637000	16093000	Capza2	430	1.136121348	1.022864838	1.162098579	12343	Capza2
P47757;P47757-2;A2AMW0;P47757-4	Capzb	7	8	31.4	11	0	24.868	58930000	44222000	50830000	10018000	Capzb	553	1.332594636	0.862548787	1.149427887	12345	Capzb
P47791-2; P47791	Gsr	2	2	4.9	3	0	5.4088	3441400	3065500	4161700	569220	Gsr	1421	1.122622737	1.209304353	1.357592562	14782	Gsr
P47857;P47857-2;P47857-3	Pfkm	5	22	35.8	52	0	131.53	529070000	873630000	624040000	70139000	Pfkm	227	0.605599625	1.179503657	0.714306972	18642	Pfkm
P47911	Rpl6	1	7	20.3	13	0	14.601	121390000	74814000	101720000	22515000	Rpl6	362	1.622557275	0.837960293	1.35963857	19988	Rpl6
P47915;A0A1L1SUN1;A0A1L1SS27;W4VSN7	Rpl29;Gm3550	4	2	11.9	2	0.0006231	2.2173	24186000	14880000	16561000	11014000	Rpl29	528	1.625403226	0.684734971	1.11297043	19944	Rpl29;Gm3550
P47962;D3YYV8	Rpl5	2	4	15.2	5	0	9.6812	14125000	5629600	10083000	2710700	Rpl5	963	2.509059258	0.713840708	1.791068637	100503670	Rpl5
P47963	Rpl13	1	7	34.1	18	0	37.455	90965000	58889000	84963000	27085000	Rpl13	335	1.544685765	0.934018579	1.442765202	270106	Rpl13
A3;J3QPZ8;Q9CXU9	Eif1; Eif1b	5	3	41.6	5	0	8.2739	7595900	10824000	11606000	7175500	Eif1	649	0.701764597	1.527929541	1.072246859	20918	Eif1; Eif1b

P48678;P48678-2;P48678-3	Lmna	4	38	54.3	92	0	206.19	1346300000	357740000	576530000	54764000	Lmna	254	3.763347683	0.428232935	1.611589422	16905	Lmna
P48722-2;P48722;E0CY23	Hspa4l	3	3	6.4	5	0	7.1007	6604100	4396800	5797100	377270	Hspa4l	1487	1.502024199	0.877803183	1.318481623	18415	Hspa4l
P48758	Cbr1	5	5	20.9	7	0	7.2145	19425000	16853000	15244000	3452500	Cbr1	875	1.152613778	0.784761905	0.904527384	12408	Cbr1
P48771	Cox7a2	1	2	27.7	12	0	7.8829	143170000	173860000	208490000	146190000	Cox7a2	143	0.823478661	1.456240833	1.199183251	12866	Cox7a2
P48787;D3YXP5	Tnni3	4	10	54	78	0	279.2	2007800000	2773600000	2629800000	1.112E+09	Tnni3	21	0.723896741	1.309791812	0.948154024	21954	Tnni3
P48962	Slc25a4	1	10	55.4	291	0	114.83	1.1319E+10	1.817E+10	1.5362E+10	2.492E+09	Slc25a4	8	0.622949917	1.357187031	0.845459549	11739	Slc25a4
P49182	Serpind1	1	2	2.9	2	0.004918	1.4514	3735500	1363500	1367100	323490	Serpind1	1510	2.739640631	0.365975104	1.002640264	15160	Serpind1
P49813	Tmod1	1	9	30.4	14	0	37.839	37768000	53021000	55581000	8278600	Tmod1	617	0.712321533	1.47164266	1.048282756	21916	Tmod1
P49817;H3BKG0;D3Z148;P49817-2;D3Z0J2	Cav1	5	5	39.3	16	0	18.659	204980000	223260000	221730000	73188000	Cav1	218	0.918122369	1.081715289	0.993147003	12389	Cav1
P50171;P50171-2;G3UX44;A0A494BA51;A0A494BAF4	Hsd17b8; H2-Ke6	5	7	36.7	13	0	23.487	42552000	81311000	50366000	16176000	Hsd17b8	427	0.523324027	1.183634142	0.619424186	14979	H2-Ke6;Hsd17b8
P50247	Ahcy	2	8	19.2	11	0	14.34	46475000	31551000	36130000	5165300	Ahcy	744	1.473011949	0.777407208	1.145130107	11615	Ahcy
P50396	Gdi1	3	7	24.2	14	0	17.144	18089000	15102000	15687000	2029500	Gdi1	1060	1.197788372	0.867212118	1.038736591	14567	Gdi1
P50462	Csrp3	2	12	67	52	0	86.74	885820000	1255500000	1607900000	398310000	Csrp3	69	0.705551573	1.81515432	1.280684986	13009	Csrp3
P50543	S100a11	1	2	20.4	4	0	11.343	70566000	8824300	22029000	19885000	S100a11	387	7.996781614	0.312175835	2.496401981	20195	S100a11
P50544;B1AR28	Acadvl	2	36	57.3	151	0	192.62	1547500000	3148900000	1948700000	196580000	Acadvl	111	0.491441456	1.259256866	0.618851027	11370	Acadvl
P50580-2; P50580	Pa2g4	2	5	15.3	7	0	5.6431	29557000	12791000	19010000	3214300	Pa2g4	904	2.310765382	0.643164056	1.486201235	18813	Pa2g4
P51150;A0A0N4SVR6;A0A0N4SVG9	Rab7a	3	8	47.3	15	0	24.919	57780000	36098000	40959000	10339000	Rab7a	543	1.600642695	0.708878505	1.1346612	19349	Rab7;Rab7a
P51637	Cav3	1	1	14.6	3	0	12.337	8365200	8576300	10315000	4178500	Cav3	814	0.975385656	1.233084684	1.202733113	12391	Cav3
P51660	Hsd17b4	1	8	11.7	11	0	17.712	29624000	36737000	37442000	3200400	Hsd17b4	908	0.806380488	1.263907642	1.019190462	15488	Hsd17b4
P51667;E9Q8Y0;F6RBQ5	Myl2	3	4	64.5	216	0	163.46	5651700000	9647800000	7271500000	2.602E+09	P51667	7	0.585801944	1.286604031	0.753695143	17906	Myl2
P51859;E0CYW7;E0CXA0	Hdgf	3	2	8	2	0.0034014	1.6296	14132000	4364000	4309200	1554200	Hdgf	1155	3.238313474	0.304924993	0.987442713	15191	Hdgf
P51881	Slc25a5	1	7	45	28	0	35.475	663370000	708890000	627220000	134980000	Slc25a5	155	0.935786935	0.945505525	0.884791717	11740	Slc25a5
P51885	Lum	2	10	36.1	17	0	83.28	776660000	36575000	104590000	56313000	Lum	250	21.23472317	0.134666392	2.859603554	17022	Lum
P52196	Tst	1	5	15.8	5	0	5.9743	12789000	17804000	18086000	3673000	Tst	859	0.718321725	1.414184064	1.015839137	22117	Tst
P52480-2	Pkm	1	2	55.9	114	0	154.74	2152500000	2476800000	2284600000	223970000	Pkm	98	0.869064922	1.061370499	0.922399871		
P52503;A0A1Y7VM38;A0A1Y7VKP8	Ndufs6	3	5	60.3	21	0	54.921	301600000	533580000	290930000	189040000	Ndufs6	117	0.565238577	0.964622016	0.545241576	407785	Ndufs6
P52785	Gucy2e	1	1	0.7	3	0.0033841	1.6087	50909000	44620000	52424000	3238700	Gucy2e	897	1.140945764	1.029758982	1.174899148	14919	Gucy2e
P52825	Cpt2	4	24	40.9	57	0	79.463	322040000	670430000	436090000	49257000	Cpt2	263	0.480348433	1.354148553	0.650463136	12896	Cpt2
P53395	Dbt	2	12	22	18	0	24.319	58131000	143320000	72262000	11366000	Dbt	518	0.405602847	1.243088885	0.504200391	13171	Dbt
P53702	Hccs	2	4	16.2	7	0	6.6327	9376200	14617000	11689000	2715000	Hccs	962	0.641458576	1.246667093	0.799685298	15159	Hccs
P53986	Slc16a1	1	4	8.3	9	0	17.427	26616000	60477000	44590000	10030000	Slc16a1	552	0.440101195	1.675308085	0.737305091	20501	Slc16a1
P53994;Q3TEG7;P59279	Rab2a;Rab2b	5	7	40.1	10	0	12.684	25197000	18611000	21805000	4922500	Rab2a	761	1.35387674	0.865380799	1.171618935	59021	Rab2b;Rab2a
P53996-2; P53996-3;A0A0N4SVS6;P53996	Cnbp	4	3	21.8	5	0	7.2547	29282000	12284000	16527000	4998500	Cnbp	757	2.383751221	0.564408169	1.345408662	12785	Cnbp
P54071	Idh2	4	23	54.4	275	0	323.31	4775300000	1.0545E+10	6817300000	1.107E+09	Idh2	22	0.452849692	1.427617113	0.64649597	269951	Idh2
P54116	Stom	1	6	27.5	12	0	17.899	24181000	28596000	26699000	5914000	Stom	701	0.845607777	1.104131343	0.933662051	13830	Stom
P54728;Q3TN85;Q8CAP3;P54726	Rad23b;Rad23a	4	2	4.1	4	0	3.531	21006000	17658000	19815000	4848000	Rad23b	767	1.189602446	0.943301914	1.122154264	19359	Rad23a;Rad23b
P55264;P55264-2;A0A286YCD7	Adk	4	5	11.9	8	0	6.9806	57521000	60721000	67152000	9841100	Adk	560	0.947299946	1.167434502	1.10591064	11534	Adk
P55302	Lrpap1	2	5	13.1	7	0	8.5726	17686000	8978700	12134000	2072500	Lrpap1	1048	1.969772907	0.686079385	1.351420584	16976	Lrpap1
P56135;F8WHP8	Atp5j2	2	4	36.4	10	0	9.657	126270000	365970000	164710000	208960000	Atp5j2	108	0.345028281	1.304427021	0.450064213	57423	Atp5j2;Atp5mf
P56375	Acyp2	1	4	34.9	9	0	24.138	67318000	85247000	87751000	33861000	Acyp2	300	0.789681748	1.303529517	1.029373468	75572	Acyp2
P56379;A0A1Y7VLP0	Mp68	2	2	29.3	13	0	8.9306	218310000	41271000	208300000	137970000	Mp68	150	5.289670713	0.954147772	5.047127523	70257	Atp5mpl
P56380	Nudt2	1	2	10.2	4	0	2.7261	4539200	6677900	5733500	1973300	Nudt2	1073	0.679734647	1.263108037	0.858578296	66401	Nudt2
P56382	Atp5e	1	3	44.2	6	0	3.0636	528080000	720680000	686010000	722250000	Atp5e	32	0.732752401	1.299064536	0.951892657	67126	Atp5f1e
[illegible]3	Cox6b1	2	7	70.9	61	0	60.573	975570000	1745800000	1219700000	781480000	Cox6b1	29	0.558809715	1.250243447	0.698648184	110323	Cox6b1
P56394	Cox17	1	2	46	5	0	6.5538	6288500	5346300	6654100	5320400	Cox17	734	1.176234031	1.058137871	1.244617773	12856	Cox17
P56480	Atp5b	1	22	54.6	512	0	323.31	1.189E+10	1.8452E+10	1.3398E+10	1.782E+09	Atp5b	13	0.644374594	1.126829268	0.726100152	11947	Atp5f1b
P56812;D3Z7Q5	Pdcd5	2	3	26.2	3	0	7.8182	11265000	6695400	7316800	2450200	Pdcd5	1006	1.682498432	0.649516201	1.092809989	56330	Pdcd5
P57759	Erp29	4	3	11.8	3	0	6.5957	26227000	10694000	15274000	4337200	Erp29	799	2.452496727	0.58237694	1.428277539	67397	Erp29
P58252	Eef2	5	27	32.8	53	0	66.765	678720000	472310000	656890000	42703000	Eef2	274	1.437022295	0.967836516	1.390802651	13629	Eef2
P58281-2;P58281; H7BX01; E0CXD1	Opa1	6	22	23.8	40	0	62.286	82398000	160620000	109350000	7541300	Opa1	636	0.512999626	1.327095318	0.680799402	74143	Opa1
P58389	Ppp2r4	1	2	5.3	4	0	3.4596	22503000	10040000	13630000	3011900	Ppp2r4	930	2.241334661	0.605697018	1.357569721	110854	Ptpa
P58771-2; E9Q450	Tpm1	2	0	68.7	1	0.0079745	1.3203	29868000	5109700	8954400	2660100	P58771-2	972	5.845352956	0.299799116	1.75243165	22003	Tpm1
P58771;Q8BSH3;E9Q452;E9Q454;Q8BP43;S4R2U0	Tpm1	6	0	76.1	308	0	323.31	1.4618E+10	2.5006E+10	1.7365E+10	3.891E+09	P58771	4	0.584579701	1.187919004	0.694433336	22003	Tpm1
P58774-2; A2AIM4	Tpm2	2	2	43.7	10	0	14.361	31308000	11767000	16366000	3779500	P58774-2	849	2.660661171	0.522741791	1.390838786	22004	Tpm2
P59017	Bcl2l13	1	5	12.7	11	0	25.528	48087000	69084000	60661000	13054000	Bcl2l13	489	0.696065659	1.261484393	0.878075965	94044	Bcl2l13
P59266	Fitm2	1	2	8.4	2	0	3.8091	5211300	10364000	4862900	4158500	Fitm2	815	0.502827094	0.93314528	0.469210729	228859	Fitm2
P59999	Arpc4	3	4	25	9	0	8.3094	79343000	27018000	38857000	16518000	Arpc4	423	2.936671848	0.489734444	1.438189355	68089	Arpc4
P60335	Pcbp1	1	3	15.2	9	0	8.5773	47822000	29076000	41911000	7304300	Pcbp1	642	1.644724171	0.876395801	1.441429358	23983	Pcbp1
P60603	Romo1	1	1	21.5	4	0	3.6565	4538200	5598700	5198400	4273100	Romo1	806	0.810581028	1.14547618	0.928501259	67067	Romo1
[illegible]5F4;E9Q1F2;G3UZ07	Actb;Actg1	14	7	57.1	61	0	228.95	1506600000	319050000	502870000	125240000	Actb	161	4.722143865	0.333778043	1.576147939	11461	Actb;Actg1
P60766-1; P60766	Cdc42	2	3	22.5	6	0	7.4888	12410000	8598100	9094800	3422500	Cdc42	877	1.443342134	0.732860596	1.057768577	12540	Cdc42
P60843	Eif4a1	1	4	29.8	26	0	37.802	150220000	90800000	123710000	16974000	Eif4a1	419	1.654405286	0.823525496	1.362444934	13681	Eif4a1
P60867	Rps20	1	2	19.3	9	0	6.5628	39968000	30749000	25688000	21201000	Rps20	372	1.299814628	0.642714171	0.835409282	67427	Rps20
P61014	Pln	1	2	23.1	9	0	3.8817	87275000	76732000	100630000	139060000	Pln	149	1.137400302	1.153022057	1.311447636	18821	Pln
P61027	Rab10	1	3	18.5	4	0	2.6968	13207000	5787200	9557900	2004100	Rab10	1065	2.282105336	0.723699553	1.651558612	19325	Rab10
P61028;P55258	Rab8b;Rab8a	11	2	25.1	5	0	4.5642	18615000	10514000	10725000	3551000	Rab8b	870	1.770496481	0.576148268	1.02006848	235442	Rab8a;Rab8b
P61082;F7CDT0;F6WMC0	Ube2m	4	4	25.1	8	0	8.6224	14407000	14567000	15201000	4360300	Ube2m	797	0.98901627	1.055112098	1.043523032	22192	Ube2m
P61089	Ube2n	4	3	18.4	8	0	11.409	67265000	58389000	60750000	20195000	Ube2n	381	1.152014934	0.90314428	1.040435699	93765	Ube2n
P61161	Actr2	1	5	14.7	10	0	9.7413	39825000	16910000	18064000	5618400	Actr2	718	2.355115316	0.453584432	1.068243643	66713	Actr2
P61202;P61202-2;A2AQE4	Cops2	3	4	9.7	7	0	7.7653	5451800	6572700	5832500	853290	Cops2	1337	0.829461256	1.069830148	0.887382659	12848	Cops2
P61255;B1ARA3;B1																		

P61358	Rpl27	2	4	36	8	0	7.0015	54412000	32703000	41464000	22816000	Rpl27	359	1.663822891	0.762037786	1.267895912	108167922	Rpl27
P61514;A0A1D5RL86	Rpl37a	2	2	18.5	4	0	2.9036	13129000	15275000	20258000	12175000	Rpl37a	502	0.859509002	1.54299691	1.326219313	19981	Rpl37a
P61620;Q9CYJ6;Q9JLR1	Sec61a1;Sec61a2	4	3	6.5	3	0	2.7563	20034000	5462500	9820300	2166400	Sec61a1	1036	3.667551487	0.490181691	1.79776659	53421	Sec61a2;Sec61a1
P61750;F6UFB9;E9Q798	Arf4	3	3	23.9	3	0	6.2891	29101000	6141200	6035500	4562800	Arf4	782	4.738650427	0.207398371	0.98278838	11843	Arf4
P61804	Dad1	1	2	19.5	3	0	4.0942	7783400	3662500	4280700	3083600	Dad1	919	2.12516041	0.549978159	1.168791809	13135	Dad1
P61971	Nutf2	1	2	17.3	4	0	12.212	5363500	6263300	5010900	2691800	Nutf2	965	0.856337713	0.934259346	0.800041512	68051	Nutf2
P61982	Ywhag	1	6	42.9	15	0	44.502	335400000	240220000	350630000	59373000	Ywhag	242	1.396220132	1.045408468	1.459620348	22628	Ywhag
P62071;A0A1B0GRT5	Rras2	2	2	25	4	0	6.3927	6309700	4586500	7727200	1262600	Rras2	1224	1.375711327	1.224654104	1.684770522	66922	Rras2
P62073	Timm10	1	2	16.7	2	0.0028986	1.7246	15300000	20112000	18850000	9258600	Timm10	581	0.760739857	1.232026144	0.937251392	30059	Timm10
P62075;A0A1W2P7H2	Timm13	3	5	56.8	16	0	47.587	66761000	81300000	84338000	47296000	Timm13	267	0.821168512	1.263282455	1.037367774	30055	Timm13
P62077	Timm8b	1	3	38.6	6	0	5.73	11060000	9846400	10050000	5034800	Timm8b	749	1.123253169	0.908679928	1.020677608	30057	Timm8b
P62082	Rps7	1	1	4.1	2	0.0049423	1.4724	21931000	13735000	14943000	5427000	Rps7	727	1.596723699	0.681364279	1.087950491	20115	Rps7
P62141;P63087;P63087-2;A0A0G2JGC1;A0A0G2JFF1	Ppp1cb;Ppp1cc	7	3	25.7	23	0	23.772	54936000	53418000	66442000	10933000	Ppp1cb	532	1.028417387	1.209443716	1.243812947	19046	Ppp1cc;Ppp1cb
P62192	Psmc1	1	4	10.5	5	0	5.9688	18332000	16831000	25521000	2620800	Psmc1	979	1.089180679	1.392155793	1.516309191	19179	Psmc1
P62242	Rps8	1	7	39.4	18	0	44.13	79586000	50076000	65939000	23420000	Rps8	355	1.589304258	0.828525117	1.316778497	20116	Rps8
P62259;D6REF3;F6WA09	Ywhae	3	10	49.4	27	0	30.569	345610000	289840000	348610000	60988000	Ywhae	238	1.192416506	1.008680304	1.202767044	22627	Ywhae
P62264;D3YVF4;D3Z7I1	Rps14	3	5	36.4	16	0	8.8866	47140000	31409000	37554000	30301000	Rps14	314	1.500843707	0.796648282	1.19564456	20044	Rps14
P62267	Rps23	1	2	13.3	5	0	4.1466	38657000	29326000	31623000	18659000	Rps23	403	1.318181818	0.818040717	1.0783264	66475	Rps23
P62274	Rps29	1	2	32.1	3	0.0006278	2.2703	8229500	3791800	7482500	10249000	Rps29	546	2.170341263	0.909228993	1.973337201	20090	Rps29
P62281;A0A1B0GRR3;A0A1B0GSE8	Rps11	3	6	33.5	9	0	8.2879	56631000	36321000	39651000	13866000	Rps11	472	1.559180639	0.700164221	1.091682498	27207	Rps11
P62317	Snrpd2	1	2	16.9	3	0	3.9436	3746800	1891100	2455100	1197300	Snrpd2	1249	1.981280736	0.655252482	1.29823912	107686	Snrpd2
P62320	Snrpd3	1	2	15.1	3	0	9.5003	19452000	10450000	11433000	7222000	Snrpd3	645	1.861435407	0.587754473	1.094066986	67332	Snrpd3
P62334	Psmc6	1	5	14.7	6	0	8.6838	9272600	13760000	6020000	1631900	Psmc6	1137	0.673880814	0.649224597	0.4375	67089	Psmc6
P62627;A2AVR9	Dynlrb1	2	2	21.9	3	0	5.0479	22415000	13896000	19520000	10066000	Dynlrb1	551	1.613054116	0.870845416	1.404720783	67068	Dynlrb1
P62631	Eef1a2	1	7	38.2	79	0	73.358	1144900000	1375000000	1128400000	198870000	Eef1a2	110	0.832654545	0.985588261	0.820654545	13628	Eef1a2
P62702;V9GWY0	Rps4x;Gm15013	2	6	24.7	10	0	10.575	89601000	60765000	80085000	15365000	Rps4x	448	1.474549494	0.893795828	1.317946186	20102	Rps4x
P62748;A2A7R5;A0A1Y7VMK3;E9PV73;P84075	Hpcal1;Hpca	5	2	10.9	1	0.0006173	2.1272	5938500	4767600	4467700	1146800	Hpcal1	1259	1.245595268	0.752328029	0.937096233	53602	Hpca;Hpcal1
P62754	Rps6	1	4	18.1	11	0	21.232	92534000	47251000	70976000	26402000	Rps6	341	1.958350088	0.767026174	1.502105776	105244208	Rps6
P62774;A0A0J9YV46	Mtpn	2	2	22.9	3	0	4.9084	11455000	3736200	6653300	3683600	Mtpn	858	3.06594936	0.580820602	1.780766554	14489	Mtpn
P62806	Hist1h4a	1	10	60.2	87	0	54.391	3392100000	1770200000	1743300000	1.251E+09	Hist1h4a	19	1.916224155	0.513929424	0.984803977	100041230	H4c1; H4c2; H4c3; H4c4; H4c6; H4c8; H4c9; H4c11; H4c12; Hist1h4m; H4c14; H4c16
P62814	Atp6v1b2	3	5	12.3	6	0	7.4574	14652000	8527900	8005700	1235100	Atp6v1b2	1237	1.718125213	0.546389571	0.938765698	11966	Atp6v1b2
P62821;Q5SW88;Q5SW87;A0A494BA38;A0A494BBL7	Rab1A;Rab1	8	4	40	18	0	12.523	254980000	177120000	232720000	45026000	Rab1A	271	1.439588979	0.912699035	1.313911472	19324	Rab1a;Rab1b;Rab1A
P62827;Q14AA6;Q61820	Ran;1700009N14Rik;Rasl2-9	3	7	36.1	14	0	21.007	120030000	68800000	109990000	26900000	Ran	337	1.744622093	0.916354245	1.59869186	19384	1700009N14Rik;Ran;Rasl2-9
P62830	Rpl23	2	4	38.6	9	0	14.445	46088000	26378000	38505000	13258000	Rpl23	485	1.747213587	0.835466933	1.459739177	65019	Rpl23
P62855	Rps26	1	3	27	7	0	5.303	79515000	44927000	56364000	36759000	Rps26	289	1.769871124	0.708847387	1.254568522	27370	Rps26
P62869;A0A3B2WBM3	Tceb2	2	6	51.7	13	0	12.68	55307000	47963000	54884000	17063000	Tceb2	418	1.153118028	0.992351782	1.14429873	67673	Elob
P62874;H3BKR2;H3BLF7	Gnb1	4	3	23.8	5	0	3.5486	27580000	13243000	18480000	5032700	Gnb1	751	2.082609681	0.670050761	1.395454202	14688	Gnb1
P62880;D3Z1M1;D3Z1T4;E9QKR0;D3YZX3;P29387	Gnb2;Gnb4	9	4	22.1	14	0	19.684	168030000	100460000	110050000	29974000	Gnb2	315	1.672606012	0.65494257	1.09546088	14693	Gnb2;Gnb4
P62889;A0A2I3BQF4	Rpl30	2	5	49.6	12	0	17.087	65173000	39006000	56737000	27801000	Rpl30	328	1.670845511	0.870559894	1.454571092	19946	Rpl30
P62897	Cycs	3	7	55.2	66	0	41.219	1870800000	3008300000	2220300000	1.364E+09	Cycs	18	0.621879467	1.186818473	0.738058039	13063	Cycs
P62900;A0A0A6YX26	Rpl31	2	3	26.4	6	0	8.1276	43890000	30740000	38673000	16541000	Rpl31	422	1.427781392	0.881134655	1.258067664	114641	Rpl31
P62908;D3YV43	Rps3	3	11	44	20	0	19.16	151980000	87159000	113930000	20366000	Rps3	379	1.743709772	0.74963811	1.307151298	27050	Rps3
P62918	Rpl8	1	3	13.2	8	0	7.2001	35768000	29588000	27700000	9909500	Rpl8	555	1.20886846	0.774435249	0.936190347	26961	Rpl8
P62960;A2BGG7	Ybxl	6	3	23	8	0	5.8365	9493600	2082200	1893300	1088800	Ybxl	1272	4.559408318	0.199429089	0.909278648	22608	Ybxl
P62983;A0A0A6YW67;E9Q9J0;E9Q4P0;E9Q5F6;E9QNP0;Q5SX22;P62984;P0CG49;P0CG50	Rps27a;Gm8797;Uba52;Kxd1;Ubc;Ubb	10	4	34	17	0	19.101	162630000	117940000	158550000	78418000	Rps27a	210	1.378921486	0.974912378	1.344327624	78294	Kxd1;Uba52;Ubb;Ubc;Gm8797;Rps27a
P63005;P63005-2	Pafah1b1	3	6	18	12	0	12.241	19615000	18697000	18194000	2513000	Pafah1b1	1001	1.049098786	0.927555442	0.973097288	18472	Pafah1b1
P63017;Q504P4	Hspa8	4	28	52.8	118	0	130.8	2407600000	1793900000	2455300000	209710000	Hspa8	105	1.342103796	1.019812261	1.368693907	15481	Hspa8
P63028;D3YU75	Tpt1	2	4	21.5	12	0	34.794	43252000	77501000	72010000	33441000	Tpt1	302	0.558083122	1.664894109	0.929149301	22070	Tpt1
P63038	Hspd1	4	25	49.4	88	0	163.03	1077100000	1724000000	1154600000	140420000	Hspd1	148	0.624767981	1.071952465	0.669721578	15510	Hspd1
P63101;A0A2I3BQ03	Ywhaz	5	9	52.2	40	0	83.286	1288900000	637410000	958880000	209030000	Ywhaz	107	2.022089393	0.743952207	1.504337867	22631	Ywhaz
P63276	Rps17	1	1	7.4	3	0	2.6484	11756000	7222900	11571000	6274700	Rps17	683	1.627601102	0.984263355	1.601988121	20068	Rps17
P63330	Ppp2ca	1	1	24.3	13	0	35.872	45843000	50499000	51149000	9562900	Ppp2ca	571	0.907800154	1.115742862	1.012871542	19052	Ppp2ca
P67778;Q5SQG5	Phb	2	12	41.2	32	0	64.926	333340000	440100000	375290000	72540000	Phb	220	0.757418768	1.125847483	0.852738014	18673	Phb;Phb1
P68033	Actc1	2	0	70	876	0	323.31	2.395E+10	2.9361E+10	3.0044E+10	4.771E+09	Actc1	2	0.815707912	1.254446764	1.02326215	11464	Actc1
P68040	Gnb2l1	1	11	35.3	24	0	24.817	119830000	71420000	92074000	14405000	Gnb2l1	460	1.677821339	0.76837186	1.289190703	14694	Rack1
P68134	Acta1	1	2	70	11	0	80.414	42810000	16316000	142210000	8601100	Acta1	602	2.623804854	3.321887409	8.71598431	11459	Acta1
P68254-2; P68254	Ywhaq	2	6	40.7	10	0	38.829	46379000	13605000	20178000	5772200	Ywhaq	709	3.408967291	0.435067595	1.483131202	22630	Ywhaq
P68372;Q9D6F9	Tubb4b;Tubb4a	5	3	54.8	119	0	150.36	1029700000	517360000	885260000	130070000	Tubb4b	157	1.990296892	0.859726134	1.711110252	227613	Tubb4a;Tubb4b
P68373;P68369;P05214	Tuba1c;Tuba1a;Tuba3a	3	0	50.1	3	0	4.9207	45012000	16109000	29369000	4346100	Tuba1c	798	2.794214414	0.652470452	1.823142343	22146	Tuba1a;Tuba1c;Tuba3a;Tuba3b
P68510	Ywhah	1	7	47.6	16	0	27.171	103700000	44863000	65924000	13259000	Ywhah	484	2.311481622	0.635718419	1.469451441	22629	Ywhah
P70168	Kpnb1	1	5	5.9	11	0	21.159	24870000	14007000	20524000	1468800	Kpnb1	1176	1.775540801	0.825251307	1.465267366	16211	Kpnb1

P70296;D3Z1V4;D6RHS6	Pebp1	3	7	58.8	24	0	35.51	237500000	306250000	215720000	85816000	Pebp1	202	0.775510204	0.908294737	0.704391837	23980	Pebp1
P70372	Elavl1	1	3	9.5	7	0	8.333	17771000	9055700	12069000	2418200	Elavl1	1011	1.962410416	0.679140172	1.332751748	15568	Elavl1
P70404	Idh3g	1	9	34.4	24	0	43.289	194400000	456170000	305110000	66284000	Idh3g	233	0.426156915	1.569495885	0.668851525	15929	Idh3g
P70670	Naca	2	32	18.7	57	0	79.609	136060000	150820000	148100000	4684700	Naca	774	0.902134995	1.088490372	0.981965257	17938	Naca
P80313;A0A0N4SV00	Cct7	3	8	15.3	16	0	18.574	41675000	30265000	34666000	3922300	Cct7	835	1.377003139	0.831817636	1.145415496	12468	Cct7
P80314	Cct2	5	7	15.9	9	0	21.553	24108000	27260000	20608000	2525400	Cct2	998	0.884372707	0.854819977	0.755979457	12461	Cct2
P80315;G5E839	Cct4	3	7	14.8	11	0	12.927	48731000	29094000	39701000	3934700	Cct4	834	1.674950162	0.81469701	1.364576889	12464	Cct4
P80316	Cct5	2	7	13.9	14	0	28.64	41700000	24204000	37802000	3591700	Cct5	865	1.722855726	0.906522782	1.561807966	12465	Cct5
P82347;A2ACH6	Sgcd	2	7	26	13	0	13.617	55297000	44211000	44531000	8661100	Sgcd	599	1.250752075	0.805305894	1.007238018	24052	Sgcd
P82348	Sgcg	1	4	17.2	9	0	12.59	24109000	25151000	26817000	5156000	Sgcg	745	0.958570236	1.112323199	1.066239911	24053	Sgcg
P82349	Sgcb	1	3	14.1	5	0	4.5042	5136800	5068300	4926600	935140	Sgcb	1312	1.01351538	0.959079583	0.972041908	24051	Sgcb
P82350	Sgca	2	7	22.5	11	0	13.714	30345000	32181000	33805000	5336000	Sgca	732	0.942947702	1.114022079	1.05046456	20391	Sgca
P84078;P61205;Q8BSL7	Arf1;Arf3;Arf2	4	2	23.8	12	0	19.368	114850000	76381000	97400000	30347000	Arf1	313	1.503646195	0.84806269	1.275186237	11840	Arf1;Arf2;Arf3
P84084	Arf5	3	1	17.2	2	0.0049641	1.4961	8727100	6901400	6795800	2225000	Arf5	1029	1.264540528	0.778700828	0.984698757	11844	Arf5
P84104-2; P84104	Srsf3	2	1	18.5	2	0	2.8246	10714000	4747900	8817500	3057600	Srsf3	923	2.256576592	0.822988613	1.857136839	20383	Srsf3
P85094	Isoc2a	1	1	8.3	3	0	3.2559	2102000	16205000	5333700	3512200	Isoc2a	871	0.129713052	2.537440533	0.329139155	664994	Isoc2a
P97315	Csrp1	1	6	46.6	9	0	12.732	35434000	9459600	18784000	5298600	Csrp1	737	3.745824348	0.530112321	1.985707641	13007	Csrp1
P97351	Rps3a	1	10	36.4	15	0	15.146	132710000	97547000	88795000	19137000	Rps3a	398	1.360472388	0.669090498	0.910279147	20091	Rps3a
P97370	Atp1b3	1	3	14.7	4	0	5.0145	8194900	8104600	5756200	1631700	Atp1b3	1138	1.011141821	0.702412476	0.71023863	11933	Atp1b3
P97384;D3Z7U0	Anxa11	2	4	9.5	11	0	13.44	37118000	33863000	26296000	7009100	Anxa11	655	1.096122612	0.708443343	0.776540767	11744	Anxa11
P97443;G5E8R7;P97443-3;P97443-2	Smyd1	6	10	21.4	22	0	37.462	96670000	130340000	134140000	16325000	Smyd1	424	0.741675618	1.387607324	1.029154519	12180	Smyd1
P97450;E9QAD6	Atp5j	2	7	50.9	28	0	47.692	656570000	1230800000	775530000	615750000	Atp5j	40	0.533449789	1.181184032	0.630102372	11957	Atp5j;Atp5pf
P97742	Cpt1a	2	3	7.6	3	0	3.9418	5723400	4266700	3798100	406040	Cpt1a	1481	1.341411395	0.663609044	0.890172733	12894	Cpt1a
P97765	Wbp2	1	1	3.8	1	0.0006124	2.0738	3313900	2485100	3965000	1415500	Wbp2	1189	1.333507706	1.196475452	1.595509235	22378	Wbp2
P97807-2; P97807	Fh	3	18	43.3	65	0	65.75	1331500000	2149200000	1685800000	241880000	Fh	91	0.619532849	1.266090875	0.784384887	14194	Fh
P97855	G3bp1	1	2	4.1	3	0	2.8224	3734100	4195300	2728100	628880	G3bp1	1400	0.890067456	0.730591039	0.650275308	27041	G3bp1
P97927	Lama4	1	5	3.4	11	0	9.1919	11599000	7515700	10440000	355390	Lama4	1495	1.543302686	0.900077593	1.389092167	16775	Lama4
P99024	Tubb5	2	1	44.6	6	0	9.4174	93490000	22156000	46381000	8694200	Tubb5	597	4.219624481	0.496106535	2.093383282	22154	Tubb5
P99026	Psmb4	1	1	3.8	1	0.0006161	2.1216	8082400	5777000	8182900	2001200	Psmb4	1067	1.399065259	1.012434425	1.416461831	19172	Psmb4
P99027;A0A5F8MPY2	Rplp2	2	4	69.6	14	0	54.09	78260000	53050000	42410000	36769000	Rplp2	288	1.475212064	0.541911577	0.799434496	67186	Rplp2;Rplp2-ps1
P99028	Uqcrh	2	7	74.2	25	0	85.042	350000000	1275700000	437000000	369530000	Uqcrh	74	0.274359175	1.248571429	0.342557028	66576	Uqcrh
P99029-2;P99029; G3UZJ4;A0A494BAZ4;H3BJQ7	Prdx5	5	9	58.6	39	0	111.32	464770000	784220000	557830000	211870000	Prdx5	104	0.592652572	1.20022807	0.711318253	54683	Prdx5
Q00519	Xdh	1	3	2.8	6	0	5.2874	8835100	6810400	3031400	408860	Xdh	1479	1.297295313	0.343108737	0.445113356	22436	Xdh
Q00623	Apoa1	3	14	48.9	56	0	32.829	840850000	832860000	574230000	180660000	Apoa1	123	1.009593449	0.682916097	0.689467618	11806	Apoa1
Q00898	Serpina1e	1	2	20.1	5	0	41.418	39854000	22427000	21793000	6048100	Serpina1e	691	1.777054443	0.546820896	0.971730503	20704	Serpina1e
Q00915	Rbp1	1	6	47.4	7	0	49.421	62532000	2182400	6705200	7562900	Rbp1	635	28.65285924	0.107228299	3.072397361	19659	Rbp1
Q00PI9	Hnrnpul2	1	5	8.3	5	0	9.003	25087000	3457200	5747500	989670	Hnrnpul2	1294	7.256450307	0.229102723	1.662472521	68693	Hnrnpul2
Q01149	Col1a2	1	5	4.2	6	0	7.5175	116100000	6307800	10031000	1923100	Col1a2	1079	18.40578332	0.086399655	1.590253337	12843	Col1a2
Q01853	Vcp	1	26	39.1	57	0	84.274	366520000	297740000	337720000	22545000	Vcp	360.5	1.231006919	0.921423115	1.134278229	269523	Vcp
Q02013	Aqp1	1	4	17.1	11	0	20.97	47990000	81971000	48672000	35248000	Aqp1	298	0.585450952	1.014211294	0.593770968	11826	Aqp1
Q02053	Uba1	2	18	21.1	34	0	72.837	128740000	116980000	118310000	6866100	Uba1	660	1.100530005	0.918983999	1.011369465	22201	Uba1
Q02248;E9Q6A9	Ctnnb1	5	5	11.5	10	0	11.694	19019000	13487000	19261000	1326600	Ctnnb1	1208	1.410172759	1.012724118	1.428115964	12387	Ctnnb1
Q02257	Jup	2	20	38	43	0	53.515	189080000	204180000	236450000	14579000	Jup	456	0.926045646	1.250528877	1.158046821	16480	Jup
Q02566	Myh6	5	54	67.1	2552	0	323.31	7.7794E+10	1.1707E+11	1.067E+11	3.396E+09	Myh6	5	0.664508414	1.371571072	0.911420518	17888	Myh6
Q02788;D3Z7D5	Col6a2	2	16	16.6	37	0	38.875	355660000	143410000	140070000	13814000	Col6a2	473	2.480022314	0.393831187	0.976710132	12834	Col6a2
Q02819;A0A1B0GR41;A0A1C7CYU3	Nucb1	5	5	11.1	6	0	12.665	19046000	2639700	8141200	996670	Nucb1	1291	7.21521385	0.427449333	3.084138349	18220	Nucb1
Q03265;D3Z6F5	Atp5a1	3	31	52.6	573	0	282.97	1.5401E+10	2.2721E+10	1.7098E+10	2.061E+09	Atp5a1	10	0.677831081	1.11018765	0.752519695	11946	Atp5a1;Atp5f1a
Q04447	Ckb	1	6	26.8	11	0	33.838	128260000	52141000	67553000	14222000	Ckb	464	2.459868434	0.526687978	1.29558313	12709	Ckb
Q04857	Col6a1	1	15	16.9	40	0	75.159	511690000	209890000	182160000	19824000	Col6a1	389	2.437896041	0.355996795	0.867883177	12833	Col6a1
Q05816	Fabp5	1	4	31.9	9	0	14.744	64701000	30416000	54101000	21610000	Fabp5	369	2.127202788	0.836169456	1.778701999	16592	Fabp5
Q05DV1;P37040	Por	2	3	4.8	3	0	3.6432	6068000	3298200	4514200	440980	Por	1468	1.839791401	0.743935399	1.36868595	18984	Por
Q06138	Cab39	1	2	6.5	2	0.0005889	1.8245	6990000	5077700	7940200	966410	Cab39	1300	1.376607519	1.135937053	1.563739488	12283	Cab39
Q06185;Q8BTB6	Atp5i;Atp5k	2	6	63.4	70	0	20.841	369820000	584800000	520840000	607470000	Atp5i	42	0.632387141	1.408360824	0.890629275	11958	Atp5i;Atp5me
Q06890;E9PUU2;E9PXG5;E9Q8Y5	Clu	6	11	27	17	0	35.443	132350000	28096000	65700000	11853000	Clu	509	4.710634966	0.496411031	2.338411162	12759	Clu
Q07417	Acads	1	14	40.3	52	0	62.381	362760000	761410000	475630000	92695000	Acads	193	0.476431883	1.311142353	0.62467002	11409	Acads
Q08857	Cd36	2	11	23.9	30	0	49.072	438800000	570160000	487440000	105440000	Cd36	176	0.769608531	1.110847767	0.854917918	12491	Cd36
Q11011;F6QYF8;E9Q039;E9Q6F4	Npepps	7	11	12.9	20	0	22.532	37989000	49013000	40386000	2893400	Npepps	946	0.775080081	1.063097212	0.823985473	19155	Npepps
Q11136	Pepd	2	4	8.9	5	0	4.0966	3960700	1830200	2800200	346930	Pepd	1500	2.164080428	0.706996238	1.529996722	18624	Pepd
Q149B8	Perm1	1	4	6.2	6	0	11.42	9944300	13322000	11186000	1486000	Perm1	1170	0.746456988	1.124865501	0.839663714	74183	Perm1
Q14BI5	Myom2	1	64	48.1	193	0	323.31	1472600000	2408400000	1981300000	80746000	Myom2	208	0.611443282	1.345443433	0.822662348	17930	Myom2
Q14C51;A0A0U1RQ61	Ptcd3	2	2	3.2	2	0	3.1464	5136300	5715100	5325000	443210	Ptcd3	1467	0.898724432	1.036738508	0.931742227	69956	Ptcd3
Q1XH17	Trim72	1	23	64.8	63	0	138.58	401810000	482800000	631440000	56287000	Trim72	252	0.832249379	1.571489012	1.307870754	434246	Trim72
Q2TPA8;B1AX78	Hsdl2	3	13	29.4	26	0	54.055	83302000	155720000	106670000	13066000	Hsdl2	488	0.534947341	1.280521476	0.685011559	72479	Hsdl2
Q3TCD4;Q9WUR2-2;Q9WUR2;E9PYC6;E9Q858;E9PUY9;E9PYA9;E9PVM6;E9Q7A8	Eci2	9	4	17.2	13	0	16.158	49366000	105750000	56865000	14432000	Eci2	459	0.466817967	1.15190617	0.537730496	23986	Eci2
Q3TF41;Q8BSH9;P28656	Nap1l1	3	2	12.2	3	0	8.6544	7420700	4618400	4600800	1230300	Nap1l1	1241	1.606768578	0.619995418	0.996189156	53605	Nap1l1
Q3THW5;P0C0S6; Q8R029;Q3UA95	H2afv;H2afz	4	2	31.2	4	0	4.7453	61028000	24564000	20508000	22398000	H2afv	364	2.484448787	0.336042472	0.834880313	77605	H2az1;H2az2
Q3TL44	Nlrx1	3	5	7	6	0	9.0206	8585500	11119000	8779500	808470	Nlrx1	1345	0.772146776	1.022596238	0.789594388	270151	Nlrx1
764;A2AC13;Q05144	Rac1;Rac3;Rac2	15	4	24.6	14	0	9.0159	102180000	50851000	81749000	28681000	Rac1	322	2.009400012	0.800048933	1.607618336	19353	Rac1;Rac3;Rac2
Q3TML0;Q922R8	Pdia6	2	5	14.6	10	0	26.175	44125000	12398000	29536000	5006400	Pdia6	755	3.559041781	0.669371105	2.382319729	71853	Pdia6
Q3TRE0;Q62147	Sspn	2	1	4.6	2	0.0049451	1.475	4669100	4358600	5354000	1866400	Sspn	1086	1.071238471	1.146687799	1.228376084	16651	Sspn
Q3TWV4;P84091	Ap2m1	3	5	13.2	5	0	6.8994	9496400	3871700	3847300	696690	Ap2m1	1381	2.452772684	0.405132471	0.993697859	11773	Ap2m1
Q3TXN1; Q8VDQ1-2;Q8VDQ1;D6RGL6	Ptgr2	4	2	8.9	5	0	8.1689	16651000	30260000	23179000	8058600	Ptgr2	621	0.550264375	1.392048526	0.765994712	77219	Ptgr2
Q3TXS7	Psmd1	2	4	5.9	5	0	7.5125	15454000	10057000	18272000	940640	Psmd1	1309	1.536641145	1.182347612	1.816843989	70247	Psmd1
Q3TYS2		1	1	5.3	2	0.0006072	2.0226	2773800	997630	1734600	507200	Q3TYS2	1444	2.780389523	0.625351503	1.738720768	217370	[illegible]

Q3U0B3;F2Z445	Dhrs11	2	2	8.1	2	0	3.6634	3360700	4691400	5131200	912550	Dhrs11	1321	0.716353327	1.526824769	1.093746003	192970	Dhrs11
Q3U125;Q9CYH2;D3Z0A8;D3YYG8;D3Z398;D3Z252	Fam213a	6	2	10.5	3	0	2.3161	4658200	6678600	7364500	1569000	Fam213a	1147	0.697481508	1.580975484	1.102701165	70564	Prxl2a
Q3U1J4;A0A494BAF6;A0A494B987	Ddb1	3	4	3.3	4	0	4.4881	8304600	7478400	9263200	458330	Ddb1	1463	1.110478177	1.115430003	1.238660676	13194	Ddb1
Q3U2G2;Q61316	Hspa4	2	18	28.5	36	0	67.204	109520000	88536000	109440000	6373500	Hspa4	680	1.237010933	0.99926954	1.236107346	15525	Hspa4
Q3U367;Q9JLJ2	Aldh9a1	2	3	6.2	3	0	4.4569	5158200	2487600	18758000	915570	Aldh9a1	1320	2.073564882	3.636539878	7.540601383	56752	Aldh9a1
Q3U3G8;Q9EPB5	Serhl	3	4	12.1	8	0	5.4415	16131000	23719000	24025000	4442000	Serhl	792	0.680087693	1.489368297	1.01290105	68607	Serhl
Q3U3J1;P50136	Bckdha	3	10	24.4	19	0	33.197	43573000	91448000	57246000	8506800	Bckdha	608	0.476478436	1.31379524	0.625995101	12039	Bckdha
Q3U4F0;Q91V61-2;Q91V61;A0A494BB84	Sfxn3	4	2	10	3	0	8.4182	2453400	1210000	3720800	411380	Sfxn3	1477	2.027603306	1.516589223	3.075041322	94280	Sfxn3
Q3U4W8;P56399;D3YYA5	Usp5	4	4	5.6	6	0	6.6241	19748000	13589000	18748000	1245700	Usp5	1230	1.453234234	0.949361961	1.379645301	22225	Usp5
Q3U7R1;Q3U7R1-2;A0A1W2P784	Esyt1	3	4	4.2	6	0	8.615	9832500	8478300	6447900	520920	Esyt1	1438	1.159725417	0.655774218	0.760518028	23943	Esyt1
0A1D5RL90;O88746-2	Tom1	4	2	4.7	4	0	3.3115	4820300	3642700	5080200	594520	Tom1	1413	1.32327669	1.053917806	1.394624866	21968	Tom1
Q3UE92;Q6P1B1;A0A494BBG8;S4R1I3	Xpnpep1	4	3	5.9	4	0	5.0183	5991900	5253100	5400400	480180	Xpnpep1	1452	1.140640765	0.901283399	1.028040586	170750	Xpnpep1
Q3UER8; Q8VCM7	Fgg	2	8	19	22	0	18.281	104240000	61429000	94034000	10221000	Fgg	547	1.696918394	0.902091328	1.530775367	99571	Fgg
Q3UGC7;Q66JS6	Eif3j1;Eif3j2	2	3	13	5	0	6.4449	12387000	4879500	10372000	2002900	Eif3j1	1066	2.538579773	0.837329458	2.125627626	78655	Eif3j1;Eif3j2
Q3UGR5;Q3UGR5-2	Hdhd2	2	4	13.9	8	0	6.3498	20828000	27214000	22198000	7292000	Hdhd2	643	0.765341368	1.065776839	0.815683104	76987	Hdhd2
Q3UHC2; A6QRF1; F2Z3W1;F2Z444;A0A0R4J1C3;Q3UHC2-2;Q6A000	Lrrk1;4933427D14Rik;Kiaa0753	7	2	0.8	3	0.0017452	1.7481	1280000000	1318500000	1493800000	45356000	Lrrk1	269	0.970800152	1.16703125	1.132954115	233328	4933427D14Rik;Kiaa0753;Lrrk1
Q3UHZ5	Lmod2	1	5	8.7	7	0	7.8654	10831000	9777400	20338000	1415400	Lmod2	1190	1.107758709	1.877758286	2.080103095	93677	Lmod2
Q3UIJ9;E9Q565	Myzap	2	4	10.1	6	0	9.0481	4947600	5577900	6640300	636270	Myzap	1397	0.887000484	1.342125475	1.190465946	102371	Myzap
Q3UIZ8;D3Z630;Q3UIZ8-2	Mylk3	4	9	13	12	0	19.497	25947000	35536000	24137000	2266700	Mylk3	1026	0.730160964	0.930242417	0.6792267	213435	Mylk3
Q3UK98; Q9DBY5	Cbx6	2	1	4.8	1	0	2.5547	27376000	15901000	26634000	4245900	Cbx6	809	1.721652726	0.972895967	1.674988994	494448	Cbx6
Q3ULD5;Q6PD20	Mccc2	2	15	30.9	29	0	59.211	84215000	171380000	92620000	15630000	Mccc2	439	0.491393395	1.099804073	0.540436457	78038	Mccc2
Q3ULJ0-2;Q3ULJ0	Gpd11	3	7	25.6	11	0	12.981	44163000	60299000	56498000	8357300	Gpd11	615	0.732400206	1.279306207	0.936964129	333433	Gpd11
Q3UM45	Ppp1r7	1	2	4.7	2	0	2.665	6227500	6372000	7004400	875370	Ppp1r7	1331	0.977322662	1.124753111	1.099246704	66385	Ppp1r7
Q3UPL0-2;Q3UPL0;S4R2A9	Sec31a	5	5	5	6	0	8.9449	23253000	4355700	1949200	711450	Sec31a	1378	5.338521937	0.083825743	0.447505567	69162	Sec31a
Q3UTJ2-2;A0A0R4J1T1;Z4YJR7;B9EKP8	Sorbs2	4	2	24.5	49	0	51.08	194330000	136500000	294170000	8581300	Sorbs2	603	1.423663004	1.513765245	2.155091575	234214	Sorbs2
Q3UV17	Krt76	1	1	5.2	3	0.0005928	1.8765	44418000	17935000	24636000	2506900	Krt76	1003	2.47660998	0.554640011	1.373626986	77055	Krt76
Q3UVT7;A0A0R4J0J0;Q9JHU2	Palmd	4	3	6.5	2	0	3.3322	6713800	9015800	8072200	1025100	Palmd	1287	0.744670467	1.20232953	0.895339293	114301	Palmd
Q3V117;Q91V92;Q3TS02	Acly	3	2	2.2	2	0	3.6492	25347000	34449000	26485000	1555400	Acly	1153.5	0.735783332	1.044896832	0.768817673	104112	Acly
Q3V132	Slc25a31	1	2	13.8	1	0.0006207	2.1662	2757900	14924000	4412000	1140600	Slc25a31	1261	0.184796301	1.599767939	0.295631198	73333	Slc25a31
Q4VAE3	Tmem65	1	2	9	5	0	4.4643	12666000	20085000	11616000	4071500	Tmem65	825	0.630619866	0.9171009	0.578342046	74868	Tmem65
Q4VWZ5;P31786;D3Z563;M0QWU8	Dbi	4	2	25.2	11	0	7.3939	28143000	57556000	30675000	20091000	Dbi	384	0.488967267	1.089969086	0.532959205	13167	Dbi
Q505D7;A0A0U1RPB1	Opa3	2	2	10.6	5	0	5.5206	15667000	30970000	18382000	9742400	Opa3	565	0.505876655	1.173294185	0.593542138	403187	Opa3
Q543K9;P23492	Pnp	4	5	19.7	7	0	22.671	69313000	14373000	19877000	6626600	Pnp	672	4.822444862	0.286771601	1.382940235	18950	Pnp
Q5EBG6	Hspb6	1	5	37	24	0	25.384	117860000	128040000	220900000	70618000	Hspb6	225	0.920493596	1.874257594	1.725242112	243912	Hspb6
Q5EBP8;P49312;P49312-2	Hnrnpa1	3	4	19.3	8	0	13.717	9554500	4606200	5937500	1236600	Hnrnpa1	1236	2.074269463	0.621434926	1.28902349	15382	Hnrnpa1
Q5M8N4;A0A2I3BQS7	Sdr39u1	4	5	20.1	6	0	12.143	9631600	19764000	14642000	4321700	Sdr39u1	801	0.4873305	1.520204327	0.740841935	654795	Sdr39u1
Q5ND35; Q61247;E9PXE0	Serpinf2	3	2	7.9	3	0	2.828	5156600	5571100	5083200	1693400	Serpinf2	1125	0.925598176	0.985765815	0.91242304	18816	Serpinf2
Q5RKN9; P47753;A0A0G2JE27	Capza1	3	3	17.8	3	0	7.1661	14710000	3257500	4624900	1736700	Capza1	1113	4.515732924	0.314405167	1.419769762	12340	Capza1
Q5RL20;Q99N89	Mrpl43	2	2	11.3	2	0	3.0642	4429700	5372800	4835100	1518300	Mrpl43	1162	0.824467689	1.091518613	0.899921828	94067	Mrpl43
Q5SS83;Q60634-3;Q60634-2;Q60634	Flot2	4	6	16.6	7	0	16.754	17553000	14365000	14141000	1694900	Flot2	1124	1.221928298	0.805617273	0.984406544	14252	Flot2
Q5SV64;Q3UH59;Q61879	Myh10	4	17	18.4	22	0	53.615	106870000	7957400	28724000	1309100	Myh10	1213	13.43026617	0.268775147	3.609721768	77579	Myh10
Q5SVG5;Q5SVG4;O35643	Ap1b1	3	3	11.6	3	0	4.3771	3119500	2215300	2965200	191340	Ap1b1	1546	1.408161423	0.950536945	1.338509457	11764	Ap1b1
Q5SVW9;Q8R1V4	Tmed4	2	1	6.5	1	0	3.3572	1587900	1597900	1729000	480470	Tmed4	1451	0.993741786	1.0888595	1.082045184	103694	Tmed4
Q5SX39	Myh4	1	0	16.6	20	0	6.2445	2692100000	675400000	3713600000	75084000	Myh4	215	3.985934261	1.379443557	5.498371336	17884	Myh4
Q5SXR6; Q68FD5	Cltc	3	36	25.4	66	0	108.76	374880000	154150000	233790000	8625000	Cltc	601	2.431916964	0.623639565	1.516639637	67300	Cltc
Q5U458;E9Q8B3	Dnajc11	3	6	14.5	8	0	9.0214	8218800	11905000	14665000	1605600	Dnajc11	1141	0.690365393	1.784323746	1.231835363	230935	Dnajc11
Q5XJY5	Arcn1	1	3	6.1	2	0	4.5669	14839000	5853700	9818900	1146700	Arcn1	1260	2.534977877	0.661695532	1.677383535	213827	Arcn1
Q60597;Z4YJV4;Q60597-2;Q60597-4;Q60597-3	Ogdh	7	38	48	182	0	271.25	2057800000	3240700000	2451600000	196560000	Ogdh	112	0.634986268	1.191369424	0.756503225	18293	Ogdh
Q60598;Q921L6	Cttn	2	4	7.5	4	0	5.2171	10065000	5605000	8770200	878490	Cttn	1330	1.795718109	0.871356185	1.56471008	13043	Cttn
Q60668-3;Q60668;F6ZV59;G5E8G0;G3X9W0;Q60668-4;Q60668-2;E9Q5B6;F6SHF3	Hnrnpd	13	6	24.8	8	0	8.925	84884000	20379000	17354000	9104200	Hnrnpd	586	4.165268168	0.204443711	0.851562883	11991	Hnrnpd
Q60675	Lama2	4	43	15.7	71	0	99.246	217990000	269820000	164450000	4645500	Lama2	776	0.807908976	0.754392403	0.609480394	16773	Lama2
Q60676;F7BX26	Ppp5c	2	3	6.8	3	0	2.6335	5614500	5106100	6100900	701690	Ppp5c	1379	1.099567184	1.086632826	1.194825797	19060	Ppp5c
Q60692	Psmb6	1	4	16.8	8	0	9.7366	33909000	30075000	37936000	9852500	Psmb6	559	1.127481297	1.118759032	1.261379884	19175	Psmb6
Q60714	Slc27a1	1	2	3.3	4	0	3.0469	2979600	6763700	3345800	518810	Slc27a1	1440	0.440528113	1.122902403	0.494670077	26457	Slc27a1
Q60766-2;Q60766	Irgm1	2	3	7.6	4	0	3.1993	6257500	5517800	6380100	1111600	Irgm1	1269	1.134057052	1.019592489	1.156276052	15944	Irgm1
Q60864	Stip1	1	17	33.5	31	0	48.991	135130000	75720000	114410000	9890900	Stip1	556	1.784601162	0.846666173	1.510961437	20867	Stip1
Q60865	Caprin1	1	1	1.6	2	0.0044444	1.5319	2895300	1482600	1805800	310550	Caprin1	1511	1.952853096	0.62370048	1.217995413	53872	Caprin1
Q60930;G3UX26;D3YZT5;A0A286YCR8	Vdac2	5	12	44.7	59	0	58.272	662690000	844760000	799230000	161290000	Vdac2	129	0.784471329	1.206039023	0.946103035	22334	Vdac2
Q60932-2;Q60932;F2Z471	Vdac1	3	16	71	97	0	118.57	1226700000	2680300000	1870100000	428280000	Vdac1	65	0.457672649	1.524496617	0.697720404	22333	Vdac1
Q60936;F7B1B6;Q60936-2	Adck3	5	9	16.1	17	0	28.376	68272000	128590000	63452000	11941000	Adck3	505	0.530927755	0.929400047	0.49344428	67426	Coq8a
Q60972;A2AFI9;A2AFJ1;Q60973	Rbbp4;Rbbp7	4	2	5.2	3	0	4.5393	9507300	6539400	6554600	1662700	Rbbp4	1131	1.453848977	0.689428124	1.002324372	19646	Rbbp7;Rbbp4
Q60994;E9PWU4	Adipoq	2	2	8.9	3	0	6.491	8052400	7398500	6127900	2191300	Adipoq	1032	1.08838278	0.761002931	0.828262486	11450	Adipoq
Q61001	Lama5	1	2	0.5	2	0.0033898	1.6107	19225000	22537000	22930000	443450	Lama5	1466	0.853041665	1.192717815	1.017437991	16776	Lama5

Q61029-3;Q61029;Q61029-2;Q61033-2;Q61029-4;Q61033	Tmpo	6	4	15	6	0	13.133	11038000	3283700	4071600	899460	Tmpo	1323	3.361452021	0.368871172	1.239942748	21917	Tmpo
Q61035	Hars	4	5	10.4	7	0	8.9296	13590000	10735000	15651000	1498200	Hars	1166	1.265952492	1.151655629	1.457941313	15115	Hars1
Q61102	Abcb7	1	1	1.6	1	0.0080043	1.3362	1453300	1789100	1131800	149620	Abcb7	1556	0.812307864	0.77877933	0.632608574	11306	Abcb7
Q61166	Mapre1	1	2	9.7	2	0	2.4173	7031000	3162400	4915000	888350	Mapre1	1326	2.223311409	0.699047077	1.554199342	13589	Mapre1
Q61171;D3Z4A4	Prdx2	2	8	43.9	27	0	26.462	572350000	637580000	617330000	155270000	Prdx2	137	0.89769127	1.078588276	0.96823928	21672	Prdx2
Q61233	Lcp1	5	12	26.5	21	0	52.367	197240000	21887000	57452000	7410200	Lcp1	638	9.01174213	0.291279659	2.624937177	18826	Lcp1
Q61292	Lamb2	2	16	10.3	27	0	35.244	75325000	58215000	42675000	2117000	Lamb2	1045	1.293910504	0.566544972	0.73305849	16779	Lamb2
Q61335	Bcap31	1	3	11.8	5	0	8.4924	45135000	11169000	36055000	6195900	Bcap31	685	4.04109589	0.798825745	3.228131435	27061	Bcap31
Q61425	Hadh	1	14	36.6	35	0	37.867	1281000000	2508000000	1545200000	469920000	Hadh	62	0.51076555	1.206245121	0.616108453	15107	Hadh
Q61553;A0A0G2JDU7	Fscn1	3	7	18.1	7	0	9.569	24648000	8659800	24566000	2126300	Fscn1	1043	2.84625511	0.996673158	2.836786069	14086	Fscn1
Q61554	Fbn1	2	58	23.6	119	0	144.93	1161500000	215030000	325230000	11885000	Fbn1	508	5.401571874	0.28000861	1.51248663	14118	Fbn1
Q61598;Q61598-2;A0A1Y7VL99	Gdi2	4	12	36.6	26	0	28.672	177220000	124720000	144240000	16050000	Gdi2	432	1.420942912	0.813903623	1.156510584	14569	Gdi2
Q61599;D3YWL7	Arhgdib	2	3	25.5	4	0	16.001	18331000	1666300	3189100	2035800	Arhgdib	1059	11.00102022	0.173973051	1.913881054	11857	Arhgdib
Q61646	Hp	1	3	9.8	6	0	5.4617	14837000	14407000	13247000	2308800	Hp	1021	1.029846602	0.892835479	0.919483584	15439	Hp
Q61696;P17879	Hspa1a;Hspa1b	2	3	15.4	7	0	7.9509	6948900	4488400	8531800	548520	Hspa1a	1425	1.548190892	1.227791449	1.900855539	193740	Hspa1a;Hspa1b
Q61768	Kif5b	5	15	17.9	19	0	45.675	36691000	24449000	33264000	1590200	Kif5b	1144	1.500715776	0.906598348	1.360546444	16573	Kif5b
Q61838	A2m	1	21	17.4	47	0	47.223	213920000	231630000	214720000	11442000	A2m	517	0.923541856	1.003739716	0.92699564		Pzp
Q62000	Ogn	1	8	27.2	13	0	12.488	357910000	15415000	40435000	29319000	Ogn	319	23.21829387	0.112975329	2.623094389	18295	Ogn
Q62009;Q62009-3;Q62009-4;Q62009-2;Q62009-5	Postn	7	20	30.5	38	0	154.9	794430000	2428500	33858000	19385000	Postn	393	327.1278567	0.042619236	13.94193947	50706	Postn
Q62093	Srsf2	1	4	21.7	4	0	10.886	19837000	8888800	12883000	4677700	Srsf2	775	2.231684817	0.64944296	1.449351994	20382	Srsf2
Q62095	Ddx3y	1	1	10.8	5	0.0011716	1.7987	1945300	3003000	2697800	255120	Ddx3y	1528	0.647785548	1.386829795	0.898368298	26900	Ddx3y
Q62165	Dag1	1	1	1.8	1	0.002867	1.6779	2484000	926860	1436300	130760	Dag1	1558	2.680016399	0.578220612	1.549640722	13138	Dag1
Q62167;P16381	Ddx3x;D1Pas1	2	2	13.1	9	0	12.427	22068000	11444000	19503000	1361600	Ddx3x	1203	1.928346732	0.883768352	1.704211814	13205	D1Pas1;Ddx3x
Q62261;A0A0A0MQG2;Q62261-2	Sptbn1	5	67	35.5	156	0	280.11	676240000	481990000	542120000	12716000	Sptbn1	495	1.40301666	0.801668047	1.124753626	20742	Sptbn1
Q62351;Q8C872	Tfrc	2	6	8.1	6	0	7.484	9763900	3353000	18782000	714140	Tfrc	1377	2.911989263	1.923616588	5.60155085	22042	Tfrc
Q62426	Cstb	1	2	18.4	2	0.0006289	2.2762	14058000	5797500	5883900	4255500	Cstb	808	2.424838292	0.418544601	1.014902975	13014	Cstb
Q62446;A0A1Y7VLK0;A0A1Y7VJ86;A0A1Y7VP01	Fkbp3	5	4	21.4	13	0	17.014	35626000	37292000	32681000	11804000	Fkbp3	511	0.955325539	0.917335654	0.876354178	30795	Fkbp3
Q62465	Vat1	1	4	12.3	6	0	13.629	25510000	4551700	8370700	2675000	Vat1	970	5.604499418	0.328134065	1.839027177	26949	Vat1
Q63918	Sdpr	1	12	32.1	19	0	28.05	78334000	78213000	64191000	10709000	Sdpr	536	1.001547057	0.8194526	0.820720341	20324	Cavin2
Q64010;Q8JZR2;F7D232;Q5ND50;Q64010-2	Crk	5	3	11.5	5	0	4.6557	7602100	5790300	6028600	1173300	Crk	1256	1.312902613	0.793017719	1.041155035	12928	Crk
Q64314;Q64314-2	Cd34	2	2	5.2	4	0	8.1363	19959000	4573500	7984900	2691100	Cd34	966	4.364053788	0.400065134	1.745905761	12490	Cd34
Q64433;Q9JI95	Hspe1;Cpn10-rs1	2	8	74.5	25	0	19.593	558520000	1022900000	850300000	341880000	Hspe1	77	0.546016228	1.522416386	0.831264053	15528	Hspe1;Hspe1-rs1
Q64669	Nqo1	1	8	28.1	14	0	13.612	30547000	37808000	57974000	9152300	Nqo1	585	0.807950698	1.897862311	1.533379179	18104	Nqo1
Q64727	Vcl	1	50	47.4	111	0	171.33	1003400000	792200000	977060000	38672000	Vcl	281	1.266599344	0.973749253	1.233350164	22330	Vcl
Q64737;Q64737-2;A0A338P6X4;D6RCGI	Gart	5	5	4.8	4	0	4.4526	11797000	8827100	10696000	723200	Gart	1372	1.336452516	0.906671188	1.211722989	14450	Gart
Q65CL1	Ctnna3	1	5	6.6	7	0	10.922	12754000	18418000	15113000	1130700	Ctnna3	1264	0.692474753	1.184961581	0.820555978	216033	Ctnna3
Q69ZP3-2;A0A0R4J1J1		2	1	7.7	2	0	2.3795	3330700	4136300	3961100	2897300	Q69ZP3-2	944	0.805236564	1.189269523	0.957643304	56695	Pnkd
Q6GT24;D3Z0Y2;O08709	Prdx6	5	10	50.4	31	0	69.25	258900000	325320000	247430000	63338000	Prdx6	235	0.795831796	0.95569718	0.760574204	11758	Prdx6
Q6IRU2;A0A571BEU1	Tpm4	2	13	57.3	20	0	78.704	197940000	64466000	93997000	28627000	Tpm4	323	3.070455744	0.474876225	1.458086433	326618	Tpm4
Q6IRU5-2;Q6IRU5;F7BHJ0;Q6IRU5-3	Cltb	4	5	20.9	8	0	12.895	41194000	19775000	39541000	11529000	Cltb	515	2.083135272	0.959872797	1.99954488	74325	Cltb
Q6P069-2;Q6P069	Sri	2	4	24	4	0	7.8175	14399000	4712600	6286500	1956200	Sri	1076	3.05542588	0.436592819	1.333976998	109552	Sri
Q6P3A8-2;Q6P3A8	Bckdhb	2	4	14.3	6	0	7.6017	11811000	31775000	12599000	6008300	Bckdhb	694	0.371707317	1.066717467	0.396506688	12040	Bckdhb
Q6P3F7;Q9ES82	Popdc2	2	3	7.5	5	0	4.1952	12622000	14906000	14312000	4069900	Popdc2	826	0.846773111	1.133893202	0.960150275	64082	Popdc2
Q6P5E4	Uggt1	2	3	2.4	4	0	5.0174	6627100	2439400	4361000	173640	Uggt1	1550	2.716692629	0.65805556	1.787734689	320011	Uggt1
Q6P5F9;F6YA11	Xpo1	2	2	1.9	2	0.0006053	2.0108	6330900	4036300	4737500	292080	Xpo1	1520	1.568490945	0.748313826	1.17372346	103573	Xpo1
Q6P8J7	Ckmt2	2	21	49.9	240	0	323.31	2903700000	5154800000	3735300000	663060000	Ckmt2	36	0.563300225	1.286393222	0.724625592	76722	Ckmt2
Q6P8V7;A0A1B0GRP7;Q9Z2Y8;A0A1B0GQY5;A0A1B0GRR5;Q80ZV3	Prosc	8	4	27.5	4	0	8.4782	19514000	32696000	28555000	9240200	Prosc	584	0.596831417	1.463308394	0.873348422	114863	Plpbp
Q6P8X1;A0A1W2P701	Snx6	2	1	5.2	1	0	2.7327	4214000	1532500	1993000	341890	Snx6	1504	2.749755302	0.472947318	1.300489396	72183	Snx6
Q6P9R2	Oxsr1	2	3	5.3	3	0	2.4953	10116000	14743000	13236000	1753100	Oxsr1	1111	0.686156142	1.308422301	0.897781998	108737	Oxsr1
Q6PB66	Lrpprc	2	32	23.7	52	0	93.942	268590000	405370000	295010000	12673000	Lrpprc	496	0.662579865	1.098365539	0.727754891	72416	Lrpprc
Q6PBC0;A2AMQ5;Q99L43	Cds2	3	2	24	4	0	8.8922	5783300	3497900	5917300	2600700	Cds2	983	1.653363447	1.023170162	1.691672146	110911	Cds2
Q6PD03;A0A0A6YVV9	Ppp2r5a	2	2	5.3	4	0	5.1462	5583800	6627000	4672600	774970	Ppp2r5a	1357	0.842583371	0.836813639	0.705085257	226849	Ppp2r5a
Q6PDI5-2;Q6PDI5;A2ALV7	Ecm29;AI314180	6	5	3	5	0	4.4994	2946300	3418900	4136400	110980	Ecm29	1562	0.861768405	1.403930353	1.209862821	230249	Ecpas
Q6PHN9	Rab35	1	1	4.5	2	0.0048833	1.4258	4721600	3413200	4249400	1094600	Rab35	1271	1.383335287	0.899991528	1.244990039	77407	Rab35
Q6W8Q3	Pcp4l1	1	1	20.6	2	0	2.3462	1302200	2788000	1292200	1633900	Pcp4l1	1136	0.467073171	0.992320688	0.46348637	66425	Pcp4l1
Q6XLQ8;O35887;G3UWV3;G3V004	Calu	8	5	16.2	7	0	24.662	34884000	3892300	18277000	3230000	Calu	899	8.962310202	0.523936475	4.695681217	12321	Calu
Q6XPS7	Tha1	1	5	12.5	7	0	7.6319	7809300	12146000	12153000	1735800	Tha1	1114	0.642952412	1.556221428	1.000576321	71776	Tha1
Q6ZQ38	Cand1	1	8	7	10	0	16.994	25164000	12823000	15915000	861980	Cand1	1335	1.962411292	0.632451121	1.241129221	71902	Cand1
Q6ZQ73	Cand2	1	8	7.3	16	0	18.898	29328000	29824000	38992000	1762100	Cand2	1108	0.983369099	1.329514457	1.307403433	67088	Cand2
Q6ZWN5;F7CJS8;D3YWH9;Q9CXW7	Rps9	5	7	36.1	16	0	14.134	99536000	64552000	86034000	24340000	Rps9	352	1.541950675	0.864350587	1.332785971	76846	Rps9
Q6ZWQ9;Q3THE2;D3YV37	Myl12a;Myl12b	4	2	40.1	13	0	28.354	99653000	34955000	55222000	20145000	Myl12a	382	2.850894007	0.554142876	1.579802603	67268	Myl12a;Myl12b
Q6ZWV7	Rpl35	1	2	15.4	6	0	5.899	19207000	14988000	19753000	18939000	Rpl35	401	1.28149186	1.028427136	1.317921003	66489	Rpl35
Q6ZWX6	Eif2s1	1	6	19	11	0	10.15	28859000	24429000	25936000	3832700	Eif2s1	843	1.181341848	0.898714439	1.061688976	13665	Eif2s1
Q6ZWY3;D6RH49;D3YYB0	Rps27l	3	1	38.1	5	0	6.6535	33231000	19351000	29966000	21233000	Rps27l	371	1.717275593	0.901748367	1.548550463	67941	Rps27l
Q6ZWY9;Q64525;Q64478;P10854	Hist1h2bc;Hist2h2bb;Hist1h2bh; Hist1h2bm	4	1	53.2	70	0	55.169	5194600000	2781100000	2745600000	1.853E+09	Hist1h2bc	11	1.867822085	0.528548878	0.987235267	319179	H2bc14;H2bc4; H2bc6; H2bc8;H2bc9; Hist2h2bb

Q6ZWZ6;F7AEH4;A0A1W2P7A1;P63323	Rps12	4	3	25.8	11	0	7.6281	50768000	40618000	35328000	20096000	Rps12	383	1.249889212	0.695871415	0.869762174	20042	Rps12
Q6ZWZ7;Q9CPR4	Rpl17	2	4	16.3	9	0	11.947	6366500	8955500	10199000	3266400	Rpl17	893	0.710903914	1.601979109	1.138853219	319195	Rpl17
Q71RI9-2;Q71RI9	Ccbl2	4	3	8.3	5	0	9.1268	10031000	14481000	9813500	2026200	Ccbl2	1061	0.69270078	0.978317217	0.677681099	229905	Kyat3
Q76MZ3	Ppp2r1a	11	16	31.4	24	0	32.03	83213000	69310000	86896000	8472200	Ppp2r1a	609	1.200591545	1.044259911	1.253729621	51792	Ppp2r1a
Q78IK2	Usmg5	1	3	44.8	17	0	25.42	127480000	260100000	195480000	230710000	Usmg5	95	0.490119185	1.533417007	0.751557093	66477	Atp5mk
Q78IK4;B1AV14	Apool	2	7	33.2	10	0	20.11	41288000	50340000	48387000	9368600	Apool	577	0.820182757	1.171938578	0.961203814	68117	Apool
Q78J03	Msrb2	1	4	22.9	4	0	5.2879	10830000	20840000	10955000	3887800	Msrb2	838	0.519673704	1.011542013	0.525671785	76467	Msrb2
Q78PY7	Snd1	2	6	7.1	9	0	9.0634	27607000	5930400	20473000	976340	Snd1	1298	4.655166599	0.741587279	3.45221233	56463	Snd1
Q791V5;A2AFW6;Q9D050	Mtch2	3	8	32	26	0	33.423	110670000	154730000	135680000	32596000	Mtch2	307	0.715245912	1.225987169	0.876882311	56428	Mtch2
Q792Z1	Try10	1	1	4.1	2	0	2.4118	7218600	7048300	7156900	2606000	Try10	981	1.024161855	0.991452636	1.015407971	436522	Try10
Q7M6Y3-2;Q7M6Y3-3;Q7M6Y3-4;Q7M6Y3-6;Q7M6Y3-5;Q7M6Y3;A0A1L1SUR7;A0A140LHQ8	Picalm	9	3	5.7	4	0	5.5666	15209000	4934800	11215000	1217000	Picalm	1245	3.081989138	0.737392333	2.272635163	233489	Picalm
Q7M739; F6ZDS4	Tpr	2	3	1.3	3	0	4.51	5291400	7197100	4315600	167250	Tpr	1551	0.735212794	0.815587557	0.599630407	108989	Tpr
Q7TMB8;A0A0U1RQ05;A0A0R4J119;Q7TMB8-2;Q5SQX6;F6QD74	Cyfip1;Cyfip2	6	4	3.7	3	0	6.1033	9575300	2874000	3574400	246180	Cyfip1	1530	3.331697982	0.373293787	1.243702157	20430	Cyfip1;Cyfip2
Q7TMD7	Dsg4	1	2	2	7	0	2.316	210840000	233670000	274470000	21701000	Dsg4	368	0.902298113	1.301792829	1.174605212	16769	Dsg4
Q7TMG8;O55126;A0A0G2JEV1	Gbas	3	10	29.5	50	0	39.669	610980000	1142700000	769600000	187210000	Gbas	118	0.534681019	1.259615699	0.673492605	14467	Nipsnap2
Q7TMM9	Tubb2a	1	1	40.4	3	0	2.5331	19697000	6089900	20280000	2181800	Tubb2a	1033	3.234371665	1.029598416	3.330103943	22151	Tubb2a
Q7TNQ1; Q9QZE5;Q9QXK3-2;Q9QXK3-3;Q9QXK3-4;Q9QXK3	Copg1;Copg2	6	2	14.9	2	0.0049834	1.5149	6696000	3737600	4447500	1784300	Copg1	1104	1.791523973	0.664202509	1.189934717	54161	Copg1;Copg2
Q7TQ48;Q7TQ48-2	Srl	2	25	36.5	93	0	171.13	697930000	1459000000	1066300000	94507000	Srl	191	0.478361892	1.527803648	0.730843043	106393	Srl
Q7TSQ8; D6RD07	Pdpr	3	5	6.5	6	0	6.338	7399500	8141900	7428300	600730	Pdpr	1408	0.908817352	1.003892155	0.91235461	319518	Pdpr
Q80SZ7	Gng5	2	3	38.2	3	0	3.0247	58085000	34675000	42850000	35652000	Gng5	295	1.675126172	0.737711974	1.235760634	14707	Gng5
Q80WS3; P35550	Fbll1;Fbl	2	1	3.5	3	0	2.9289	1735100	1650300	1309100	293260	Fbll1	1518	1.051384597	0.75448101	0.793249712	237730	Fbl;Fbll1
Q80X90	Flnb	1	12	7.1	13	0	19.922	25514000	9369100	12907000	342950	Flnb	1503	2.723207138	0.505879125	1.377613645	286940	Flnb
Q80XB4-2;Q80XB4;E9PZF4;Q80XB4-4;Q80XB4-3;E9Q641	Nrap	7	41	25.8	55	0	83.524	113080000	46054000	312490000	3324600	Nrap	885	2.455378469	2.763441811	6.785295523	18175	Nrap
Q80XN0; D3Z2Y8	Bdh1	2	12	39.7	37	0	43.122	336820000	381540000	538240000	76505000	Bdh1	214	0.882790795	1.598004869	1.410703989	71911	Bdh1
Q80XR6;Q20BD0;Q99020	Hnrnpab	3	3	9.3	6	0	4.2657	15256000	10282000	13430000	3815200	Hnrnpab	845	1.483758024	0.880309386	1.306166116	15384	Hnrnpab
Q80Y14;A0A1Y7VN70	Glrx5	2	3	29.6	9	0	16.612	22479000	34640000	27848000	14468000	Glrx5	458	0.648931871	1.238845144	0.803926097	73046	Glrx5
Q80YD1	Supv3l1	2	3	4	3	0	4.3026	5074700	6173100	6150800	507000	Supv3l1	1445	0.822066709	1.212051944	0.996387552	338359	Supv3l1
Q80YV4-2;Q80YV4	Pank4	4	3	4	3	0	4.6825	3281900	6043900	3754900	365040	Pank4	1492	0.543010308	1.144123831	0.621271034	269614	Pank4
Q811U4;F6XRI9	Mfn1	4	8	12.8	13	0	14.81	15651000	20760000	17940000	1441100	Mfn1	1182	0.753901734	1.146252636	0.86416185	67414	Mfn1
Q8BFP9	Pdk1	2	10	28.3	22	0	31.398	47121000	78266000	67241000	9068800	Pdk1	588	0.602062198	1.426985845	0.859134235	228026	Pdk1
Q8BFR5;Q8BFR5-2	Tufm	4	20	46.9	62	0	102.79	651790000	1003400000	726650000	89556000	Tufm	197	0.649581423	1.114852943	0.724187762	233870	Tufm
Q8BG05-2;A2AL12;Q8BG05	Hnrnpa3	3	6	21.8	17	0	18.77	115140000	40697000	69418000	13088000	Hnrnpa3	487	2.82920117	0.602900816	1.705727695	229279	Hnrnpa3
Q8BG32;G3UZ33	Psmd11	10	8	21.1	13	0	11.272	15625000	16998000	17542000	1805200	Psmd11	1096	0.919225791	1.122688	1.032003765	69077	Psmd11
Q8BG51;Q8BG51-2;Q8BG51-3;Q8BG51-4;F7ASU3	Rhot1	5	3	6.2	4	0	4.4199	6336800	5237000	3568800	676070	Rhot1	1389	1.210005728	0.563186466	0.68145885	59040	Rhot1
Q8BGC4; A0A494BB38	Zadh2	2	8	23.3	12	0	15.568	28601000	49079000	32462000	6240500	Zadh2	684	0.582754335	1.13499528	0.661423419	225791	Ptgr3;Zadh2
Q8BGD8	Coa6	1	2	30.4	3	0	3.0238	2256900	4737100	3071000	2389200	Coa6	1014	0.476430728	1.360716026	0.648286927	67892	Coa6
Q8BGH2	Samm50	1	13	30.9	30	0	45.136	147940000	239330000	194450000	20975000	Samm50	375	0.618142314	1.31438421	0.812476497	68653	Samm50
Q8BGX2		1	1	6	1	0	3.0825	1663300	2175800	1124300	463810	Q8BGX2	1458	0.764454454	0.67594541	0.516729479	69773	Timm29
Q8BGY7	Fam210a	1	3	13.6	3	0	4.551	2263300	3679000	2230400	598730	Fam210a	1410	0.615194346	0.985463703	0.606251699	108654	Fam210a
Q8BH59	Slc25a12	2	19	37.1	74	0	116.12	432790000	734080000	515970000	54436000	Slc25a12	255	0.589567895	1.192194829	0.702879795	78830	Slc25a12
Q8BH61	F13a1	1	2	2.7	3	0	2.3609	7436100	5926400	8828200	600270	F13a1	1409	1.254741496	1.187208348	1.489639579	74145	F13a1
Q8BH64	Ehd2	2	14	26.3	33	0	61.73	153850000	190170000	164040000	19267000	Ehd2	395	0.809012988	1.066233344	0.862596624	259300	Ehd2
Q8BH80;Q9QY76	Vapb	2	5	27.2	11	0	10.831	64981000	78600000	70089000	15710000	Vapb	438	0.82673028	1.078607593	0.891717557	56491	Vapb
Q8BH95	Echs1	1	13	45.9	42	0	106.72	694240000	1348300000	862090000	213390000	Echs1	102	0.514900245	1.241775179	0.639390343	93747	Echs1
Q8BHE8		1	1	3.8	1	0.000615	2.1107	1110800	2899900	1183400	305360	Q8BHE8	1512	0.383047691	1.0653583	0.408083037	68115	Maip1
Q8BHN3; Q8BHN3-2;Q8BHN3-3	Ganab	3	8	8.4	10	0	13.783	20324000	6810100	18199000	908240	Ganab	1322	2.984390831	0.89544381	2.672354297	14376	Ganab
Q8BIJ6;E9PWN2	Iars2	3	17	20	30	0	36.657	73372000	118650000	88330000	7907100	Iars2	627	0.618390223	1.203865235	0.744458491	381314	Iars2
Q8BJ03-2;Q8BJ03	Cox15	2	1	3.5	1	0.0080128	1.3383	823200	1392200	871300	200030	Cox15	1545	0.591294354	1.058430515	0.625843988	226139	Cox15
Q8BJS4-3;Q8BJS4-2;Q8BJS4	Sun2	3	2	3.3	2	0	4.0131	5094500	4194300	4557700	445190	Sun2	1465	1.21462461	0.894631465	1.086641394	223697	Sun2
Q8BJY1	Psmd5	1	2	5.6	3	0.0006266	2.2528	3771300	2191700	1881600	324150	Psmd5	1509	1.720719077	0.4989261	0.858511658	66998	Psmd5
Q8BK30	Ndufv3	3	3	23.1	10	0	9.7182	67960000	112680000	97097000	38572000	Ndufv3	282	0.603123891	1.428737493	0.861705715	78330	Ndufv3
Q8BK64	Ahsa1	1	2	5.6	3	0.0006246	2.2301	5473000	5276200	4713100	795840	Ahsa1	1349	1.037299572	0.86115476	0.893275463	217737	Ahsa1
Q8BK72	Mrps27	1	1	3.6	3	0	3.0303	1397300	1684300	1271900	225860	Mrps27	1535	0.829602802	0.910255493	0.755150508	218506	Mrps27
Q8BKC5;Q8BKC5-2	Ipo5	2	4	3.6	2	0	7.4171	8767100	7799700	12736000	569130	Ipo5	1422	1.124030411	1.452703859	1.632883316	70572	Ipo5
Q8BKY8	Mterf2	1	2	5.2	2	0	3.0639	2081300	4335300	3711700	547100	Mterf2	1427	0.480082117	1.783356556	0.85615759	74238	Mterf2
Q8BKZ9	Pdhx	3	9	18.2	22	0	19.136	165260000	242450000	221830000	29815000	Pdhx	316	0.681625077	1.342309089	0.914951536	27402	Pdhx
Q8BL66	Eea1	1	1	0.6	3	0.0017472	1.7524	5684700	8999400	6864100	298980	Eea1	1513	0.631675445	1.207469172	0.762728626	216238	Eea1
Q8BLF1;Q8BYQ0	Nceh1	3	6	16.9	10	0	12.32	44428000	58784000	51531000	9600900	Nceh1	569	0.755783887	1.159876654	0.876616086	320024	Nceh1
Q8BMF3	Me3	3	8	15.1	9	0	15.452	47981000	86157000	63411000	6599500	Me3	674	0.556901935	1.321585628	0.735993593	109264	Me3
Q8BMF4	Dlat	1	17	35.5	63	0	118.56	1144400000	1505400000	1337800000	128390000	Dlat	158	0.760196625	1.168996854	0.888667464	235339	Dlat
Q8BMJ2;A0A494BA98	Lars	2	2	2.3	2	0	2.8521	1611900	1181700	1441200	69737	Lars	1566	1.36405179	0.89410013	1.219598883	107045	Lars;Lars1
Q8BMK4	Ckap4	1	13	25.4	21	0	50.591	98295000	17251000	50213000	4726900	Ckap4	770	5.697930555	0.510839819	2.910729813	216197	Ckap4
Q8BMS1	Hadha	1	34	55.6	144	0	295.74	3667200000	7577100000	4459800000	494510000	Hadha	58	0.483984638	1.216132199	0.588589302	97212	Hadha
Q8BMS4	Coq3	1	4	17	7	0	15.247	16813000	35128000	19117000	5396200	Coq3	730	0.478621043	1.137036817	0.544209747	230027	Coq3
Q8BP40	Acp6	1	2	5.3	3	0	3.0366	6805300	8438900	6954300	1189500	Acp6	1251	0.806420268	1.0218947	0.824076598	66659	Acp6
Q8BP67	Rpl24	1	6	35.7	13	0	15.352	77606000	43976000	52520000	23052000	Rpl24	358	1.76473531	0.676751798	1.194287793	68193	Rpl24
Q8BPB5	Efemp1	2	6	14.6	8	0	7.4773	31777000	797120	4593200	1477600	Efemp1	1173	39.86476315	0.144544797	5.762244079	216616	Efemp1

Q8BS97;E9QMK3;E9QMK2;G3XA35;E9PYH0;Q62059-4;Q62059-3;Q62059-2;Q62059	Vcan	9	3	10.9	4	0	4.3859	12537000	1204900	5820500	1022100	Vcan	1288	10.40501286	0.464265773	4.830691344	13003	Vcan
Q8BT60;Q9D6C8;A0A0R4J1D0;A0A0R4J0J1;Q3UYN2;P59108;Q1RLL3;Q9Z140;Q8BLR2;Q0VE82;Q9DC53;Q8JZW4	Cpne3;Cpne4;Cpne6;Cpne2;Cpne9;Cpne7;Cpne8;Cpne5	12	2	3.9	2	0.00491	1.4507	4854200	2567600	3662300	464760	Cpne3	1457	1.890559277	0.754460055	1.426351457	70568	Cpne2;Cpne4;Cpne6;Cpne9;Cpne3;Cpne5;Cpne7;Cpne8
Q8BTE5	Cebpzos	1	1	13.8	1	0	3.0591	3088700	3870400	4213700	2391000	Cebpzos	1013	0.798031211	1.364230906	1.088698843	68554	Cebpzos
Q8BTN3;D3Z2V8;D6RH68;Q99K56;A0A338P7G4;P54797	Tango2	9	3	9.1	7	0	3.623	8832000	15086000	8557400	2687000	Tango2	967	0.585443458	0.968908514	0.567241151	27883	Tango2
Q8BTS0;Q61656;S4R1I6	Ddx5	5	6	15.4	13	0	16.296	42233000	17884000	22487000	2685600	Ddx5	968	2.36149631	0.532450927	1.257380899	13207	Ddx5
Q8BTU6;P10630;P10630-2;A0A338P6X5;E9Q561	Eif4a2	9	2	26.8	3	0	3.9505	7520500	7276100	3808200	991910	Eif4a2	1293	1.033589423	0.506375906	0.52338478	13682	Eif4a2
Q8BVQ5;A0A140LI84	Ppme1	2	2	5.2	2	0.0006204	2.1633	817440	2737900	5486600	547380	Ppme1	1426	0.298564593	6.711929928	2.003944629	72590	Ppme1
Q8BVQ9;P46471	Psmc2	2	6	15.2	11	0	16.169	28906000	19859000	28710000	2435100	Psmc2	1008	1.45556171	0.993219401	1.44569213	19181	Psmc2
Q8BW75	Maob	3	11	25	13	0	26	33961000	60466000	27352000	5676600	Maob	716	0.561654484	0.805394423	0.452353389	109731	Maob
Q8BWF0	Aldh5a1	1	8	20.1	14	0	19.961	19349000	50120000	25191000	4104000	Aldh5a1	821	0.386053472	1.301927748	0.502613727	214579	Aldh5a1
Q8BWM0	Ptges2	1	5	13.8	6	0	10.392	28931000	34899000	31083000	4202300	Ptges2	812	0.828992235	1.074383879	0.890655893	96979	Ptges2
Q8BWT1	Acaa2	2	23	71	135	0	298.24	1743800000	5128200000	1732300000	490020000	Acaa2	59	0.34004134	0.993405207	0.337798838	52538	Acaa2
Q8BXK9	Clic5	1	3	13.5	4	0	4.0294	6784600	5081700	12497000	1764800	Clic5	1107	1.335104394	1.841965628	2.459216404	224796	Clic5
Q8BYL4;E9PX65	Yars2	2	2	4.4	3	0.00059	1.8349	2619900	5185900	3667200	522880	Yars2	1436	0.505196784	1.399748082	0.707148229	70120	Yars2
Q8BZA9; A0A0J9YV11	Tigar	2	2	8.9	5	0	4.2382	7990500	6233300	8701500	1720200	Tigar	1116	1.281905251	1.088980665	1.395970032	319801	Tigar
Q8BZF8	Pgm5	1	12	24.7	22	0	23.706	67543000	43115000	83276000	5896900	Pgm5	703	1.566577757	1.232933094	1.931485562	226041	Pgm5
Q8C0E2;Q8C0E2-2	Vps26b	2	1	5.4	2	0	4.4036	6386700	5795100	5390700	985960	Vps26b	1297	1.102086245	0.844050918	0.930216907	69091	Vps26b
Q8C0M9	Asrgl1	1	4	13.8	5	0	7.37	13446000	18613000	20302000	3735800	Asrgl1	853	0.722398324	1.509891418	1.090743029	66514	Asrgl1
Q8C129	Lnpep	1	4	4.1	8	0	7.4784	9450800	11581000	7856000	667770	Lnpep	1392	0.816060789	0.831252381	0.678352474	240028	Lnpep
Q8C266;P35278;P61021	Rab5c;Rab5b	5	2	24.8	10	0	10.331	43567000	26960000	28675000	10309000	Rab5c	544	1.615986647	0.658181651	1.06361276	19345	Rab5c;Rab5b
Q8C2Q7;P70333;O35737	Hnrnph1; Hnrnph2	5	3	9.1	7	0	24.416	25967000	10680000	12554000	2535100	Hnrnph1	996	2.431367041	0.483459776	1.175468165	59013	Hnrnph1; Hnrnph2
Q8C2Q8;A2AKU9;Q91VR2;A2AKV1;A2AKV2;A2AKV3	Atp5c1	7	12	36.9	85	0	49.699	1595400000	3462400000	2573300000	828570000	Atp5c1	28	0.460778651	1.61294973	0.7432128	11949	Atp5c1;Atp5f1c
Q8C3X2-2;Q8C3X2	Ccdc90b	2	6	24.8	6	0	7.1331	17244000	25270000	23024000	4899300	Ccdc90b	762	0.682390186	1.335189051	0.911119905	66365	Ccdc90b
Q8C483;P26638	Sars	4	3	5.6	5	0	4.4039	12007000	5596300	10293000	1039200	Sars	1284	2.145524722	0.857249938	1.839250934	20226	Sars;Sars1
Q8C605;Q9WUA3;Q9WUA3-2	Pfkp	4	4	9.4	7	0	7.6366	9898300	4555900	13254000	782840	Pfkp	1354	2.172633289	1.339017811	2.909194671	56421	Pfkp
Q8C6B0;Q9D7S5;H3BJI7;A0A2R8VI07;A0A2R8VK72;Q76I24;Q5I0W6;Q76I26	Mettl7a1; mCG_20149;Methig1;UbiE2;Mettl7a2	10	4	18	5	0	6.8	14288000	9094100	11237000	2677600	Mettl7a1	969	1.571128534	0.786464166	1.235636292	70152	Mettl7a1; Methig1;Mettl7a2
Q8C7H1	Mmaa	1	2	6	2	0.0029036	1.7313	1793900	3947100	2348500	416280	Mmaa	1473	0.454485572	1.309158816	0.594993793	109136	Mmaa
Q8C845;Q9D8Y0	Efhd2	2	4	17.1	7	0	14.975	22273000	5859900	21404000	4193300	Efhd2	813	3.800918104	0.960984151	3.652622058	27984	Efhd2
Q8CAK1	Iba57	1	2	7	4	0	4.1182	1965000	3985700	2533000	720090	Iba57	1373	0.49301252	1.289058524	0.635521991	216792	Iba57
Q8CB58;Q8BGJ5;Q922I7; F7AXP1;E9QMW9;P17225	Ptbp1	6	3	9.2	3	0	3.8373	6451600	1394000	1633900	537690	Ptbp1	1434	4.628120517	0.253255007	1.172094692	19205	Ptbp1
Q8CBB6; Q8CGP2;Q8CGP1;Q64475;P10853;Q8CGP2-2;Q9D2U9;Q8CGP0; P70696;Q64524	Hist1h2br;Hist1h2bp;Hist1h2bk;Hist1h2bb;Hist1h2bf;Hist3h2ba;Hist3h2bb;Hist1h2ba;Hist2h2be	10	1	50	2	1	-2	1032400000	232070000	529570000	221700000	Hist1h2br	99	4.448657733	0.512950407	2.281940794	665596	H2bc1;H2bc12;H2bc21;H2bc24;H2bc26;H2bc3;H2bc7;H2bc11;H2bc13;H2bc15;H2bu2;Hist1h2bp
Q8CBB7; P22892	Ap1g1	2	1	1.1	1	0.000596	1.9171	1813600	1623400	1769100	159290	Ap1g1	1552	1.117161513	0.975463167	1.089749908	11765	Ap1g1
Q8CBM2;A2AL85;Q8BSY0	Asph	11	3	8.7	6	0	12.803	19567000	7668100	12039000	1397200	Asph	1192	2.551740327	0.615270609	1.570010824	65973	Asph
Q8CBY8-2;Q8CBY8	Dctn4	2	1	2.4	1	0	3.0052	1851200	1337400	1782600	219660	Dctn4	1537	1.384178256	0.962942956	1.332884702	67665	Dctn4
Q8CC88;Q8CC88-2	Vwa8	3	35	19.6	59	0	85.882	135370000	264660000	162230000	5942100	Vwa8	698	0.511486435	1.198419148	0.612975138	219189	Vwa8
Q8CG48	Smc2	1	1	0.8	1	0.0028539	1.6592	18692000	13201000	20945000	838010	Smc2	1340	1.415953337	1.120532848	1.586622226	14211	Smc2
Q8CG76	Akr7a2	1	5	18.8	7	0	15.152	22990000	34803000	24244000	6085900	Akr7a2	688	0.660575238	1.054545455	0.696606614	110198	Akr7a2
Q8CGC7	Eprs	3	17	13.3	26	0	35.696	74250000	50450000	71694000	2455600	Eprs	1005	1.471754212	0.965575758	1.421090188	107508	Eprs1
Q8CGK3	Lonp1	1	18	23.8	34	0	60.025	111210000	122590000	133110000	9616700	Lonp1	568	0.907170242	1.196924737	1.085814504	74142	Lonp1
Q8CGY6-2;Q8CGY6	Unc45b	2	3	3.6	2	0	7.115	7467900	8560800	10635000	487030	Unc45b	1449	0.872336698	1.424095127	1.24229044	217012	Unc45b
Q8CHP8	Pgp	1	4	16.2	6	0	8.3626	8981900	7996200	7094400	1599900	Pgp	1142	1.123271054	0.789855153	0.88722143	67078	Pgp
Q8CHS7	Dhrs7c	1	2	8.7	4	0	4.2654	6319500	11449000	8334200	1673900	Dhrs7c	1127	0.551969604	1.318806868	0.727941305	68460	Dhrs7c
Q8CHT0	Aldh4a1	1	11	23.1	23	0	27.036	148590000	252690000	184810000	26547000	Aldh4a1	338	0.588032767	1.243757992	0.731370454	212647	Aldh4a1
Q8CI94	Pygb	1	27	47.2	87	0	169.11	510810000	669720000	552500000	41199000	Pygb	275	0.762721734	1.081615473	0.82497163	110078	Pygb
Q8CIB5;A6X941	Fermt2	2	5	10.3	8	0	10.221	30842000	21339000	27377000	2292500	Q8CIB5	1023	1.445334833	0.8876532	1.28295609	218952	Fermt2
Q8CJ53-4;Q8CJ53-3;Q8CJ53-2;Q8CJ53	Trip10	4	2	3.8	3	0	2.7235	3385800	2283200	3652600	366140	Trip10	1491	1.482918711	1.078799693	1.599772249	106628	Trip10
Q8JZN5	Acad9	3	14	23.7	24	0	37.916	58405000	68756000	66128000	5865200	Acad9	706	0.849453139	1.132231829	0.961777881	229211	Acad9
Q8JZQ2	Afg3l2	3	13	17.1	21	0	48.358	62142000	73341000	70786000	4990400	Afg3l2	758	0.847302327	1.139100769	0.965162733	69597	Afg3l2
Q8JZQ9	Eif3b	2	5	6.4	5	0	9.2445	11466000	2236700	4999900	458550	Eif3b	1462	5.126302142	0.436063143	2.235391425	27979	Eif3b
Q8K010;E9Q484	Oplah	4	4	3.7	5	0	10.237	3916500	8112000	2681000	364260	Oplah	1493	0.482803254	0.684539768	0.330498028	75475	Oplah
Q8K0D5	Gfm1	1	10	16.5	16	0	17.261	40432000	67585000	44231000	4286700	Gfm1	805	0.598239254	1.09396023	0.654449952	28030	Gfm1
Q8K0E8	Fgb	2	9	23.1	14	0	48.069	84515000	54188000	78958000	8755300	Fgb	594	1.559662656	0.934248358	1.457112276	110135	Fgb
Q8K0Z7	Taco1	1	4	16.7	7	0	8.0991	18775000	23200000	26912000	5248700	Taco1	741	0.809267241	1.433395473	1.16	70207	Taco1
Q8K157	Galm	1	2	8.5	2	0.0017442	1.7418	4046600	3753800	3233100	887150	Galm	1327	1.078000959	0.798967034	0.861287229	319625	Galm
Q8K1K2; P62196	Psmc5	2	4	14.9	8	0	8.8814	16364000	16849000	21607000	2737300	Psmc5	960	0.971214909	1.320398436	1.282390646	19184	Psmc5
Q8K1M3;A0A0A6YX73;P12367	Prkar2a	6	5	12.9	8	0	8.2776	123830000	138340000	148640000	29770000	Prkar2a	317	0.895113489	1.200355326	1.074454243	19087	Prkar2a
Q8K1Z0;F6SFF5	Coq9	3	8	30.7	19	0	104.44	231950000	450310000	263630000	103750000	Coq9	179	0.515089605	1.13658116	0.585441141	67914	Coq9
Q8K2B3	Sdha	2	28	46.7	178	0	211.77	1266600000	2710400000	1766600000	204270000	Sdha	109	0.467311098	1.394757619	0.651785714	66945	Sdha
Q8K2I3	Fmo2	2	3	7.3	3	0	8.357	6796500	1406900	1732900	335970	Fmo2	1505	4.830833748	0.25496947	1.231715118	55990	Fmo2
[illegible]2;Q8K2M0	Mrpl38	2	1	2.9	1	0.0005924	1.8594	2716100	4126300	3223100	668170	Mrpl38	1391	0.658241039	1.186664703	0.781111407	60441	Mrpl38
Q8K2T4	Uqcc3	1	1	11.2	2	0.0033917	1.6189	2217600	4005900	3930000	2837100	Uqcc3	949	0.553583464	1.772186147	0.981052947	107197	Uqcc3
Q8K310;A0A494BAZ2	Matr3	11	3	3	3	0	3.0197	7007200	5506500	6949300	560910	Matr3	1423	1.272532462	0.99173707	1.262917616	17184	Matr3

Q8K370	Acad10	1	2	12.1	2	0	3.1168	17542000	22594000	12840000	1036500	Acad10	1285	0.776400814	0.731957588	0.568292467	71985	Acad10
Q8K3C3	Lzic	1	2	11.1	1	0	2.6486	18099000	12961000	12109000	4711300	Lzic	773	1.396420029	0.669042489	0.934264331	69151	Lzic
Q8K3J1;A0A494BAW8	Ndufs8	2	7	34.4	20	0	18.381	414810000	614120000	374950000	127470000	Ndufs8	159	0.675454309	0.903907813	0.610548427	225887	Ndufs8
Q8K411-2;Q8K411;Q8K411-3	Pitrm1	3	4	4.1	7	0	8.4085	7686400	4208800	9622500	405090	Pitrm1	1482	1.82626877	1.251886449	2.286281125	69617	Pitrm1
Q8K4F5;D3YYK0	Abhd11	3	3	9.1	4	0	4.8729	15580000	22978000	15853000	3804800	Abhd11	847	0.678039864	1.017522465	0.689920794	68758	Abhd11
Q8K4L2;E9Q3Z5;A0A1B0GS91;Q8K4L3;Q8K4L3-3	Svil	6	5	2.9	8	0	4.9866	7077800	6038200	8857100	202290	Svil	1544	1.172170514	1.251391675	1.466844424	225115	Svil
Q8K4Z3	Apoa1bp	1	4	17.4	8	0	9.2518	36495000	41173000	34176000	9241900	Apoa1bp	583	0.886381852	0.936457049	0.830058534	246703	Naxe
Q8QZR5;A0A2R8VHV7;A0A2R8VHW0	Gpt	4	3	6.5	4	0	5.5439	5420400	9146500	5079600	1227400	Gpt	1242	0.592620128	0.937126411	0.555359974	76282	Gpt
Q8QZS1;E0CX19	Hibch	2	15	44.4	32	0	71.498	130520000	250910000	195840000	27694000	Hibch	329	0.520186521	1.5004597	0.780518911	227095	Hibch
Q8QZT1	Acat1	1	17	42.2	66	0	180.17	799990000	1295700000	821720000	157460000	Acat1	133	0.617419156	1.02716284	0.634190013	110446	Acat1
Q8QZY1	Eif3l	1	6	9.6	6	0	8.0598	22089000	14748000	13200000	1800000	Eif3l	1098	1.497762408	0.597582507	0.895036615	223691	Eif3l
Q8R086	Suox	1	3	5.5	3	0	4.1874	12272000	8903100	11372000	1386100	Suox	1195	1.378396289	0.926662321	1.277307904	211389	Suox
Q8R0F8	Fahd1	1	2	10.1	6	0	14.414	12223000	17329000	18493000	3705900	Fahd1	856	0.705349414	1.512967357	1.067170639	68636	Fahd1
Q8R0Y8	Slc25a42	1	2	5.3	2	0.0011744	1.8073	5118000	15482000	7186200	2142900	Slc25a42	1039	0.330577445	1.404103165	0.464164837	73095	Slc25a42
Q8R164	Bphl	1	7	27.1	13	0	20.929	26678000	34375000	26536000	5611900	Bphl	719	0.776087273	0.994677262	0.771956364	68021	Bphl
Q8R1B4	Eif3c	2	6	7.1	9	0	12.273	15187000	8219800	9192100	989430	Eif3c	1295	1.847611864	0.605261079	1.11828755	56347	Eif3c
Q8R1G2	Cmbl	3	3	11.4	6	0	2.7064	14080000	14900000	17165000	3037400	Cmbl	927	0.944966443	1.219105114	1.152013423	69574	Cmbl
Q8R1I1	Uqcr10	1	3	50	20	0	13.355	482840000	1003500000	466710000	581030000	Uqcr10	43	0.481155954	0.966593489	0.465082212	66152	Uqcr10
Q8R1Q8	Dync1li1	1	2	3.3	2	0	2.5729	16901000	7914900	14630000	1311700	Dync1li1	1212	2.135339676	0.865629253	1.848412488	235661	Dync1li1
Q8R1S0;D3YW66	Coq6	4	9	21.8	22	0	31.293	65801000	90694000	79364000	13444000	Coq6	481	0.725527598	1.206121488	0.875074426	217707	Coq6
Q8R2K3;Q9CYR0	Ssbp1	3	3	21.6	5	0	4.6527	11845000	18736000	15224000	6526200	Ssbp1	676	0.63220538	1.285268046	0.812553373	381760	Ssbp1
Q8R2P8;Q99MN1	Kars	2	4	6.2	8	0	4.4942	14415000	12657000	12287000	1396200	Kars	1193	1.138895473	0.852375997	0.970767164	85305	Kars;Kars1
Q8R3K3	Ptcd2	1	1	3.1	1	0	2.8092	1391500	2915000	1200600	356570	Ptcd2	1494	0.477358491	0.862809917	0.41186964	68927	Ptcd2
Q8R3V2;Q8R010	Aimp2	2	3	12.1	3	0	3.5752	6359300	6559200	7344000	1839100	Aimp2	1093	0.969523722	1.154844087	1.119648738	231872	Aimp2
Q8R404	Qil1	1	2	24.4	2	0	10.731	9660600	24030000	10117000	19039000	Qil1	400	0.402022472	1.047243442	0.421015397	224904	Micos13
Q8R4N0	Clybl	1	9	32.5	18	0	21.891	71609000	79999000	73301000	11555000	Clybl	514	0.895123689	1.023628315	0.916273953	69634	Clybl
Q8R5J9	Arl6ip5	1	1	5.9	3	0	4.4623	11622000	11635000	9470700	7355100	Arl6ip5	639	0.998882682	0.814894166	0.81398367	65106	Arl6ip5
Q8R5L1;O35658	C1qbp	2	4	16.8	13	0	45.568	68790000	121990000	81616000	26523000	C1qbp	339	0.56389868	1.186451519	0.669038446	12261	C1qbp
Q8VBT1	Txlnb	1	4	6.3	6	0	7.1414	5806100	5574600	7540900	654610	Txlnb	1396	1.041527643	1.298789204	1.352724859	378431	Txlnb
Q8VCF0	Mavs	1	1	3.4	2	0	2.7151	1355000	935440	1152000	182590	Mavs	1547	1.448516206	0.850184502	1.231506029	228607	Mavs
Q8VCQ8;S4R1T7;E9Q0M9;E9QA16;D3Z6I7;E9QA15	Cald1	11	9	19.8	13	0	21.44	53675000	4742900	19523000	2969100	Cald1	938	11.31691581	0.363726129	4.116257986	109624	Cald1
Q8VCT4	Ces1d	1	3	11	4	0	5.3547	5350300	18081000	5442500	1327300	Ces1d	1207	0.295907306	1.017232679	0.301006581	104158	Ces1d
Q8VCW8	Acsf2	1	7	13.8	12	0	16.99	23927000	27837000	28241000	3312500	Acsf2	887	0.859539462	1.180298408	1.014513058	264895	Acsf2
Q8VDD5	Myh9	4	42	31.7	102	0	242.52	799390000	194510000	352020000	12780000	Myh9	494	4.109762994	0.440360775	1.809778418	17886	Myh9
Q8VDG5-2;Q8VDG5	Ppcs	2	1	8	1	0.0034169	1.6473	1813400	1602700	1785300	553730	Ppcs	1424	1.131465652	0.984504246	1.113932739	106564	Ppcs
Q8VDJ3;A0A087WP83	Hdlbp	6	9	9.1	11	0	28.451	21421000	13231000	14962000	805250	Hdlbp	1347	1.619000831	0.698473461	1.130829113	110611	Hdlbp
Q8VDM4	Psmd2	2	6	7.3	13	0	16.584	35798000	23352000	31469000	1789900	Psmd2	1102	1.532973621	0.879071457	1.347593354	21762	Psmd2
Q8VDN2	Atp1a1	4	21	37.6	112	0	239.32	720070000	1007000000	787360000	60708000	Atp1a1	239	0.715064548	1.093449248	0.781886792	11928	Atp1a1
Q8VE38-2;Q8VE38;E9PZL5;E9Q690;E9Q8U8	Oxnad1	8	3	12.4	2	0	2.9272	8413200	14467000	13488000	2434400	Oxnad1	1009	0.581544204	1.603194979	0.932328748	218885	Oxnad1
Q8VE95;Q9D1W4		2	2	11.5	4	0	5.8957	6545800	12035000	7312300	2047700	Q8VE95	1055	0.543896967	1.117097987	0.607586207	223665	C030006K11Rik
Q8VEE1	Lmcd1	1	3	9.9	5	0	6.0788	6804700	3067000	6933100	696020	Lmcd1	1382	2.218682752	1.018869311	2.260547767	30937	Lmcd1
Q8VEK3-2;Q8VEK3	Hnrnpu	2	8	10	13	0	13.39	54410000	29789000	30694000	3584200	Hnrnpu	867	1.826513142	0.564124242	1.030380342	51810	Hnrnpu
Q8VFC3	Olfr266	1	1	3.2	3	1	-2	74868000	99284000	72958000	35497000	Olfr266	296	0.754079207	0.974488433	0.734841465	258482	Or11i1
Q8VHX6-2;Q8VHX6	Flnc	2	58	29	139	0	225.06	618040000	409220000	1105000000	13518000	Flnc	480	1.510287865	1.787910168	2.700259029	68794	Flnc
Q8VIJ6	Sfpq	2	7	13.2	12	0	12.959	48140000	20609000	25164000	3250600	Sfpq	896	2.335872677	0.522725384	1.221019943	71514	Sfpq
Q8WTY4;E0CYY1	Ciapin1	3	3	11.3	6	0	4.5228	3356500	6752800	5088600	949040	Ciapin1	1305	0.497053074	1.516043498	0.753554081	109006	Ciapin1
Q91V16	Lyrm5	1	2	18.6	5	0	4.744	9793700	23997000	19811000	15468000	Lyrm5	441	0.408121849	2.022831004	0.825561529	67636	Etfrf1
Q91V41;Q50HX3	Rab14	3	6	30.7	9	0	9.9675	36736000	36215000	37982000	7650400	Rab14	633	1.014386304	1.033917683	1.048791937	68365	Rab14
Q91V55;P97461;D3YYM6;D3Z1S8	Rps5	4	5	27.5	9	0	6.7948	60167000	35728000	54711000	15411000	Rps5	443	1.684029333	0.909319062	1.531319973	20103	Rps5
Q91V77;P56565	S100a1	2	3	37.2	8	0	33.552	167940000	175480000	297000000	136240000	S100a1	153	0.95703214	1.768488746	1.69250057	20193	S100a1
Q91VA7	Idh3b	2	15	45.3	51	0	76.922	543630000	909250000	700850000	134320000	Idh3b	156	0.59788837	1.289204054	0.77080011	170718	Idh3b
Q91VB8;P01942	haemaglobin alpha 2;Hba	2	4	43	203	0	49.844	4049300000	4638000000	4162300000	1.812E+09	haemaglobin alpha 2	12	0.873070289	1.027906058	0.897434239	110257	Hba; Hba-a1
Q91VC9;A0A286YDK2	Ghitm	2	2	5.8	3	0	6.3508	11406000	4025600	6722600	2133500	Ghitm	1041	2.833366455	0.589391548	1.669962242	66092	Ghitm
Q91VD9	Ndufs1	5	31	50.8	134	0	220.66	1866300000	2955700000	2098800000	192470000	Ndufs1	114	0.631424028	1.124578042	0.710085597	227197	Ndufs1
Q91VJ2	Prkcdbp	1	2	7.7	3	0.0006238	2.2186	3226500	2842300	3249400	812860	Prkcdbp	1344	1.13517222	1.007097474	1.143229075	109042	Cavin3
Q91VK1;A0A1Y7VIU9;A0A1Y7VJ43;A0A1Y7VM24;A0A1Y7VM47	Bzw2	6	3	10	6	0	9.4791	11146000	15527000	15091000	1716200	Bzw2	1118	0.717846332	1.353938633	0.971919881	66912	Bzw2
Q91VR5	Ddx1	2	5	6.8	5	0	8.504	14161000	7819900	14074000	942410	Ddx1	1308	1.810892722	0.993856366	1.79976726	104721	Ddx1
Q91VT4;D3Z6C4	Cbr4	2	6	33.5	11	0	19.293	36178000	59488000	44464000	17927000	Cbr4	407	0.608156267	1.22903422	0.747444863	234309	Cbr4
Q91VW3;I7HPY0	Sh3bgrl3	2	2	18.3	4	0	3.1668	85688000	9220300	25373000	28692000	Sh3bgrl3	321	9.293406939	0.29610914	2.751862738	73723	Sh3bgrl3
Q91WD5;D3YXT0	Ndufs2	4	15	41	72	0	90.486	687890000	1145500000	768300000	113400000	Ndufs2	171	0.600515059	1.116893689	0.67071148	226646	Ndufs2
Q91WK1	Spryd4	1	3	15	3	0	4.5682	3604800	5455400	5030700	1236900	Spryd4	1234.5	0.660776476	1.395555925	0.92215053	66701	Spryd4
Q91WM2	Cecr5	1	1	3.3	1	0	2.6227	1760500	2710400	2573300	374450	Cecr5	1488	0.649535124	1.461687021	0.94941706	214932	Hdhd5
Q91WS0	Cisd1	1	5	44.4	22	0	22.197	171170000	197910000	156250000	100650000	Cisd1	183	0.86488808	0.912835193	0.789500278	52637	Cisd1
Q91X72	Hpx	2	15	34.3	34	0	31.25	197150000	135430000	185000000	20430000	Hpx	377	1.455733589	0.938371798	1.366019346	15458	Hpx
Q91XH5;Q64105;G3UXX3	Spr	4	11	55	28	0	32.551	96149000	102240000	90734000	24896000	Spr	347	0.940424491	0.943681162	0.887460876	20751	Spr
Q91XL1	Lrg1	1	1	4.1	1	0	2.3135	2066500	1899500	2801200	587890	Lrg1	1414	1.087917873	1.355528672	1.474703869	76905	Lrg1
Q91YN9	Bag2	1	4	19.5	4	0	3.1502	11223000	9736500	18351000	2535500	Bag2	995	1.152672932	1.635124298	1.884763519	213539	Bag2
Q91YP0	L2hgdh	1	4	9.5	4	0	12.642	5433200	11322000	6600600	1070300	L2hgdh	1276	0.47987988	1.214864168	0.582988871	217666	L2hgdh
Q91YQ5	Rpn1	2	14	23.5	20	0	27.317	91392000	29251000	49523000	4797400	Rpn1	769	3.124406003	0.541874562	1.693036136	103963	Rpn1
Q91YR1	Twf1	1	3	14	4	0	3.7779	5332600	1784700	2318000	472700	Twf1	1456	2.987953157	0.434684769	1.298817728	19230	Twf1
Q91YZ8;A3KFU5;A3KFU8;Q6PHQ9	Pabpc4	5	5	12.4	12	0	12.159	16721000	13939000	16703000	1663800	Pabpc4	1129	1.199583901	0.998923509	1.19829256	230721	Pabpc4
Q91Z25;Q9WV32;F6THG2	Arpc1b	4	4	15.4	5	0	11.976	25578000	6496300	7559900	2224000	Arpc1b	1030	3.937318166	0.295562593	1.163723966	11867	Arpc1b
Q91Z83	Myh7	5	36	64	193	0	323.31	4277900000	401150000	8048400000	105680000	Myh7	175	10.66409074	1.881390402	20.06331796	140781	Myh7

Q91ZA3	Pcca	6	21	34.1	42	0	55.5	145680000	269910000	190920000	16304000	Pcca	425	0.539735467	1.310543657	0.707346893	110821	Pcca
Q91ZJ5-2;Q91ZJ5	Ugp2	2	13	28.2	26	0	32.783	126160000	163200000	287480000	22545000	Ugp2	360.5	0.773039216	2.278693722	1.761519608	216558	Ugp2
Q920Q6-3;Q920Q6-2;Q920Q6;B1AT10	Msi2	5	4	20.2	4	0	8.7519	6620200	5112100	5963500	1421500	Msi2	1187	1.295005966	0.900803601	1.166546038	76626	Msi2
Q921G7;Q6PF96	Etfdh	2	25	48.2	101	0	181.69	1018600000	2101100000	1343800000	160970000	Etfdh	130	0.48479368	1.319261732	0.639569749	66841	Etfdh
Q921I1	Tf	8	35	53.7	103	0	155.42	870080000	674740000	576260000	58215000	Tf	245	1.289504105	0.662306914	0.854047485	22041	Tf
Q921M7; A0A2I3BRN5	Fam49b	5	4	13.9	3	0	6.0749	8123200	3804000	3976900	924570	Fam49b	1317	2.135436383	0.489573075	1.045452156	223601	Cyrib
Q921R2;P62301	Rps13	3	4	31.4	10	0	9.4	58055000	34225000	50350000	18556000	Rps13	404	1.696274653	0.867281027	1.471146823	68052	Rps13
Q921S7	Mrpl37	1	1	3.8	3	0	3.0781	1491700	1960600	1154300	180660	Mrpl37	1548	0.760838519	0.77381511	0.588748342	56280	Mrpl37
Q922B1	Macrod1	1	5	20.7	11	0	25.49	94373000	222010000	119510000	37293000	Macrod1	286	0.425084456	1.266357963	0.538309085	107227	Macrod1
Q922B2	Dars	2	12	25.1	23	0	21.211	80634000	51245000	68031000	5917500	Dars	700	1.573499854	0.843701168	1.327563665	226414	Dars1
Q922D8;A0A1W2P733	Mthfd1	2	2	2	2	0.0006135	2.0829	5729200	4126800	6047300	333310	Mthfd1	1507	1.38829117	1.055522586	1.465372686	108156	Mthfd1
Q922Q1	Marc2	2	4	12.1	4	0	7.3301	9364800	9406400	14371000	2037000	Marc2	1058	0.995577479	1.534576286	1.52778959	67247	Mtarc2
Q922Q8	Lrrc59	1	4	14.7	5	0	6.4626	12409000	3876400	5542600	1491400	Lrrc59	1169	3.20116603	0.446659682	1.429831803	98238	Lrrc59
Q924B0;O55023;Q80ZJ2	Impa1	5	6	23.1	8	0	5.5893	20694000	12091000	18725000	3314700	Impa1	886	1.711520966	0.904851648	1.548672566	55980	Impa1
Q924M7	Mpi	1	4	13.7	6	0	8.4695	12326000	13056000	13766000	2059700	Mpi	1052	0.94408701	1.116826221	1.054381127	110119	Mpi
Q924X2	Cpt1b	3	23	30.8	94	0	135.37	878380000	1576900000	1202300000	112630000	Cpt1b	172	0.557029615	1.368769781	0.762445304	12895	Cpt1b
Q925I1-2; Q925I1	Atad3	4	8	17.6	11	0	16.824	22455000	30018000	33444000	3194700	Atad3	910	0.748051169	1.489378758	1.114131521	108888	Atad3
Q99J39-2;Q99J39	Mlycd	2	6	13.9	8	0	13.321	18175000	21924000	17257000	3149100	Mlycd	912	0.829000182	0.949491059	0.787128261	56690	Mlycd
Q99J99;A0A2R8VHE5;A0A2R8VHT1	Mpst	3	2	7.4	1	0	2.4437	3120200	11765000	5014200	1501500	Mpst	1164	0.26521037	1.607012371	0.426196345	246221	Mpst
Q99JB8	Pacsin3	8	8	23.1	14	0	18.193	24217000	24088000	35147000	4149800	Pacsin3	816	1.005355364	1.451335838	1.45910827	80708	Pacsin3
Q99JI4	Psmd6	2	7	21.6	10	0	19.914	11066000	8645000	9846400	1295900	Psmd6	1214	1.28004627	0.889788541	1.138970503	66413	Psmd6
Q99JY0	Hadhb	2	21	48.6	119	0	125.62	1838400000	4079600000	2239800000	400570000	Hadhb	68	0.450632415	1.218342037	0.549024414	231086	Hadhb
Q99JY9	Actr3	6	6	17.5	8	0	11.973	31473000	8477300	13663000	2447300	Actr3	1007	3.712620764	0.434118133	1.611715994	74117	Actr3
Q99JZ4;P36536	Sar1a	5	2	23.7	8	0	8.5532	55815000	36441000	52925000	15499000	Sar1a	440	1.531653906	0.948221804	1.45234763	20224	Sar1a
Q99KC8	Vwa5a	2	8	10.8	11	0	9.8632	25848000	11864000	15243000	1252400	Vwa5a	1229	2.178691841	0.589716806	1.284811194	67776	Vwa5a
Q99KI0	Aco2	3	36	55.5	294	0	323.31	5655300000	9985400000	6785500000	691620000	Aco2	35	0.566356881	1.19984793	0.679542132	11429	Aco2
Q99KJ8	Dctn2	1	11	33.8	22	0	23.577	46761000	36457000	43131000	6288700	Dctn2	682	1.282634336	0.922371207	1.183064981	69654	Dctn2
Q99KQ4	Nampt	1	11	23.2	23	0	21.418	49497000	85251000	53013000	7574600	Nampt	634	0.58060316	1.071034608	0.621846078	59027	Nampt
Q99KR7	Ppif	2	7	24.3	23	0	9.0648	264320000	381960000	293640000	80254000	Ppif	209	0.692009635	1.11092615	0.768771599	105675	Ppif
Q99KV1	Dnajb11	1	1	3.1	2	0.0006192	2.1586	3596600	1096900	1908100	417610	Dnajb11	1472	3.278876835	0.530528833	1.7395387	67838	Dnajb11
Q99L13	Hibadh	1	9	34	25	0	44.973	48393000	94621000	53745000	17677000	Hibadh	413	0.511440378	1.110594507	0.568002875	58875	Hibadh
Q99LB2;A0A2I3BRG8	Dhrs4	5	6	26.2	16	0	22.219	76800000	125670000	87951000	22404000	Dhrs4	363	0.611124373	1.145195313	0.699856768	28200	Dhrs4
Q99LC3	Ndufa10	2	16	52.4	50	0	55.109	677440000	1043900000	898610000	142840000	Ndufa10	146	0.648951049	1.326479098	0.860820002	67273	Ndufa10
Q99LC5	Etfa	1	14	52.6	118	0	179.36	2044600000	3962700000	2214200000	495030000	Etfa	57	0.515961339	1.08295021	0.558760441	110842	Etfa
Q99LF4	Rtcb	1	7	16.2	9	0	11.476	17474000	11211000	13862000	1799000	Rtcb	1099	1.558647757	0.793292892	1.236464187	28088	Rtcb
Q99LP6	Grpel1	1	5	23	6	0	8.2737	37096000	44735000	48412000	9857000	Grpel1	558	0.829238851	1.305046366	1.082195149	17713	Grpel1
Q99LX0;A2A813;A2A815;A2A817;A2A816	Park7	5	8	50.3	24	0	24.163	269560000	230260000	264620000	70781000	Park7	224	1.170676626	0.981673839	1.149222618	57320	Park7
Q99LY9;B1ARW4	Ndufs5	2	5	50.9	20	0	39.914	203690000	376690000	235410000	161950000	Ndufs5	128	0.540736415	1.15572684	0.624943588	595136	Ndufs5
Q99M07	Coa5	1	1	13.5	2	0.0075107	1.3531	1031500	1592500	2370400	1743100	Coa5	1112	0.647723705	2.298012603	1.488477237	76178	Coa5
Q99M28-2		1	1	2.5	1	0.010058	1.3048	15301000	16708000	18795000	4537400	Q99M28-2	783	0.915788844	1.228351088	1.124910223		
Q99M87-3;Q99M87-2;Q99M87	Dnaja3	3	4	12.8	5	0	8.6804	7355800	11318000	9020000	1498100	Dnaja3	1167	0.649920481	1.226243237	0.796960594	83945	Dnaja3
Q99MN9;E9Q1J7	Pccb	4	15	33.8	31	0	78.385	102050000	187370000	134410000	17120000	Pccb	416	0.544644287	1.317099461	0.717350696	66904	Pccb
Q99MQ4	Aspn	1	7	19.3	9	0	40.157	151230000	4815700	24289000	9243000	Aspn	582	31.40353427	0.160609667	5.043711195	66695	Aspn
Q99MR8	Mccc1	3	15	26.6	29	0	37.415	58028000	118790000	65182000	8281900	Mccc1	616	0.488492297	1.123285311	0.548716222	72039	Mccc1
Q99MR9	Ppp1r3a	1	2	2.3	4	0	4.5538	3691600	3625300	3063300	203480	Ppp1r3a	1543	1.018288142	0.829802796	0.844978347	140491	Ppp1r3a
Q99N15;A2AFQ2;O08756	Hsd17b10	3	12	58.6	38	0	99.605	161090000	308870000	207150000	56719000	Hsd17b10	249	0.521546282	1.285927121	0.670670509	15108	Hsd17b10
Q99N93	Mrpl16	1	2	9.2	3	0	3.7992	2952200	3314400	3570500	1045200	Mrpl16	1283	0.890719286	1.20943703	1.077268887	94063	Mrpl16
Q99NB1	Acss1	1	19	31.2	42	0	77.891	262730000	456790000	301360000	36474000	Acss1	290	0.575165831	1.147033076	0.659734232	68738	Acss1
Q99P30-5;Q99P30-3;Q99P30;Q99P30-2;Q99P30-4	Nudt7	5	2	14	3	0	4.8631	4109400	13339000	6860000	3385400	Nudt7	882	0.308074069	1.669343456	0.51428143	67528	Nudt7
Q99PR8	Hspb2	2	3	20.9	3	0	10.005	16243000	11370000	44206000	8195300	Hspb2	618	1.428583993	2.721541587	3.887950748	69253	Hspb2
Q99PT1	Arhgdia	1	8	49.5	19	0	42.2	102420000	42418000	77534000	25238000	Arhgdia	346	2.414540997	0.757020113	1.827856099	192662	Arhgdia
Q9CPP6	Ndufa5	2	5	43.1	19	0	34.948	216370000	465840000	292380000	229280000	Ndufa5	96	0.46447278	1.35129639	0.627640392	68202	Ndufa5
Q9CPQ1	Cox6c	2	10	63.2	60	0	32.556	1171600000	1951700000	1424300000	1.001E+09	Cox6c	25	0.600297177	1.215687948	0.729774043	12864	Cox6c
Q9CPQ3	Tomm22	3	4	48.6	8	0	32.445	13552000	15835000	16119000	7104400	Tomm22	651	0.855825703	1.189418536	1.017934954	223696	Tomm22
Q9CPQ8	Atp5l	2	6	55.3	36	0	19.827	514680000	905880000	644480000	504840000	Atp5l	53	0.568154722	1.252195539	0.711440809	27425	Atp5mg
Q9CPU0; A0A494BAF7;A0A494BBE7	Glo1	4	6	31.5	15	0	17.153	88457000	132620000	104660000	33070000	Glo1	305	0.666995928	1.183173745	0.789172071	109801	Glo1
Q9CPU2	Ndufb2	1	1	8.6	4	0.0005914	1.8437	6274300	11287000	4299100	7063000	Ndufb2	652	0.555887304	0.685191974	0.380889519	68198	Ndufb2
Q9CPU4	Mgst3	1	5	37.9	14	0	14.438	187030000	336270000	246420000	155360000	Mgst3	136	0.556189966	1.31754264	0.732803997	66447	Mgst3
Q9CPV4;E9Q197;Q9CPV4-3;Q9CPV4-2;F6ZTG3	Glod4	8	11	45.6	22	0	23.65	65524000	60749000	65266000	10427000	Glod4	541	1.078602117	0.996062511	1.074355133	67201	Glod4
Q9CPW4	Arpc5	2	3	25.2	5	0	5.6905	19523000	3784400	6808800	2980000	Arpc5	936	5.158809851	0.348757875	1.799175563	67771	Arpc5
Q9CPX7	Mrps16	1	1	5.9	2	0.0033822	1.6021	2038800	5122100	3812300	1429000	Mrps16	1185	0.398039866	1.869874436	0.744284571	66242	Mrps16
Q9CPY7-2;Q9CPY7	Lap3	3	9	22.5	15	0	17.045	32416000	33510000	37245000	4270800	Lap3	807	0.967353029	1.148969645	1.111459266	66988	Lap3
Q9CQ09;E9PWZ3	Rpl31	2	2	15.4	3	0	4.2308	5320700	8698600	7177400	1368700	Rpl31	1199	0.611673143	1.348957844	0.825121284	66211	Rpl31
Q9CQ19	Myl9	1	1	34.3	2	0	11.244	4989200	1325500	2625500	993590	Myl9	1292	3.76401358	0.526236671	1.980761977	98932	Myl9
Q9CQ54;A0A0U1RPJ5	Ndufc2	2	8	41.7	25	0	42.213	306580000	560560000	401760000	186050000	Ndufc2	120	0.546917368	1.310457303	0.71671186	68197	Ndufc2
Q9CQ60;F6X8L5;Q8CBG6	Pgls	4	5	22.2	12	0	7.4272	36154000	34275000	45659000	10259000	Pgls	545	1.054821298	1.262903137	1.332137126	66171	Pgls
Q9CQ62	Decr1	1	12	40	56	0	123.55	611060000	1335500000	791200000	175240000	Decr1	125	0.457551479	1.294799201	0.592437289	67460	Decr1
Q9CQ69	Uqcrq	2	6	54.9	34	0	58.735	656470000	1207000000	804560000	530810000	Uqcrq	48	0.543885667	1.225585328	0.666578293	22272	Uqcrq
Q9CQ73	Pkp2	1	20	28.2	36	0	47.657	214440000	198790000	277410000	15465000	Pkp2	442	1.078726294	1.293648573	1.395492731	67451	Pkp2
Q9CQ75	Ndufa2	1	7	57.6	28	0	41.553	558950000	677030000	628110000	300110000	Ndufa2	82	0.825591185	1.123731998	0.927743231	17991	Ndufa2
Q9CQ91;D3Z670	Ndufa3	2	3	50	10	0	6.5127	85507000	138920000	123700000	98437000	Ndufa3	187	0.615512525	1.446665185	0.890440541	66091	Ndufa3
Q9CQA3	Sdhb	1	14	49.3	59	0	57.678	798720000	1191500000	869070000	176220000	Sdhb	124	0.6703483	1.088078425	0.729391523	67680	Sdhb
Q9CQB4;Q9D855	Uqcrb	2	9	50.5	55	0	82.241	690530000	1154800000	932750000	539120000	Uqcrb	46	0.597965016	1.350774043	0.807715622	67530	Uqcrb
Q9CQB5;D3Z3X4	Cisd2	2	2	21.5	4	0	3.7258	7188400	5889800	7342700	2324000	Cisd2	1019	1.220482869	1.021465138	1.246680702	67006	Cisd2
Q9CQC7	Ndufb4	1	7	47.3	23	0	30.748	165550000	259510000	183000000	145300000	Ndufb4	145	0.637933028	1.105406222	0.705175138	100042503	Ndufb4
Q9CQD1	Rab5a	1	1	20.5	2	0	3.6665	3308800	3743700	3443200	1061900	Rab5a	1279	0.883831504	1.040618956	0.919731816	271457	Rab5a

Q9CQH3; D3Z568;F6Y6V5;D3YX99;D3Z6W9	Ndufb5	5	6	28	16	0	16.272	278500000	536730000	325650000	148550000	Ndufb5	140.5	0.518882865	1.16929982	0.606729641	66046	Ndufb5
Q9CQI6;A0A1D5RLP1	Cotl1	2	3	29.6	4	0	3.4299	28713000	2439600	3269900	3834800	Cotl1	842	11.76955239	0.113882214	1.340342679	72042	Cotl1
Q9CQJ8	Ndufb9	1	6	37.4	19	0	14.994	182290000	147410000	195550000	68413000	Ndufb9	229	1.236618954	1.072741236	1.326572146	66218	Ndufb9
Q9CQM5	Txndc17	1	3	26.8	5	0	4.8168	17724000	11804000	15076000	6685300	Txndc17	668	1.501524907	0.850598059	1.277194171	52700	Txndc17
Q9CQM8;O09167	Rpl21	2	2	13.8	2	0	3.1765	10272000	8085200	12141000	5205400	Rpl21	743	1.2704695	1.181950935	1.501632613	19933	Rpl21
Q9CQN1	Trap1	1	13	20.7	26	0	38.114	62103000	86200000	64446000	5972900	Trap1	696	0.720452436	1.037727646	0.747633411	68015	Trap1
Q9CQN6	Tmem14c	1	1	9.6	4	0	4.5006	10802000	6994600	7246400	5403000	Tmem14c	729	1.544334201	0.670838734	1.035999199	66154	Tmem14c
Q9CQQ7; A0A0G2JGX3	Atp5f1	2	15	41	70	0	94.677	1518300000	2232800000	1743700000	370100000	Atp5f1	73	0.679998209	1.148455509	0.780947689	11950	Atp5pb
Q9CQR2	Rps21	1	2	22.9	4	0	2.8284	37641000	19529000	25080000	17149000	Rps21	415	1.927441241	0.666294732	1.284243945	66481	Rps21
Q9CQR4	Acot13	1	3	21.4	17	0	8.1848	259730000	421210000	285230000	158870000	Acot13	131	0.616628285	1.098178878	0.677168158	66834	Acot13
Q9CQU3	Rer1	1	1	5.6	1	0.000601	1.9602	3147500	973370	1403600	687580	Rer1	1385	3.233611063	0.445941223	1.442000473	67830	Rer1
Q9CQV1	Pam16	1	1	10.4	1	0.0049806	1.5148	2079600	2556800	3160800	1225400	Pam16	1244	0.813360451	1.519907675	1.236232791	66449	Pam16
Q9CQV8-2;Q9CQV8;A2A5N1	Ywhab	3	4	40.6	10	0	13.048	61428000	29622000	39366000	9568600	Ywhab	570	2.073728985	0.640847822	1.328944703	54401	Ywhab
Q9CQW1	Ykt6	1	2	8.6	3	0.0005952	1.9051	8252300	5825100	7657200	1446200	Ykt6	1180	1.416679542	0.927886771	1.314518206	56418	Ykt6
Q9CQX8; Q9D6T9	Mrps36	2	4	70.6	9	0	13.847	17849000	28702000	24885000	19959000	Mrps36	386	0.62187304	1.394195753	0.867012752	66128	Mrps36
Q9CQZ5	Ndufa6	3	6	33.6	19	0	11.878	231700000	350150000	246160000	119940000	Ndufa6	167	0.661716407	1.062408287	0.703012994	67130	Ndufa6
Q9CQZ6	Ndufb3	1	3	33.7	16	0	23.405	67786000	134590000	95789000	64505000	Ndufb3	234	0.503648117	1.413108902	0.711709637	66495	Ndufb3
Q9CR00	Psmd9	1	2	8.1	2	0.0044346	1.5228	1702900	1797900	2600400	539100	Psmd9	1430	0.947160576	1.527042105	1.44635408	67151	Psmd9
Q9CR13		1	1	13.3	1	0	2.6832	968120	2874900	1114000	1231700	Q9CR13	1239	0.336749104	1.1506838	0.387491739	66117	Fmc1
Q9CR24	Nudt8	1	2	16.2	3	0	5.2145	2323500	3502100	3717400	936960	Nudt8	1311	0.663459067	1.599913923	1.061477399	66387	Nudt8
Q9CR51	Atp6v1g1	1	1	9.3	3	0	4.2996	2996700	1337300	3483400	1261900	Atp6v1g1	1225	2.240858446	1.162411987	2.604800718	66290	Atp6v1g1
Q9CR57;A0A1L1SUF6	Rpl14	2	4	21.7	7	0	11.828	62801000	40618000	56522000	33450000	Rpl14	301	1.546137181	0.900017516	1.391550544	67115	Rpl14
Q9CR59	Gadd45gip1	1	2	7.7	4	0	3.1608	9497700	8878600	10557000	2591600	Gadd45gip1	986	1.069729462	1.111532266	1.189038812	102060	Gadd45gip1
Q9CR61	Ndufb7	1	7	46	39	0	26.065	89817000	182490000	93942000	57001000	Ndufb7	248	0.492174914	1.045926718	0.514778892	66916	Ndufb7
Q9CR62;Q5SX46	Slc25a11	3	14	48.1	58	0	234.83	847030000	1405500000	1097700000	228710000	Slc25a11	97	0.60265386	1.295939931	0.781003202	67863	Slc25a11
Q9CR68	Uqcrfs1	1	12	38.3	58	0	49.035	1048700000	2036000000	1145100000	353720000	Uqcrfs1	76	0.515078585	1.091923334	0.562426326	66694	Uqcrfs1
Q9CR76	Tmem186	1	1	5.6	2	0	3.5217	4612100	6490400	6485400	1771700	Tmem186	1105	0.710603353	1.406170725	0.999229631	66690	Tmem186
Q9CRA7	Atp5s	1	2	10	3	0	2.3704	3419500	4308600	4309600	1207800	Atp5s	1246	0.793645268	1.260301214	1.000232094	68055	Dmac2l
Q9CRB8	Mtfp1	1	4	27.7	8	0	10.308	43559000	88797000	50193000	24732000	Mtfp1	350	0.490545852	1.15229918	0.565255583	67900	Mtfp1
Q9CRB9; Q9D9P1;D3Z0L4	Chchd3	5	9	35.2	24	0	32.606	411260000	540670000	418550000	124510000	Chchd3	163	0.760648825	1.017726013	0.774132095	66075	Chchd3
Q9CRD0;A0A0J9YTV7;A0A0J9YUK7;A0A0J9YUB6;Q9CRD0-3;Q9CRD0-2	Ociad1	6	2	10.1	4	0	4.8924	15259000	18867000	18005000	4868100	Ociad1	764	0.80876663	1.179959368	0.954311761	68095	Ociad1
Q9CRD2	Emc2	1	2	7.4	3	0	3.0798	3943300	1928500	2900500	579190	Emc2	1417	2.044749806	0.735551442	1.504018667	66736	Emc2
Q9CVB6; D3YXG6	Arpc2	3	10	31.7	14	0	21.484	68617000	21900000	37618000	6396100	Arpc2	679	3.133196347	0.548231488	1.717716895	76709	Arpc2
Q9CWF2	Tubb2b	1	1	40.4	9	0	7.4838	31997000	14028000	17280000	3222900	Tubb2b	900	2.280938124	0.54005063	1.23182207	73710	Tubb2b
Q9CWJ9	Atic	1	8	16.6	10	0	16.93	17968000	14288000	16924000	1556000	Atic	1152	1.257558791	0.941896705	1.184490482	108147	Atic
Q9CWK8	Snx2	1	3	10.8	9	0	8.997	20602000	9904300	10811000	1557100	Snx2	1151	2.08010662	0.524754878	1.091546096	67804	Snx2
Q9CWU3;Q6P1F6;F6RVI7;Q925E7	Ppp2r2a;Ppp2r2d	4	2	6	4	0	3.5547	3992500	5211200	3947700	742970	Ppp2r2a	1365	0.766138317	0.988778961	0.757541449	71978	Ppp2r2a;Ppp2r2d
Q9CX33;Q9CZR8;D3Z4M7	Tsfm	3	2	14	5	0	5.8778	17914000	19766000	21898000	7193400	Tsfm	647	0.906303754	1.222395891	1.107861985	66399	Tsfm
Q9CX80	Cygb	1	3	16.3	3	0	4.0879	16958000	943320	2708900	1483400	Cygb	1171	17.97693254	0.159741715	2.871666031	114886	Cygb
Q9CXD6-2;Q9CXD6	Mcur1	2	1	3.8	2	0	3.1279	2289800	2751500	2398500	439460	Mcur1	1469	0.832200618	1.047471395	0.871706342	76137	Mcur1
Q9CXI0	Coq5	1	3	11	6	0	5.5815	13523000	16715000	15478000	3069200	Coq5	922	0.809033802	1.144568513	0.925994616	52064	Coq5
Q9CXJ4	Abcb8	2	12	16.6	18	0	20.155	40034000	75071000	46514000	4943000	Abcb8	760	0.533281827	1.161862417	0.619600112	74610	Abcb8
Q9CXT8	Pmpcb	1	4	10.6	9	0	8.443	17037000	18249000	23319000	2600800	Pmpcb	982	0.933585402	1.368726889	1.277823442	73078	Pmpcb
Q9CXV1	Sdhd	1	2	10.7	8	0	2.699	122670000	187040000	173240000	85727000	Sdhd	203	0.655849016	1.412244232	0.926218991	66925	Sdhd
Q9CXW2	Mrps22	1	3	8.9	2	0	4.2155	8286500	9261200	8538300	1171700	Mrps22	1257	0.894754459	1.030386774	0.921943161	64655	Mrps22
Q9CXW3;A0A0A6YY29	Cacybp	2	2	8.7	2	0	4.1437	4850300	3629800	3493900	1079500	Cacybp	1275	1.336244421	0.720347195	0.962559921	12301	Cacybp
Q9CXW4;A2BH06	Rpl11	2	3	18.5	6	0	11.203	37230000	22759000	33696000	9693300	Rpl11	566	1.635836372	0.905076551	1.480557142	67025	Rpl11
Q9CY64	Blvra	1	5	18.6	6	0	7.8265	15520000	9393800	15554000	2266600	Blvra	1027	1.652153548	1.002190722	1.655772957	109778	Blvra
Q9CYT6; D3YTR7;A0A286YCS6	Cap2	3	4	9.5	4	0	6.4392	8261800	7317200	8940600	897380	Cap2	1324	1.129093096	1.082161272	1.221860821	67252	Cap2
Q9CZ13	Uqcrc1	4	17	44.2	116	0	170.89	3090700000	5274100000	3790400000	642340000	Uqcrc1	37	0.586014675	1.226388844	0.71868186	22273	Uqcrc1
Q9CZ30	Ola1	1	3	13.1	7	0	9.6672	21556000	20208000	22983000	2628800	Ola1	975	1.066706255	1.066199666	1.137321853	67059	Ola1
Q9CZ44;Q9CZ44-3;A2AT02;Q9CZ44-2	Nsfl1c	4	10	34.6	19	0	27.005	34086000	29844000	29211000	4587300	Nsfl1c	780	1.142139123	0.856979405	0.978789706	386649	Nsfl1c
Q9CZB0; F8WGB3	Sdhc	3	5	29	22	0	14.297	151610000	296100000	228620000	89839000	Sdhc	195	0.512022965	1.507948025	0.772104019	66052	Sdhc
Q9CZD3	Gars	1	7	9.2	7	0	6.7432	23961000	16094000	21856000	1530900	Gars	1158	1.488815708	0.912148909	1.358021623	353172	Gars1
Q9CZJ2	Hspa12b	1	3	4.7	4	0	2.8927	5561800	11084000	8062600	733040	Hspa12b	1367	0.501786359	1.449638606	0.727408878	72630	Hspa12b
Q9CZM2;B8JKK2	Rpl15	2	4	18.1	9	0	6.7139	58569000	42444000	57274000	14684000	Rpl15	455	1.379912355	0.977889327	1.349401564	66480	Rpl15
Q9CZN7-2;Q9CZN7	Shmt2	5	6	14	6	0	9.4403	12925000	18185000	17095000	1842500	Shmt2	1092	0.710750619	1.322630561	0.940060489	108037	Shmt2
Q9CZP5	Bcs1l	1	2	4.5	2	0	2.4772	4978600	4922700	8455900	764180	Bcs1l	1360	1.011355557	1.698449363	1.717736202	66821	Bcs1l
Q9CZU6	Cs	2	18	34.7	102	0	76.349	3384200000	5098300000	4101800000	706060000	Cs	33	0.663789891	1.212044205	0.804542691	12974	Cs
Q9CZW5	Tomm70a	1	3	4.6	5	0	7.7239	16635000	11723000	15162000	1561800	Tomm70a	1150	1.419005374	0.911451758	1.293354943	28185	Tomm70
Q9CZX8;D3YUT3;D3YUG3;D3Z5R8;D3Z722;S4R223	Rps19	6	6	36.6	13	0	13.584	107410000	71418000	101430000	32251000	Rps19	308	1.503962586	0.944325482	1.420230194	20085	Rps19
Q9D020-1;Q9D020	Nt5c3a	3	9	33.7	15	0	20.854	20224000	46775000	31779000	5019700	Nt5c3a	753	0.432367718	1.57135087	0.67940139	107569	Nt5c3a
Q9D023	Mpc2	5	6	42.5	16	0	17.313	230460000	235640000	369160000	115070000	Mpc2	170	0.978017315	1.601839799	1.566627058	70456	Mpc2
Q9D031;Q01730;A2AUR7	Rsu1	4	6	25.3	9	0	9.958	17958000	14783000	14615000	3819300	Rsu1	844	1.214773727	0.813843412	0.988635595	20163	Rsu1
Q9D051	Pdhb	1	11	38.7	78	0	154.04	896750000	1311500000	1049400000	184570000	Pdhb	122	0.683759055	1.170225815	0.800152497	68263	Pdhb
Q9D0F3	Lman1	1	1	2.1	1	0.0006105	2.062	6614200	4359700	4623600	855150	Lman1	1336	1.517122738	0.699041456	1.060531688	70361	Lman1
Q9D0F9;A2CEK3	Pgm1;Pgm2	2	21	42.7	60	0	57.951	478420000	662830000	559190000	57041000	Pgm1	247	0.721783866	1.168826554	0.843640149	72157	Pgm1
Q9D0G0	Mrps30	1	2	4.8	4	0	4.2315	4844800	4758600	6235600	736200	Mrps30	1366	1.018114572	1.287070674	1.310385407	59054	Mrps30
Q9D0I9	Rars	1	9	14.8	13	0	12.873	22824000	13887000	18309000	1528500	Rars	1159	1.643551523	0.802181914	1.318427306	104458	Rars1
Q9D0J8	Ptms	1	2	22.8	5	0	4.7617	28418000	7574100	11542000	15097000	Ptms	453	3.751996937	0.406151031	1.523877424	69202	Ptms
Q9D0K2; Q3UJQ9	Oxct1	2	12	24.6	42	0	62.136	522880000	646250000	663950000	90871000	Oxct1	194	0.809098646	1.269794217	1.027388781	67041	Oxct1
Q9D0L4;Q9D0L4-2	Adck1	2	2	4.8	4	0	3.0765	2009700	2775100	2967100	327190	Adck1	1508	0.724190119	1.476389511	1.069186696	72113	Adck1

Q9D0M3-2;Q9D0M3;A0A2R8VHK1	Cyc1	3	11	39.5	71	0	69.76	538710000	1090300000	606180000	254140000	Cyc1	87	0.494093369	1.125243638	0.55597542	66445	Cyc1
Q9D0M5;P63168;Q80ZS7	Dynll2;Dynll1;BC048507	3	2	24.7	6	0	5.0065	8213300	12162000	6304200	7734500	Dynll2	632	0.675324782	0.767559933	0.518352245	68097	BC048507;Dynll1;Dynll2
Q9D0S9	Hint2	1	4	32.5	9	0	9.3843	47578000	62817000	58175000	17711000	Hint2	411	0.757406435	1.222728992	0.926102807	68917	Hint2
Q9D172;A0A1W2P7B6; A0A1W2P870	D10Jhu81e	3	10	46.6	30	0	55.499	711130000	1287600000	804640000	247190000	D10Jhu81e	89	0.552291084	1.131494945	0.62491457	28295	Gatd3a;Gatd3
Q9D1A2;A0A494B9S3	Cndp2	3	4	11.4	4	0	9.0294	22288000	8413900	10394000	1754100	Cndp2	1110	2.648949952	0.466349605	1.235336764	66054	Cndp2
Q9D1D4; A0A1Y7VM54	Tmed10	3	3	14.6	7	0	15.929	33919000	18905000	17376000	8566400	Tmed10	605	1.794181433	0.512279254	0.919121925	68581	Tmed10
Q9D1E6	Tbcb	1	3	11.9	4	0	2.5656	6889200	6356600	8677900	1313900	Tbcb	1211	1.08378693	1.259638274	1.365179498	66411	Tbcb
Q9D1G3	Hhatl	3	5	10.1	11	0	12.349	38643000	76898000	47426000	9355400	Hhatl	578	0.502522822	1.227285666	0.616739057	74770	Hhatl
Q9D1H6	Ndufaf4	1	2	13.9	4	0	11.556	5536000	9161500	7416700	2255600	Ndufaf4	1028	0.60426786	1.339721821	0.809550838	68493	Ndufaf4
Q9D1I5	Mcee	1	1	10.7	3	0	6.8583	2155800	5959100	2854100	1363500	Mcee	1201	0.361766038	1.323916875	0.478948163	73724	Mcee
Q9D1I6	Mrpl14	1	2	11.7	2	0.008	1.3339	4251300	2886000	4274600	1474800	Mrpl14	1174	1.473076923	1.005480677	1.481150381	68463	Mrpl14
Q9D1L9;G3UW70	Lamtor5	2	2	37.4	2	0	5.4734	3078500	1582100	2341800	1721400	Lamtor5	1115	1.94583149	0.760695144	1.480184565	68576	Lamtor5
Q9D1R9;A0A0G2JGY8;A0A0G2JEY6	Rpl34	3	4	23.1	7	0	7.6779	57613000	19929000	36255000	19040000	Rpl34	399	2.89091274	0.629285057	1.819208189	619547	Rpl34
Q9D1X0	Nol3	1	3	16.8	5	0	5.7173	11002000	10781000	14949000	3494400	Nol3	872	1.020499026	1.358752954	1.386606066	78688	Nol3
Q9D2G2;Q9D2G2-2	Dlst	2	9	23.6	44	0	49.988	493010000	1013400000	588120000	103440000	Dlst	180	0.48649102	1.192916979	0.580343398	78920	Dlst
Q9D2R6;E9Q6W2	Coa3	2	2	18.5	3	0	4.9523	10237000	13223000	10063000	6154300	Coa3	686	0.774181351	0.983002833	0.761022461	52469	Coa3
Q9D3B1	Hacd2	1	1	3.9	2	0.0006101	2.0582	11200000	8875400	12629000	3108000	Hacd2	917	1.261914956	1.127589286	1.422921784	70757	Hacd2
Q9D3D9	Atp5d	2	3	22.6	28	0	27.463	742470000	1252600000	816560000	639730000	Atp5d	38	0.592743094	1.099788544	0.651892065	66043	Atp5f1d
Q9D404	Oxsm	1	2	6.3	3	0	4.3183	3300200	4398600	3436700	634290	Oxsm	1398	0.750284181	1.04136113	0.781316783	71147	Oxsm
Q9D517	Agpat3	3	4	10.1	5	0	6.3092	10166000	14962000	14576000	2791700	Agpat3	953	0.679454618	1.433798938	0.97420131	28169	Agpat3
Q9D5T0;A0A494BAB5;A0A494B9L7;A0A494B9Y0	Atad1	4	5	14.1	4	0	8.1503	7967300	20236000	11704000	2525900	Atad1	997	0.393719114	1.469004556	0.578375173	67979	Atad1
Q9D6J5;Q3V406	Ndufb8	5	5	33.9	22	0	24.893	105850000	152870000	110270000	43011000	Ndufb8	272	0.692418395	1.041757204	0.721331851	67264	Ndufb8
Q9D6J6;Q9D6J6-2	Ndufv2	4	11	54	49	0	77.414	245120000	401940000	232610000	71446000	Ndufv2	222	0.609842265	0.948963773	0.578718217	72900	Ndufv2
Q9D6K8	Fundc2	1	4	22.5	4	0	5.4922	25549000	40308000	27675000	15253000	Fundc2	450	0.633844398	1.08321265	0.68658827	67391	Fundc2
Q9D6M3;E9PV90;E9PY45;E9Q6M6;A0A0J9YTY6; Q9DB41-2;Q9DB41	Slc25a22;Slc25a18	12	5	16.1	6	0	9.1386	6515700	14655000	7857000	2196600	Slc25a22	1031	0.444605937	1.205856623	0.536131013	68267	Slc25a22;Slc25a18
Q9D6S7	Mrrf	1	2	8.8	2	0	2.8091	10971000	14052000	13205000	3460600	Mrrf	874	0.780742955	1.203627746	0.939723883	67871	Mrrf
Q9D6U8	Fam162a	2	6	28.4	11	0	14.561	146110000	140850000	144190000	47432000	Fam162a	266	1.037344693	0.986859216	1.02371317	70186	Fam162a
Q9D6Y7-2;Q9D6Y7;A0A1B0GT40;Q9D6Y7-4;Q9D6Y7-3	Msra	5	5	31.9	10	0	10.522	17591000	20314000	22450000	4816600	Msra	768	0.865954514	1.276220795	1.105149158	110265	Msra
Q9D710;D3Z2J6	Tmx2	2	2	5.8	3	0	3.8269	11850000	8742500	9090500	1851600	Tmx2	1089	1.355447526	0.767130802	1.039805548	66958	Tmx2
Q9D7J4	Cox20	1	2	22.2	7	0	11.144	17337000	31504000	16509000	15751000	Cox20	436	0.550311072	0.952240872	0.524028695	66359	Cox20
Q9D7J9	Echdc3	1	2	7.3	3	0	2.5861	5095500	9580700	5517300	1703400	Echdc3	1122	0.531850491	1.082778923	0.575876502	67856	Echdc3
Q9D7N9	Apmap	1	1	2.9	1	0	3.2121	4773400	3809900	4671000	616960	Apmap	1404	1.252893777	0.978547786	1.226016431	71881	Apmap
Q9D7X8	Ggct	1	1	6.9	2	0.0049779	1.5148	2039300	2174300	3434000	719080	Ggct	1375	0.937911052	1.683911146	1.579358874	110175	Ggct
Q9D819	Ppa1	1	3	13.5	6	0	7.3916	14213000	13574000	15403000	2544700	Ppa1	992	1.047075291	1.083726166	1.134742891	67895	Ppa1
Q9D832	Dnajb4	3	4	14.5	8	0	6.2338	10687000	5796900	14324000	1562600	Dnajb4	1149	1.843571564	1.340320015	2.470975866	67035	Dnajb4
Q9D880	Timm50	1	5	17.8	11	0	20.703	25688000	29962000	29003000	5759600	Timm50	710	0.857352647	1.129048583	0.967992791	66525	Timm50
Q9D881;P19536	Cox5b	4	6	38	65	0	19.711	638870000	1184500000	856860000	401960000	Cox5b	67	0.539358379	1.341211827	0.723393837	12859	Cox5b;Cox5b-ps
Q9D898	Arpc5l	1	2	16.3	3	0	8.2001	12875000	6512000	9711100	3217900	Arpc5l	901	1.977119165	0.754260194	1.491262285	74192	Arpc5l
Q9D8E6	Rpl4	1	13	27.9	21	0	27.43	122070000	64496000	91565000	18400000	Rpl4	406	1.892675515	0.7501024	1.419700447	67891	Rpl4
Q9D8N0	Eef1g	1	12	26.8	25	0	35.348	152830000	97697000	164740000	17738000	Eef1g	410	1.564326438	1.077929726	1.686233968	67160	Eef1g
Q9D8U8	Snx5	1	2	8.4	3	0	5.3466	8217100	2064300	2317500	539340	Snx5	1429	3.980574529	0.282033808	1.122656591	69178	Snx5
Q9D8Y1	Tmem126a	2	3	14.8	4	0	2.4948	15157000	17956000	19235000	6731900	Tmem126a	667	0.844118957	1.269050604	1.071229673	66271	Tmem126a
Q9DAK9	Phpt1	1	4	40.3	5	0	7.718	25954000	32963000	31894000	11321000	Phpt1	519	0.787367655	1.228866456	0.967569699	75454	Phpt1
Q9DB05	Napa	1	4	14.6	5	0	6.1294	9516200	8486800	8849300	1493800	Napa	1168	1.121294245	0.929919506	1.04271339	108124	Napa
Q9DB15	Mrpl12	1	2	12.4	2	0	3.2275	6078300	6236700	5898400	2763900	Mrpl12	955	0.974601953	0.970402909	0.94575657	56282	Mrpl12
Q9DB20;A0A338P776	Atp5o	5	11	61	49	0	85.958	2029400000	3130500000	2065900000	700500000	Atp5o	34	0.64826705	1.017985612	0.659926529	28080	Atp5o;Atp5po
Q9DB60	Fam213b	2	5	24.4	11	0	11.448	49006000	44944000	64755000	12913000	Fam213b	492	1.090379138	1.321368812	1.440792987	66469	Prxl2b
Q9DB77	Uqcrc2	2	20	46.1	127	0	166.82	2161800000	3901800000	2911000000	547820000	Uqcrc2	45	0.554051976	1.346563049	0.746065918	67003	Uqcrc2
Q9DBC7;A2AI69	Prkar1a	7	7	15.5	15	0	17.274	36874000	31462000	44174000	6038600	Prkar1a	692	1.172017036	1.19797147	1.404042972	19084	Prkar1a
Q9DBG3;Q9DBG3-2;H3BIY9;H3BKM0	Ap2b1	5	4	12.3	13	0	18.049	59852000	27174000	33244000	2758200	Ap2b1	958	2.202546552	0.555436744	1.223375285	71770	Ap2b1
Q9DBG5	Plin3	2	7	24.3	14	0	17.049	24120000	24385000	23305000	3231600	Plin3	898	0.989132664	0.966210614	0.955710478	66905	Plin3
Q9DBG9	Tax1bp3	1	1	13.7	2	0.0064865	1.3788	2852100	859510	1848200	1103600	Tax1bp3	1270	3.318286	0.648013744	2.150294935	76281	Tax1bp3
Q9DBH5	Lman2	1	4	11.2	7	0	10.908	21338000	7956600	10108000	2043700	Lman2	1056	2.681798758	0.473708876	1.270391876	66890	Lman2
Q9DBJ1	Pgam1	1	8	55.9	20	0	32.394	54896000	41119000	51350000	11299000	Pgam1	521	1.335051922	0.93540513	1.248814417	18648	Pgam1
Q9DBL1; E9Q5L3	Acadsb	3	10	27.1	20	0	20.575	65113000	89427000	70591000	13586000	Acadsb	477	0.728113433	1.084130665	0.789370101	66885	Acadsb
Q9DBS1	Tmem43	1	2	5	3	0.0005967	1.922	9586100	1503600	2759400	582840	Tmem43	1415	6.375432296	0.287854289	1.835195531	74122	Tmem43
Q9DC61;A2AIW9	Pmpca	2	8	17.6	11	0	14.639	31899000	40625000	32751000	3701000	Pmpca	857	0.785206154	1.026709301	0.806178462	66865	Pmpca
Q9DC70	Ndufs7	1	7	22.8	19	0	48.646	267290000	418700000	310280000	100900000	Ndufs7	182	0.638380702	1.160836545	0.741055648	75406	Ndufs7
Q9DC77	Smpx	1	2	38.8	5	0	7.8704	15009000	15691000	21653000	11457000	Smpx	516	0.956535594	1.442667733	1.379963036	66106	Smpx
Q9DCC4	Pycrl	1	2	8.8	3	0	2.7295	2842500	1720400	1890800	538890	Pycrl	1432	1.652232039	0.665189094	1.099046733	66194	Pycr3
Q9DCC5;P23198;D3Z313	Cbx3	3	2	12.6	5	0	2.6442	8569900	3725200	5339900	2592500	Cbx3	985	2.300520777	0.623099453	1.433453237	12417	Cbx3
Q9DCD0	Pgd	1	6	16.1	13	0	15.92	28433000	15958000	19507000	3160700	Pgd	911	1.781739566	0.686069004	1.22239629	110208	Pgd
Q9DCH4	Eif3f	1	3	10.2	5	0	4.0476	19591000	11725000	16033000	3215900	Eif3f	903	1.6708742	0.818385994	1.367420043	66085	Eif3f
Q9DCJ5	Ndufa8	1	10	61.6	46	0	76.276	437600000	706320000	593860000	220640000	Ndufa8	100	0.619549213	1.357084095	0.840780383	68375	Ndufa8
Q9DCL9	Paics	3	7	18.4	11	0	13.479	32172000	31242000	30440000	5229600	Paics	742	1.029767621	0.946164367	0.974329428	67054	Paics
Q9DCM0	Ethe1	1	5	22	8	0	9.7629	22386000	27088000	28019000	6059600	Ethe1	690	0.826417602	1.251630483	1.034369462	66071	Ethe1
Q9DCM2;A0A0N4SVE5	Gstk1	2	8	44.2	22	0	47.606	48621000	121880000	58800000	17686000	Gstk1	412	0.398925172	1.209353983	0.482441746	76263	Gstk1
Q9DCN2-2;Q9DCN2;F2Z456	Cyb5r3	3	8	35.6	19	0	27.21	89844000	37491000	60609000	13890000	Cyb5r3	471	2.39641514	0.674602645	1.616627991	109754	Cyb5r3
Q9DCS3	Mecr	1	2	6.7	3	0	2.6828	7409200	10162000	5395200	1651300	Mecr	1133	0.729108443	0.728175781	0.53091911	26922	Mecr
Q9DCS9; D3YUK4	Ndufb10	2	9	55.7	39	0	129.81	421560000	696130000	538980000	191840000	Ndufb10	115	0.605576545	1.278536863	0.774251936	68342	Ndufb10
Q9DCT2	Ndufs3	1	11	40.7	42	0	121.86	489700000	805610000	636020000	137260000	Ndufs3	151	0.607862365	1.298795181	0.78948871	68349	Ndufs3
Q9DCT8;A0A0G2JF37	Crip2	2	7	45.7	15	0	53.434	62033000	93064000	45464000	24810000	Crip2	349	0.666562795	0.732900231	0.488524026	68337	Crip2
Q9DCV4;A2AGJ2	Rmdn1	2	6	20.7	12	0	14.496	46054000	75263000	63008000	10839000	Rmdn1	533	0.611907577	1.368133061	0.837170987	66302	Rmdn1

Q9DCW4;A0A0U1RNP5;A0A0N4SVE0;A0A0U1RNR3;A0A0N4SWE9;A0A0U1RNK9	Etfb	7	15	56.9	120	0	142.23	1778100000	4464700000	2080100000	779100000	Etfb	30	0.398257442	1.169844216	0.465899165	110826	Etfb; Etfbl
Q9DCX2; B1ASE2	Atp5h	2	14	64	80	0	100.07	1093200000	2468900000	1684800000	558110000	Atp5h	44	0.442788286	1.541163557	0.68240917	71679	Atp5h;Atp5pd
Q9DCZ1;F6VY18	Gmpr	3	6	18.8	9	0	7.537	11623000	17683000	14351000	3332100	Gmpr	883	0.65729797	1.234707046	0.811570435	66355	Gmpr
Q9DCZ4-2;Q9DCZ4;Q9DCZ4-3	Apoo	3	4	26.2	15	0	21.901	92169000	176330000	113240000	56304000	Apoo	251	0.522707424	1.228612657	0.642204957	68316	Apoo
Q9EQ06;Q9EQ06-2	Hsd17b11	2	2	6.4	4	0	5.2648	7151400	5905200	3698000	1065600	Hsd17b11	1278	1.211034343	0.517101547	0.626227731	114664	Hsd17b11
Q9EQ20	Aldh6a1	2	17	32.3	38	0	90.129	216540000	453840000	292850000	39513000	Aldh6a1	280	0.477128503	1.352406022	0.645271461	104776	Aldh6a1
Q9EQH2	Erap1	1	1	1.4	2	0	2.7462	2040500	781740	1884600	96003	Erap1	1563	2.610202881	0.923597158	2.410775961	80898	Erap1
Q9EQH3	Vps35	1	11	17.1	22	0	27.778	63784000	51984000	53612000	4378400	Vps35	796	1.226992921	0.840524269	1.031317328	65114	Vps35
Q9EQI8	Mrpl46	1	2	8.5	3	0	4.1671	4738200	5608600	3798100	958250	Mrpl46	1302	0.844809756	0.801591322	0.677192169	67308	Mrpl46
Q9EQP2	Ehd4	1	15	40.9	48	0	111.33	236810000	178490000	309590000	24828000	Ehd4	348	1.326740994	1.307334994	1.73449493	98878	Ehd4
Q9ERD7	Tubb3	1	0	27.3	1	0.0034091	1.6381	4602400	2868100	5072200	622240	Tubb3	1403	1.604686029	1.102077177	1.768487849	22152	Tubb3
Q9ERI6	Rdh14	1	3	9.9	2	0	3.2548	4553600	6089400	4141200	816380	Rdh14	1343	0.747791244	0.909434294	0.680067002	105014	Rdh14
Q9ERS2	Ndufa13	1	5	38.9	21	0	18.113	347790000	548550000	396840000	184860000	Ndufa13	121	0.634016954	1.141033382	0.723434509	67184	Ndufa13
Q9ES97-3;Q9ES97-4;Q9ES97-5;Q9ES97-2;Q9ES97	Rtn3	5	2	11	6	0	6.2926	19213000	24810000	23998000	13192000	Rtn3	486	0.774405482	1.249050122	0.967271262	20168	Rtn3
Q9ESL4-2;A2ASW6;Q9ESL4-3;Q9ESL4	Zak	4	2	5.9	3	0.0033632	1.5903	2922300	2563600	3002500	715630	Zak	1376	1.139920424	1.027444136	1.171204556	65964	Map3k20
Q9ET54-3;Q9ET54-5;Q9ET54-4;Q9ET54-6;Q9ET54;Q9ET54-2	Palld	7	5	9	10	0	9.2212	35183000	15095000	26882000	2648600	Palld	973	2.330771779	0.764062189	1.780854588	72333	Palld
Q9ET78	Jph2	1	6	10.5	11	0	20.624	18049000	26692000	23282000	2998100	Jph2	932.5	0.676195115	1.28993296	0.872246366	59091	Jph2
Q9JHI5	Ivd	1	14	31.1	38	0	78.362	417150000	805310000	524260000	92909000	Ivd	192	0.51799928	1.256766151	0.651003961	56357	Ivd
Q9JHU4	Dync1h1	2	54	12.7	85	0	104.57	425600000	282300000	403400000	4401900	Dync1h1	793	1.507616011	0.947838346	1.428976266	13424	Dync1h1
Q9JHW2;A0A338P6G0;A0A338P7A1	Nit2	4	7	31.9	10	0	15.993	10622000	18652000	14946000	3297000	Nit2	889	0.569483165	1.407079646	0.801308171	52633	Nit2
Q9JI39	Abcb10	1	4	6.6	5	0	6.1769	3462900	7823600	5265600	538730	Abcb10	1433	0.442622322	1.52057524	0.673040544	56199	Abcb10
Q9JI91	Actn2	3	41	64.5	401	0	323.31	5149000000	6369500000	7249800000	372400000	Actn2	72	0.808383704	1.408001554	1.138205511	11472	Actn2
Q9JIF9	Myot	1	9	19.4	15	0	18.952	18894000	28467000	44784000	3610500	Myot	863	0.663715882	2.370276278	1.573190009	58916	Myot
Q9JII6;B1AXW3	Akr1a1	2	6	22.2	9	0	24.938	41875000	18339000	25286000	3773200	Akr1a1	850	2.283385136	0.603844776	1.378810186	58810	Akr1a1
Q9JIK9	Mrps34	1	2	6.9	4	0.0017483	1.7531	5483800	8637000	7383600	1889900	Mrps34	1083	0.634919532	1.346438601	0.854880167	79044	Mrps34
Q9JIQ3	Diablo	1	2	5.9	2	0.010016	1.2859	10499000	8511500	15495000	2595900	Diablo	984	1.233507607	1.475854843	1.820478177	66593	Diablo
Q9JJ28	Flii	1	1	0.7	1	0.0028571	1.6694	2080800	788890	1372200	65475	Flii	1568	2.637630088	0.659457901	1.739406001	14248	Flii
Q9JJI8	Rpl38	1	2	32.9	3	0	6.3237	7714400	8571300	25043000	13594000	Rpl38	476	0.900026834	3.246266722	2.921727159	67671	Rpl38
Q9JJW5	Myoz2	2	9	36.4	31	0	78.461	385920000	588520000	505910000	115450000	Myoz2	169	0.655746619	1.310919362	0.859630939	59006	Myoz2
Q9JJZ2	Tuba8	1	3	34.7	4	0	6.4822	24619000	31662000	29341000	4244900	Tuba8	810	0.777556693	1.191803079	0.92669446	53857	Tuba8
Q9JK42	Pdk2	1	10	32.9	18	0	32.96	63269000	117700000	82545000	15874000	Pdk2	434	0.537544605	1.304667373	0.701316907	18604	Pdk2
Q9JKB1;A0A2I3BQ39;P58321	Uchl3;Uchl4	3	4	24.8	9	0	9.4808	17588000	20134000	20650000	4864100	Uchl3	765	0.873547234	1.174095975	1.02562829	50933	Uchl3;Uchl4
Q9JKF1	Iqgap1	3	10	6.8	11	0	20.734	47493000	4044400	7893100	684090	Iqgap1	1387	11.74290377	0.166195018	1.951612106	29875	Iqgap1
Q9JKL4;A0A0A6YVW1	Ndufaf3	2	2	10.3	4	0	3.0051	6258000	12016000	9268800	4004100	Ndufaf3	830	0.520805593	1.481112176	0.771371505	66706	Ndufaf3
Q9JKR6	Hyou1	2	4	4.8	4	0	8.8803	11644000	4776000	8643000	539080	Hyou1	1431	2.438023451	0.742270697	1.809673367	12282	Hyou1
Q9JKS4-3;Q9JKS4;E9PYJ9;Q9JKS4-4;Q9JKS4-2	Ldb3	5	10	42.1	97	0	121.87	752520000	949340000	986260000	102730000	Q9JKS4-3	181	0.792677018	1.310609685	1.038890176	24131	Ldb3
Q9JKS4-5;Q9JKS4-6	Ldb3	2	1	50.2	5	0	3.2916	1769300	5320300	3110800	805320	Q9JKS4-5	1346	0.332556435	1.758209461	0.58470387		
Q9JLV1	Bag3	1	13	26.2	26	0	42.178	81225000	94292000	121840000	8707700	Bag3	596	0.861419845	1.500030779	1.29215628	29810	Bag3
Q9JM76;A0A0G2JFK7;D3Z2F7;D3Z2F8;H7BWZ3	Arpc3	5	2	11.2	2	0.0005893	1.8317	9431900	1825800	3005700	1438800	Arpc3	1184	5.16589988	0.318673862	1.646237266	56378	Arpc3
Q9JMC3	Dnaja4	2	5	14.1	10	0	14.524	18030000	28907000	44433000	4456400	Dnaja4	790	0.623724357	2.464392679	1.53710174	58233	Dnaja4
Q9JMH6-2;Q9JMH6	Txnrd1	2	3	10.2	6	0	7.6074	8846000	7237600	6049800	969550	Txnrd1	1299	1.222228363	0.683902329	0.835884824	50493	Txnrd1
Q9QUI0;A0A0A6YXF6; A0A0G2JEP8;H3BL56;Q9CR99	Rhoa;Rhoc;4930544G11Rik	7	2	35.8	13	0	29.008	66012000	43819000	37339000	19667000	Rhoa	390	1.506469796	0.565639581	0.852118944	11848	Rhoa;Rhoc;4930544G11Rik
Q9QUM9;E0CXB1	Psma6	3	6	28.9	8	0	20.101	23722000	16167000	18400000	4009600	Psma6	829	1.467309952	0.775651294	1.138120863	26443	Psma6
Q9QUR6	Prep	1	2	4.5	4	0	15.817	10422000	9420200	9025400	682480	Prep	1388	1.106345937	0.865995011	0.958090062	19072	Prep
Q9QXS1-15;Q9QXS1-10;Q9QXS1-9;Q9QXS1-12;Q9QXS1-11;Q9QXS1-13;Q9QXS1-5;Q9QXS1-3;Q9QXS1-16;Q9QXS1-4;Q9QXS1-14;Q9QXS1-6;Q9QXS1-7;Q9QXS1-8;Q9QXS1-2;Q9QXS1;E9Q3W4	Plec	25	121	28.7	213	0	254.67	4168200000	3083900000	3622800000	40842000	Plec	276	1.351600246	0.869152152	1.174746263	18810	Plec
Q9QXX4	Slc25a13	1	17	33.3	45	0	55.806	251670000	378350000	310900000	33164000	Slc25a13	304	0.665177745	1.235347876	0.821725915	50799	Slc25a13
Q9QYA2	Tomm40	1	4	13.6	7	0	9.2482	22704000	26545000	19603000	7205000	Tomm40	646	0.855302317	0.863416138	0.738481823	53333	Tomm40
Q9QYB1	Clic4	1	8	32.4	15	0	22.873	44850000	32591000	44424000	8551700	Clic4	606	1.376146789	0.990501672	1.363075696	29876	Clic4
Q9QYG0;Q9QYG0-2;A0A2I3BPW0;A0A2I3BQR5;A0A2I3BQM5;A0A2I3BPL1	Ndrg2	7	8	29.6	29	0	41.256	188160000	266980000	199690000	45045000	Ndrg2	270	0.704771893	1.061277636	0.747958649	29811	Ndrg2
Q9QYJ0	Dnaja2	1	6	15.5	10	0	11.503	24429000	27618000	26515000	4324300	Dnaja2	800	0.884531827	1.085390315	0.960062278	56445	Dnaja2
Q9QYR9;O55137	Acot2;Acot1	10	10	25.6	26	0	33.217	218710000	565930000	291730000	55931000	Acot2	253	0.386461223	1.333866764	0.515487781	171210	Acot1;Acot2
Q9QZ06;Q8C5G6;A9JEI5;F7AT44	Tollip	4	4	15.3	4	0	5.7481	4975700	14381000	1343200	2404500	Tollip	1012	0.345991238	0.269951967	0.093401015	54473	Tollip
Q9QZD9	Eif3i	1	2	6.8	2	0	3.8418	5133100	2402200	3998500	594800	Eif3i	1412	2.136832903	0.778963979	1.66451586	54709	Eif3i
Q9QZF2	Gpc1	1	2	4.1	2	0	3.8237	2916200	2941700	3749100	367260	Gpc1	1490	0.991331543	1.285611412	1.274467145	14733	Gpc1
Q9QZQ8-2;Q9QZQ8	H2afy	3	5	20.9	7	0	9.1494	28816000	5701400	9419800	2637700	H2afy	974	5.054197215	0.326894781	1.65219069	26914	Macroh2a1
Q9R062;V9GX26;K3W4S6	Gyg1;Gyg	3	4	17.4	9	0	43.371	30104000	26916000	29743000	8529300	Gyg1	607	1.118442562	0.988008238	1.105030465	27357	Gyg; Gyg1

Q9R069;D3YTK7	Bcam	2	4	8.5	8	0	7.694	11734000	11112000	8028500	1080000	Bcam	1274	1.055975522	0.684208284	0.722507199	57278	Bcam
Q9R0H0-2;Q9R0H0;A2A848	Acox1	3	9	17.5	9	0	24.543	11243000	37544000	25970000	2836700	Acox1	950	0.299461965	2.309881704	0.691721713	11430	Acox1
Q9R0P5	Dstn	1	7	33.9	14	0	25.623	103370000	41148000	66171000	19827000	Dstn	388	2.512151259	0.640137371	1.608121901	56431	Dstn
Q9R0P9	Uchl1	1	5	24.7	9	0	9.718	50960000	12711000	26658000	8735800	Uchl1	595	4.009125954	0.52311617	2.097238612	22223	Uchl1
Q9R0Q6	Arpc1a	1	1	2.7	3	0.000625	2.246	5126900	4444200	7280100	848030	Arpc1a	1339	1.153615949	1.419980885	1.638112596	56443	Arpc1a
Q9R0X4;Q32MW3	Acot9;Acot10	2	9	19.8	14	0	14.313	34614000	22327000	36754000	3078100	Acot9	920	1.55032024	1.061824695	1.646168316	56360	Acot10;Acot9
Q9R0Y5;Q9R0Y5-2	Ak1	4	12	43.3	36	0	63.153	696180000	1427000000	821400000	326680000	Ak1	78	0.487862649	1.179867276	0.575613174	11636	Ak1
Q9R112;F6ZKZ3	Sqrdl	3	5	14.7	6	0	16.137	21686000	26375000	24029000	2575200	Sqrdl	989	0.822218009	1.108042055	0.911052133	59010	Sqor
Q9R1P0;E9PW69;E9Q0X0	Psma4	3	4	16.5	9	0	7.4548	40545000	21323000	30524000	6932100	Psma4	659	1.901467899	0.752842521	1.431505886	26441	Psma4
Q9R1P1	Psmb3	1	2	11.2	4	0	2.8955	23288000	21905000	21325000	7793900	Psmb3	631	1.06313627	0.915707661	0.973522027	26446	Psmb3
Q9R1P4;A0A1B0GS70	Psma1	2	6	24.3	9	0	11.492	22738000	14510000	19989000	3422600	Psma1	876	1.567057202	0.879101064	1.377601654	26440	Psma1
Q9R1Q7	Plp2	1	1	7.9	6	0	6.3148	7360500	2126800	7593900	3216100	Plp2	902	3.460833177	1.031709802	3.570575513	18824	Plp2
2;Q9WTI7-3;Q9WTI7;Q9WTI7-4	Myo1c	4	9	10.7	12	0	19.283	35743000	22518000	27347000	1696200	Myo1c	1123	1.587307931	0.765100859	1.214450662	17913	Myo1c
Q9WTM5;A0A1B0GSR4	Ruvbl2	5	4	9.7	2	0	5.6312	8313200	3800400	4850500	628350	Ruvbl2	1401	2.187453952	0.583469663	1.27631302	20174	Ruvbl2
Q9WTP6-2;Q9WTP6;F7BP55	Ak2	3	4	24.6	7	0	12.411	24721000	35617000	31356000	7183700	Ak2	648	0.69407867	1.268395291	0.880366117	11637	Ak2
Q9WTP7	Ak3	2	10	52.9	23	0	26.768	66100000	90733000	72899000	16173000	Ak3	428	0.728511126	1.102859304	0.803445273	56248	Ak3
Q9WTR5	Cdh13	1	5	7.7	8	0	24.825	51589000	59293000	59401000	5878700	Cdh13	705	0.870068979	1.15142763	1.001821463	12554	Cdh13
Q9WTX5	Skp1	1	2	15.3	4	0	7.3676	10779000	7069900	8488800	2841500	Skp1	948	1.524632597	0.787531311	1.200695908	21402	Skp1
Q9WU78;Q9WU78-3	Pdcd6ip	2	9	12.2	15	0	27.899	35080000	21676000	31794000	2088800	Pdcd6ip	1046	1.618379775	0.906328392	1.466783539	18571	Pdcd6ip
Q9WUA2;A0A087WS80	Farsb	3	3	5.8	2	0	2.7419	3593400	1688600	2571200	279430	Farsb	1523	2.128035059	0.715534035	1.522681511	23874	Farsb
Q9WUB3;E9PUM3	Pygm	4	41	58.8	174	0	246.67	1459900000	2502300000	1528000000	124810000	Pygm	162	0.583423251	1.046647031	0.610638213	19309	Pygm
Q9WUK2-2;Q9WUK2	Eif4h	2	4	16.7	4	0	6.6411	22483000	13095000	14717000	4595600	Eif4h	778	1.716914853	0.654583463	1.12386407	22384	Eif4h
Q9WUM3;A0A494BAI1;D3YUG6;A0A494B9Y4	Coro1b	4	3	6	4	0	5.9508	10599000	2513600	3969400	1046600	Coro1b	1282	4.216661362	0.374507029	1.579169319	23789	Coro1b
Q9WUM4	Coro1c	4	6	14.3	8	0	8.7155	22758000	3228500	9471500	1791800	Coro1c	1101	7.049094007	0.41618332	2.933715348	23790	Coro1c
Q9WUM5	Suclg1	2	8	32.9	42	0	51.978	149950000	362820000	218370000	60431000	Suclg1	240	0.413290337	1.456285428	0.601868695	56451	Suclg1
Q9WUR9;A2ARF6;F6TEU8	Ak4	4	6	32.7	10	0	12.088	20020000	30229000	22899000	7051200	Ak4	653	0.662277945	1.143806194	0.757517616	11639	Ak4
Q9WUU7	Ctsz	1	4	12.4	4	0	5.2159	33910000	5505500	6526600	3580600	Ctsz	868	6.15929525	0.192468298	1.185469076	64138	Ctsz
Q9WV35	Apobec2	1	2	6.7	4	0.0080386	1.3479	45729000	34227000	40841000	13260000	Apobec2	483	1.336050486	0.893109405	1.193239256	11811	Apobec2
Q9WV55;A0A3B2W837	Vapa	2	3	21.7	4	0	3.1634	3214000	4450100	3888300	748070	Vapa	1364	0.722230961	1.209800871	0.873755646	30960	Vapa
Q9WV85	Nme3	1	1	5.9	2	0	2.3949	2839700	2591700	3579500	947610	Nme3	1306	1.095690088	1.260520478	1.381139792	79059	Nme3
Q9WV98;A0A1Y7VL11;A0A1Y7VIZ6	Timm9	3	4	56.2	7	0	7.5482	12482000	24317000	16708000	10779000	Timm9	535	0.51330345	1.338567537	0.687091335	30056	Timm9
Q9WVA2;Q4FZG7	Timm8a1; Timm8a2	2	2	22.7	4	0	13.96	21829000	28188000	23136000	16604000	Timm8a1	421	0.774407549	1.059874479	0.820774798	30058	Timm8a1; Timm8a2
Q9WVA4	Tagln2	3	12	62.8	26	0	47.783	333710000	66022000	145970000	37894000	Tagln2	285	5.054527279	0.43741572	2.21092969	21346	Tagln2
Q9WVE8;Q3UP40;A0A2R8W6S4	Pacsin2	6	8	15.6	10	0	14.347	38531000	37823000	45421000	5594100	Pacsin2	721	1.018718769	1.178817056	1.200883061	23970	Pacsin2
Q9WVJ2;E9Q5I9;E9Q0U1	Psmd13	6	8	21	10	0	13.652	36466000	29195000	31522000	4020600	Psmd13	828	1.249049495	0.864421653	1.079705429	23997	Psmd13
Q9WVK4	Ehd1	1	7	32.6	30	0	35.766	76400000	90714000	84187000	9743200	Ehd1	564	0.842207377	1.101924084	0.928048592	13660	Ehd1
Q9WVQ5	Apip	1	2	13.3	3	0	4.0853	3244500	4004100	4000000	675160	Apip	1390	0.810294448	1.232855602	0.99897605	56369	Apip
Q9Z0N1;A2AAW9;Q9Z0N2	Eif2s3x;Eif2s3y	3	4	8.7	4	0	4.101	4709200	3078200	2800500	477980	Eif2s3x	1453	1.52985511	0.594686996	0.909784939	26905	Eif2s3x;Eif2s3y
Q9Z0P5-2;Q9Z0P5;A0A1L1STC8; A0A1L1SU53	Twf2	5	4	19.3	10	0	7.3399	18026000	14066000	17807000	2614500	Twf2	980	1.28152993	0.987850882	1.265960472	23999	Twf2
Q9Z1D1	Eif3g	1	4	14.1	6	0	5.1836	7066700	3960100	4116300	698770	Eif3g	1380	1.78447514	0.582492535	1.039443448	53356	Eif3g
Q9Z1J3	Nfs1	3	7	15.3	9	0	10.79	13420000	16003000	15858000	1844600	Nfs1	1090	0.838592764	1.181669151	0.990939199	18041	Nfs1
Q9Z1N5;Q8VDW0;Q8VDW0-2	Ddx39b;Ddx39a	5	4	9.6	7	0	6.5951	28368000	16924000	14222000	3389100	Ddx39b	881	1.67619948	0.501339538	0.840345072	53817	Ddx39a;Ddx39b
Q9Z1P6	Ndufa7	1	6	55.8	23	0	13.504	233870000	304060000	273340000	152240000	Ndufa7	139	0.769157403	1.168768974	0.898967309	66416	Ndufa7
Q9Z1Q5	Clic1	1	4	23.2	5	0	8.4517	17874000	3032400	10493000	2018400	Clic1	1062	5.894341116	0.587053821	3.460295476	114584	Clic1
Q9Z1Q9;G3UY93	Vars	4	4	4	6	0	6.1784	11866000	3782900	5732200	353690	Vars	1496	3.13674694	0.483077701	1.5152925	22321	Vars;Vars1
Q9Z1Z2	Strap	1	4	12	5	0	5.4007	9969300	4320600	3353500	952150	Strap	1303	2.307387863	0.336382695	0.776165347	20901	Strap
Q9Z204-4;Q9Z204-3;Q9Z204-2;Q9Z204-5;Q9Z204	Hnrnpc	7	5	20.5	5	0	9.3673	39360000	7376100	14922000	3611600	Hnrnpc	862	5.336153252	0.379115854	2.023020295	15381	Hnrnpc
Q9Z2I0	Letm1	2	15	23.8	31	0	85.005	103870000	167490000	133600000	10832000	Letm1	534	0.620156427	1.286223164	0.797659562	56384	Letm1
Q9Z2I8-2;Q9Z2I8	Suclg2	3	12	34.9	23	0	65.829	91999000	195740000	137180000	19974000	Suclg2	385	0.470006131	1.491103164	0.700827628	20917	Suclg2
Q9Z2I9	Sucla2	1	23	47.9	75	0	141.8	1436600000	2469000000	1753000000	235560000	Sucla2	92	0.581855002	1.220242239	0.71000405	20916	Sucla2
Q9Z2M7	Pmm2	1	2	10.3	4	0	3.5259	2041200	1979800	2619900	439390	Pmm2	1470	1.031013234	1.2835097	1.323315486	54128	Pmm2
Q9Z2U0;A0A338P7D7;Q9CWH6	Psma7;Psma8	4	5	25.4	11	0	13.952	39518000	26947000	38462000	7875500	Psma7	628	1.466508331	0.973278	1.427320295	26444	Psma7;Psma8
Q9Z2U1	Psma5	2	4	19.1	6	0	5.8172	19935000	10894000	15407000	4528300	Psma5	784	1.82990637	0.772861801	1.414264733	26442	Psma5
Q9Z2Z6	Slc25a20	1	13	39.5	36	0	37.367	292680000	553630000	363110000	83981000	Slc25a20	205	0.528656323	1.24063824	0.65587125	57279	Slc25a20
S4R1E5;O70325-2;O70325;O70325-3	Gpx4	4	5	34.4	9	0	9.814	29094000	27159000	29859000	9798000	Gpx4	562	1.0712471	1.026294081	1.099414559	625249	Gpx4
S4R1R2;D3Z5H6;D3Z0T0;Q62311	Taf6	4	1	14	2	0.0006211	2.1678	4202700	16011000	8078000	5047500	Taf6	747	0.262488289	1.922097699	0.504528137	21343	Taf6
S4R2F3;Q8C8R3;Q8C8R3-2;Q8C8R3-4;S4R2R5;Q8C8R3-5;Q8C8R3-3	Ank2	15	4	1.3	5	0	5.8395	5883400	8049300	7336600	112470	Ank2	1561	0.730920701	1.247000034	0.911458139	109676	Ank2
V9GXA0; V9GXX2; V9GWW6;V9GXP3	Mlip	10	4	5.5	7	0	6.3076	225510000	257680000	300890000	23625000	Mlip	354	0.875155231	1.334264556	1.167688606		Mlip
Z4YJE4;E9QQ96;A2AAJ9;A0A1W2P6H1;H7BX05	Obscn	8	69	20.8	158	0	174.93	697470000	814340000	932260000	6742400	Obscn	666	0.856485006	1.336630966	1.144804381	380698	Obscn
Z4YJF5;Q62234-2;Q62234	Myom1	4	76	55.4	194	0	319.15	1489100000	1914100000	2183500000	62556000	Myom1	236	0.777963534	1.466321939	1.140744998	17929	Myom1
Z4YK85;M0QWP1;A2ASQ1-3;A2ASQ1-2;A2ASQ1	Agrn	5	3	2.1	2	0	6.0137	4560900	2458900	4945400	124820	Agrn	1559	1.854853796	1.084303537	2.011224531	11603	Agrn
Z4YKM2;B2RWE3;B1AR13	Cisd3	3	3	21.7	3	0	3.2999	18187000	26313000	36526000	11062000	Cisd3	527	0.691179265	2.008357618	1.388135142	217149	Cisd3

Z4YKV1; P63094-2;P63094; Q6R0H7-4;Q6R0H7;A0A571BEM2;A0A571BEI3;P63094-3	Gnas	9	3	12.1	5	0	4.9237	17450000	13554000	12195000	2457900	Gnas	1004	1.287442821	0.698853868	0.899734396	14683	Gnas
