# Supplementary material for: Comparative Proteomic and Phosphoproteomic Analyses Reveal Molecular Signatures of Myocardial Infarction and Transverse Aortic Constriction in Aged Mouse Models
Source: Cardiol Res Pract. 2024 Oct 28;2024:9395213. doi: 10.1155/2024/9395213 (PMC11535427; doi:10.1155/2024/9395213)
Supplement: Supporting Information — Table S2: List of significant differentially expressed proteins (DEPs) in MI vs sham. [file 9395213.f2.pdf]

ENTREZID	Majority.protein.IDs	Gene.names	Number.of.proteins	Unique.peptides	Sequence.coverage....	MS.MS.count	Q.value	Score	LFQ.intensity.MI	LFQ.intensity.sham	iBAQ	Symbol	fold change(MI/sham)	Alias	preferred_name	protein_size	annotation	SubPPI	STRING_id
	A0A075B5P4;A	Ighg1	4	2	6.8	1	0.001	1.9768	13717000	5121100	1953600	Ighg1	2.678526098	Ighg1					
	A0A338P6I9		1	2	39	2	0	3.4208	938970	2250000	1284300	A0A338P6	0.41732	Gm56451					
	A0A5H1ZRK8;P01837		2	2	23.4	4	0	4.5266	34176000	5829200	11730000	A0A5H1Z	5.862897139	Igkc					
	F6RPJ9;Q9JHR7	Ide	2	7	7.6	9	0	6.4472	15060000	5054400	540200	Ide	2.979582146	Ide					
	F7CVJ5;F7DBB	Ahnak2	3	4	20.3	5	0	10.214	32664000	2377100	625270	Ahnak2	13.74111312						
	P0DN34		1	2	31.6	8	0	4.9085	182400000	416800000	322000000	P0DN34	0.437619962	Ndufb1					
	P20065-2		1	2	31.8	9	0	9.908	136280000	37746000	122460000	P20065-2	3.610448789						
100503670	P47962;D3YYV8	Rpl5	2	4	15.2	5	0	9.6812	14125000	5629600	2710700	Rpl5	2.509059258	Rpl5	Rpl5	297	60S ribosomal pro	2	10090.ENSMUSP00000080854
102866	A0A1C7CYV0;I	Pls3	4	10	24.3	12	0	37.061	68257000	11221000	2983200	Pls3	6.082969432	Pls3	Pls3	630	Plastin-3; Actin-bi	1	10090.ENSMUSP00000033547
103963	Q91YQ5	Rpn1	2	14	23.5	20	0	27.317	91392000	29251000	4797400	Rpn1	3.124406003	Rpn1	Rpn1	608	Dolichyl-diphosph	2	10090.ENSMUSP00000032143
104130	O09111	Ndufb11	1	4	45	11	0	17.767	121820000	245850000	78024000	Ndufb11	0.495505389	Ndufb11	Ndufb11	151	NADH dehydroge	3	10090.ENSMUSP00000112320
104158	Q8VCT4	Ces1d	1	3	11	4	0	5.3547	5350300	18081000	1327300	Ces1d	0.295907306	Ces1d	Ces1d	565	Carboxylesterase	3	10090.ENSMUSP00000034172
104776	Q9EQ20	Aldh6a1	2	17	32.3	38	0	90.129	216540000	453840000	39513000	Aldh6a1	0.477128503	Aldh6a1	Aldh6a1	535	Methylmalonate-s	3	10090.ENSMUSP00000082288
106393	Q7TQ48;Q7TQ4	Srl	2	25	36.5	93	0	171.13	697930000	1459000000	94507000	Srl	0.478361892	Srl	Srl	910	Sarcalumenin; Ma	4	10090.ENSMUSP00000023161
107227	Q922B1	Macrod1	1	5	20.7	11	0	25.49	94373000	222010000	37293000	Macrod1	0.425084456	Macrod1	Macrod1	323	O-acetyl-ADP-ribo	18	10090.ENSMUSP00000039507
107569	Q9D020-1;Q9D0	Nt5c3a	3	9	33.7	15	0	20.854	20224000	46775000	5019700	Nt5c3a	0.432367718	Nt5c3a	Nt5c3	331	Cytosolic 5'-nucle	4	10090.ENSMUSP00000031793
108075	D3Z598;Q8K4G	Ltbp4	7	6	5.1	6	0	9.5114	20566000	4547100	404280	Ltbp4	4.522882716	Ltbp4	Ltbp4	1666	Latent-transformin	6	10090.ENSMUSP00000037536
109006	Q8WTY4;E0CY	Ciapin1	3	3	11.3	6	0	4.5228	3356500	6752800	949040	Ciapin1	0.497053074	Ciapin1	Ciapin1	309	Anamorsin; Comp	7	10090.ENSMUSP00000125451
109136	Q8C7H1	Mmaa	1	2	6	2	0.003	1.7313	1793900	3947100	416280	Mmaa	0.454485572	Mmaa	Mmaa	415	Methylmalonic ac	3	10090.ENSMUSP00000048826
109552	Q6P069-2;Q6P0	Sri	2	4	24	4	0	7.8175	14399000	4712600	1956200	Sri	3.05542588	Sri	Sri	198	Sorcin; Calcium-bi	4	10090.ENSMUSP00000118221
109624	Q8VCQ8;S4R1T	Cald1	11	9	19.8	13	0	21.44	53675000	4742900	2969100	Cald1	11.31691581	Cald1	Cald1	530	Caldesmon 1	1	10090.ENSMUSP00000110673
109711	A1BN54;Q7TPR	Actn1	2	15	40.4	29	0	80.779	285180000	76530000	9543600	Actn1	3.726381811	Actn1	Actn1	892	Alpha-actinin-1; F	1	10090.ENSMUSP00000021554
109754	Q9DCN2-2;Q9D	Cyb5r3	3	8	35.6	19	0	27.21	89844000	37491000	13890000	Cyb5r3	2.39641514	Cyb5r3	Cyb5r3	301	NADH-cytochrom	12	10090.ENSMUSP00000018186
109905	A0A0G2JDL9;A	Rap1a;Rap1b	5	4	41.5	10	0	7.8338	122770000	50846000	32705000	Rap1a	2.414545884	Rap1a;Rap1b	Rap1a	184	Ras-related protei	1	10090.ENSMUSP00000088174
109979	F6RER8;E9QNU	Art3	16	4	13.2	8	0	6.1786	15166000	32952000	5003300	Art3	0.460245205	Art3	Art3	408	ADP-ribosyltrans	25	10090.ENSMUSP00000113493
110826	Q9DCW4;A0A	Etfb	7	15	56.9	120	0	142.23	1778100000	4464700000	779100000	Etfb	0.398257442	Etfb;Etfbl	Etfb	255	Electron transfer f	3	10090.ENSMUSP00000004729
110854	P58389	Ppp2r4	1	2	5.3	4	0	3.4596	22503000	10040000	3011900	Ppp2r4	2.241334661	Ptpa	Ppp2r4	323	Serine/threonine-p	5	10090.ENSMUSP00000046837
11364	P45952	Acadm	2	20	53	86	0	105.1	1987900000	4025200000	496890000	Acadm	0.493863659	Acadm	Acadm	421	Medium-chain spe	3	10090.ENSMUSP00000072483
11370	P50544;B1AR28	Acadvl	2	36	57.3	151	0	192.62	1547500000	3148900000	196580000	Acadvl	0.491441456	Acadvl	Acadvl	656	Very long-chain sp	3	10090.ENSMUSP00000099634
11409	Q07417	Acads	1	14	40.3	52	0	62.381	362760000	761410000	92695000	Acads	0.476431883	Acads	Acads	412	Short-chain specif	3	10090.ENSMUSP00000031524
11421	P09470;P09470-	Ace	3	6	4.9	8	0	12.166	30209000	7776300	932060	Ace	3.884752389	Ace	Ace	1312	Angiotensin-conv	1	10090.ENSMUSP00000001963
11430	Q9R0H0-2;Q9R	Acox1	3	9	17.5	9	0	24.543	11243000	37544000	2836700	Acox1	0.299461965	Acox1	Acox1	661	Peroxisomal acyl-c	3	10090.ENSMUSP00000063325
114584	Q9Z1Q5	Clic1	1	4	23.2	5	0	8.4517	17874000	3032400	2018400	Clic1	5.894341116	Clic1	Clic1	241	Chloride intracellu	5	10090.ENSMUSP00000007257
11459	P68134	Acta1	1	2	70	11	0	80.414	42810000	16316000	8601100	Acta1	2.623804854	Acta1	Acta1	377	Actin, alpha skelet	1	10090.ENSMUSP00000034453
11461	P60710;P63260;I	Actb;Actg1	14	7	57.1	61	0	228.95	1506600000	319050000	125240000	Actb	4.722143865	Actb;Actg1	Actb	375	Actin, cytoplasmic	1	10090.ENSMUSP00000098066
114886	Q9CX80	Cygb	1	3	16.3	3	0	4.0879	16958000	943320	1483400	Cygb	17.97693254	Cygb	Cygb	190	Cytoglobin; May	4	10090.ENSMUSP00000021166
11636	Q9R0Y5;Q9R0Y	Ak1	4	12	43.3	36	0	63.153	696180000	1427000000	326680000	Ak1	0.487862649	Ak1	Ak1	210	Adenylate kinase	4	10090.ENSMUSP00000068479
11668	P24549	Aldh1a1	3	6	15.4	6	0	17.178	43051000	6092700	2376000	Aldh1a1	7.065997013	Aldh1a1	Aldh1a1	501	Retinal dehydroge	1	10090.ENSMUSP00000084918
11745	O35639;Q3TET	Anxa3	4	6	20.4	12	0	16.745	86317000	19630000	7022800	Anxa3	4.397198166	Anxa3	Anxa3	323	Annexin A3; Inhib	1	10090.ENSMUSP00000031447
11746	A0A0N4SW89;	Anxa4	6	7	30.4	12	0	23.324	52510000	14737000	6018900	Anxa4	3.563140395	Anxa4	Anxa4	319	Annexin A4; Calci	6	10090.ENSMUSP00000109305
11747	P48036	Anxa5	2	18	58.9	39	0	61.568	1001400000	351980000	84576000	Anxa5	2.845048014	Anxa5	Anxa5	319	Annexin A5; This	1	10090.ENSMUSP00000029266
11757	P20108	Prdx3	1	6	26.8	22	0	18.322	267230000	635060000	87029000	Prdx3	0.420794886	Prdx3	Prdx3	257	Thioredoxin-deper	3	10090.ENSMUSP00000025961
11771	P17426-2;P1742	Ap2a1	5	6	10.5	11	0	12.153	44797000	16619000	1716100	Ap2a1	2.695529214	Ap2a1	Ap2a1	977	AP-2 complex sub	2	10090.ENSMUSP00000127842
11773	Q3TWV4;P8409	Ap2m1	3	5	13.2	5	0	6.8994	9496400	3871700	696690	Ap2m1	2.452772684	Ap2m1	Ap2m1	435	AP-2 complex sub	2	10090.ENSMUSP00000007216
11816	P08226;A0A1B	Apoe	4	6	19.3	11	0	11.272	39375000	10207000	4379900	Apoe	3.857646713	Apoe	Apoe	311	Apolipoprotein E;	6	10090.ENSMUSP00000133302
11843	P61750;F6UFB9	Arf4	3	3	23.9	3	0	6.2891	29101000	6141200	4562800	Arf4	4.738650427	Arf4	Arf4	180	ADP-ribosylation	2	10090.ENSMUSP00000022429
11857	Q61599;D3YWL	Arhgdib	2	3	25.5	4	0	16.001	18331000	1666300	2035800	Arhgdib	11.00102022	Arhgdib	Arhgdib	200	Rho GDP-dissocia	1	10090.ENSMUSP00000032344
11867	Q91Z25;Q9WV	Arpc1b	4	4	15.4	5	0	11.976	25578000	6496300	2224000	Arpc1b	3.937318166	Arpc1b	Arpc1b	372	Actin-related prot	1	10090.ENSMUSP00000082822
11949	Q8C2Q8;A2AK	Atp5c1	7	12	36.9	85	0	49.699	1595400000	3462400000	828570000	Atp5c1	0.460778651	Atp5c1;Atp5f1c	Atp5c1	298	ATP synthase sub	3	10090.ENSMUSP00000110547
11991	Q60668-3;Q6066	Hnrnpd	13	6	24.8	8	0	8.925	84884000	20379000	9104200	Hnrnpd	4.165268168	Hnrnpd	Hnrnpd	355	Heterogeneous nu	2	10090.ENSMUSP00000132735
12010	P01887	B2m	1	2	15.1	2	0	2.4736	42524000	13665000	9397300	B2m	3.111891694	B2m	B2m	119	Beta-2-microglobu	1	10090.ENSMUSP00000099534
12039	Q3U3J1;P50136	Bckdha	3	10	24.4	19	0	33.197	43573000	91448000	8506800	Bckdha	0.476478436	Bckdha	Bckdha	446	The branched-cha	3	10090.ENSMUSP00000071292
12040	Q6P3A8-2;Q6P3	Bckdhb	2	4	14.3	6	0	7.6017	11811000	31775000	6008300	Bckdhb	0.371707317	Bckdhb	Bckdhb	390	2-oxoisovalerate d	3	10090.ENSMUSP00000139684
12111	P28653	Bgn	1	13	46.3	31	0	55.991	828820000	33143000	57297000	Bgn	25.00739221	Bgn	Bgn	369	Biglycan; May be	6	10090.ENSMUSP00000033741
12282	Q9JKR6	Hyou1	2	4	4.8	4	0	8.8803	11644000	4776000	539080	Hyou1	2.438023451	Hyou1	Hyou1	999	Hypoxia up-regula	2	10090.ENSMUSP00000068594
12304	A0A0R4J0Z1;P	Pdia4	2	7	13.3	9	0	10.766	40198000	8712000	1912600	Pdia4	4.6140955	Pdia4	Pdia4	641	Protein disulfide-i	2	10090.ENSMUSP00000076521
12306	P07356;B0V2N7	Anxa2	4	18	54.3	41	0	130.03	505110000	137260000	39563000	Anxa2	3.679950459	Anxa2	Anxa2	339	Annexin A2; Calci	1	10090.ENSMUSP00000034756
12317	P14211	Calr	1	12	31.5	21	0	18.218	368000000	154160000	30405000	Calr	2.387130254	Calr	Calr	416	Calreticulin; Calciu	1	10090.ENSMUSP00000003912
12321	Q6XLQ8;O3588	Calu	8	5	16.2	7	0	24.662	34884000	3892300	3230000	Calu	8.962310202	Calu	Calu	315	Calumenin; Involv	2	10090.ENSMUSP00000031779

12331	P40124	Cap1	2	12	32.3	11	0	18.895	86182000	20540000	4966400	Cap1	4.195813048	Cap1	Cap1	474	Adenylyl cyclase-	1	10090.ENSMU SP00000101862
12336	A0A0R4J1C2;A	Capns1	3	2	14	3	0	5.9244	6835300	3378400	1467400	Capns1	2.023235851	Capns1	Capns1	268	Calpain small subu	1	10090.ENSMU SP00000001845
12340	Q5RKN9;P4775	Capza1	3	3	17.8	3	0	7.1661	14710000	3257500	1736700	Capza1	4.515732924	Capza1					
12406	P19324	Serpinh1	4	11	35.3	31	0	97.134	296940000	69581000	21399000	Serpinh1	4.267544301	Serpinh1	Serpinh1	417	Serpin H1; Binds	2	10090.ENSMU SP00000126390
12417	Q9DCC5;P2319	Cbx3	3	2	12.6	5	0	2.6442	8569900	3725200	2592500	Cbx3	2.300520777	Cbx3	Cbx3	183	Component of het	1	10090.ENSMU SP00000031862
12490	Q64314;Q64314-	Cd34	2	2	5.2	4	0	8.1363	19959000	4573500	2691100	Cd34	4.364053788	Cd34	Cd34	382	Hematopoietic pro	1	10090.ENSMU SP00000016638
12496	O55026;O55026-	Entpd2	2	2	5.3	2	0	10.154	18273000	1661200	1008500	Entpd2	10.99987961	Entpd2	Entpd2	495	Ectonucleoside tri	4	10090.ENSMU SP00000028328
12558	D3YYT0;P15116	Cdh2	2	6	10.2	9	0	23.277	9723400	28225000	2159700	Cdh2	0.344496014	Cdh2	Cdh2	906	Cadherin-2; Cadhe	1	10090.ENSMU SP00000025166
12631	P18760;F8WGL	Cfl1	3	9	59.6	20	0	45.569	376480000	160280000	82176000	Cfl1	2.348889443	Cfl1	Cfl1	227	Cofilin-1; Binds to	1	10090.ENSMU SP00000112259
12709	Q04447	Ckb	1	6	26.8	11	0	33.838	128260000	52141000	14222000	Ckb	2.459868434	Ckb	Ckb	381	Creatine kinase B-	3	10090.ENSMU SP00000001304
12715	P07310	Ckm	1	4	54.9	153	0	258.64	2908100000	5895400000	768090000	Ckm	0.493282899	Ckm	Ckm	381	Creatine kinase M	3	10090.ENSMU SP00000003643
12751	O89023	Tpp1	1	3	7.5	3	0	8.6247	12637000	4702700	1176100	Tpp1	2.687179705	Tpp1	Tpp1	562	Tripeptidyl-peptid	2	10090.ENSMU SP00000033184
12759	Q06890;E9PUU	Clu	6	11	27	17	0	35.443	132350000	28096000	11853000	Clu	4.710634966	Clu	Clu	448	Clusterin; Functio	2	10090.ENSMU SP00000022616
12785	P53996-2;P5399	Cnbp	4	3	21.8	5	0	7.2547	29282000	12284000	4998500	Cnbp	2.383751221	Cnbp	Cnbp	178	Cellular nucleic ac	2	10090.ENSMU SP00000032138
12819	A2AJY2;O3520	Col15a1	4	13	10.9	22	0	22.014	132480000	49363000	5975000	Col15a1	2.683791504	Col15a1	Col15a1	1367	Collagen alpha-1(	6	10090.ENSMU SP00000099981
12833	Q04857	Col6a1	1	15	16.9	40	0	75.159	511690000	209890000	19824000	Col6a1	2.437896041	Col6a1	Col6a1	1025	Collagen alpha-1(	6	10090.ENSMU SP00000001147
12834	Q02788;D3Z7D	Col6a2	2	16	16.6	37	0	38.875	355660000	143410000	13814000	Col6a2	2.480022314	Col6a2	Col6a2	1034	Collagen alpha-2(	6	10090.ENSMU SP00000001181
12835	J3QQ16;A0A08	Col6a3	2	2	22.9	112	0	243.48	1234100000	405650000	14059000	Col6a3	3.042277826	Col6a3	Col6a3	3284	Collagen, type VI,	6	10090.ENSMU SP00000057131
12842	P11087-2;P1108	Col1a1	3	6	5.7	8	0	17.202	127850000	4630400	2275600	Col1a1	27.61100553	Col1a1	Col1a1	1453	Collagen alpha-1(	1	10090.ENSMU SP00000001547
12843	Q01149	Col1a2	1	5	4.2	6	0	7.5175	116100000	6307800	1923100	Col1a2	18.40578332	Col1a2	Col1a2	1372	Collagen alpha-2(	6	10090.ENSMU SP00000031668
12847	F8WHL2;Q8CI	Copa	3	10	8.3	13	0	15.476	42709000	16473000	1238800	Copa	2.592666788	Copa	Copa	1224	Coatomer subunit	2	10090.ENSMU SP00000118179
12865	A0A140LIU4;P	Cox7a1	2	2	25.8	16	0	8.442	510360000	1073500000	538030000	Cox7a1	0.475416861	Cox7a1	Cox7a1	80	Cytochrome c oxi	3	10090.ENSMU SP00000096193
12896	P52825	Cpt2	4	24	40.9	57	0	79.463	322040000	670430000	49257000	Cpt2	0.480348433	Cpt2	Cpt2	658	Carnitine O-palmit	3	10090.ENSMU SP00000030345
12908	H7BX88;P47934	Crat	5	20	35.5	53	0	60.445	371850000	771460000	74443000	Crat	0.48200814	Crat	Crat	626	Carnitine O-acetyl	3	10090.ENSMU SP00000028207
12925	A0A0G2JEK2;P	Crip1	2	2	14.8	7	0	5.0055	56190000	12011000	17834000	Crip1	4.678211639	Crip1	Crip1	77	Cysteine-rich prot	3	10090.ENSMU SP00000006523
12934	O08553	Dpysl2	2	13	37.8	31	0	35.523	224060000	73863000	13523000	Dpysl2	3.033453827	Dpysl2	Dpysl2	572	Dihydropyrimidina	1	10090.ENSMU SP00000022629
13003	Q8BS97;E9QM	Vcan	9	3	10.9	4	0	4.3859	12537000	1204900	1022100	Vcan	10.40501286	Vcan	Vcan	3354	May play a role in	6	10090.ENSMU SP00000105173
13007	P97315	Csrp1	1	6	46.6	9	0	12.732	35434000	9459600	5298600	Csrp1	3.745824348	Csrp1	Csrp1	193	Cysteine and glyc	1	10090.ENSMU SP00000027677
13010	A2APX3;P2146	Cst3	2	2	26.5	2	0	3.0497	7190800	1088100	1719300	Cst3	6.60858377	Cst3	Cst3	140	Cystatin-C; As an	2	10090.ENSMU SP00000028938
13014	Q62426	Cstb	1	2	18.4	2	0.001	2.2762	14058000	5797500	4255500	Cstb	2.424838292	Cstb	Cstb	98	Cystatin-B; This i	2	10090.ENSMU SP00000005185
13135	P61804	Dad1	1	2	19.5	3	0	4.0942	7783400	3662500	3083600	Dad1	2.12516041	Dad1	Dad1	113	Dolichyl-diphosph	2	10090.ENSMU SP00000122366
13167	Q4VWZ5;P3178	Dbi	4	2	25.2	11	0	7.3939	28143000	57556000	20091000	Dbi	0.488967267	Dbi	Dbi	135	Acyl-CoA-binding	3	10090.ENSMU SP00000114705
13171	P53395	Dbt	2	12	22	18	0	24.319	58131000	143320000	11366000	Dbt	0.405602847	Dbt	Dbt	482	Dihydrolipoamide	3	10090.ENSMU SP00000000349
13177	P42125;A0A3Q	Eci1	4	10	39.1	53	0	46.799	427760000	1113200000	145960000	Eci1	0.384261588	Eci1	Eci1	289	Enoyl-CoA delta i	3	10090.ENSMU SP00000024946
13179	P28654	Dcn	1	8	22.6	19	0	70.036	222460000	28695000	15169000	Dcn	7.752570134	Dcn	Dcn	354	Decorin; May affe	6	10090.ENSMU SP00000100924
13200	O54734	Ddost	1	3	7.7	4	0	14.707	18108000	5274300	1284300	Ddost	3.433251806	Ddost	Ddost	441	Dolichyl-diphosph	2	10090.ENSMU SP00000030538
13207	Q8BTS0;Q6165	Ddx5	5	6	15.4	13	0	16.296	42233000	17884000	2685600	Ddx5	2.36149631	Ddx5	Ddx5	615	Involved in the alt	2	10090.ENSMU SP00000021062
13382	O08749	Dld	1	16	36.3	80	0	123.25	765410000	1620400000	189880000	Dld	0.472358677	Dld	Dld	509	Dihydrolipoyl deh	3	10090.ENSMU SP00000106481
13427	A2BFF8;Q3TPJ	Dync1i2	5	2	3.1	4	0	3.4521	25626000	10906000	3047600	Dync1i2	2.349715753	Dync1i2	Dync1i2	612	Cytoplasmic dyne	1	10090.ENSMU SP00000080410
13479	P31428;A0A1D	Dpep1	3	4	12.4	6	0	17.314	25576000	10033000	2118900	Dpep1	2.549187681	Dpep1	Dpep1	410	Dipeptidase 1; Hy	1	10090.ENSMU SP00000019422
13589	Q61166	Mapre1	1	2	9.7	2	0	2.4173	7031000	3162400	888350	Mapre1	2.223311409	Mapre1	Mapre1	268	Microtubule-asso	1	10090.ENSMU SP00000028981
13627	P10126	Eef1a1	3	6	30.5	16	0	23.492	211450000	64884000	16889000	Eef1a1	3.258892793	Eef1a1	Eef1a1	462	Elongation factor	2	10090.ENSMU SP00000042457
13849	E9PWK1;Q9D3	Ephx1	3	5	10	8	0	9.1202	42205000	9954600	2568200	Ephx1	4.239748458	Ephx1	Ephx1	455	Epoxide hydrolase	5	10090.ENSMU SP00000047551
140781	Q91Z83	Myh7	5	36	64	193	0	323.31	4277900000	401150000	105680000	Myh7	10.66409074	Myh7	Myh7	1935	Myosin-7; Myosi	1	10090.ENSMU SP00000099867
14086	Q61553;A0A0G	Fscn1	3	7	18.1	7	0	9.569	24648000	8659800	2126300	Fscn1	2.84625511	Fscn1	Fscn1	493	Fascin; Organizes	1	10090.ENSMU SP00000031565
14118	Q61554	Fbn1	2	58	23.6	119	0	144.93	1161500000	215030000	11885000	Fbn1	5.401571874	Fbn1	Fbn1	2873	Fibrillin-1; Fibrillin	6	10090.ENSMU SP00000028633
14151	F6T2S0;Q91W0	Fech	8	4	12.9	4	0	5.1602	4947800	22933000	2293400	Fech	0.215750229	Fech	Fech	422	Ferrochelatase, mi	7	10090.ENSMU SP00000025484
14161	E9PV24-2;E9PV	Fga	2	11	23.3	27	0	49.628	125500000	61036000	10675000	Fga	2.056163576	Fga	Fga	789	Fibrinogen alpha	1	10090.ENSMU SP00000133117
14199	A2AEX8;A2AE	Fhl1	10	10	38.5	29	0	31.346	104060000	38213000	18510000	Fhl1	2.723157041	Fhl1	Fhl1	323	Four and a half LI	1	10090.ENSMU SP00000110417
14268	A0A087WS56;	Fn1	11	21	12	20	0	54.244	132200000	17490000	1597600	Fn1	7.558604917	Fn1	Fn1	2477	Fibronectin; Fibro	1	10090.ENSMU SP00000054499
14376	Q8BHN3;Q8BH	Ganab	3	8	8.4	10	0	13.783	20324000	6810100	908240	Ganab	2.984390831	Ganab	Ganab	966	Neutral alpha-gluc	2	10090.ENSMU SP00000093965
14489	P62774;A0A0J9	Mtpn	2	2	22.9	3	0	4.9084	11455000	3736200	3683600	Mtpn	3.06594936	Mtpn	Mtpn	118	Myotrophin; Pro	3	10090.ENSMU SP00000031866
14688	P62874;H3BKR	Gnb1	4	3	23.8	5	0	3.5486	27580000	13243000	5032700	Gnb1	2.082609681	Gnb1	Gnb1	340	Guanine nucleotid	1	10090.ENSMU SP00000030940
14701	A0A0N4SVT3;	Gng12	3	2	38.5	5	0	5.06	13984000	6138400	7129200	Gng12	2.278118076	Gng12	Gng12	72	Guanine nucleotid	1	10090.ENSMU SP00000109863
14718	P05201	Got1	2	20	52.8	92	0	127.47	1107100000	2419600000	250250000	Got1	0.457554968	Got1	Got1	413	Aspartate aminotr	3	10090.ENSMU SP00000026196
14768	B2KGR2;O8911	Lancl1	2	2	11.6	2	0.001	2.0786	5895200	2519700	1244100	Lancl1	2.339643608	Lancl1	Lancl1	399	LanC-like protein	2	10090.ENSMU SP00000109612
14775	P11352;A0A0A	Gpx1	3	6	39.3	11	0	12.929	27089000	11912000	3750200	Gpx1	2.274093351	Gpx1	Gpx1	201	Glutathione peroxi	3	10090.ENSMU SP00000081010
14778	A0A1C7ZMZ5;	Gpx3	4	6	32.7	7	0	8.6635	86249000	23441000	17633000	Gpx3	3.679407875	Gpx3	Gpx3	226	Glutathione peroxi	3	10090.ENSMU SP00000081011

14964	G3UZP7;P01899	H2-D1;H2-Q7;H2-Q6;H2-Q4;H2-Q8;H2-L	19	2	13.9	6	0	7.9968	30504000	8443500	2061800	H2-D1	3.612719844	H2-Q7;H2-D1;H2-L;H2-Q4;H2-Q6;H2-Q8	H2-D1	362	H-2 class I histocc	1	10090.ENSMUSP00000134570
15078	E0CZ27;F8WI3	H3f3a;Hist1h3b;Hist1h3a;H3f3c	9	3	19.3	16	0	3.3918	1914400000	866470000	1062100000	H3f3a	2.209424446	H3f3a;H3-3a; H3-3b;H3-5;H3c1; H3c8; H3c10; H3c11;H3c14;H3c2; H3c3; H3c4; H3c6; H3c7; H3c13; H3c14; H3c15	H3f3a	136	Histone H3.3; Vari	1	10090.ENSMUSP00000124509
15078	E0CZ27;F8WI3	H3f3a;Hist1h3b;Hist1h3a;H3f3c	9	3	19.3	16	0	3.3918	1914400000	866470000	1062100000	H3f3a	2.209424446	H3f3a;H3-3a; H3-3b;H3-5;H3c1; H3c8; H3c10; H3c11;H3c14;H3c2; H3c3; H3c4; H3c6; H3c7; H3c13; H3c14; H3c15	H3f3b	136	Variant histone H3	1	10090.ENSMUSP00000102062
15160	P49182	Serpind1	1	2	2.9	2	0.005	1.4514	3735500	1363500	323490	Serpind1	2.739640631	Serpind1	Serpind1	478	Heparin cofactor 2	1	10090.ENSMUSP00000023450
15191	P51859;E0CYW	Hdgf	3	2	8	2	0.003	1.6296	14132000	4364000	1554200	Hdgf	3.238313474	Hdgf	Hdgf	237	Hepatoma-derived	2	10090.ENSMUSP00000005017
15289	A0A0J9YUD8;	Hmgb1	5	4	27.5	7	0	5.8083	47057000	11266000	12665000	Hmgb1	4.176903959	Hmgb1	Hmgb1	215	High mobility grou	1	10090.ENSMUSP00000082682
15381	Q9Z204-4;Q9Z2	Hnrnpc	7	5	20.5	5	0	9.3673	39360000	7376100	3611600	Hnrnpc	5.336153252	Hnrnpc	Hnrnpc	313	Heterogeneous nu	2	10090.ENSMUSP00000107237
15382	Q5EBP8;P49312	Hnrnpa1	3	4	19.3	8	0	13.717	9554500	4606200	1236600	Hnrnpa1	2.074269463	Hnrnpa1	Hnrnpa1	373	Involved in the pa	2	10090.ENSMUSP00000042658
15387	B2M1R6;P6197	Hnrnpk	17	13	36.4	31	0	46.943	250970000	111780000	21126000	Hnrnpk	2.245213813	Hnrnpk	Hnrnpk	463	Heterogeneous nu	2	10090.ENSMUSP00000039269
15388	G5E924;Q8R081	Hnrnpl	3	4	7	7	0	8.1682	26912000	10090000	2512200	Hnrnpl	2.667195243	Hnrnpl	Hnrnpl	615	Heterogeneous nu	2	10090.ENSMUSP00000134734
15452	P00493	Hprt1	1	5	25.7	6	0	8.7877	14973000	6852800	2314500	Hprt1	2.184946299	Hprt1	Hprt	218	Hypoxanthine-gua	1	10090.ENSMUSP00000026723
15929	P70404	Idh3g	1	9	34.4	24	0	43.289	194400000	456170000	66284000	Idh3g	0.426156915	Idh3g	Idh3g	393	Isocitrate dehydro	3	10090.ENSMUSP00000056502
16423	A0A2R8VK70;	Cd47	8	3	9.3	3	0	2.6171	7474300	2891300	1198500	Cd47	2.585100128	Cd47	Cd47	324	Leukocyte surface	1	10090.ENSMUSP00000099853
16424	F8WJ05;Q61702	Itih1	3	5	6.7	8	0	9.7564	26984000	8661100	1204200	Itih1	3.115539597	Itih1	Itih1	907	Inter-alpha-trypsin	1	10090.ENSMUSP00000126449
16549	A0A3B2WCD8;	Khsrp	3	3	7.9	5	0	3.8302	23383000	6351900	1445200	Khsrp	3.681260725	Khsrp	Khsrp	748	Far upstream elem	2	10090.ENSMUSP00000007814
16592	Q05816	Fabp5	1	4	31.9	9	0	14.744	64701000	30416000	21610000	Fabp5	2.127202788	Fabp5	Fabp5	135	Fatty acid-binding	3	10090.ENSMUSP00000029046
16796	A2A6G6;A2A6	Lasp1	9	3	36	3	0	7.2671	22402000	10210000	9092400	Lasp1	2.194123408	Lasp1	Lasp1	263	LIM and SH3 dom	1	10090.ENSMUSP00000042123
16852	P16045	Lgals1	2	8	71.9	34	0	63.458	603740000	232520000	135090000	Lgals1	2.596507827	Lgals1	Lgals1	135	Galectin-1; Lectin	1	10090.ENSMUSP00000086795
16905	P48678;P48678-	Lmna	4	38	54.3	92	0	206.19	1346300000	357740000	54764000	Lmna	3.763347683	Lmna	Lmna	665	Prelamin-A/C; Lan	1	10090.ENSMUSP00000029699
16906	P14733	Lmnb1	1	12	23	17	0	23.149	87774000	34428000	4588800	Lmnb1	2.549494597	Lmnb1	Lmnb1	588	Lamin-B1; Lamins	1	10090.ENSMUSP00000025486
16952	P10107;A0A494	Anxa1	7	9	26.6	11	0	37.857	116410000	12917000	6961100	Anxa1	9.012154525	Anxa1	Anxa1	346	Annexin A1; Plays	1	10090.ENSMUSP00000025561
16956	P11152	Lpl	1	4	9.7	6	0	7.7038	12526000	27811000	3259100	Lpl	0.450397325	Lpl	Lpl	474	Lipoprotein lipase	3	10090.ENSMUSP00000015712
17022	P51885	Lum	2	10	36.1	17	0	83.28	776660000	36575000	56313000	Lum	21.23472317	Lum	Lum	338	Lumican; Belongs	6	10090.ENSMUSP00000040877
170728	A0A0R4J0S3;Q	Rtn4ip1	2	3	8.3	4	0	5.0507	10939000	22079000	2537500	Rtn4ip1	0.495448163	Rtn4ip1	Rtn4ip1	396	Reticulon-4-intera	5	10090.ENSMUSP00000060940
171210	Q9QYR9;O5513	Acot2;Acot1	10	10	25.6	26	0	33.217	218710000	565930000	55931000	Acot2	0.386461223	Acot1;Acot2	Acot2	453	Acyl-coenzyme A	3	10090.ENSMUSP00000021649
17698	P26041	Msn	2	18	35.9	54	0	56.48	532880000	219470000	36346000	Msn	2.428031166	Msn	Msn	577	Moesin; Probably	1	10090.ENSMUSP00000113071
17705	P00848	Mtatp6	1	2	8.4	21	0	5.5235	17543000	52219000	67293000	Mtatp6	0.335950516	Mtatp6	mt-Atp6	226	ATP synthase sub	3	10090.ENSMUSP00000080996
17758	P27546-3;P2754	Map4	4	5	10.2	17	0	16.332	60246000	28895000	2552000	Map4	2.084997404	Map4	Map4	1125	Microtubule-asso	1	10090.ENSMUSP00000035055
17880	A0A2R8VHF9;	Myh11	5	15	15.4	20	0	26.852	60088000	24582000	1274000	Myh11	2.444390204	Myh11	Myh11	1938	Muscle contractio	1	10090.ENSMUSP00000087756
17886	Q8VDD5	Myh9	4	42	31.7	102	0	242.52	799390000	194510000	12780000	Myh9	4.109762994	Myh9	Myh9	1960	Myosin-9; During	1	10090.ENSMUSP00000016771
17901	E9PWG4;P0597	Myl1	4	3	37.3	5	0	7.9061	9569500	3034600	1889400	Myl1	3.153463389	Myl1	Myl1	188	Myosin light chair	1	10090.ENSMUSP00000027151
17904	A0A1W2P6F6;	Myl6	6	6	39.5	13	0	14.377	150910000	27303000	26456000	Myl6	5.52723144	Myl6	Myl6	151	Myosin light poly	1	10090.ENSMUSP00000128803
17955	B7ZNL2;Q78ZA	Nap1l4	4	4	17.6	9	0	7.171	28693000	5461300	2771500	Nap1l4	5.253877282	Nap1l4	Nap1l4	375	Nucleosome asser	2	10090.ENSMUSP00000072510
17975	P09405	Ncl	1	12	18	22	0	26.76	87945000	38039000	4890900	Ncl	2.311969295	Ncl	Ncl	707	Nucleolin; Nucleol	2	10090.ENSMUSP00000027438
18000	E9Q3V6;P42208	Sept2	10	7	26.5	12	0	16.941	88029000	14395000	5879500	Sept2	6.11524835	Septin2	Sept2	361	Septin-2; Filament	1	10090.ENSMUSP00000027495
18175	Q80XB4-2;Q80	Nrap	7	41	25.8	55	0	83.524	113080000	46054000	3324600	Nrap	2.455378469	Nrap	Nrap	1728	Nebulin-related-an	4	10090.ENSMUSP00000073228
18220	Q02819;A0A1B	Nucb1	5	5	11.1	6	0	12.665	19046000	2639700	996670	Nucb1	7.21521385	Nucb1	Nucb1	459	Nucleobindin-1; N	2	10090.ENSMUSP00000033096
18295	Q62000	Ogn	1	8	27.2	13	0	12.488	357910000	15415000	29319000	Ogn	23.21829387	Ogn	Ogn	298	Mimecan; Induces	6	10090.ENSMUSP00000021822
18453	P09103	P4hb	2	18	37.3	37	0	66.944	355510000	98188000	19289000	P4hb	3.620707215	P4hb	P4hb	509	Protein disulfide-i	2	10090.ENSMUSP00000026122
18458	P29341;Q62029;	Pabpc1;Pabpc2;Pabpc6	5	2	8.3	2	0	4.1673	9034000	2439200	410270	Pabpc1	3.703673336	Pabpc1;Pabpc2;Pabpc6	Pabpc1	636	Polyadenylate-bin	2	10090.ENSMUSP00000001809
18624	Q11136	Pepd	2	4	8.9	5	0	4.0966	3960700	1830200	346930	Pepd	2.164080428	Pepd	Pepd	493	Xaa-Pro dipeptida	3	10090.ENSMUSP00000075683
18813	P50580-2;P5058	Pa2g4	2	5	15.3	7	0	5.6431	29557000	12791000	3214300	Pa2g4	2.310765382	Pa2g4	Pa2g4	394	Proliferation-asso	2	10090.ENSMUSP00000026425
18826	Q61233	Lcp1	5	12	26.5	21	0	52.367	197240000	21887000	7410200	Lcp1	9.01174213	Lcp1	Lcp1	627	Plastin-2; Actin-bi	1	10090.ENSMUSP00000116271
																			10090.ENSMU

19035	P24369	Ppib	1	8	36.1	15	0	17.38	173550000	54652000	25901000	Ppib	3.175547098	Ppib	Ppib	216	Peptidyl-prolyl cis	2	10090.ENSMU SP00000034947
19156	E9PZ00;Q8BFQ	Psap	5	8	14.2	11	0	14.216	85925000	33191000	5052200	Psap	2.588804194	Psap	Psap	554	Prosaposin; Sapos	2	10090.ENSMU SP00000101105
19205	Q8CB58;Q8BGJ	Ptbp1	6	3	9.2	3	0	3.8373	6451600	1394000	537690	Ptbp1	4.628120517	Ptbp1	Ptbp1	555	Plays a role in pre-	2	10090.ENSMU SP00000126192
192176	B7FAV1;B7FAU	Flna	7	31	17.8	38	0	76.518	226100000	22324000	2522700	Flna	10.12811324	Flna	Flna	2647	Filamin-A; Actin b	1	10090.ENSMU SP00000033699
19223	O35074;Q8BXC	Ptgis	2	8	18.2	8	0	16.393	62610000	6261300	2760600	Ptgis	9.999520866	Ptgis	Ptgis	501	Prostacyclin synth	11	10090.ENSMU SP00000018113
19230	Q91YR1	Twf1	1	3	14	4	0	3.7779	5332600	1784700	472700	Twf1	2.987953157	Twf1	Twf1	350	Twinfilin-1; Actin-	1	10090.ENSMU SP00000023087
19231	P26350	Ptma	4	6	35.1	15	0	20.492	101670000	24230000	52863000	Ptma	4.196037969	Ptma	Ptma	111	Prothymosin alpha	5	10090.ENSMU SP00000044188
192662	Q99PT1	Arhgdia	1	8	49.5	19	0	42.2	102420000	42418000	25238000	Arhgdia	2.414540997	Arhgdia	Arhgdia	204	Rho GDP-dissocia	1	10090.ENSMU SP00000063714
19325	P61027	Rab10	1	3	18.5	4	0	2.6968	13207000	5787200	2004100	Rab10	2.282105336	Rab10	Rab10	200	Ras-related protei	3	10090.ENSMU SP00000021001
19353	Q3TLP8;P63001	Rac1;Rac3; Rac2	15	4	24.6	14	0	9.0159	102180000	50851000	28681000	Rac1	2.009400012	Rac1;Rac3; Rac2	Rac1	192	Ras-related C3 bot	1	10090.ENSMU SP00000079380
19656	A0A2I3BRL8;Q	Rbmxl1;Rb mx	8	5	13.1	6	0	7.2963	25017000	9745100	1771200	Rbmxl1	2.567136304	Rbmx;Gm73 24;Rbmxl1	Rbmxl1	388	RNA binding moti	2	10090.ENSMU SP00000048153
19659	Q00915	Rbp1	1	6	47.4	7	0	49.421	62532000	2182400	7562900	Rbp1	28.65285924	Rbp1	Rbp1	135	Retinol-binding pr	3	10090.ENSMU SP00000059749
19941	P61255;B1ARA	Rpl26	3	5	22.8	5	0	8.3121	43465000	15799000	13981000	Rpl26	2.751123489	Rpl26	Rpl26	145	Component of the	2	10090.ENSMU SP00000073175
20014	A2ACG7;Q9DB	Rpn2	2	7	15.1	8	0	21.668	31337000	8578600	2179900	Rpn2	3.652927051	Rpn2	Rpn2	631	Dolichyl-diphosph	2	10090.ENSMU SP00000112081
20055	P14131	Rps16	1	8	41.8	16	0	14.259	198140000	90549000	40052000	Rps16	2.18820749	Rps16	Rps16	146	Ribosomal protein	2	10090.ENSMU SP00000103940
20090	P62274	Rps29	1	2	32.1	3	0.001	2.2703	8229500	3791800	10249000	Rps29	2.170341263	Rps29	Rps29	56	Ribosomal protein	2	10090.ENSMU SP00000038352
20167	O70622-2;O7062	Rtn2	2	2	12.3	5	0	6.1027	16533000	40899000	14135000	Rtn2	0.404239712	Rtn2	Rtn2	471	Reticulon-2; Reticu	2	10090.ENSMU SP00000032559
20174	Q9WTM5;A0A	Ruvbl2	5	4	9.7	2	0	5.6312	8313200	3800400	628350	Ruvbl2	2.187453952	Ruvbl2	Ruvbl2	463	RuvB-like 2; Invol	1	10090.ENSMU SP00000103400
20195	P50543	S100a11	1	2	20.4	4	0	11.343	70566000	8824300	19885000	S100a11	7.996781614	S100a11	S100a11	98	Protein S100-A11;	1	10090.ENSMU SP00000029515
20196	A0A0A0MQ90;	S100a13	2	4	25	4	0	9.8342	16443000	6883500	3941900	S100a13	2.38875572	S100a13	S100a13	160	Protein S100-A13;	2	10090.ENSMU SP00000047737
20200	P14069	S100a6	1	3	25.8	4	0	3.8432	142220000	27294000	48108000	S100a6	5.210669012	S100a6	S100a6	89	Protein S100-A6; I	1	10090.ENSMU SP00000001051
20226	Q8C483;P26638	Sars	4	3	5.6	5	0	4.4039	12007000	5596300	1039200	Sars	2.145524722	Sars;Sars1	Sars	536	Serine--tRNA ligas	2	10090.ENSMU SP00000099685
20340	F8WHM5;Q615	Glg1	3	3	3	5	0	4.8828	9239900	1829400	259640	Glg1	5.050781677	Glg1	Glg1	1175	Golgi apparatus pr	2	10090.ENSMU SP00000131355
20382	Q62093	Srsf2	1	4	21.7	4	0	10.886	19837000	8888800	4677700	Srsf2	2.231684817	Srsf2	Srsf2	221	Serine/arginine-ric	2	10090.ENSMU SP00000090059
20430	Q7TMB8;A0A(	Cyfip1;Cyfi p2	6	4	3.7	3	0	6.1033	9575300	2874000	246180	Cyfip1	3.331697982	Cyfip1;Cyfi p2	Cyfip1	1253	Cytoplasmic FMR	1	10090.ENSMU SP00000032629
20501	P53986	Slc16a1	1	4	8.3	9	0	17.427	26616000	60477000	10030000	Slc16a1	0.440101195	Slc16a1	Slc16a1	493	Monocarboxylate	20	10090.ENSMU SP00000045216
20638	P27048;P63163;	Snrpb;Snrp n	3	2	6.5	5	0.001	1.9392	21886000	8734700	2998100	Snrpb	2.505638431	Snrpb;Snrp n	Snrpb	231	Small nuclear ribo	2	10090.ENSMU SP00000099488
20650	B7ZNU9;Q6123	Sntb2	2	2	4.1	2	0.001	2.0567	4268500	1844400	345230	Sntb2	2.314302754	Sntb2	Sntb2	520	Beta-2-syntrophin	1	10090.ENSMU SP00000037324
20823	P32067;F6SXM	Ssb	4	3	8.4	4	0	5.8714	8138800	3656400	794950	Ssb	2.225905262	Ssb	Ssb	415	Lupus La protein l	2	10090.ENSMU SP00000088365
20901	Q9Z1Z2	Strap	1	4	12	5	0	5.4007	9969300	4320600	952150	Strap	2.307387863	Strap	Strap	350	Serine-threonine k	2	10090.ENSMU SP00000068267
20917	Q9Z2I8-2;Q9Z2I	Suclg2	3	12	34.9	23	0	65.829	91999000	195740000	19974000	Suclg2	0.470006131	Suclg2	Suclg2	433	Succinate--CoA lig	3	10090.ENSMU SP00000078774
20928	E9PUE8;P70170	Abcc9	4	2	1.6	1	0.005	1.4266	2188200	4947100	156440	Abcc9	0.442319743	Abcc9	Abcc9	1546	ATP-binding cass	3	10090.ENSMU SP00000084805
210582	A0A1W2P7G5;	Coq10a	7	6	27.6	12	0	12.983	27095000	77108000	11929000	Coq10a	0.351390258	Coq10a	Coq10a	259	Coenzyme Q10 ho	3	10090.ENSMU SP00000036213
21345	P37804;A0A1L1	Tagln	2	10	52.7	20	0	36.608	126570000	32763000	14392000	Tagln	3.863199341	Tagln	Tagln	201	Transgelin; Actin	1	10090.ENSMU SP00000034590
21346	Q9WVA4	Tagln2	3	12	62.8	26	0	47.783	333710000	66022000	37894000	Tagln2	5.054527279	Tagln2	Tagln2	199	Transgelin-2; Tran	1	10090.ENSMU SP00000106861
213827	Q5XJY5	Arcn1	1	3	6.1	2	0	4.5669	14839000	5853700	1146700	Arcn1	2.534977877	Arcn1	Arcn1	511	Coatomer subunit	2	10090.ENSMU SP00000034607
214579	Q8BWF0	Aldh5a1	1	8	20.1	14	0	19.961	19349000	50120000	4104000	Aldh5a1	0.386053472	Aldh5a1	Aldh5a1	523	Succinate-semiald	3	10090.ENSMU SP00000040591
216197	Q8BMK4	Ckap4	1	13	25.4	21	0	50.591	98295000	17251000	4726900	Ckap4	5.697930555	Ckap4	Ckap4	575	Cytoskeleton-ass	2	10090.ENSMU SP00000050336
216616	Q8BPB5	Efemp1	2	6	14.6	8	0	7.4773	31777000	797120	1477600	Efemp1	39.86476315	Efemp1	Efemp1	493	EGF-containing fib	6	10090.ENSMU SP00000020759
216792	Q8CAK1	Iba57	1	2	7	4	0	4.1182	1965000	3985700	720090	Iba57	0.49301252	Iba57	Iba57	358	Putative transferas	7	10090.ENSMU SP00000049823
217666	Q91YP0	L2hgdh	1	4	9.5	4	0	12.642	5433200	11322000	1070300	L2hgdh	0.47987988	L2hgdh	L2hgdh	464	L-2-hydroxyglutara	3	10090.ENSMU SP00000021370
217830	E9QMK9;Q8BH	9030617O03 Rik	3	6	11.9	8	0	10.779	3461700	21630000	1315300	9030617O0	0.160041609	Dglucy	Dglucy	617	D-glutamate cycla	8	10090.ENSMU SP00000067830
21838	A0A1L1SUX8;I	Thy1	2	2	19.2	3	0	2.9362	7298500	3261100	2057900	Thy1	2.238048511	Thy1	Thy1	162	Thy-1 membrane g	1	10090.ENSMU SP00000110489
21881	P40142;A0A286	Tkt	2	8	17.7	10	0	16.588	62435000	11041000	3638700	Tkt	5.65483199	Tkt	Tkt	623	Transketolase; Ca	3	10090.ENSMU SP00000022529
21894	P26039	Tln1	4	30	18.4	73	0	112.11	275220000	111790000	4304500	Tln1	2.461937562	Tln1	Tln1	2541	Talin-1; Probably	1	10090.ENSMU SP00000030187
21917	Q61029-3;Q6102	Tmpo	6	4	15	6	0	13.133	11038000	3283700	899460	Tmpo	3.361452021	Tmpo	Tmpo	693	Lamina-associated	1	10090.ENSMU SP00000020123
22004	P58774-2;A2AI	Tpm2	2	2	43.7	10	0	14.361	31308000	11767000	3779500	P58774-2	2.660661171	Tpm2	Tpm2	284	Tropomyosin beta	1	10090.ENSMU SP00000030184
22027	P08113;F7C312	Hsp90b1	2	18	24.4	31	0	50.277	319990000	104380000	15109000	Hsp90b1	3.065625599	Hsp90b1	Hsp90b1	802	Endoplasmin; Mol	1	10090.ENSMU SP00000020238
22042	Q62351;Q8C872	Tfrc	2	6	8.1	6	0	7.484	9763900	3353000	714140	Tfrc	2.911989263	Tfrc	Tfrc	763	Transferrin recept	1	10090.ENSMU SP00000023486
22195	A0A338P7E5;P(	Ube2l3	4	4	32.8	9	0	5.8535	13027000	35478000	11906000	Ube2l3	0.367185298	Ube2l3	Ube2l3	154	Ubiquitin-conjuga	3	10090.ENSMU SP00000111363
22223	Q9R0P9	Uchl1	1	5	24.7	9	0	9.718	50960000	12711000	8735800	Uchl1	4.009125954	Uchl1	Uchl1	223	Ubiquitin carboxyl	1	10090.ENSMU SP00000031131
22240	E9PWE8;Q3TT	Dpysl3	3	11	28.8	13	0	24.167	77543000	8512700	2589300	Dpysl3	9.109095822	Dpysl3	Dpysl3	570	Dihydropyrimidina	1	10090.ENSMU SP00000025379
22321	Q9Z1Q9;G3UY	Vars	4	4	4	6	0	6.1784	11866000	3782900	353690	Vars	3.13674694	Vars;Vars1	Vars	1263	Valine--tRNA ligas	2	10090.ENSMU SP00000084572
22333	Q60932-2;Q6093	Vdac1	3	16	71	97	0	118.57	1226700000	2680300000	428280000	Vdac1	0.457672649	Vdac1	Vdac1	283	Voltage-dependen	3	10090.ENSMU SP00000099819
22352	P20152;A0A0A	Vim	21	30	70.2	128	0	254.27	8662100000	1077100000	364740000	Vim	8.042057376	Vim	Vim	466	Vimentin; Vimentin	1	10090.ENSMU SP00000028062
223601	Q921M7;A0A2I	Fam49b	5	4	13.9	3	0	6.0749	8123200	3804000	924570	Fam49b	2.135436383	Cyrib	Fam49b	324	Protein FAM49B;	1	10090.ENSMU SP00000066359
22370	P29788	Vtn	1	4	9.4	7	0	7.8022	33309000	4678600	2038100	Vtn	7.119437439	Vtn	Vtn	478	Vitronectin; Vitron	6	10090.ENSMU SP00000017488
																			10090.ENSMU

224904	Q8R404	Qil1	1	2	24.4	2	0	10.731	9660600	24030000	19039000	Qil1	0.402022472	Micos13	Mic13	119	MICOS complex s	3	10090.ENSMUSP00000052908
225027	A0A3Q4EH04;	Srsf7	7	3	32.1	6	0	5.3422	21045000	9030200	6316800	Srsf7	2.330513167	Srsf7	Srsf7	238	Serine/arginine-ric	2	10090.ENSMUSP00000070983
22608	P62960;A2BGG	Ybx1	6	3	23	8	0	5.8365	9493600	2082200	1088800	Ybx1	4.559408318	Ybx1	Ybx1	322	Nuclease-sensitiv	2	10090.ENSMUSP00000078589
22629	P68510	Ywhah	1	7	47.6	16	0	27.171	103700000	44863000	13259000	Ywhah	2.311481622	Ywhah	Ywhah	246	14-3-3 protein eta;	1	10090.ENSMUSP00000019109
22630	P68254-2;P6825	Ywhaq	2	6	40.7	10	0	38.829	46379000	13605000	5772200	Ywhaq	3.408967291	Ywhaq	Ywhaq	245	14-3-3 protein thet	1	10090.ENSMUSP00000100067
22631	P63101;A0A2I3	Ywhaz	5	9	52.2	40	0	83.286	1288900000	637410000	209030000	Ywhaz	2.022089393	Ywhaz	Ywhaz	245	14-3-3 protein zeta	1	10090.ENSMUSP00000022894
227753	P13020-2;P1302	Gsn	6	11	17.4	20	0	22.315	97407000	24869000	5007100	Gsn	3.916804053	Gsn	Gsn	780	Gelsolin; Calcium-	1	10090.ENSMUSP00000028239
229279	Q8BG05-2;A2A	Hnrnpa3	3	6	21.8	17	0	18.77	115140000	40697000	13088000	Hnrnpa3	2.82920117	Hnrnpa3	Hnrnpa3	379	Heterogeneous nu	2	10090.ENSMUSP00000107595
230027	Q8BMS4	Coq3	1	4	17	7	0	15.247	16813000	35128000	5396200	Coq3	0.478621043	Coq3	Coq3	370	Ubiquinone biosy	3	10090.ENSMUSP00000029909
230908	A0A087WR97;	Tardbp	12	2	8.6	3	0	4.5314	11621000	3399700	1958300	Tardbp	3.418242786	Tardbp	Tardbp	414	TAR DNA-bindin	2	10090.ENSMUSP00000081142
231086	Q99JY0	Hadhb	2	21	48.6	119	0	125.62	1838400000	4079600000	400570000	Hadhb	0.450632415	Hadhb	Hadhb	475	Trifunctional enzy	3	10090.ENSMUSP00000110434
232440	A0A0N4SV66;C	H2afj;Hist2h2ac;Hist2h2aa1;Hist1h2aa;Hist1h2ah;Hist1h2ak;Hist1h2af;Hist3h2a;Hist2h2ab	20	2	38.9	28	0	13.625	4261900000	1564300000	1628900000	H2afj	2.724477402	H2aj;H2ac1;H2ac11;H2ac12;H2ac13;H2ac15;H2ac20;H2ac21;H2ac25;H2ac4;H2ac6;H2ac7;H2ac8;Hist1h2af;Hist1h2an;Hist1h2ao;Hist1h2ap;Hist2h2aa1;Hist2h2aa2	H2afj	129	Histone H2A.J; C	1	10090.ENSMUSP00000074142
233489	Q7M6Y3-2;Q7M	Picalm	9	3	5.7	4	0	5.5666	15209000	4934800	1217000	Picalm	3.081989138	Picalm	Picalm	660	Phosphatidylinosi	2	10090.ENSMUSP00000051092
235072	E9Q9F5;E9Q1G	Sept7	9	8	19.2	16	0	37.199	119700000	25268000	9262600	Sept7	4.737217033	Septin7	Sept7	437	Septin-7; Filament	1	10090.ENSMUSP00000110927
235661	Q8R1Q8	Dync1li1	1	2	3.3	2	0	2.5729	16901000	7914900	1311700	Dync1li1	2.135339676	Dync1li1	Dync1li1	523	Cytoplasmic dyne	1	10090.ENSMUSP00000035366
23789	Q9WUM3;A0A	Coro1b	4	3	6	4	0	5.9508	10599000	2513600	1046600	Coro1b	4.216661362	Coro1b	Coro1b	484	Regulates leading	1	10090.ENSMUSP00000008893
23790	Q9WUM4	Coro1c	4	6	14.3	8	0	8.7155	22758000	3228500	1791800	Coro1c	7.049094007	Coro1c	Coro1c	474	Coronin-1C; May	1	10090.ENSMUSP00000004646
23874	Q9WUA2;A0A	Farsb	3	3	5.8	2	0	2.7419	3593400	1688600	279430	Farsb	2.128035059	Farsb	Farsb	589	phenylalanyl-tRN	3	10090.ENSMUSP00000129828
23876	A0A1Y7VJW9;	Fbln5	2	11	25.6	18	0	49.189	240480000	12174000	15410000	Fbln5	19.75357319	Fbln5	Fbln5	448	Fibulin-5; Essentia	6	10090.ENSMUSP00000021603
239017	B2RXT3;E9Q7L	Ogdhl	2	20	29.9	43	0	58.787	151500000	322700000	16163000	Ogdhl	0.469476294	Ogdhl	Ogdhl	1029	Oxoglutarate dehy	3	10090.ENSMUSP00000022480
23950	A0A0G2JER9;G	Dnajb6;Dnajb7	7	2	9.9	4	0	2.9137	4788300	2324200	1135500	Dnajb6	2.060192755	Dnajb6;Dnajb7	Dnajb6	365	DnaJ homolog sub	2	10090.ENSMUSP00000008733
23986	Q3TCD4;Q9WU	Eci2	9	4	17.2	13	0	16.158	49366000	105750000	14432000	Eci2	0.466817967	Eci2	Eci2	391	Enoyl-CoA delta i	3	10090.ENSMUSP00000131735
241431	A0A5F8MPR4;	Xirp2	7	32	10.3	40	0	58.737	21624000	3753700	932600	Xirp2	5.760716093	Xirp2	Xirp2	3283	Xin actin-binding	4	10090.ENSMUSP00000107966
246221	Q99J99;A0A2R	Mpst	3	2	7.4	1	0	2.4437	3120200	11765000	1501500	Mpst	0.26521037	Mpst	Mpst	297	Transfer of a sulfu	3	10090.ENSMUSP00000043061
257633	A0A1D5RMC8;	Acsf3	3	3	7.1	2	0	3.9765	2419400	5558700	388650	Acsf3	0.435245651	Acsf3	Acsf3	583	Acyl-CoA synthet	3	10090.ENSMUSP00000015160
26457	Q60714	Slc27a1	1	2	3.3	4	0	3.0469	2979600	6763700	518810	Slc27a1	0.440528113	Slc27a1	Slc27a1	646	Long-chain fatty a	3	10090.ENSMUSP00000034267
26462	A0A0U1RPC6;	Txnrd2	14	3	30.9	5	0	5.1101	5083800	10470000	2695300	Txnrd2	0.485558739	Txnrd2	Txnrd2	524	Thioredoxin reduc	3	10090.ENSMUSP00000111269
268373	P17742;A0A1L	Ppia	5	7	47	20	0	18.312	617920000	216470000	121050000	Ppia	2.854529496	Ppia	Ppia	164	Peptidyl-prolyl cis	1	10090.ENSMUSP00000117987
26914	Q9QZQ8-2;Q9Q	H2afy	3	5	20.9	7	0	9.1494	28816000	5701400	2637700	H2afy	5.054197215	Macroh2a1	H2afy	372	Core histone macr	1	10090.ENSMUSP00000016081
26949	Q62465	Vat1	1	4	12.3	6	0	13.629	25510000	4551700	2675000	Vat1	5.604499418	Vat1	Vat1	406	Synaptic vesicle n	6	10090.ENSMUSP00000048350
269951	P54071	Idh2	4	23	54.4	275	0	323.31	4775300000	10545000000	1107300000	Idh2	0.452849692	Idh2	Idh2	452	Isocitrate dehydro	3	10090.ENSMUSP00000103007
27061	Q61335	Bcap31	1	3	11.8	5	0	8.4924	45135000	11169000	6195900	Bcap31	4.04109589	Bcap31	Bcap31	245	B-cell receptor-as	2	10090.ENSMUSP00000002091
27273	O70571	Pdk4	1	5	12.9	8	0	7.8725	4184600	23671000	2998300	Pdk4	0.176781716	Pdk4	Pdk4	412	[Pyruvate dehydro	3	10090.ENSMUSP00000019721
27367	P27659;A0A2R	Rpl3	4	6	17.6	10	0	10.219	110200000	11779000	8394400	Rpl3	9.355632906	Rpl3	Rpl3	403	60S ribosomal pro	2	10090.ENSMUSP00000080354
27979	Q8JZQ9	Eif3b	2	5	6.4	5	0	9.2445	11466000	2236700	458550	Eif3b	5.126302142	Eif3b	Eif3b	803	Eukaryotic transla	2	10090.ENSMUSP00000098076
27984	Q8C845;Q9D8Y	Efhd2	2	4	17.1	7	0	14.975	22273000	5859900	4193300	Efhd2	3.800918104	Efhd2	Efhd2	240	May regulate B-ce	1	10090.ENSMUSP00000044502
286940	Q80X90	Flnb	1	12	7.1	13	0	19.922	25514000	9369100	342950	Flnb	2.723207138	Flnb	Flnb	2602	Filamin-B; Connec	1	10090.ENSMUSP00000052020
29875	Q9JKF1	Iqgap1	3	10	6.8	11	0	20.734	47493000	4044400	684090	Iqgap1	11.74290377	Iqgap1	Iqgap1	1657	Ras GTPase-activa	1	10090.ENSMUSP00000128278
30937	Q8VEE1	Lmcd1	1	3	9.9	5	0	6.0788	6804700	3067000	696020	Lmcd1	2.218682752	Lmcd1	Lmcd1	365	LIM and cysteine-	14	10090.ENSMUSP00000032376
320011	Q6P5E4	Uggt1	2	3	2.4	4	0	5.0174	6627100	2439400	173640	Uggt1	2.716692629	Uggt1	Uggt1	1551	UDP-glucose:glyc	2	10090.ENSMUSP00000037930
321022	A0A087WNP6;	Cdv3	3	2	12.8	3	0	5.4383	10998000	2582100	1714500	Cdv3	4.259323806	Cdv3	Cdv3	281	Protein CDV3; Ca	4	10090.ENSMUSP00000044420
326618	Q6IRU2;A0A57	Tpm4	2	13	57.3	20	0	78.704	197940000	64466000	28627000	Tpm4	3.070455744	Tpm4	Tpm4	248	Tropomyosin alph	1	10090.ENSMUSP00000003575
330217	E9Q7N6;Q3V1B	Gal3st4	2	2	3.4	4	0.003	1.6674	12314000	29634000	3037200	Gal3st4	0.415536208	Gal3st4	Gal3st4	480	galactose-3-O-sul	23	10090.ENSMUSP00000098099
50706	Q62009;Q62009-	Postn	7	20	30.5	38	0	154.9	794430000	2428500	19385000	Postn	327.1278567	Postn	Postn	811	Periostin; Induces	6	10090.ENSMUSP00000072773
50709	P43274	Hist1h1e	3	2	33.8	8	0	3.8405	67898000	31088000	15388000	Hist1h1e	2.184058157	H1-4	Hist1h1e	219	Histone H1.4; Hist	1	10090.ENSMUSP00000057308
50797	O55029	Copb2	1	5	5.5	7	0	7.6184	15802000	6761500	768400	Copb2	2.337055387	Copb2	Copb2	905	Coatomer subunit	2	10090.ENSMUSP00000035033
51793	O08972;Q99LD	Ddah2	3	2	9	3	0	7.0945	20576000	4454500	2135000	Ddah2	4.619149175	Ddah2	Ddah2	285	N(G),N(G)-dimeth	5	10090.ENSMUSP00000007255
51798	O35459;F7B227	Ech1	2	13	48.9	38	0	140.53	472780000	1277200000	148550000	Ech1	0.37016912	Ech1	Ech1	327	Delta(3,5)-Delta(2,	3	10090.ENSMUSP00000066092
52377	A0A1B0GSK5;	Rcn3	6	4	23.7	4	0	19.372	37400000	439910	4291500	Rcn3	85.01738992	Rcn3	Rcn3	328	Reticulocalbin-3; 1	6	10090.ENSMUSP00000019683
52398	A0A0J9YTY0;	Sept11	14	3	6.8	4	0	4.8077	22603000	4471700	1526500	Sept11	5.054677192	Septin11	Sept11	429	Septin-11; Filamen	1	10090.ENSMUSP00000074293
52538	Q8BWT1	Acaa2	2	23	71	135	0	298.24	1743800000	5128200000	490020000	Acaa2	0.34004134	Acaa2	Acaa2	397	3-ketoacyl-CoA th	3	10090.ENSMUSP00000037348

54127	G3UYV7;P62858	Rps28	2	3	57.1	4	0	25.482	71458000	30189000	50166000	Rps28	2.3670211	Rps28	Rps28	69	Ribosomal protein	2	10090.ENSMU SP00000110013
54132	O70400;S4R1V0	Pdlim1	2	11	33.9	21	0	21.347	79856000	29282000	11151000	Pdlim1	2.727136125	Pdlim1	Pdlim1	327	PDZ and LIM dom	1	10090.ENSMU SP00000064545
54381	A0A2I3BPG0;A	Cpq	3	2	18.3	2	0	3.7716	25848000	4054700	6949400	Cpq	6.374824278	Cpq	Cpq	470	Carboxypeptidase	17	10090.ENSMU SP00000039046
54401	Q9CQV8-2;Q9C	Ywhab	3	4	40.6	10	0	13.048	61428000	29622000	9568600	Ywhab	2.073728985	Ywhab	Ywhab	246	14-3-3 protein beta	1	10090.ENSMU SP00000018470
54473	Q9QZ06;Q8C5C	Tollip	4	4	15.3	4	0	5.7481	4975700	14381000	2404500	Tollip	0.345991238	Tollip	Tollip	274	Toll-interacting pr	9	10090.ENSMU SP00000001950
54709	Q9QZD9	Eif3i	1	2	6.8	2	0	3.8418	5133100	2402200	594800	Eif3i	2.136832903	Eif3i	Eif3i	325	Eukaryotic translat	2	10090.ENSMU SP00000099653
55990	Q8K2I3	Fmo2	2	3	7.3	3	0	8.357	6796500	1406900	335970	Fmo2	4.830833748	Fmo2	Fmo2	535	Dimethylaniline m	19	10090.ENSMU SP00000044405
56086	A2BE92;A2BE9	Set	5	3	23.2	4	0	7.9981	19859000	8375400	6775400	Set	2.371110634	Set	Set	289	Protein SET; Multi	5	10090.ENSMU SP00000099930
56199	Q9JI39	Abcb10	1	4	6.6	5	0	6.1769	3462900	7823600	538730	Abcb10	0.442622322	Abcb10	Abcb10	715	ATP-binding cass	7	10090.ENSMU SP00000075011
56295	A0A1L1ST61;A	Higd1a	5	2	35.5	6	0	4.9797	10081000	1948100	5961700	Higd1a	5.174785689	Higd1a	Higd1a	95	HIG1 domain famil	3	10090.ENSMU SP00000054881
56378	Q9JM76;A0A0C	Arpc3	5	2	11.2	2	0.001	1.8317	9431900	1825800	1438800	Arpc3	5.16589988	Arpc3	Arpc3	178	Actin-related prot	1	10090.ENSMU SP00000099584
56421	Q8C605;Q9WU/	Pfkp	4	4	9.4	7	0	7.6366	9898300	4555900	782840	Pfkp	2.172633289	Pfkp	Pfkp	784	"ATP-dependent	3	10090.ENSMU SP00000117030
56431	Q9R0P5	Dstn	1	7	33.9	14	0	25.623	103370000	41148000	19827000	Dstn	2.512151259	Dstn	Dstn	165	Destrin; Actin-dep	1	10090.ENSMU SP00000099461
56451	Q9WUM5	Suclg1	2	8	32.9	42	0	51.978	149950000	362820000	60431000	Suclg1	0.413290337	Suclg1	Suclg1	346	Succinate--CoA li	3	10090.ENSMU SP00000065113
56463	Q78PY7	Snd1	2	6	7.1	9	0	9.0634	27607000	5930400	976340	Snd1	4.655166599	Snd1	Snd1	910	Staphylococcal nu	2	10090.ENSMU SP00000001460
56530	A0A1W2P729;(	Cnpy2	2	2	19.1	3	0	3.7742	6567200	2903200	1370700	Cnpy2	2.262055663	Cnpy2	Cnpy2	182	Protein canopy ho	2	10090.ENSMU SP00000026446
56702	P43276	Hist1h1b	1	6	32.7	10	0	9.031	187550000	25246000	30404000	Hist1h1b	7.428899628	H1-5	Hist1h1b	223	Histone H1.5; Hist	1	10090.ENSMU SP00000079356
56752	Q3U367;Q9JLJ2	Aldh9a1	2	3	6.2	3	0	4.4569	5158200	2487600	915570	Aldh9a1	2.073564882	Aldh9a1	Aldh9a1	518	4-trimethylaminob	3	10090.ENSMU SP00000028004
57423	P56135;F8WHP	Atp5j2	2	4	36.4	10	0	9.657	126270000	365970000	208960000	Atp5j2	0.345028281	Atp5j2;Atp5mf	Atp5j2	88	ATP synthase sub	3	10090.ENSMU SP00000125504
58810	Q9JII6;B1AXW	Akr1a1	2	6	22.2	9	0	24.938	41875000	18339000	3773200	Akr1a1	2.283385136	Akr1a1	Akr1a1	325	Alcohol dehydrog	3	10090.ENSMU SP00000030455
59013	Q8C2Q7;P70333	Hnrnph1;Hnrnph2	5	3	9.1	7	0	24.416	25967000	10680000	2535100	Hnrnph1	2.431367041	Hnrnph1;Hnrnph2	Hnrnph1	472	Heterogeneous nu	2	10090.ENSMU SP00000076989
59029	O35593	Psmd14	1	2	6.5	2	0	2.8685	13331000	5929100	1843300	Psmd14	2.24840195	Psmd14	Psmd14	310	26S proteasome n	2	10090.ENSMU SP00000028278
60595	A0A1L1SV25;P	Actn4	6	14	36.3	21	0	26.241	123460000	29845000	3789300	Actn4	4.136706316	Actn4	Actn4	912	Alpha-actinin-4; F	1	10090.ENSMU SP00000066068
619547	Q9D1R9;A0A0C	Rpl34	3	4	23.1	7	0	7.6779	57613000	19929000	19040000	Rpl34	2.89091274	Rpl34	Rpl34	117	60S ribosomal pro	2	10090.ENSMU SP00000086614
63985	A0A2I3BR94;A	Gmfb;Gmfg	6	2	14.6	2	0	2.6512	16408000	5446800	3589700	Gmfb	3.012410957	Gmfb;Gmfg	Gmfb	142	Glia maturation fa	1	10090.ENSMU SP00000107448
64138	Q9WUU7	Ctsz	1	4	12.4	4	0	5.2159	33910000	5505500	3580600	Ctsz	6.15929525	Ctsz	Ctsz	306	Cathepsin Z; Exhit	2	10090.ENSMU SP00000016400
654795	Q5M8N4;A0A2	Sdr39u1	4	5	20.1	6	0	12.143	9631600	19764000	4321700	Sdr39u1	0.4873305	Sdr39u1	Sdr39u1	293	Epimerase family p	24	10090.ENSMU SP00000106957
65973	Q8CBM2;A2AI	Asph	11	3	8.7	6	0	12.803	19567000	7668100	1397200	Asph	2.551740327	Asph	Asph	741	Aspartyl/asparagi	4	10090.ENSMU SP00000077273
66054	Q9D1A2;A0A4	Cndp2	3	4	11.4	4	0	9.0294	22288000	8413900	1754100	Cndp2	2.648949952	Cndp2	Cndp2	475	Cytosolic non-spe	3	10090.ENSMU SP00000128696
66092	Q91VC9;A0A28	Ghitm	2	2	5.8	3	0	6.3508	11406000	4025600	2133500	Ghitm	2.833366455	Ghitm	Ghitm	346	Growth hormone-i	3	10090.ENSMU SP00000046212
66152	Q8R1I1	Uqcr10	1	3	50	20	0	13.355	482840000	1003500000	581030000	Uqcr10	0.481155954	Uqcr10	Uqcr10	64	Cytochrome b-c1	3	10090.ENSMU SP00000054856
66212	E9PW43;Q9CQ	Gm10320;Sec61b	2	2	18.6	5	0	4.2471	18291000	6659100	5255100	Gm10320	2.746767581	Gm10320;Sec61b	Sec61b	96	Protein transport	2	10090.ENSMU SP00000067681
66395	E9Q616	Ahnak	4	68	32	108	0	160.6	631030000	187070000	3074700	Ahnak	3.373229272	Ahnak	Ahnak	5656	AHNAK nucleopr	1	10090.ENSMU SP00000090633
66414	A0A0R4J275;Q	Ndufa12	3	9	75.8	43	0	39.433	190820000	399400000	109780000	Ndufa12	0.47776665	Ndufa12	Ndufa12	149	NADH dehydroge	3	10090.ENSMU SP00000136313
66433	B1AXC8;Q8K2	Chchd7	5	2	34.2	2	0	6.2439	940010	8245200	3026100	Chchd7	0.114006937	Chchd7	Chchd7	85	Coiled-coil-helix-c	3	10090.ENSMU SP00000041196
66445	Q9D0M3-2;Q9D	Cyc1	3	11	39.5	71	0	69.76	538710000	1090300000	254140000	Cyc1	0.494093369	Cyc1	Cyc1	325	Cytochrome c1, he	3	10090.ENSMU SP00000023210
66477	Q78IK2	Usmg5	1	3	44.8	17	0	25.42	127480000	260100000	230710000	Usmg5	0.490119185	Atp5mk	Usmg5	58	Up-regulated duri	3	10090.ENSMU SP00000093713
66576	P99028	Uqcrh	2	7	74.2	25	0	85.042	350000000	1275700000	369530000	Uqcrh	0.274359175	Uqcrh	Uqcrh	89	Cytochrome b-c1	3	10090.ENSMU SP00000077744
66695	Q99MQ4	Aspn	1	7	19.3	9	0	40.157	151230000	4815700	9243000	Aspn	31.40353427	Aspn	Aspn	373	Asporin; Binds ca	6	10090.ENSMU SP00000021820
66713	P61161	Actr2	1	5	14.7	10	0	9.7413	39825000	16910000	5618400	Actr2	2.355115316	Actr2	Actr2	394	Actin-related prot	1	10090.ENSMU SP00000000137
66736	Q9CRD2	Emc2	1	2	7.4	3	0	3.0798	3943300	1928500	579190	Emc2	2.044749806	Emc2	Emc2	297	ER membrane prot	3	10090.ENSMU SP00000022962
66841	Q921G7;Q6PF9	Etfdh	2	25	48.2	101	0	181.69	1018600000	2101100000	160970000	Etfdh	0.48479368	Etfdh	Etfdh	616	Electron transfer f	3	10090.ENSMU SP00000029386
66870	A0A0N4SUQ1;	Serbp1	11	4	37.4	9	0	22.504	25129000	12038000	8671800	Serbp1	2.087473002	Serbp1	Serbp1	407	Plasminogen activ	2	10090.ENSMU SP00000039110
66890	Q9DBH5	Lman2	1	4	11.2	7	0	10.908	21338000	7956600	2043700	Lman2	2.681798758	Lman2	Lman2	358	Vesicular integral-	2	10090.ENSMU SP00000021940
66916	Q9CR61	Ndufb7	1	7	46	39	0	26.065	89817000	182490000	57001000	Ndufb7	0.492174914	Ndufb7	Ndufb7	137	NADH dehydroge	3	10090.ENSMU SP00000037341
66945	Q8K2B3	Sdha	2	28	46.7	178	0	211.77	1266600000	2710400000	204270000	Sdha	0.467311098	Sdha	Sdha	664	Succinate dehydro	3	10090.ENSMU SP00000022062
67268	Q6ZWQ9;Q3TH	Myl12a;Myl12b	4	2	40.1	13	0	28.354	99653000	34955000	20145000	Myl12a	2.850894007	Myl12a;Myl12b	Myl12a	172	Myosin, light chai	1	10090.ENSMU SP00000123412
67300	Q5SXR6;Q68FD	Cltc	3	36	25.4	66	0	108.76	374880000	154150000	8625000	Cltc	2.431916964	Cltc	Cltc	1675	Clathrin heavy cha	1	10090.ENSMU SP00000099475
67397	P57759	Erp29	4	3	11.8	3	0	6.5957	26227000	10694000	4337200	Erp29	2.452496727	Erp29	Erp29	262	Endoplasmic retic	2	10090.ENSMU SP00000117347
67460	Q9CQ62	Decr1	1	12	40	56	0	123.55	611060000	1335500000	175240000	Decr1	0.457551479	Decr1	Decr1	335	2,4-dienoyl-CoA r	3	10090.ENSMU SP00000029877
67511	A0A286YDS5;Q	Tmed9	2	2	9.1	2	0	6.4447	18592000	8697200	3097800	Tmed9	2.137699489	Tmed9	Tmed9	235	Transmembrane e	2	10090.ENSMU SP00000105531
67528	Q99P30-5;Q99P	Nudt7	5	2	14	3	0	4.8631	4109400	13339000	3385400	Nudt7	0.308074069	Nudt7	Nudt7	236	Peroxisomal coenz	3	10090.ENSMU SP00000073213
67636	Q91V16	Lyrm5	1	2	18.6	5	0	4.744	9793700	23997000	15468000	Lyrm5	0.408121849	Etfrf1	Lyrm5	86	Electron transfer f	3	10090.ENSMU SP00000107354
67771	Q9CPW4	Arpc5	2	3	25.2	5	0	5.6905	19523000	3784400	2980000	Arpc5	5.158809851	Arpc5	Arpc5	151	Actin-related prot	1	10090.ENSMU SP00000076933
67776	Q99KC8	Vwa5a	2	8	10.8	11	0	9.8632	25848000	11864000	1252400	Vwa5a	2.178691841	Vwa5a	Vwa5a	793	Von Willebrand fa	1	10090.ENSMU SP00000001544
67804	Q9CWK8	Snx2	1	3	10.8	9	0	8.997	20602000	9904300	1557100	Snx2	2.08010662	Snx2	Snx2	519	Sorting nexin-2; In	2	10090.ENSMU SP00000039243
67861	G5E895;S4R2G9	Akr1b10;Akr1b7	4	4	15.2	6	0	10.782	18708000	44632000	5711900	Akr1b10	0.41916114	Akr1b10;Akr1b7	Akr1b10	316	Aldo-keto reducta	3	10090.ENSMU SP00000039114 10090.ENSMU

67900	Q9CRB8	Mtfp1	1	4	27.7	8	0	10.308	43559000	88797000	24732000	Mtfp1	0.490545852	Mtfp1	Mtfp1	166	Mitochondrial fiss	10	10090.ENSMUSP00000004868
67979	Q9D5T0;A0A49	Atad1	4	5	14.1	4	0	8.1503	7967300	20236000	2525900	Atad1	0.393719114	Atad1	Atad1	361	ATPase family A/	2	10090.ENSMUSP00000069962
68089	P59999	Arpc4	3	4	25	9	0	8.3094	79343000	27018000	16518000	Arpc4	2.936671848	Arpc4	Arpc4	168	Actin-related prot	1	10090.ENSMUSP00000114839
68202	Q9CPP6	Ndufa5	2	5	43.1	19	0	34.948	216370000	465840000	229280000	Ndufa5	0.46447278	Ndufa5	Ndufa5	116	NADH dehydroge	3	10090.ENSMUSP00000023851
68267	Q9D6M3;E9PV9	Slc25a22;Slc25a18	12	5	16.1	6	0	9.1386	6515700	14655000	2196600	Slc25a22	0.444605937	Slc25a22;Slc25a18	Slc25a22	323	Mitochondrial glu	13	10090.ENSMUSP00000019226
68682	A0A1L1SVG6;Q	Slc44a2	3	2	3.4	5	0	4.569	10304000	3693300	766890	Slc44a2	2.789916877	Slc44a2	Slc44a2	706	Choline transporte	15	10090.ENSMUSP00000034697
68693	Q00PI9	Hnrnpul2	1	5	8.3	5	0	9.003	25087000	3457200	989670	Hnrnpul2	7.256450307	Hnrnpul2	Hnrnpul2	745	Heterogeneous nu	2	10090.ENSMUSP00000094515
68760	B2RQK7;Q8BW	Synpo2l	3	14	19.3	22	0	31.756	52600000	18392000	3997300	Synpo2l	2.859939104	Synpo2l	Synpo2l	975	Synaptopodin 2-li	1	10090.ENSMUSP00000053176
69162	Q3UPL0-2;Q3U	Sec31a	5	5	5	6	0	8.9449	23253000	4355700	711450	Sec31a	5.338521937	Sec31a	Sec31a	1230	Protein transport p	2	10090.ENSMUSP00000092157
69178	Q9D8U8	Snx5	1	2	8.4	3	0	5.3466	8217100	2064300	539340	Snx5	3.980574529	Snx5	Snx5	404	Sorting nexin-5; In	2	10090.ENSMUSP00000105657
69202	Q9D0J8	Ptms	1	2	22.8	5	0	4.7617	28418000	7574100	15097000	Ptms	3.751996937	Ptms	Ptms	101	Parathymosin; Par	5	10090.ENSMUSP00000032216
69288	E9PZ72;Q9DAK	Rhobtb1;Rhobtb2	8	2	3.2	4	0.003	1.6481	25496000	12144000	1987400	Rhobtb1	2.099472991	Rhobtb1;Rhobtb2	Rhobtb1	695	Rho-related BTB d	1	10090.ENSMUSP00000020101
69875	G5E814;Q9D8B	Ndufa11	4	3	35	12	0	22.981	47499000	104340000	49636000	Ndufa11	0.455232892	Ndufa11	Ndufa11	143	Accessory subuni	3	10090.ENSMUSP00000002452
70257	P56379;A0A1Y	Mp68	2	2	29.3	13	0	8.9306	218310000	41271000	137970000	Mp68	5.289670713	Atp5mpl	Mp68	58	6.8 kDa mitochond	3	10090.ENSMUSP00000021719
71514	Q8VIJ6	Sfpq	2	7	13.2	12	0	12.959	48140000	20609000	3250600	Sfpq	2.335872677	Sfpq	Sfpq	699	Splicing factor, pr	2	10090.ENSMUSP00000030623
71679	Q9DCX2;B1ASI	Atp5h	2	14	64	80	0	100.07	1093200000	2468900000	558110000	Atp5h	0.442788286	Atp5h;Atp5pd	Atp5h	161	ATP synthase sub	3	10090.ENSMUSP00000102147
71770	Q9DBG3;Q9DB	Ap2b1	5	4	12.3	13	0	18.049	59852000	27174000	2758200	Ap2b1	2.202546552	Ap2b1	Ap2b1	951	AP-2 complex sub	2	10090.ENSMUSP00000018875
71853	Q3TML0;Q922F	Pdia6	2	5	14.6	10	0	26.175	44125000	12398000	5006400	Pdia6	3.559041781	Pdia6	Pdia6	445	May function as a	2	10090.ENSMUSP00000052912
71941	G3X975;Q8BYM	Cars2	3	2	5.1	4	0	4.202	1033800	2362400	298580	Cars2	0.437605825	Cars2	Cars2	552	Probable cysteine	2	10090.ENSMUSP00000046453
72039	Q99MR8	Mccc1	3	15	26.6	29	0	37.415	58028000	118790000	8281900	Mccc1	0.488492297	Mccc1	Mccc1	717	Methylcrotonoyl-	3	10090.ENSMUSP00000029259
72042	Q9CQI6;A0A1D	Cotl1	2	3	29.6	4	0	3.4299	28713000	2439600	3834800	Cotl1	11.76955239	Cotl1	Cotl1	142	Coactosin-like pro	1	10090.ENSMUSP00000034285
72333	Q9ET54-3;Q9ET	Palld	7	5	9	10	0	9.2212	35183000	15095000	2648600	Palld	2.330771779	Palld	Palld	1408	Palladin; Cytoskel	1	10090.ENSMUSP00000112442
72560	G3UWC2;Q9CZ	Naalad2	2	5	7.8	7	0	10.499	22584000	1254900	729200	Naalad2	17.99665312	Naalad2	Naalad2	740	N-acetylated-alpha	7	10090.ENSMUSP00000128674
72590	Q8BVQ5;A0A1	Ppme1	2	2	5.2	2	0.001	2.1633	817440	2737900	547380	Ppme1	0.298564593	Ppme1	Ppme1	386	Protein phosphata	5	10090.ENSMUSP00000032963
73095	Q8R0Y8	Slc25a42	1	2	5.3	2	0.001	1.8073	5118000	15482000	2142900	Slc25a42	0.330577445	Slc25a42	Slc25a42	318	Mitochondrial coe	3	10090.ENSMUSP00000105754
73333	Q3V132	Slc25a31	1	2	13.8	1	0.001	2.1662	2757900	14924000	1140600	Slc25a31	0.184796301	Slc25a31	Slc25a31	320	ADP/ATP transloc	3	10090.ENSMUSP00000088723
73723	Q91VW3;I7HPY	Sh3bgrl3	2	2	18.3	4	0	3.1668	85688000	9220300	28692000	Sh3bgrl3	9.293406939	Sh3bgrl3	Sh3bgrl3	93	SH3 domain-bindi	1	10090.ENSMUSP00000030651
74117	Q99JY9	Actr3	6	6	17.5	8	0	11.973	31473000	8477300	2447300	Actr3	3.712620764	Actr3	Actr3	418	Actin-related prot	1	10090.ENSMUSP00000137503
74122	Q9DBS1	Tmem43	1	2	5	3	0.001	1.922	9586100	1503600	582840	Tmem43	6.375432296	Tmem43	Tmem43	400	Transmembrane pr	1	10090.ENSMUSP00000032183
74238	Q8BKY8	Mterf2	1	2	5.2	2	0	3.0639	2081300	4335300	547100	Mterf2	0.480082117	Mterf2	Mterfd3	385	Transcription term	22	10090.ENSMUSP00000062762
74325	Q6IRU5-2;Q6IR	Cltb	4	5	20.9	8	0	12.895	41194000	19775000	11529000	Cltb	2.083135272	Cltb	Cltb	211	Clathrin light chair	2	10090.ENSMUSP00000089198
75475	Q8K010;E9Q484	Oplah	4	4	3.7	5	0	10.237	3916500	8112000	364260	Oplah	0.482803254	Oplah	Oplah	1288	5-oxoprolinase; Ca	8	10090.ENSMUSP00000129100
76263	Q9DCM2;A0A0	Gstk1	2	8	44.2	22	0	47.606	48621000	121880000	17686000	Gstk1	0.398925172	Gstk1	Gstk1	226	Glutathione S-tran	3	10090.ENSMUSP00000031897
76491	A0A087WP24;A	Abhd14b	6	4	29.6	5	0	3.7559	31103000	5949200	6625400	Abhd14b	5.228097896	Abhd14b	Abhd14b	210	Has hydrolase act	16	10090.ENSMUSP00000038755
76709	Q9CVB6;D3YX	Arpc2	3	10	31.7	14	0	21.484	68617000	21900000	6396100	Arpc2	3.133196347	Arpc2	Arpc2	300	Actin-related prot	1	10090.ENSMUSP00000006467
76936	B8JK33;B8JK32	Hnrnpm	7	7	12.8	9	0	10.451	43981000	13935000	1663300	Hnrnpm	3.15615357	Hnrnpm	Hnrnpm	729	Heterogeneous nu	2	10090.ENSMUSP00000120115
77579	Q5SV64;Q3UH5	Myh10	4	17	18.4	22	0	53.615	106870000	7957400	1309100	Myh10	13.43026617	Myh10	Myh10	1976	Myosin-10; Involv	1	10090.ENSMUSP00000099671
77605	Q3THW5;P0C0	H2afv;H2afz	4	2	31.2	4	0	4.7453	61028000	24564000	22398000	H2afv	2.484448787	H2az1;H2az2	H2afv	128	Histone H2A.V; V	1	10090.ENSMUSP00000105359
78038	Q3ULD5;Q6PD	Mccc2	2	15	30.9	29	0	59.211	84215000	171380000	15630000	Mccc2	0.491393395	Mccc2	Mccc2	563	Methylcrotonoyl-	3	10090.ENSMUSP00000022148
78388	E9Q3X0;Q9EQK	Mvp	3	20	24.9	31	0	33.158	108880000	38921000	5044700	Mvp	2.797461525	Mvp	Mvp	870	Major vault protei	6	10090.ENSMUSP00000127250
78655	Q3UGC7;Q66JS	Eif3j1;Eif3j2	2	3	13	5	0	6.4449	12387000	4879500	2002900	Eif3j1	2.538579773	Eif3j1;Eif3j2	Eif3j1	261	Eukaryotic transla	2	10090.ENSMUSP00000028668
78920	Q9D2G2;Q9D2C	Dlst	2	9	23.6	44	0	49.988	493010000	1013400000	103440000	Dlst	0.48649102	Dlst	Dlst	454	Dihydrolipoamide	3	10090.ENSMUSP00000060346
81910	A2AVJ7;Q99PL	Rrbp1	13	16	11.6	21	0	35.989	91045000	19994000	1390700	Rrbp1	4.553616085	Rrbp1	Rrbp1	1464	Ribosome-binding	6	10090.ENSMUSP00000016072
83493	A0A5F8MPK9;	Sacm1l	2	6	9.4	7	0	7.1104	21451000	10507000	1518700	Sacm1l	2.04159132	Sacm1l	Sacm1l	587	Phosphatidylinosi	2	10090.ENSMUSP00000026270
83997	F6UV57;F6YCM	Slmap	10	11	23.8	18	0	54.758	99659000	49229000	8879200	Slmap	2.024396189	Slmap	Slmap	845	Sarcolemmal meml	3	10090.ENSMUSP00000117816
94280	Q3U4F0;Q91V6	Sfxn3	4	2	10	3	0	8.4182	2453400	1210000	411380	Sfxn3	2.027603306	Sfxn3	Sfxn3	321	Sideroflexin-3; Pot	21	10090.ENSMUSP00000059419
97212	Q8BMS1	Hadha	1	34	55.6	144	0	295.74	3667200000	7577100000	494510000	Hadha	0.483984638	Hadha	Hadha	763	Trifunctional enzy	3	10090.ENSMUSP00000120976
98238	Q922Q8	Lrrc59	1	4	14.7	5	0	6.4626	12409000	3876400	1491400	Lrrc59	3.20116603	Lrrc59	Lrrc59	307	Leucine-rich repea	2	10090.ENSMUSP00000021239
