# Supplementary material for: Comparative Proteomic and Phosphoproteomic Analyses Reveal Molecular Signatures of Myocardial Infarction and Transverse Aortic Constriction in Aged Mouse Models
Source: Cardiol Res Pract. 2024 Oct 28;2024:9395213. doi: 10.1155/2024/9395213 (PMC11535427; doi:10.1155/2024/9395213)
Supplement: Supporting Information — Table S3: List of significant differentially expressed proteins (DEPs) in TAC vs sham. [file 9395213.f3.pdf]

ENTREZID	Majority.protein.IDs	Gene.names	Number.of.proteins	Unique.peptides	Sequence.coverage....	MS.MS.count	Q.value	Score	LFQ.intensity.sham	LFQ.intensity.TAC	iBAQ	Symbol	fold change(TAC/sham)	Alias	preferred_name	protein_size	annotation	SubPPI	STRING_id
	A0A338P6I9		1	2	39	2	0	3.4208	2250000	1044300	1284300	A0A338P6I9	0.464133333	Gm56451					
	F7CVJ5;F7DBB3;E9PYB0	Ahnak2	3	4	20.3	5	0	10.214	2377100	5366600	625270	Ahnak2	2.257624837						
	P0DN34		1	2	31.6	8	0	4.9085	416800000	203420000	322000000	P0DN34	0.488051823	Ndufb1					
102866	A0A1C7CYV0;B1AX58;Q99K51	Pls3	4	10	24.3	12	0	37.061	11221000	32403000	2983200	Pls3	2.887710543	Pls3	Pls3	630	Plastin-3; Actin-bu	1	10090.ENSMUSP00000033547
104027	E9Q3E2;Q8CC35-3;Q8CC35-2;Q8CC35	Synpo	4	4	3.9	5	0	4.6398	14908000	29860000	1116600	Synpo	2.002951435	Synpo	Synpo	901	Synaptopodin; Acti	1	10090.ENSMUSP00000095174
104158	Q8VCT4	Ces1d	1	3	11	4	0	5.3547	18081000	5442500	1327300	Ces1d	0.301006581	Ces1d	Ces1d	565	Carboxylesterase 1I	3	10090.ENSMUSP00000034172
109624	Q8VCQ8;S4R1T7;E9Q0M9;E9QA16;D3Z6I7;E9QA15	Cald1	11	9	19.8	13	0	21.44	4742900	19523000	2969100	Cald1	4.116257986	Cald1	Cald1	530	Caldesmon 1	1	10090.ENSMUSP00000110673
109731	Q8BW75	Maob	3	11	25	13	0	26	60466000	27352000	5676600	Maob	0.452353389	Maob	Maob	520	Amine oxidase [fla	3	10090.ENSMUSP00000040550
110826	Q9DCW4;A0A0U1RNP5;A0A0N4SVE0;A0A0U1RNR3;A0A0N4SWE9;A0A0U1RNK9	Etfb	7	15	56.9	120	0	142.23	4464700000	2080100000	779100000	Etfb	0.465899165	Etfb;Etfbl	Etfb	255	Electron transfer fla	3	10090.ENSMUSP00000004729
11421	P09470;P09470-2;F6QCP8	Ace	3	6	4.9	8	0	12.166	7776300	27618000	932060	Ace	3.551560511	Ace	Ace	1312	Angiotensin-conver	2	10090.ENSMUSP00000001963
114584	Q9Z1Q5	Clic1	1	4	23.2	5	0	8.4517	3032400	10493000	2018400	Clic1	3.460295476	Clic1	Clic1	241	Chloride intracellul	1	10090.ENSMUSP00000007257
11459	P68134	Acta1	1	2	70	11	0	80.414	16316000	142210000	8601100	Acta1	8.71598431	Acta1	Acta1	377	Actin, alpha skeleta	1	10090.ENSMUSP00000034453
114886	Q9CX80	Cygb	1	3	16.3	3	0	4.0879	943320	2708900	1483400	Cygb	2.871666031	Cygb	Cygb	190	Cytoglobin; May h	13	10090.ENSMUSP00000021166
11603	Z4YK85;M0QWP1;A2ASQ1-3;A2ASQ1-2;A2ASQ1	Agrn	5	3	2.1	2	0	6.0137	2458900	4945400	124820	Agrn	2.011224531	Agrn	Agrn	2034	Agrin; Isoform 1: I	5	10090.ENSMUSP00000137931
11745	O35639;Q3TET3	Anxa3	4	6	20.4	12	0	16.745	19630000	43432000	7022800	Anxa3	2.212531839	Anxa3	Anxa3	323	Annexin A3; Inhibi	1	10090.ENSMUSP00000031447
11746	A0A0N4SW89;P97429;D3Z0S1;A0A0N4SV57;S4R1F2;F7ANV6	Anxa4	6	7	30.4	12	0	23.324	14737000	40109000	6018900	Anxa4	2.721652982	Anxa4	Anxa4	319	Annexin A4; Calciu	1	10090.ENSMUSP00000109305
12040	Q6P3A8-2;Q6P3A8	Bckdhb	2	4	14.3	6	0	7.6017	31775000	12599000	6008300	Bckdhb	0.396506688	Bckdhb	Bckdhb	390	2-oxoisovalerate de	3	10090.ENSMUSP00000139684
12111	P28653	Bgn	1	13	46.3	31	0	55.991	33143000	113560000	57297000	Bgn	3.426364542	Bgn	Bgn	369	Biglycan; May be i	5	10090.ENSMUSP00000033741
12304	A0A0R4J0Z1;P08003	Pdia4	2	7	13.3	9	0	10.766	8712000	18540000	1912600	Pdia4	2.128099174	Pdia4	Pdia4	641	Protein disulfide-is	4	10090.ENSMUSP00000076521
12321	Q6XLQ8;O35887;G3UWV3;G3V004	Calu	8	5	16.2	7	0	24.662	3892300	18277000	3230000	Calu	4.695681217	Calu	Calu	315	Calumenin; Involve	4	10090.ENSMUSP00000031779
12539	A0A1L1STC0;Q61081	Cdc37	2	3	8.2	5	0	3.7811	7019200	14165000	2178000	Cdc37	2.018036243	Cdc37	Cdc37	379	Hsp90 co-chaperone	2	10090.ENSMUSP00000019615
12759	Q06890;E9PUU2;E9PXG5;E9Q8Y5	Clu	6	11	27	17	0	35.443	28096000	65700000	11853000	Clu	2.338411162	Clu	Clu	448	Clusterin; Functior	2	10090.ENSMUSP00000022616
12842	P11087-2;P11087	Col1a1	3	6	5.7	8	0	17.202	4630400	11205000	2275600	Col1a1	2.419877332	Col1a1	Col1a1	1453	Collagen alpha-1(I)	5	10090.ENSMUSP00000001547

13003	Q8BS97;E9QMK3;E9QMK2;G3XA35;E9PYH0;Q62059-4;Q62059-3;Q62059-2;Q62059	Vcan	9	3	10.9	4	0	4.3859	1204900	5820500	1022100	Vcan	4.830691344	Vcan	Vcan	3354	May play a role in	5	10090.ENSMUSP00000105173
13010	A2APX3;P21460	Cst3	2	2	26.5	2	0	3.0497	1088100	2522200	1719300	Cst3	2.317985479	Cst3	Cst3	140	Cystatin-C; As an i	2	10090.ENSMUSP00000028938
13177	P42125;A0A3Q4EC00;A0A452J8A5	Eci1	4	10	39.1	53	0	46.799	1113200000	507580000	145960000	Eci1	0.455964786	Eci1	Eci1	289	Enoyl-CoA delta is	3	10090.ENSMUSP00000024946
13427	A2BFF8;Q3TPJ8;A2BFF5;A2BFF9;O88487	Dync1i2	5	2	3.1	4	0	3.4521	10906000	25618000	3047600	Dync1i2	2.348982212	Dync1i2	Dync1i2	612	Cytoplasmic dynei	2	10090.ENSMUSP00000080410
140781	Q91Z83	Myh7	5	36	64	193	0	323.31	401150000	8048400000	105680000	Myh7	20.06331796	Myh7	Myh7	1935	Myosin-7; Myosin	6	10090.ENSMUSP00000099867
14086	Q61553;A0A0G2JDU7	Fscn1	3	7	18.1	7	0	9.569	8659800	24566000	2126300	Fscn1	2.836786069	Fscn1	Fscn1	493	Fascin; Organizes f	1	10090.ENSMUSP00000031565
14151	F6T2S0;Q91W09;A0A494BAI5;Q544X6;P22315;A0A494B9Y8;A0A494BBL4	Fech	8	4	12.9	4	0	5.1602	22933000	4251700	2293400	Fech	0.18539659	Fech	Fech	422	Ferrochelatase, mit	2	10090.ENSMUSP00000025484
14199	A2AEX8;A2AEX6;P97447;A2AEY1;A2AEY2;A2AEX7;P97447-2	Fhl1	10	10	38.5	29	0	31.346	38213000	222500000	18510000	Fhl1	5.822625808	Fhl1	Fhl1	323	Four and a half LI	6	10090.ENSMUSP00000110417
14376	Q8BHN3;Q8BHN3-2;Q8BHN3-3	Ganab	3	8	8.4	10	0	13.783	6810100	18199000	908240	Ganab	2.672354297	Ganab	Ganab	966	Neutral alpha-gluco	4	10090.ENSMUSP00000093965
15381	Q9Z204-4;Q9Z204-3;Q9Z204-2;Q9Z204-5;Q9Z204	Hnrnpc	7	5	20.5	5	0	9.3673	7376100	14922000	3611600	Hnrnpc	2.023020295	Hnrnpc	Hnrnpc	313	Heterogeneous nucl	8	10090.ENSMUSP00000107237
15388	G5E924;Q8R081;G3UY38	Hnrnpl	3	4	7	7	0	8.1682	10090000	20803000	2512200	Hnrnpl	2.061744301	Hnrnpl	Hnrnpl	615	Heterogeneous nucl	8	10090.ENSMUSP00000134734
15507	P14602-2;P14602;P14602-3;D3YZ06	Hspb1	4	10	61.4	33	0	61.159	220550000	521900000	73125000	Hspb1	2.366356835	Hspb1	Hspb1	209	Heat shock protein	2	10090.ENSMUSP00000005077
15519	P07901	Hsp90aa1	4	13	28.9	29	0	45.661	45478000	92427000	7345100	Hsp90aa1	2.03234531	Hsp90aa1	Hsp90aa1	733	Heat shock protein	2	10090.ENSMUSP00000091921
16427	A0A2K6EDJ7;E9Q5L2;E9PVD2;A6X935-2;A6X935	Itih4	5	7	8.4	10	0	10.453	11448000	26014000	1243000	Itih4	2.272361985	Itih4	Itih4	942	Inter alpha-trypsin	4	10090.ENSMUSP00000006703
16549	A0A3B2WCD8;Q3U0V1	Khsrp	3	3	7.9	5	0	3.8302	6351900	17804000	1445200	Khsrp	2.802940852	Khsrp	Khsrp	748	Far upstream eleme	8	10090.ENSMUSP00000007814
16952	P10107;A0A494BBD8	Anxa1	7	9	26.6	11	0	37.857	12917000	27347000	6961100	Anxa1	2.117132461	Anxa1	Anxa1	346	Annexin A1; Plays	1	10090.ENSMUSP00000025561
17022	P51885	Lum	2	10	36.1	17	0	83.28	36575000	104590000	56313000	Lum	2.859603554	Lum	Lum	338	Lumican; Belongs	5	10090.ENSMUSP00000040877
170728	A0A0R4J0S3;Q924D0	Rtn4ip1	2	3	8.3	4	0	5.0507	22079000	7334100	2537500	Rtn4ip1	0.33217537	Rtn4ip1	Rtn4ip1	396	Reticulon-4-interac	10	10090.ENSMUSP00000060940
17698	P26041	Msn	2	18	35.9	54	0	56.48	219470000	451880000	36346000	Msn	2.058960222	Msn	Msn	577	Moesin; Probably i	1	10090.ENSMUSP00000113071
17901	E9PWG4;P05977-2;P05977;E0CZ30	Myl1	4	3	37.3	5	0	7.9061	3034600	8971100	1889400	Myl1	2.956271008	Myl1	Myl1	188	Myosin light chain	6	10090.ENSMUSP00000027151
17904	A0A1W2P6F6;A0A1W2P7Q9;Q60605-2;Q60605;A0A1W2P6G5	Myl6	6	6	39.5	13	0	14.377	27303000	61867000	26456000	Myl6	2.265941472	Myl6	Myl6	151	Myosin light poly	1	10090.ENSMUSP00000128803

17955	B7ZNL2;Q78ZA7	Nap1l4	4	4	17.6	9	0	7.171	5461300	14260000	2771500	Nap1l4	2.611099921	Nap1l4	Nap1l4	375	Nucleosome assem	4	10090.ENSMUSP00000072510
18000	E9Q3V6;P42208;F6WYM0;D3YYB1;D3Z3C0	Sept2	10	7	26.5	12	0	16.941	14395000	34662000	5879500	Sept2	2.407919416	Septin2	Sept2	361	Septin-2; Filament	8	10090.ENSMUSP00000027495
18175	Q80XB4-2;Q80XB4;E9PZF4;Q80XB4-4;Q80XB4-3;E9Q641	Nrap	7	41	25.8	55	0	83.524	46054000	312490000	3324600	Nrap	6.785295523	Nrap	Nrap	1728	Nebulin-related-anc	6	10090.ENSMUSP00000073228
18220	Q02819;A0A1B0GR41;A0A1C7CYU3	Nucb1	5	5	11.1	6	0	12.665	2639700	8141200	996670	Nucb1	3.084138349	Nucb1	Nucb1	459	Nucleobindin-1; M	4	10090.ENSMUSP00000033096
18295	Q62000	Ogn	1	8	27.2	13	0	12.488	15415000	40435000	29319000	Ogn	2.623094389	Ogn	Ogn	298	Mimecan; Induces	5	10090.ENSMUSP00000021822
18453	P09103	P4hb	2	18	37.3	37	0	66.944	98188000	197070000	19289000	P4hb	2.007068073	P4hb	P4hb	509	Protein disulfide-is	4	10090.ENSMUSP00000026122
18826	Q61233	Lcp1	5	12	26.5	21	0	52.367	21887000	57452000	7410200	Lcp1	2.624937177	Lcp1	Lcp1	627	Plastin-2; Actin-bi	1	10090.ENSMUSP00000116271
192176	B7FAV1;B7FAU9;Q8BTM8	Flna	7	31	17.8	38	0	76.518	22324000	107510000	2522700	Flna	4.815893209	Flna	Flna	2647	Filamin-A; Actin b	1	10090.ENSMUSP00000033699
19659	Q00915	Rbp1	1	6	47.4	7	0	49.421	2182400	6705200	7562900	Rbp1	3.072397361	Rbp1	Rbp1	135	Retinol-binding pr	3	10090.ENSMUSP00000059749
20195	P50543	S100a11	1	2	20.4	4	0	11.343	8824300	22029000	19885000	S100a11	2.496401981	S100a11	S100a11	98	Protein S100-A11;	1	10090.ENSMUSP00000029515
20200	P14069	S100a6	1	3	25.8	4	0	3.8432	27294000	73566000	48108000	S100a6	2.695317652	S100a6	S100a6	89	Protein S100-A6; M	1	10090.ENSMUSP00000001051
20340	F8WHM5;Q61543	Glg1	3	3	3	5	0	4.8828	1829400	5701000	259640	Glg1	3.116322291	Glg1	Glg1	1175	Golgi apparatus pr	30	10090.ENSMUSP00000131355
20928	E9PUE8;P70170-3;P70170-2;P70170	Abcc9	4	2	1.6	1	0.0049	1.4266	4947100	2125500	156440	Abcc9	0.429645651	Abcc9	Abcc9	1546	ATP-binding casse	1	10090.ENSMUSP00000084805
210582	A0A1W2P7G5;A0A1W2P7J1;E9Q3H6	Coq10a	7	6	27.6	12	0	12.983	77108000	37074000	11929000	Coq10a	0.480806142	Coq10a	Coq10a	259	Coenzyme Q10 ho	11	10090.ENSMUSP00000036213
21345	P37804;A0A1L1STN8	Tagln	2	10	52.7	20	0	36.608	32763000	75041000	14392000	Tagln	2.29041907	Tagln	Tagln	201	Transgelin; Actin c	1	10090.ENSMUSP00000034590
21346	Q9WVA4	Tagln2	3	12	62.8	26	0	47.783	66022000	145970000	37894000	Tagln2	2.21092969	Tagln2	Tagln2	199	Transgelin-2; Trans	1	10090.ENSMUSP00000106861
216197	Q8BMK4	Ckap4	1	13	25.4	21	0	50.591	17251000	50213000	4726900	Ckap4	2.910729813	Ckap4	Ckap4	575	Cytoskeleton-assoc	1	10090.ENSMUSP00000050336
216616	Q8BPB5	Efemp1	2	6	14.6	8	0	7.4773	797120	4593200	1477600	Efemp1	5.762244079	Efemp1	Efemp1	493	EGF-containing fib	5	10090.ENSMUSP00000020759
217830	E9QMK9;Q8BH86-2;Q8BH86	9030617O03Rik	3	6	11.9	8	0	10.779	21630000	5901700	1315300	9030617O03Rik	0.272847896	Dglucy	Dglucy	617	D-glutamate cyclas	4	10090.ENSMUSP00000067830
22042	Q62351;Q8C872	Tfrc	2	6	8.1	6	0	7.484	3353000	18782000	714140	Tfrc	5.60155085	Tfrc	Tfrc	763	Transferrin receptor	2	10090.ENSMUSP00000023486
22223	Q9R0P9	Uchl1	1	5	24.7	9	0	9.718	12711000	26658000	8735800	Uchl1	2.097238612	Uchl1	Uchl1	223	Ubiquitin carboxyl-	2	10090.ENSMUSP00000031131
22240	E9PWE8;Q3TT92;Q62188	Dpysl3	3	11	28.8	13	0	24.167	8512700	18993000	2589300	Dpysl3	2.231137007	Dpysl3	Dpysl3	570	Dihydropyrimidina	1	10090.ENSMUSP00000025379
22352	P20152;A0A0A6YWC8	Vim	21	30	70.2	128	0	254.27	1077100000	2795300000	364740000	Vim	2.595209358	Vim	Vim	466	Vimentin; Vimenti	1	10090.ENSMUSP00000028062
22436	Q00519	Xdh	1	3	2.8	6	0	5.2874	6810400	3031400	408860	Xdh	0.445113356	Xdh	Xdh	1335	Xanthine dehydrog	2	10090.ENSMUSP00000024866
22437	A0A571BDS0;E9QQ93;O70373	Xirp1	3	17	12	28	0	37.686	20722000	101060000	1547200	Xirp1	4.87694238	Xirp1	Xirp1	1132	Protects actin filam	6	10090.ENSMUSP00000107262
224796	Q8BXK9	Clic5	1	3	13.5	4	0	4.0294	5081700	12497000	1764800	Clic5	2.459216404	Clic5	Clic5	251	Chloride intracellul	1	10090.ENSMUSP00000024755
224904	Q8R404	Qil1	1	2	24.4	2	0	10.731	24030000	10117000	19039000	Qil1	0.421015397	Micos13	Mic13	119	MICOS complex s	3	10090.ENSMUSP00000052908

228859	P59266	Fitm2	1	2	8.4	2	0	3.8091	10364000	4862900	4158500	Fitm2	0.469210729	Fitm2	Fitm2	262	Fat storage-inducin	26	10090.ENSMUSP00000105045
233489	Q7M6Y3-2;Q7M6Y3-3;Q7M6Y3-4;Q7M6Y3-6;Q7M6Y3-5;Q7M6Y3;A0A1L1SUR7;A0A140LHQ8	Picalm	9	3	5.7	4	0	5.5666	4934800	11215000	1217000	Picalm	2.272635163	Picalm	Picalm	660	Phosphatidylinosit	2	10090.ENSMUSP00000051092
234214	Q3UTJ2-2;A0A0R4J1T1;Z4YJR7;B9EKP8	Sorbs2	4	2	24.5	49	0	51.08	136500000	294170000	8581300	Sorbs2	2.155091575	Sorbs2	Sorbs2	652	Sorbin and SH3 do	28	10090.ENSMUSP00000121619
23790	Q9WUM4	Coro1c	4	6	14.3	8	0	8.7155	3228500	9471500	1791800	Coro1c	2.933715348	Coro1c	Coro1c	474	Coronin-1C; May l	1	10090.ENSMUSP00000004646
23876	A0A1Y7VJW9;Q9WVH9	Fbln5	2	11	25.6	18	0	49.189	12174000	27039000	15410000	Fbln5	2.22104485	Fbln5	Fbln5	448	Fibulin-5; Essentia	5	10090.ENSMUSP00000021603
23950	A0A0G2JER9;G3X8S5;O54946-2;O54946;A0A0G2JGN9;A0A0G2JEI3;Q9QYI8	Dnajb6;Dnajb7	7	2	9.9	4	0	2.9137	2324200	5029400	1135500	Dnajb6	2.163927373	Dnajb6;Dnajb7	Dnajb6	365	DnaJ homolog sub	6	10090.ENSMUSP00000008733
241431	A0A5F8MPR4;Q4U4S6-2;Q4U4S6	Xirp2	7	32	10.3	40	0	58.737	3753700	225400000	932600	Xirp2	60.04741988	Xirp2	Xirp2	3283	Xin actin-binding r	6	10090.ENSMUSP00000107966
246221	Q99J99;A0A2R8VHE5;A0A2R8VHT1	Mpst	3	2	7.4	1	0	2.4437	11765000	5014200	1501500	Mpst	0.426196345	Mpst	Mpst	297	Transfer of a sulfur	20	10090.ENSMUSP00000043061
26457	Q60714	Slc27a1	1	2	3.3	4	0	3.0469	6763700	3345800	518810	Slc27a1	0.494670077	Slc27a1	Slc27a1	646	Long-chain fatty ac	17	10090.ENSMUSP00000034267
27061	Q61335	Bcap31	1	3	11.8	5	0	8.4924	11169000	36055000	6195900	Bcap31	3.228131435	Bcap31	Bcap31	245	B-cell receptor-asso	4	10090.ENSMUSP00000002091
27367	P27659;A0A2R8VHN4	Rpl3	4	6	17.6	10	0	10.219	11779000	49174000	8394400	Rpl3	4.174717718	Rpl3	Rpl3	403	60S ribosomal prot	2	10090.ENSMUSP00000080354
27979	Q8JZQ9	Eif3b	2	5	6.4	5	0	9.2445	2236700	4999900	458550	Eif3b	2.235391425	Eif3b	Eif3b	803	Eukaryotic translati	2	10090.ENSMUSP00000098076
27984	Q8C845;Q9D8Y0	Efhd2	2	4	17.1	7	0	14.975	5859900	21404000	4193300	Efhd2	3.652622058	Efhd2	Efhd2	240	May regulate B-cell	6	10090.ENSMUSP00000044502
29818	P35385	Hspb7	1	3	26	9	0	6.5468	22405000	53812000	11185000	Hspb7	2.401785316	Hspb7	Hspb7	169	Heat shock protein	6	10090.ENSMUSP00000099544
30937	Q8VEE1	Lmcd1	1	3	9.9	5	0	6.0788	3067000	6933100	696020	Lmcd1	2.260547767	Lmcd1	Lmcd1	365	LIM and cysteine-ri	16	10090.ENSMUSP00000032376
321022	A0A087WNP6;Q4VAA2-2;Q4VAA2	Cdv3	3	2	12.8	3	0	5.4383	2582100	12760000	1714500	Cdv3	4.941714109	Cdv3	Cdv3	281	Protein CDV3; Ca	6	10090.ENSMUSP00000044420
50706	Q62009;Q62009-3;Q62009-4;Q62009-2;Q62009-5	Postn	7	20	30.5	38	0	154.9	2428500	33858000	19385000	Postn	13.94193947	Postn	Postn	811	Periostin; Induces	5	10090.ENSMUSP00000072773
51798	O35459;F7B227	Ech1	2	13	48.9	38	0	140.53	1277200000	600380000	148550000	Ech1	0.470075164	Ech1	Ech1	327	Delta(3,5)-Delta(2,4	3	10090.ENSMUSP00000066092
52377	A0A1B0GSK5;A0A1B0GR19;A0A1B0GR86;Q8BH97;A0A1B0GS22	Rcn3	6	4	23.7	4	0	19.372	439910	1590700	4291500	Rcn3	3.615966902	Rcn3	Rcn3	328	Reticulocalbin-3; R	7	10090.ENSMUSP00000019683
52538	Q8BWT1	Acaa2	2	23	71	135	0	298.24	5128200000	1732300000	490020000	Acaa2	0.337798838	Acaa2	Acaa2	397	3-ketoacyl-CoA thi	3	10090.ENSMUSP00000037348
54132	O70400;S4R1V0	Pdlim1	2	11	33.9	21	0	21.347	29282000	79911000	11151000	Pdlim1	2.729014412	Pdlim1	Pdlim1	327	PDZ and LIM dom	1	10090.ENSMUSP00000064545
54473	Q9QZ06;Q8C5G6;A9JEI5;F7AT44	Tollip	4	4	15.3	4	0	5.7481	14381000	1343200	2404500	Tollip	0.093401015	Tollip	Tollip	274	Toll-interacting pro	12	10090.ENSMUSP00000001950

56295	A0A1L1ST61;A0A1L1SV00;Q8R472;Q9JLR9;A0A1L1SR69	Higd1a	5	2	35.5	6	0	4.9797	1948100	11269000	5961700	Higd1a	5.784610646	Higd1a	Higd1a	95	HIG1 domain famil	21	10090.ENSMUSP00000054881
56421	Q8C605;Q9WUA3;Q9WUA3-2	Pfkp	4	4	9.4	7	0	7.6366	4555900	13254000	782840	Pfkp	2.909194671	Pfkp	Pfkp	784	"ATP-dependent 6-	3	10090.ENSMUSP00000117030
56430	D3Z2Z1;F8WIA1;Q922J3;D3Z3M7;Q922J3-2;Q8C0S5;F6RCU2	Clip1	10	10	7.4	12	0	18.593	13374000	27448000	689100	Clip1	2.052340362	Clip1	Clip1	1391	CAP-Gly domain-c	2	10090.ENSMUSP00000107192
56433	D3YYD5;D3Z645;Q9QZ88;Q9QZ88-2	Vps29	5	3	25	4	0	3.5979	7877800	20091000	3714100	Vps29	2.550331311	Vps29	Vps29	182	Vacuolar protein so	27	10090.ENSMUSP00000121020
56463	Q78PY7	Snd1	2	6	7.1	9	0	9.0634	5930400	20473000	976340	Snd1	3.45221233	Snd1	Snd1	910	Staphylococcal nuc	7	10090.ENSMUSP00000001460
56702	P43276	Hist1h1b	1	6	32.7	10	0	9.031	25246000	73377000	30404000	Hist1h1b	2.906480234	H1-5	Hist1h1b	223	Histone H1.5; Hist	23	10090.ENSMUSP00000079356
56752	Q3U367;Q9JLJ2	Aldh9a1	2	3	6.2	3	0	4.4569	2487600	18758000	915570	Aldh9a1	7.540601383	Aldh9a1	Aldh9a1	518	4-trimethylaminobu	3	10090.ENSMUSP00000028004
57423	P56135;F8WHP8	Atp5j2	2	4	36.4	10	0	9.657	365970000	164710000	208960000	Atp5j2	0.450064213	Atp5j2;Atp5mf	Atp5j2	88	ATP synthase subu	3	10090.ENSMUSP00000125504
59029	O35593	Psmd14	1	2	6.5	2	0	2.8685	5929100	12156000	1843300	Psmd14	2.050226847	Psmd14	Psmd14	310	26S proteasome no	2	10090.ENSMUSP00000028278
60595	A0A1L1SV25;P57780;E9Q2W9	Actn4	6	14	36.3	21	0	26.241	29845000	62330000	3789300	Actn4	2.088457028	Actn4	Actn4	912	Alpha-actinin-4; F-	1	10090.ENSMUSP00000066068
66152	Q8R1I1	Uqcr10	1	3	50	20	0	13.355	1003500000	466710000	581030000	Uqcr10	0.465082212	Uqcr10	Uqcr10	64	Cytochrome b-c1 c	3	10090.ENSMUSP00000054856
66433	B1AXC8;Q8K2Q5;Q3TFT3;B1AXC7;B1AXC5	Chchd7	5	2	34.2	2	0	6.2439	8245200	1100800	3026100	Chchd7	0.13350798	Chchd7	Chchd7	85	Coiled-coil-helix-c	19	10090.ENSMUSP00000041196
66576	P99028	Uqcrh	2	7	74.2	25	0	85.042	1275700000	437000000	369530000	Uqcrh	0.342557028	Uqcrh	Uqcrh	89	Cytochrome b-c1 c	3	10090.ENSMUSP00000077744
66695	Q99MQ4	Aspn	1	7	19.3	9	0	40.157	4815700	24289000	9243000	Aspn	5.043711195	Aspn	Aspn	373	Asporin; Binds cal	5	10090.ENSMUSP00000021820
67035	Q9D832	Dnajb4	3	4	14.5	8	0	6.2338	5796900	14324000	1562600	Dnajb4	2.470975866	Dnajb4	Dnajb4	337	DnaJ homolog sub	6	10090.ENSMUSP00000114356
67089	P62334	Psmc6	1	5	14.7	6	0	8.6838	13760000	6020000	1631900	Psmc6	0.4375	Psmc6	Psmc6	389	26S proteasome reg	2	10090.ENSMUSP00000022380
67426	Q60936;F7B1B6;Q60936-2	Adck3	5	9	16.1	17	0	28.376	128590000	63452000	11941000	Adck3	0.49344428	Coq8a	Adck3	645	Atypical kinase CC	11	10090.ENSMUSP00000027766
67671	Q9JJI8	Rpl38	1	2	32.9	3	0	6.3237	8571300	25043000	13594000	Rpl38	2.921727159	Rpl38	Rpl38	70	Ribosomal protein	2	10090.ENSMUSP00000102213
68337	Q9DCT8;A0A0G2JF37	Crip2	2	7	45.7	15	0	53.434	93064000	45464000	24810000	Crip2	0.488524026	Crip2	Crip2	208	Cysteine rich protei	2	10090.ENSMUSP00000081943
68572	A2A6T4;Q8R035-2;Q8R035	Ict1	3	2	11.8	2	0	2.4301	1985500	985720	537550	Ict1	0.49645933	Mrpl58	Ict1	206	Peptidyl-tRNA hyc	3	10090.ENSMUSP00000116746
68760	B2RQK7;Q8BWB1;D3YU08	Synpo2l	3	14	19.3	22	0	31.756	18392000	121660000	3997300	Synpo2l	6.614832536	Synpo2l	Synpo2l	975	Synaptopodin 2-lik	6	10090.ENSMUSP00000053176
68794	Q8VHX6-2;Q8VHX6	Flnc	2	58	29	139	0	225.06	409220000	1105000000	13518000	Flnc	2.700259029	Flnc	Flnc	2737	Filamin-C; Muscle	6	10090.ENSMUSP00000064163
69156	H3BJ37;Q8BIG7	Comtd1	2	2	10.5	2	0	2.4816	2856200	8874900	1854900	Comtd1	3.107240389	Comtd1	Comtd1	262	Catechol O-methylt	9	10090.ENSMUSP00000119330
69162	Q3UPL0-2;Q3UPL0;S4R2A9	Sec31a	5	5	5	6	0	8.9449	4355700	1949200	711450	Sec31a	0.447505567	Sec31a	Sec31a	1230	Protein transport pr	25	10090.ENSMUSP00000092157
69253	Q99PR8	Hspb2	2	3	20.9	3	0	10.005	11370000	44206000	8195300	Hspb2	3.887950748	Hspb2	Hspb2	182	Heat shock protein	2	10090.ENSMUSP00000042374
69288	E9PZ72;Q9DAK3;E9PZ17;E9QAZ7;E9Q922;E9PYH9;E9Q1X7;Q91V93	Rhobtb1;Rhobtb2	8	2	3.2	4	0.0028	1.6481	12144000	29387000	1987400	Rhobtb1	2.419878129	Rhobtb1;Rhobtb2	Rhobtb1	695	Rho-related BTB d	1	10090.ENSMUSP00000020101

69617	Q8K411-2;Q8K411;Q8K411-3	Pitrm1	3	4	4.1	7	0	8.4085	4208800	9622500	405090	Pitrm1	2.286281125	Pitrm1	Pitrm1	1036	Presequence proteas	9	10090.ENSMUSP00000021611
70257	P56379;A0A1Y7VLP0	Mp68	2	2	29.3	13	0	8.9306	41271000	208300000	137970000	Mp68	5.047127523	Atp5mpl	Mp68	58	6.8 kDa mitochond	3	10090.ENSMUSP00000021719
71853	Q3TML0;Q922R8	Pdia6	2	5	14.6	10	0	26.175	12398000	29536000	5006400	Pdia6	2.382319729	Pdia6	Pdia6	445	May function as a c	4	10090.ENSMUSP00000052912
72560	G3UWC2;Q9CZR2	Naalad2	2	5	7.8	7	0	10.499	1254900	4595900	729200	Naalad2	3.662363535	Naalad2	Naalad2	740	N-acetylated-alpha-l	29	10090.ENSMUSP00000128674
72590	Q8BVQ5;A0A140LI84	Ppme1	2	2	5.2	2	0.0006	2.1633	2737900	5486600	547380	Ppme1	2.003944629	Ppme1	Ppme1	386	Protein phosphatas	10	10090.ENSMUSP00000032963
73095	Q8R0Y8	Slc25a42	1	2	5.3	2	0.0012	1.8073	15482000	7186200	2142900	Slc25a42	0.464164837	Slc25a42	Slc25a42	318	Mitochondrial coen	2	10090.ENSMUSP00000105754
73333	Q3V132	Slc25a31	1	2	13.8	1	0.0006	2.1662	14924000	4412000	1140600	Slc25a31	0.295631198	Slc25a31	Slc25a31	320	ADP/ATP transloc	24	10090.ENSMUSP00000088723
73723	Q91VW3;I7HPY0	Sh3bgrl3	2	2	18.3	4	0	3.1668	9220300	25373000	28692000	Sh3bgrl3	2.751862738	Sh3bgrl3	Sh3bgrl3	93	SH3 domain-bindi	15	10090.ENSMUSP00000030651
75475	Q8K010;E9Q484	Oplah	4	4	3.7	5	0	10.237	8112000	2681000	364260	Oplah	0.330498028	Oplah	Oplah	1288	5-oxoprolinase; Ca	4	10090.ENSMUSP00000129100
76263	Q9DCM2;A0A0N4SVE5	Gstk1	2	8	44.2	22	0	47.606	121880000	58800000	17686000	Gstk1	0.482441746	Gstk1	Gstk1	226	Glutathione S-trans	3	10090.ENSMUSP00000031897
76491	A0A087WP24;A0A087WRJ2;A0A087WPF8;A0A087WSR2;E9QN99;Q8VCR7	Abhd14b	6	4	29.6	5	0	3.7559	5949200	13352000	6625400	Abhd14b	2.244335373	Abhd14b	Abhd14b	210	Has hydrolase activ	18	10090.ENSMUSP00000038755
77579	Q5SV64;Q3UH59;Q61879	Myh10	4	17	18.4	22	0	53.615	7957400	28724000	1309100	Myh10	3.609721768	Myh10	Myh10	1976	Myosin-10; Involv	1	10090.ENSMUSP00000099671
78388	E9Q3X0;Q9EQK5	Mvp	3	20	24.9	31	0	33.158	38921000	89859000	5044700	Mvp	2.308753629	Mvp	Mvp	870	Major vault protein	7	10090.ENSMUSP00000127250
78655	Q3UGC7;Q66JS6	Eif3j1;Eif3j2	2	3	13	5	0	6.4449	4879500	10372000	2002900	Eif3j1	2.125627626	Eif3j1;Eif3j2	Eif3j1	261	Eukaryotic translati	2	10090.ENSMUSP00000028668
83493	A0A5F8MPK9;Q9EP69	Sacm1l	2	6	9.4	7	0	7.1104	10507000	25329000	1518700	Sacm1l	2.410678595	Sacm1l	Sacm1l	587	Phosphatidylinositi	14	10090.ENSMUSP00000026270
83997	F6UV57;F6YCM8;F8WIH0;D3Z7V3;H7BX64;Q3URD3-4;Q3URD3-3;Q3URD3-5;Q3URD3-2;Q3URD3	Slmap	10	11	23.8	18	0	54.758	49229000	145610000	8879200	Slmap	2.957809421	Slmap	Slmap	845	Sarcolemmal meml	1	10090.ENSMUSP00000117816
93677	Q3UHZ5	Lmod2	1	5	8.7	7	0	7.8654	9777400	20338000	1415400	Lmod2	2.080103095	Lmod2	Lmod2	550	Leiomodin-2; Medi	6	10090.ENSMUSP00000031694
93692	A0A1Y7VM65;Q9QUH0	Glrx	2	3	23.8	5	0	5.4459	13902000	6905400	5362900	Glrx	0.496719896	Glrx	Glrx	107	Glutaredoxin-1; Ha	2	10090.ENSMUSP00000022082
94280	Q3U4F0;Q91V61-2;Q91V61;A0A494BB84	Sfxn3	4	2	10	3	0	8.4182	1210000	3720800	411380	Sfxn3	3.075041322	Sfxn3	Sfxn3	321	Sideroflexin-3; Pot	22	10090.ENSMUSP00000059419
