# Supplementary material for: Comparative Proteomic and Phosphoproteomic Analyses Reveal Molecular Signatures of Myocardial Infarction and Transverse Aortic Constriction in Aged Mouse Models
Source: Cardiol Res Pract. 2024 Oct 28;2024:9395213. doi: 10.1155/2024/9395213 (PMC11535427; doi:10.1155/2024/9395213)
Supplement: Supporting Information — Table S4: List of coexpressed proteins in MI and TAC. [file 9395213.f4.pdf]

MI(Num:378)	TAC(Num:138)	MI|TAC(Num:112)
Chchd7	Tollip	Chchd7
9030617O03Rik	Chchd7	9030617O03Rik
Pdk4	Fech	Slc25a31
Slc25a31	9030617O03Rik	Fech
Fech	Slc25a31	Mpst
Mpst	Ces1d	Uqcrh
Uqcrh	Oplah	Ces1d
Ces1d	Rtn4ip1	Ppme1
Ppme1	Acaa2	Slc25a42
Acox1	Uqcrh	Acaa2
Nudt7	Bckdhb	Atp5j2
Slc25a42	Qil1	Tollip
Mtatp6	Mpst	Coq10a
Acaa2	Abcc9	Ech1
Cdh2	Psmc6	Bckdhb
Atp5j2	Xdh	Eci1
Tollip	Sec31a	Etfb
Coq10a	Atp5j2	Gstk1
Ube2l3	Maob	Qil1
Ech1	Eci1	A0A338P6I9
Bckdhb	A0A338P6I9	P0DN34
Eci1	Slc25a42	Slc27a1
Aldh5a1	Uqcr10	Abcc9
Acot2	Etfb	Uqcr10
Atad1	Fitm2	Oplah
Etfb	Ech1	Rtn4ip1
Gstk1	Coq10a	Slmap
Qil1	Gstk1	Sfxn3
Rtn2	P0DN34	Sacm1l
Dbt	Crip2	Dnajb6
Lyrm5	Adck3	Aldh9a1
Suclg1	Slc27a1	Rhobtb1
Gal3st4	Ict1	Pfkp
A0A338P6I9	Glrx	Lmcd1
Akr1b10	Synpo	Psmd14
Prdx3	Ppme1	Dync1i2
Macrod1	P4hb	Msn
Idh3g	Agrn	Nrap
Nt5c3a	Cdc37	Eif3j1
Acsf3	Hnrnpc	Acta1
Cars2	Hsp90aa1	Hnrnpl
P0DN34	Psmd14	Xirp1
Slc16a1	Clip1	Fhl1
Slc27a1	Msn	Pdlim1
Abcc9	Hnrnpl	Mvp
Abcb10	Lmod2	Fscn1
Atp5h	Actn4	Synpo2l
Slc25a22	Uchl1	Tfrc
Lpl	Anxa1	Ganab
Hadhb	Eif3j1	Picalm
Idh2	Pdia4	Myl1
Mmaa	Sorbs2	Pdia6
Ndufa11	Dnajb6	Anxa4

Decr1	Tagln2	P4hb
Got1	Anxa3	Khsrp
Vdac1	Fbln5	Efhd2
Art3	Dpysl3	Tagln
Atp5c1	Eif3b	Ace
Ndufa5	Abhd14b	Uchl1
Eci2	Ahnak2	Bcap31
Sdha	Lmcd1	Actn4
Ogdhl	Myl6	Cdv3
Suclg2	Itih4	Anxa3
Dld	Picalm	Pdia4
Cox7a1	Pitrm1	Snd1
Coa6	Tagln	Clu
Acads	Mvp	Glg1
Bckdha	Cst3	Tagln2
Aldh6a1	Clu	Eif3b
Ndufa12	Dync1i2	Higd1a
Srl	Hspb1	S100a6
Coq3	Pdia6	Abhd14b
L2hgdh	Hspb7	Nap1l4
Mterf2	Sept2	Mp68
Cpt2	Sacm1l	Hnrnpc
Uqcr10	Col1a1	Sec31a
Crat	Rhobtb1	Myl6
Oplah	Clic5	Ckap4
Hadha	Dnajb4	Xirp2
Etfdh	S100a11	Clic1
Txnrd2	Vps29	Pls3
Dlst	Vim	Sept2
Sdr39u1	Nap1l4	Cst3
Ak1	Ogn	Coro1c
Mccc1	Lcp1	Nucb1
Dbi	Ganab	Hist1h1b
Usmg5	S100a6	S100a11
Mtfp1	Flnc	Vim
Mccc2	Anxa4	Calu
Acadvl	Pdlim1	Lcp1
Ndufb7	Sh3bgrl3	Anxa1
Iba57	Khsrp	Dpysl3
Ckm	Fscn1	Sh3bgrl3
Acadm	Lum	Rpl3
Cyc1	Cygb	Flna
Rtn4ip1	Pls3	Vcan
Ndufb11	Hist1h1b	Myh7
Ciapin1	Pfkp	Cald1
Rac1	Ckap4	Myh10
Ywhaz	Rpl38	Ahnak2
Capns1	Coro1c	Cygb
Slmap	Myl1	Naalad2
Sfxn3	Slmap	Fbln5
Sacm1l	Rbp1	Lum
Emc2	Sfxn3	Ogn
Fga	Nucb1	Bgn
Dnajb6	Comtd1	Col1a1

Aldh9a1
Ywhab
Hnrnpa1
Snx2
Gnb1
Cltb
Map4
Serbp1
Rhobtb1
Dad1
Fabp5
Farsb
Dync1li1
Fam49b
Eif3i
Tmed9
Sars
Pepd
Rps29
Pfkp
Vwa5a
Hist1h1e
Hprt1
Ruvbl2
Rps16
Lasp1
Ap2b1
H3f3a
H3f3a
Lmcd1
Mapre1
Ssb
Srsf2
Thy1
Ppp2r4
Hnrnpk
Psmd14
Cnpy2
Gpx1
Gng12
Rab10
Akr1a1
Cbx3
Strap
Pa2g4
Ywhah
Ncl
Sntb2
Srsf7
Palld
Sfpq
Copb2
Lancl1
Cfl1

Glg1
Bcap31
Bgn
Snd1
Clic1
Ace
Myh10
Rcn3
Efhd2
Naalad2
Hspb2
Cald1
Rpl3
Calu
Flna
Vcan
Xirp1
Cdv3
Aspn
Mp68
Tfrc
Efemp1
Higd1a
Fhl1
Synpo2l
Nrap
Aldh9a1
Acta1
Postn
Myh7
Xirp2

Rbp1
Aspn
Efemp1
Rcn3
Postn

Dync1i2
Actr2
Ddx5
Rps28
Set
Cnbp
Calr
S100a13
Cyb5r3
Arhgdia
Rap1a
Cstb
Msn
Hnrnph1
Cltc
Col6a1
Hyou1
Myh11
Erp29
Ap2m1
Nrap
Ckb
Tln1
Col6a2
H2afv
Snrpb
Rpl5
Dstn
Arcn1
Eif3j1
Dpep1
Lmnb1
Asph
Rbmxl1
Cd47
Psap
Copa
Lgals1
Acta1
Cndp2
P58774-2
Hnrnpl
Xirp1
Ighg1
Lman2
Col15a1
Tpp1
Ap2a1
Uggt1
Fhl1
Flnb
H2afj
Pdlim1
Serpind1

Gm10320
Rpl26
Slc44a2
Mvp
Hnrnpa3
Ghitm
Anxa5
Fscn1
Myl12a
Ppia
Synpo2l
Rpl34
Tfrc
Arpc4
Ide
Ganab
Twf1
Gmfb
Dpysl2
Col6a3
Sri
Hsp90b1
Mtpn
Tpm4
Picalm
B2m
Itih1
Rpn1
Arpc2
Vars
Myl1
Hnrnpm
Ppib
Lrrc59
Hdgf
Eef1a1
Cyfip1
Tmpo
Ahnak
Ywhaq
Tardbp
Ddost
Pdia6
Anxa4
P20065-2
H2-D1
P4hb
Rpn2
Sec61a1
Gpx3
Anxa2
Khsrp
Pabpc1
Actr3

Actn1
Csrp1
Ptms
Pdia3
Lmna
Efhd2
Apoe
Tagln
Ace
Gsn
Arpc1b
Snx5
Uchl1
Bcap31
Myh9
Actn4
Hnrnpd
Hmgb1
Cap1
Ptma
Coro1b
Ephx1
Cdv3
Serpinh1
Cd34
Anxa3
Capza1
Ltbp4
Rrbp1
Ybx1
Pdia4
Ddah2
Ptbp1
Snd1
Crip1
Clu
Actb
Sept7
Arf4
Pnp
Fmo2
Glg1
H2afy
Tagln2
Sept11
Eif3b
Arpc5
Arpc3
Higd1a
S100a6
Abhd14b
Nap1l4
Mp68
Hnrnpc

Sec31a
Fbn1
Myl6
Vat1
Tkt
Ckap4
Xirp2
A0A5H1ZRK8
Clic1
Pls3
Sept2
Ctsz
Cpq
Tmem43
Cst3
Coro1c
Aldh1a1
Vtn
Nucb1
Hnrnpul2
Hist1h1b
Fn1
Dcn
S100a11
Vim
Calu
Lcp1
Anxa1
Dpysl3
Sh3bgrl3
Rpl3
Ptgis
Flna
Vcan
Myh7
Entpd2
Arhgdib
Cald1
Iqgap1
Cotl1
Myh10
Ahnak2
Cygb
Naalad2
Col1a2
Fbln5
Lum
Ogn
Bgn
Col1a1
Rbp1
Aspn
Efemp1
Rcn3

Postn
