# Supplementary material for: Comparative Proteomic and Phosphoproteomic Analyses Reveal Molecular Signatures of Myocardial Infarction and Transverse Aortic Constriction in Aged Mouse Models
Source: Cardiol Res Pract. 2024 Oct 28;2024:9395213. doi: 10.1155/2024/9395213 (PMC11535427; doi:10.1155/2024/9395213)
Supplement: Supporting Information — Table S5: List of the identified phosphoproteins in global phosphoproteomic analysis. [file 9395213.f5.pdf]

Track_id	Intensity MI	Intensity sham	Intensity TAC	Intensity MI	Intensity sham	Intensity TAC	Protein group IDs	Leading proteins	Position	Protein	Amino acid	Number of Phospho (STY)	Score diff	PEP	Score	Gene names
A0A087WQA0_S_177_1	21810000	0	25617000	0.276816588	0	0.119598352	14	A0A087WQA0	177	A0A087WQA0	S	1	144.803	0.000612875	144.8	Bclaf1
A0A087WQA0_S_494_1	0	0	0	0	0	0	14	A0A087WQA0	494	A0A087WQA0	S	1	67.704	0.0210858	67.704	Bclaf1
A0A087WQA0_S_510_1	0	0	35763000	0	0	0.166967087	14	A0A087WQA0	510	A0A087WQA0	S	1	64.7759	0.00869284	90.754	Bclaf1
A0A087WQA0_S_656_1	0	0	15201000	0	0	0.070969066	14	A0A087WQA0	656	A0A087WQA0	S	1	37.0441	0.0113247	115.7	Bclaf1
A0A087WQQ3_S_2_3	0	336220000	231990000	0	5.525804332	1.083094106	16	A0A087WQQ3	2	A0A087WQQ3	S	3	53.6502	0.0310392	61.161	Rnf2
A0A087WQQ3_T_10_3	0	336220000	231990000	0	5.525804332	1.083094106	16	A0A087WQQ3	10	A0A087WQQ3	T	3	53.8427	0.0310392	61.161	Rnf2
A0A087WQQ3_T_7_3	0	336220000	231990000	0	5.525804332	1.083094106	16	A0A087WQQ3	7	A0A087WQQ3	T	3	54.2112	0.0310392	61.161	Rnf2
A0A087WRQ4_S_2_1	0	0	41110000	0	0	0.191930681	19	A0A087WRQ4	2	A0A087WRQ4	S	1	73.8964	0.00012603	128.99	Sumo1
A0A087WRU0_S_1164_1	12901000	0	30714000	0.163741898	0	0.143394769	13	A0A087WRU0	1164	A0A087WRU0	S	1	15.366	0.000579889	142.38	Tns1
A0A087WRU0_T_1161_1	0	0	0	0	0	0	13	A0A087WRU0	1161	A0A087WRU0	T	1	13.2261	0.00605388	95.428	Tns1
A0A087WRY3_S_58_2	17431000	0	0	0.221237503	0	0	20	A0A087WRY3	58	A0A087WRY3	S	2	98.5062	1.25E-24	206.28	Nucks1
A0A087WRY3_S_61_2	0	0	0	0	0	0	20	A0A087WRY3	61	A0A087WRY3	S	2	109.218	1.25E-24	206.28	Nucks1
A0A0A0MQC7_S_494_1	0	0	37424000	0	0	0.174721815	27	A0A0A0MQC7	494	A0A0A0MQC7	S	1	20.2793	0.000232247	150.05	Mapt
A0A0A6YVP7_S_34_1	0	0	0	0	0	0	35	A0A0A6YVP7	34	A0A0A6YVP7	S	1	31.2524	0.00260926	92.051	Mlf1
A0A0G2JEX1_S_352_1	0	0	25665000	0	0	0.11982245	61;65	A0A0G2JEX1;A0A0G2JFD8	352	A0A0G2JEX1	S	1	54.4733	0.0482422	101.2	Nexn
A0A0G2JFH2_S_594_1	8463200	0	11187000	0.107416513	0	0.052228862	66	A0A0G2JFH2	594	A0A0G2JFH2	S	1	90.449	0.012489	105.13	
A0A0J9YUS5_S_914_1	0	0	0	0	0	0	82	A0A0J9YUS5	914	A0A0J9YUS5	S	1	21.2897	0.00938292	69.261	Eif4g1
A0A0J9YVE0_S_160_2	0	0	46020000	0	0	0.214854049	75	A0A0J9YVE0	160	A0A0J9YVE0	S	2	67.1694	0.0551194	67.169	Rbpj
A0A0J9YVE0_S_163_2	0	0	46020000	0	0	0.214854049	75	A0A0J9YVE0	163	A0A0J9YVE0	S	2	67.1694	0.0551194	67.169	Rbpj
A0A0N4SV32_S_284_1	0	0	34049000	0	0	0.158964918	91	A0A0N4SV32	284	A0A0N4SV32	S	1	12.2169	0.00257907	78.354	Serbp1
A0A0R4J0S1_S_207_1	0	0	34297000	0	0	0.160122758	124	A0A0R4J0S1	207	A0A0R4J0S1	S	1	17.0171	0.0517681	91.961	Cdc42ep1
A0A0R4J146_S_224_2	0	229690000	0	0	3.77497471	0	129	A0A0R4J146	224	A0A0R4J146	S	2	35.0057	0.0465515	53.751	Nt5c3b
A0A0R4J146_Y_225_2	0	229690000	0	0	3.77497471	0	129	A0A0R4J146	225	A0A0R4J146	Y	2	34.5082	0.0465515	53.751	Nt5c3b
A0A0U1RNJ1_T_987_2	27444000	0	0	0.348324367	0	0	148	A0A0U1RNJ1	987	A0A0U1RNJ1	T	2	47.559	0.0434629	47.559	Fasn
A0A0U1RNJ1_Y_992_2	27444000	0	0	0.348324367	0	0	148	A0A0U1RNJ1	992	A0A0U1RNJ1	Y	2	47.559	0.0434629	47.559	Fasn
A0A1B0GS20_S_619_2	380060000	0	198650000	4.823792414	0	0.927439304	183	A0A1B0GS20	619	A0A1B0GS20	S	2	44.6394	0.0435198	58.699	

A0A1B0GS20_T_612_2	380060000	0	198650000	4.823792414	0	0.927439304	183	A0A1B0GS20	612	A0A1B0GS20	T	2	44.6394	0.0435198	58.699	
A0A1B0GSC6_S_143_1	44151000	17289000	107050000	0.560372728	0.284146187	0.499785439	188	A0A1B0GSC6	143	A0A1B0GSC6	S	1	87.94	0.0127801	93.258	
A0A1B0GSZ4_S_9_2	0	0	27411000	0	0	0.127974018	192	A0A1B0GSZ4	9	A0A1B0GSZ4	S	2	57.8026	0.0481458	65.347	
A0A1B0GSZ4_Y_6_2	0	0	27411000	0	0	0.127974018	192	A0A1B0GSZ4	6	A0A1B0GSZ4	Y	2	58.7662	0.0481458	65.347	
A0A1B0GT92_S_589_1	19618000	0	47068000	0.248995315	0	0.219746857	194	A0A1B0GT92	589	A0A1B0GT92	S	1	26.3833	0.000740686	138.21	Gys1
A0A1L1SS78_T_168_1	0	10718000	0	0	0.176151243	0	216	A0A1L1SS78	168	A0A1L1SS78	T	1	30.6953	0.0149931	92.692	Kif15
A0A1L1SUS7_S_113_1	0	0	0	0	0	0	226	A0A1L1SUS7	113	A0A1L1SUS7	S	1	69.2612	0.00938292	69.261	Cdk17;Cdk16
A0A1W2P6K9_S_101_1	9256300	4918700	38483000	0.117482686	0.080839253	0.179665979	229	A0A1W2P6K9	101	A0A1W2P6K9	S	1	20.6522	0.0441692	94.454	Sgpp1
A0A1W2P737_S_349_2	39544000	23462000	0	0.501899824	0.385599968	0	234	A0A1W2P737	349	A0A1W2P737	S	2	38.0897	0.0211045	67.563	Pcnt
A0A1W2P737_T_352_2	39544000	23462000	0	0.501899824	0.385599968	0	234	A0A1W2P737	352	A0A1W2P737	T	2	30.3715	0.0211045	67.563	Pcnt
A0A1Y7VNX8_T_199_1	28515000	0	93738000	0.361917699	0	0.437635568	249	A0A1Y7VNX8	199	A0A1Y7VNX8	T	1	9.22276	0.056821	40.179	Ptpn21
A0A1Y7VP73_Y_243_1	29977000	14716000	24126000	0.380473676	0.241858713	0.112637305	256	A0A1Y7VP73	243	A0A1Y7VP73	Y	1	59.2052	0.0569296	59.205	
A0A286YD39_T_84_1	0	0	29534000	0	0	0.13788569	262	A0A286YD39	84	A0A286YD39	T	1	105.195	0.0422218	105.2	Rmdn2
A0A338P6D9_S_126_1	0	0	38252000	0	0	0.178587507	291	A0A338P6D9	126	A0A338P6D9	S	1	101.712	0.0145848	101.71	Eif2b5
A0A338P6F2_S_8_1	0	18073000	702560000	0	0.297031294	3.28004912	292	A0A338P6F2	8	A0A338P6F2	S	1	35.7033	0.0432971	66.267	Osbpl11
A0A338P703_S_59_1	0	0	97665000	0	0	0.455969593	297	A0A338P703	59	A0A338P703	S	1	24.3456	2.71E-08	163.45	Ahsg
A0A3B2W8B1_T_92_2	48761000	8575500	72958000	0.618883708	0.140939073	0.340619767	300	A0A3B2W8B1	92	A0A3B2W8B1	T	2	45.598	0.0454788	45.598	Vmn2r3;Vmn2r2;Vmn2r5;Vmn2r1;Vmn2r4;Vmn2r6;Vmn2r7
A0A3B2W8B1_T_96_2	48761000	8575500	72958000	0.618883708	0.140939073	0.340619767	300	A0A3B2W8B1	96	A0A3B2W8B1	T	2	45.598	0.0454788	45.598	Vmn2r3;Vmn2r2;Vmn2r5;Vmn2r1;Vmn2r4;Vmn2r6;Vmn2r7
A0A494B9K4_S_675_1	15526000	0	25533000	0.197058888	0	0.11920618	316	A0A494B9K4	675	A0A494B9K4	S	1	8.82934	4.32E-11	195.6	Add3
A0A571BDS0_S_295_1	0	0	272120000	0	0	1.270449451	340	A0A571BDS0	295	A0A571BDS0	S	1	150.493	0.00000101	172.82	Xirp1
A0A5F8MPJ2_S_516_1	0	1774900	67514000	0	0.029170633	0.315203308	258;347	A0A286YCI8;A0A5F8MPJ2	516	A0A5F8MPJ2	S	1	110.662	0.00000472	167.18	Sorbs1
A0A5F8MPJ2_S_62_1	0	0	23793000	0	0	0.111082625	258;347	A0A286YCI8;A0A5F8MPJ2	62	A0A5F8MPJ2	S	1	11.7864	0.0285979	93.381	Sorbs1
A0A5F8MPR4_S_1441_1	0	0	53593000	0	0	0.250210192	349	A0A5F8MPR4	1441	A0A5F8MPR4	S	1	18.7224	0.0118308	114.89	Xirp2
A0A5F8MPR4_S_2297_2	0	0	0	0	0	0	349	A0A5F8MPR4	2297	A0A5F8MPR4	S	2	44.6282	0.00476147	73.927	Xirp2
A0A5F8MPR4_S_2421_1	0	0	34177000	0	0	0.159562512	349	A0A5F8MPR4	2421	A0A5F8MPR4	S	1	57.4225	0.0224014	130.66	Xirp2

A0A5F8MPR4_S_796_1	0	0	62409000	0	0	0.291369542	349	A0A5F8MPR4	796	A0A5F8MPR4	S	1	99.9731	0.0569719	99.973	Xirp2
A2AJW4_S_58_1	0	0	0	0	0	0	384	A2AJW4	58	A2AJW4	S	1	17.3687	0.0048731	79.148	Ppp1r3d
A2AMM0_T_334_1	0	0	98394000	0	0	0.459373083	390	A2AMM0	334	A2AMM0	T	1	102.443	0.00174344	131.25	Murc
A2APF7_T_44_2	0	9643200	0	0	0.158486813	0	393	A2APF7	44	A2APF7	T	2	51.2109	0.0355989	51.211	Zbp1
A2APF7_Y_50_2	0	9643200	0	0	0.158486813	0	393	A2APF7	50	A2APF7	Y	2	51.2109	0.0355989	51.211	Zbp1
A2ASS6-3_S_3432_1	0	0	57442000	0	0	0.268180058	401	A2ASS6-3	3432	A2ASS6-3	S	1	8.14774	0.017874	64.203	Ttn
A2ASS6_S_12871_1	0	0	50411000	0	0	0.235354356	353;400	A0A5K1VVQ9;A2ASS6	12871	A2ASS6	S	1	28.2161	0.0271728	136.93	Ttn
A2ASS6_S_13764_1;2	7806800000	5405300000	13738000000	99.08536182	88.83656582	64.13874232	353;400	A0A5K1VVQ9;A2ASS6	13764	A2ASS6	S	1;2	51.4453	0.0225269	59.426	Ttn
A2ASS6_S_1424_1	7782800	0	48457000	0.098780749	0	0.226231696	353;400;401	A0A5K1VVQ9;A2ASS6;A2ASS6-3	1424	A2ASS6	S	1	53.6898	0.00806126	77.062	Ttn
A2ASS6_S_2080_1	154170000	118040000	1326700000	1.956754398	1.939997452	6.193977976	353;400;401	A0A5K1VVQ9;A2ASS6;A2ASS6-3	2080	A2ASS6	S	1	42.9742	0.00793451	124.6	Ttn
A2ASS6_S_264_1;2	96574000	19116000	502910000	1.225735222	0.314173088	2.347941105	353;400;401	A0A5K1VVQ9;A2ASS6;A2ASS6-3	264	A2ASS6	S	1;2	14.5299	0.00000229	175.01	Ttn
A2ASS6_S_322_1	51393000	43978000	565210000	0.652289542	0.722782175	2.638801758	353;400;401	A0A5K1VVQ9;A2ASS6;A2ASS6-3	322	A2ASS6	S	1	42.5908	0.0154305	101.38	Ttn
A2ASS6_S_33861_1;2	0	0	51110000	0	0	0.238617784	353;400	A0A5K1VVQ9;A2ASS6	33861	A2ASS6	S	1;2	36.8116	0.000000604	176.23	Ttn
A2ASS6_S_33933_1	1040100000	725980000	2550700000	13.20114321	11.93154312	11.9084794	353;400	A0A5K1VVQ9;A2ASS6	33933	A2ASS6	S	1	31.7977	0.0587908	86.882	Ttn
A2ASS6_S_34097_1;2	0	0	325730000	0	0	1.520739011	353;400	A0A5K1VVQ9;A2ASS6	34097	A2ASS6	S	1;2	62.7061	8.72E-08	152.99	Ttn
A2ASS6_S_34107_1;2	0	6489900	22762000	0	0.106662059	0.106269184	353;400	A0A5K1VVQ9;A2ASS6	34107	A2ASS6	S	1;2	50.0452	0.0251378	66.056	Ttn
A2ASS6_S_34109_1;2	12639000	6489900	56407000	0.160416546	0.106662059	0.263347943	353;400	A0A5K1VVQ9;A2ASS6	34109	A2ASS6	S	1;2	42.7422	0.0251378	93.096	Ttn
A2ASS6_S_34464_2	183630000	202150000	701080000	2.330666213	3.322352465	3.273139428	353;400	A0A5K1VVQ9;A2ASS6	34464	A2ASS6	S	2	40.8437	0.0241939	96.135	Ttn
A2ASS6_S_34470_1;2	202660000	223190000	759700000	2.572198523	3.668146657	3.546819227	353;400	A0A5K1VVQ9;A2ASS6	34470	A2ASS6	S	1;2	41.3633	0.00495395	96.135	Ttn
A2ASS6_S_34476_1	221190000	196850000	559030000	2.807384739	3.235246514	2.609949128	353;400	A0A5K1VVQ9;A2ASS6	34476	A2ASS6	S	1	62.5843	0.000978557	143.98	Ttn
A2ASS6_S_34488_1	1176300000	755430000	2492100000	14.92981902	12.41555638	11.63489298	353;400	A0A5K1VVQ9;A2ASS6	34488	A2ASS6	S	1	34.4882	0.000488172	150.69	Ttn
A2ASS6_S_814_1	0	0	764980000	0	0	3.571470017	353;400;401	A0A5K1VVQ9;A2ASS6;A2ASS6-3	814	A2ASS6	S	1	31.2177	0.0048115	119.27	Ttn
A2ASS6_T_33859_2	0	0	16126000	0	0	0.075287623	353;400	A0A5K1VVQ9;A2ASS6	33859	A2ASS6	T	2	43.5909	0.0186247	69.737	Ttn
A2ASS6_T_34099_1;2	0	0	378520000	0	0	1.767200229	353;400	A0A5K1VVQ9;A2ASS6	34099	A2ASS6	T	1;2	63.0136	0.000792385	82.65	Ttn
A2AUL9_S_353_1	32263000	12398000	50936000	0.409488014	0.203762186	0.237805429	410	A2AUL9	353	A2AUL9	S	1	40.8364	0.0588548	44.457	Eif2ak4
A2AWA9_S_920_1	8696400	0	26165000	0.110376331	0	0.122156805	413	A2AWA9	920	A2AWA9	S	1	98.0483	0.0286457	98.048	Rabgap1

A2BGI8_T_140_1	0	0	0	0	0	0	419	A2BGI8	140	A2BGI8	T	1	52.2783	0.0550653	52.278	Ppih
A2CG76_S_275_2	0	23399000	43772000	0	0.384564558	0.204358788	420	A2CG76	275	A2CG76	S	2	49.0755	0.0208363	61.344	Ehmt2
A2CG76_T_274_2	0	23399000	43772000	0	0.384564558	0.204358788	420	A2CG76	274	A2CG76	T	2	48.3358	0.0208363	61.344	Ehmt2
B1AR69_S_1303_1	9683200	6873400	0	0.122900981	0.112964914	0	436	B1AR69	1303	B1AR69	S	1	23.1581	0.0198656	58.596	Myh13
B1AUE5_T_201_1	0	0	38453000	0	0	0.179525918	443	B1AUE5	201	B1AUE5	T	1	8.70671	0.00399169	111.95	Pex10
B2RQK7_S_792_1	0	0	22347000	0	0	0.104331669	453	B2RQK7	792	B2RQK7	S	1	10.9764	0.0135787	98.942	Synpo2l
B2RQK7_T_705_1	0	0	32317000	0	0	0.150878711	453	B2RQK7	705	B2RQK7	T	1	56.7293	0.0335629	56.729	Synpo2l
B2RQK7_T_721_1	0	0	43752000	0	0	0.204265414	453	B2RQK7	721	B2RQK7	T	1	85.613	0.0198724	95.401	Synpo2l
B7ZNU9_S_88_1	45955000	0	22960000	0.583269432	0	0.107193589	467	B7ZNU9	88	B7ZNU9	S	1	5.95633	0.00049134	147.33	Sntb2
B7ZNU9_S_90_1	15147000	0	0	0.192248549	0	0	467	B7ZNU9	90	B7ZNU9	S	1	14.4749	0.00241102	127.71	Sntb2
B8A5X0_S_358_1	86212000	0	160460000	1.094218785	0	0.749141257	468	B8A5X0	358	B8A5X0	S	1	43.2974	0.0516202	43.297	Ttc25
B9EJX2_S_1336_3	14500000	0	57268000	0.184036705	0	0.267367702	444	B9EJX2	1336	B9EJX2	S	3	17.8922	0.050821	40.113	Nhs
B9EJX2_T_1331_3	14500000	0	57268000	0.184036705	0	0.267367702	444	B9EJX2	1331	B9EJX2	T	3	19.4499	0.050821	40.113	Nhs
D3YTR7_S_199_1	30382000	6688600	28352000	0.385614011	0.109927711	0.132367275	260	D3YTR7	199	D3YTR7	S	1	16.187	0.0141245	102.46	Cap2
D3YUQ9_S_162_1	0	0	191050000	0	0	0.891957106	134	D3YUQ9	162	D3YUQ9	S	1	142.719	1.55E-27	205.09	Eef1d
D3YUX5_T_166_2	0	24655000	21961000	0	0.405207025	0.102529547	493	D3YUX5	166	D3YUX5	T	2	33.5462	0.0360127	54.416	Smarcal1
D3YUX5_Y_162_2	0	24655000	21961000	0	0.405207025	0.102529547	493	D3YUX5	162	D3YUX5	Y	2	33.5462	0.0360127	54.416	Smarcal1
D3YVV9_S_895_1	25884000	7100500	163300000	0.328524556	0.116697322	0.762400395	497	D3YVV9	895	D3YVV9	S	1	55.9479	0.000000564	176.08	Synpo2
D3Z0E9_S_28_1	38492000	0	67844000	0.488547644	0	0.316743983	521	D3Z0E9	28	D3Z0E9	S	1	5.16574	0.00524538	115.7	Sorbs2
D3Z313_S_95_1	127890000	23811000	30054000	1.623203736	0.391335813	0.14031342	530	D3Z313	95	D3Z313	S	1	31.1731	0.00977603	109.72	Cbx3
D3Z3M7_S_347_1	0	0	25382000	0	0	0.118501205	529	D3Z3M7	347	D3Z3M7	S	1	10.4845	0.00000941	163.9	Clip1;Clip2
D3Z589_S_72_1	0	0	36141000	0	0	0.16873186	343	D3Z589	72	D3Z589	S	1	21.6605	0.000980224	130.29	Limch1
D3Z7U4_S_97_1	0	0	23616000	0	0	0.110256263	553	D3Z7U4	97	D3Z7U4	S	1	16.5829	0.000475753	120.32	Mecp2
D6RCX5_S_100_1	0	0	0	0	0	0	499	D6RCX5	100	D6RCX5	S	1	53.3271	0.0555331	53.327	Mtfr1l
D6RIM8_S_432_2	0	15000000	29943000	0	0.246526277	0.139795193	566	D6RIM8	432	D6RIM8	S	2	46.1582	0.0578862	46.158	Ush1c
D6RIM8_Y_430_2	0	15000000	29943000	0	0.246526277	0.139795193	566	D6RIM8	430	D6RIM8	Y	2	46.1582	0.0578862	46.158	Ush1c
E0CXA0_S_133_1	55059000	13044000	0	0.698819098	0.214379251	0	569	E0CXA0	133	E0CXA0	S	1	96.6656	0.000551476	124.3	Hdgf
E0CYV9_S_1338_1	21379000	5414800	80216000	0.271346256	0.088992699	0.374505267	577	E0CYV9	1338	E0CYV9	S	1	45.3656	0.0168153	106.58	1110002E22Rik

E0CZ80_Y_116_2	0	160330000	300630000	0	2.635037204	1.403554382	425	E0CZ80	116	E0CZ80	Y	2	20.7546	0.0511114	43.246	Ttll11
E9PV63_S_10_3	170210000	198210000	573810000	2.16033707	3.25759823	2.678952666	591	E9PV63	10	E9PV63	S	3	41.2419	0.0393165	51.286	Gstm5
E9PV63_T_8_3	170210000	198210000	573810000	2.16033707	3.25759823	2.678952666	591	E9PV63	8	E9PV63	T	3	41.2419	0.0393165	51.286	Gstm5
E9PV63_Y_16_3	170210000	198210000	573810000	2.16033707	3.25759823	2.678952666	591	E9PV63	16	E9PV63	Y	3	41.2419	0.0393165	51.286	Gstm5
E9PYB0_S_286_1	0	0	19448000	0	0	0.090797078	607	E9PYB0	286	E9PYB0	S	1	32.9893	0.0108389	118.37	Ahnak2
E9PZF4_S_275_1	6318000	0	43343000	0.080189235	0	0.202355911	619	E9PZF4	275	E9PZF4	S	1	67.1389	0.0087969	81.703	Nrap
E9Q043_S_387_1	5929000	0	0	0.075251974	0	0	621	E9Q043	387	E9Q043	S	1	9.97233	0.0224606	96.745	Fndc1
E9Q197_S_265_1	17339000	0	88124000	0.220069822	0	0.411425428	625	E9Q197	265	E9Q197	S	1	6.33836	0.0547039	60.598	Glod4
E9Q1Q4_S_201_2	99864000	39516000	8492500	1.267492516	0.649448825	0.039649022	622	E9Q1Q4	201	E9Q1Q4	S	2	28.4192	0.0529053	49.418	Tro
E9Q1Q4_Y_195_2	99864000	39516000	8492500	1.267492516	0.649448825	0.039649022	622	E9Q1Q4	195	E9Q1Q4	Y	2	27.6726	0.0529053	49.418	Tro
E9Q3E2_S_535_1	0	0	46963000	0	0	0.219256643	635	E9Q3E2	535	E9Q3E2	S	1	93.649	0.00543428	93.649	Synpo
E9Q3E2_S_765_1	0	0	0	0	0	0	635	E9Q3E2	765	E9Q3E2	S	1	61.9993	0.000128747	128.27	Synpo
E9Q3E2_S_833_1	0	12655000	114440000	0	0.207986003	0.534287209	635	E9Q3E2	833	E9Q3E2	S	1	72.0608	0.00247771	122.14	Synpo
E9Q3V6_S_178_1	0	0	72890000	0	0	0.340302295	637	E9Q3V6	178	E9Q3V6	S	1	49.6023	0.000140156	139.11	Sept2
E9Q405_T_992_2	10066000	0	28829000	0.12775955	0	0.13459425	198	E9Q405	992	E9Q405	T	2	41.4482	0.0544485	41.448	Myo18a
E9Q405_Y_994_2	10066000	0	28829000	0.12775955	0	0.13459425	198	E9Q405	994	E9Q405	Y	2	41.4482	0.0544485	41.448	Myo18a
E9Q447_S_2335_1	3667000000	0	6255800000	46.54224801	0	29.20651799	424;965	E9Q447;P16546-2	2335	E9Q447	S	1	44.3175	0.0521429	44.318	Sptan1
E9Q4Z2_S_136_1	0	0	0	0	0	0	643	E9Q4Z2	136	E9Q4Z2	S	1	8.34522	0.0477895	58.699	Acacb
E9Q5C9-2_S_563_1	0	0	43272000	0	0	0.202024433	263	E9Q5C9-2	563	E9Q5C9-2	S	1	163.696	5.33E-41	267.56	Nolc1
E9Q616_S_217_1	0	6696400	49371000	0	0.110055904	0.230498897	649	E9Q616	217	E9Q616	S	1	21.8118	1.16E-17	199.41	Ahnak
E9Q616_S_5504_1	0	0	0	0	0	0	649	E9Q616	5504	E9Q616	S	1	23.9253	0.0472724	65.467	Ahnak
E9Q855_S_78_1	0	79977000	141350000	0	1.314428806	0.659922203	663	E9Q855	78	E9Q855	S	1	59.8255	0.0297413	148.32	Scamp3
E9Q8T1_S_2796_2	0	0	48499000	0	0	0.226427782	668	E9Q8T1	2796	E9Q8T1	S	2	44.2941	0.0479074	44.294	Tacc2
E9Q8T1_Y_2794_2	0	0	48499000	0	0	0.226427782	668	E9Q8T1	2794	E9Q8T1	Y	2	44.2941	0.0479074	44.294	Tacc2
E9Q9Q7_S_110_1	11417000	8905000	77705000	0.144906694	0.146354433	0.362782135	620	E9Q9Q7	110	E9Q9Q7	S	1	20.6083	0.00000958	162.36	Ablim1
E9Q9Q7_S_209_1	0	0	20267000	0	0	0.094620752	620	E9Q9Q7	209	E9Q9Q7	S	1	9.34225	0.0341203	102.28	Ablim1
E9Q9Q7_S_84_1	0	53700000	30591000	0	0.882564073	0.142820517	620	E9Q9Q7	84	E9Q9Q7	S	1	57.2063	0.0316991	104.43	Ablim1
E9Q9Q7_S_89_1	18444000	25464000	274900000	0.234094688	0.418503009	1.283428466	620	E9Q9Q7	89	E9Q9Q7	S	1	30.3516	0.00225526	123.29	Ablim1

E9Q9T8_S_281_1	3375000000	5965800000	5846300000	42.83612955	98.04843105	27.29468112	670	E9Q9T8	281	E9Q9T8	S	1	17.2372	0.0400393	94.616	Mybpc3
E9QA56_T_60_2	20783000	5121600	0	0.263781713	0.084173932	0	615	E9QA56	60	E9QA56	T	2	40.4755	0.0570432	40.475	Adam3
E9QA56_Y_59_2	20783000	5121600	0	0.263781713	0.084173932	0	615	E9QA56	59	E9QA56	Y	2	40.4755	0.0570432	40.475	Adam3
E9QKA4_S_220_1;2	675230000	729000000	3227100000	8.570145112	11.98117708	15.06639506	368	E9QKA4	220	E9QKA4	S	1;2	47.559	0.00625355	84.289	Srrm1
E9QKA4_S_227_2	675230000	729000000	3227100000	8.570145112	11.98117708	15.06639506	368	E9QKA4	227	E9QKA4	S	2	47.559	0.0332182	59.067	Srrm1
E9QKA4_S_448_1	17538000	3219000	37058000	0.222595567	0.052904539	0.173013067	368	E9QKA4	448	E9QKA4	S	1	8.04621	0.0056372	79.875	Srrm1
E9QN69_S_956_1	0	53570000	74029000	0	0.880427512	0.345619956	681	E9QN69	956	E9QN69	S	1	94.3023	0.0278209	94.302	Pcnxl4
E9QP49_S_1462_1	0	0	29077000	0	0	0.13575209	683	E9QP49	1462	E9QP49	S	1	28.9788	0.0214809	99.53	Ehbp1l1
E9QQ25_S_2114_1	13033000	0	25532000	0.165417267	0	0.119201512	688	E9QQ25	2114	E9QQ25	S	1	14.6437	0.0000881	143.28	Speg
E9QQ25_S_2413_1	0	0	25451000	0	0	0.118823346	688	E9QQ25	2413	E9QQ25	S	1	9.85053	0.0203161	108.1	Speg
E9QQ25_S_2777_1	0	0	0	0	0	0	688	E9QQ25	2777	E9QQ25	S	1	31.6606	0.000749089	94.551	Speg
E9QQ25_S_860_1	6994600	16504000	9324400	0.088776768	0.271244645	0.043532922	688	E9QQ25	860	E9QQ25	S	1	30.9349	0.00319718	116.78	Speg
F2Z3X7_S_192_1	0	0	0	0	0	0	689	F2Z3X7	192	F2Z3X7	S	1	34.6885	0.00000969	124.12	Raph1
F6TAZ4_S_27_1	3860900	5214700	7510000	0.049003263	0.085704039	0.035062014	703	F6TAZ4	27	F6TAZ4	S	1	30.7246	0.0449822	90.827	Rbm20
F6TFN2_S_1092_1	0	0	0	0	0	0	705	F6TFN2	1092	F6TFN2	S	1	11.7965	0.0266708	66.056	Lmo7
F6ZGN3_S_242_1	58877000	46242000	201110000	0.747277866	0.759991208	0.938924332	714	F6ZGN3	242	F6ZGN3	S	1	24.0602	0.0303655	103.88	Myo18a
F6ZGN3_S_340_1	0	0	81302000	0	0	0.379575486	714	F6ZGN3	340	F6ZGN3	S	1	84.0612	0.0268451	136.33	Myo18a
G3UX38_S_329_2	0	0	0	0	0	0	28	G3UX38	329	G3UX38	S	2	42.8692	0.0409493	42.869	Arhgap21
G3UX38_S_330_2	0	0	0	0	0	0	28	G3UX38	330	G3UX38	S	2	42.8692	0.0409493	42.869	Arhgap21
G3XA35_S_1321_1	9361700	0	36414000	0.118820443	0	0.170006417	609	G3XA35	1321	G3XA35	S	1	100.878	0.0554442	100.88	Vcan
G5E8J6_S_324_1	0	0	0	0	0	0	772	G5E8J6	324	G5E8J6	S	1	103.863	8.61E-26	190.23	Hrc
H3BIW0_S_554_1	0	0	0	0	0	0	762	H3BIW0	554	H3BIW0	S	1	8.38955	0.00689956	82.171	Zc3h18
H7BX95_S_199_1	0	0	32367000	0	0	0.151112147	788	H7BX95	199	H7BX95	S	1	6.64791	0.0552517	50.653	Srsf1
J3QN49_T_5_1	47835000	44301000	0	0.607130743	0.728090708	0	796	J3QN49	5	J3QN49	T	1	29.3274	0.0582801	40.589	Esp3
J3QNR9_Y_61_2	19141000	33185000	0	0.242941142	0.545398301	0	797	J3QNR9	61	J3QNR9	Y	2	11.5623	0.053075	40.724	1110059E24Rik
J3QPI3_T_14_1	11743000	8234800	31994000	0.149044346	0.135339639	0.149370718	385	J3QPI3	14	J3QPI3	T	1	18.9338	0.0501474	48.741	Klhl32
O35367_T_109_2	0	144160000	0	0	2.369281877	0	827	O35367	109	O35367	T	2	104.167	0.00289838	104.17	Kera
O35367_Y_111_2	0	144160000	0	0	2.369281877	0	827	O35367	111	O35367	Y	2	104.167	0.00289838	104.17	Kera

O35738_T_372_2	10273000	0	32357000	0.130386832	0	0.15106546	835	O35738	372	O35738	T	2	14.082	0.0523916	46.706	Klf12
O35738_Y_368_2	10273000	0	32357000	0.130386832	0	0.15106546	835	O35738	368	O35738	Y	2	11.8858	0.0523916	46.706	Klf12
O35887_Y_47_1	82681000	4449300	67965000	1.049402675	0.073124624	0.317308897	837;1406	O35887;Q6XLQ8	47	O35887	Y	1	118.156	0.0000056	118.16	Calu
O35954_T_947_2	12153000	10890000	0	0.154248143	0.178978077	0	838	O35954	947	O35954	T	2	25.8399	0.0586597	41.704	Pitpnm1
O35954_Y_940_2	12153000	10890000	0	0.154248143	0.178978077	0	838	O35954	940	O35954	Y	2	25.3266	0.0586597	41.704	Pitpnm1
O54724_S_169_1	95280000	38930000	210820000	1.209311533	0.639817865	0.984257509	839	O54724	169	O54724	S	1	14.6303	0.0414199	100.82	Ptrf
O54724_S_42_1	0	7708600	102440000	0	0.126691497	0.478262685	839	O54724	42	O54724	S	1	13.638	0.000696781	136.6	Ptrf
O55143-2_S_663_1	16599000	36242000	597220000	0.210677604	0.595640356	2.788247175	851	O55143-2	663	O55143-2	S	1	38.896	0.00000298	180.24	Atp2a2
O70548_S_161_1	149620000	77040000	135170000	1.899004949	1.266158961	0.631069573	863	O70548	161	O70548	S	1	24.4793	0.00539791	78.655	Tcap
P04247_S_121_1	0	0	41896000	0	0	0.195600287	286;889	A0A2R8W6F8;P04247	121	P04247	S	1	74.4422	0.000214284	112.82	Mb
P05064_S_36_1	0	0	69287000	0	0	0.323480932	891	P05064	36	P05064	S	1	8.53144	0.000719353	138.77	Aldoa;Aldoart2
P05064_S_39_1	0	0	0	0	0	0	891	P05064	39	P05064	S	1	24.0459	2.67E-14	160.65	Aldoa;Aldoart2
P05064_S_46_1	0	0	16521000	0	0	0.077131763	891	P05064	46	P05064	S	1	29.5652	0.00682721	115.12	Aldoa;Aldoart1;Aldoart2
P07901_S_231_1	0	0	25745000	0	0	0.120195947	907	P07901	231	P07901	S	1	235.209	9.76E-26	235.21	Hsp90aa1
P11499_S_255_1	75578000	60192000	776420000	0.959250074	0.989260646	3.624880063	938	P11499	255	P11499	S	1	85.0408	2.88E-29	238.32	Hsp90ab1
P11499_Y_484_2	0	0	0	0	0	0	938	P11499	484	P11499	Y	2	9.64821	0.0274387	48.796	Hsp90ab1
P13541_S_1066_1	124360000	0	163720000	1.578400317	0	0.764361253	946	P13541	1066	P13541	S	1	163.332	0.000000472	163.33	Myh3
P14152_S_241_1	83466000	50947000	179560000	1.059366041	0.837318284	0.838313624	951	P14152	241	P14152	S	1	14.1822	0.0224995	62.466	Mdh1
P14602-3_S_15_1	0	11564000	417750000	0	0.190055325	1.950353734	954	P14602-3	15	P14602-3	S	1	59.547	0.011263	136.57	Hspb1
P14602-3_S_52_1	39227000	48426000	237780000	0.497876401	0.795885434	1.110125939	954	P14602-3	52	P14602-3	S	1	25.9706	0.00943768	110.12	Hspb1
P14873_S_561_1	12823000	0	0	0.162751908	0	0	957	P14873	561	P14873	S	1	56.5548	0.000225722	162.88	Map1b
P20152_S_73_1	424130000	21651000	205580000	5.383137074	0.355836029	0.959793467	978	P20152	73	P20152	S	1	8.89287	0.041851	100.48	Vim
P26039_S_2040_1	0	0	16032000	0	0	0.074848764	994	P26039	2040	P26039	S	1	156.139	0.0000452	156.14	Tln1
P26231_S_641_1	0	0	30668000	0	0	0.143180008	997	P26231	641	P26231	S	1	59.3128	1.48E-32	256.45	Ctnna1
P26231_S_655_1	0	0	39935000	0	0	0.186444946	997	P26231	655	P26231	S	1	4.82008	0.000267582	142.85	Ctnna1;Ctnna2
P27546-3_S_1008_1	0	0	28648000	0	0	0.133749213	1003	P27546-3	1008	P27546-3	S	1	164.01	0.000000548	164.01	Map4
P28667_T_85_1	21744000	0	14208000	0.275978904	0	0.066333036	1014	P28667	85	P28667	T	1	81.0022	0.0534579	81.002	Marcksl1
P35486_S_232_1	0	79910000	3652900	0	1.313327655	0.017054332	1031	P35486	232	P35486	S	1	17.8431	0.0394049	110.38	Pdha1

P35486_S_293_1	18411000	0	411230000	0.233675846	0	1.919913743	1031	P35486	293	P35486	S	1	28.9292	0.00990591	117.7	Pdha1
P35564_S_563_1	0	0	20303000	0	0	0.094788826	1032	P35564	563	P35564	S	1	38.0109	7.91E-12	187.75	Canx
P35564_S_582_1	0	11583000	181970000	0	0.190367591	0.849565216	1032	P35564	582	P35564	S	1	138.02	0.00076079	146.96	Canx
P36552_S_233_3	18455000	54174000	44334000	0.234234302	0.890354303	0.206982603	1036	P36552	233	P36552	S	3	44.3175	0.0498013	44.318	Cpox
P36552_S_234_3	18455000	54174000	44334000	0.234234302	0.890354303	0.206982603	1036	P36552	234	P36552	S	3	44.3175	0.0498013	44.318	Cpox
P36552_T_228_3	18455000	54174000	44334000	0.234234302	0.890354303	0.206982603	1036	P36552	228	P36552	T	3	44.3175	0.0498013	44.318	Cpox
P43274_S_2_1	0	0	49837000	0	0	0.232674516	1048	P43274	2	P43274	S	1	7.80686	0.00735604	86.497	Hist1h1e
P43276_S_18_1	10532000	0	30424000	0.133674109	0	0.142040843	1050	P43276	18	P43276	S	1	43.7998	0.000146973	118.23	Hist1h1b
P47757_S_263_1	0	6337200	79250000	0	0.104152422	0.369995293	1060	P47757	263	P47757	S	1	61.4535	0.0127617	113.41	Capzb
P48678_S_22_1	27959000	0	0	0.354860843	0	0	1066	P48678	22	P48678	S	1	7.42661	0.0017382	130.72	Lmna
P48678_S_390_1	93413000	14272000	103190000	1.185615221	0.234561535	0.481764218	1066	P48678	390	P48678	S	1	20.0427	0.0317407	101.72	Lmna
P48787_S_6_1	0	0	49667000	0	0	0.231880835	1070	P48787	6	P48787	S	1	9.05515	0.000388185	111.43	Tnni3
P48962_S_149_1	45141000	32196000	54948000	0.572937992	0.529144002	0.256536294	1071	P48962	149	P48962	S	1	11.6803	0.0533003	95.854	Slc25a4
P50462_S_111_1	10526000	9908400	6690000	0.133597955	0.162845398	0.031233672	1078	P50462	111	P50462	S	1	10.5276	0.0357721	101.32	Csrp3
P51944-3_S_575_1	0	0	0	0	0	0	1091	P51944-3	575	P51944-3	S	1	46.4258	0.0596033	46.426	Ccnf
P53986_S_213_1	338970000	437200000	847760000	4.302270469	7.185419232	3.957945857	1099	P53986	213	P53986	S	1	70.4503	0.000169253	175.48	Slc16a1
P53986_S_461_1	59241000	27100000	106870000	0.751897822	0.445390808	0.498945071	1099	P53986	461	P53986	S	1	13.0249	1.01E-74	321.02	Slc16a1
P58771_S_283_1	0	10805000	4136200000	0	0.177581095	19.31071961	1120	P58771	283	P58771	S	1	6.78584	0.00000379	154.84	Tpm1
P59017_S_387_1	0	26925000	69036000	0	0.442514668	0.322309085	1124	P59017	387	P59017	S	1	12.7979	0.017191	97.456	Bcl2l13
P61014_S_16_1;2	82600000	61692000	107440000	1.048374608	1.013913274	0.501606236	1128	P61014	16	P61014	S	1;2	40.373	0.00105822	101.45	Pln
P61014_T_17_2	0	0	31476000	0	0	0.146952326	1128	P61014	17	P61014	T	2	40.373	0.0475852	45.79	Pln
P62754_S_240_1	9565800	0	9843400	0.121410918	0	0.045955983	1155	P62754	240	P62754	S	1	10.6023	0.0468454	95.498	Rps6
P70670_T_590_1	0	11321000	42868000	0	0.186061599	0.200138274	1190	P70670	590	P70670	T	1	20.071	0.0517474	55.401	Naca
Q02257_S_182_1	0	0	114250000	0	0	0.533400154	1219	Q02257	182	Q02257	S	1	117.916	0.00982067	117.92	Jup
Q02566_S_1368_1	0	0	40050000	0	0	0.186981848	436;946;1220;1313;1626	B1AR69;P13541;Q02566;Q5SX39;Q91Z83	1368	Q02566	S	1	106.291	0.0172346	106.29	Myh13;Myh3;Myh6;Myh4;Myh7
Q02566_T_2_1	11124000	13021000	0	0.141187883	0.214001244	0	1220	Q02566	2	Q02566	T	1	52.1059	0.0281771	55.885	Myh6
Q0VBL3-2_S_293_1	0	0	26652000	0	0	0.124430467	1235	Q0VBL3-2	293	Q0VBL3-2	S	1	24.8161	0.0373016	93.551	Rbm15
Q14BI5_S_76_1	0	0	45781000	0	0	0.213738227	1239	Q14BI5	76	Q14BI5	S	1	16.7294	0.0189732	99.392	Myom2

Q3T9T1_S_293_2	29898000	18820000	47118000	0.379470993	0.309308303	0.219980293	351	Q3T9T1	293	Q3T9T1	S	2	64.3739	0.0528171	64.374	Tmcc2
Q3T9T1_S_298_2	29898000	18820000	47118000	0.379470993	0.309308303	0.219980293	351	Q3T9T1	298	Q3T9T1	S	2	64.3739	0.0528171	64.374	Tmcc2
Q3TYF1_S_299_3	0	0	147470000	0	0	0.68849471	1260	Q3TYF1	299	Q3TYF1	S	3	15.4864	0.0527591	40.725	Nab2
Q3TYF1_Y_298_3	0	0	147470000	0	0	0.68849471	1260	Q3TYF1	298	Q3TYF1	Y	3	14.6793	0.0527591	40.725	Nab2
Q3TYF1_Y_302_3	0	0	147470000	0	0	0.68849471	1260	Q3TYF1	302	Q3TYF1	Y	3	22.3602	0.0527591	40.725	Nab2
Q3U3J1_S_338_1	32250000	0	232390000	0.409323016	0	1.08496159	1076	Q3U3J1	338	Q3U3J1	S	1	11.7833	1.04E-21	234.85	Bckdha
Q3U3Q1-2_S_339_2	79215000	41909000	26722000	1.005411556	0.688777984	0.124757277	1264	Q3U3Q1-2	339	Q3U3Q1-2	S	2	67.1532	0.0183967	69.721	Ulk3
Q3U3Q1-2_Y_337_2	79215000	41909000	26722000	1.005411556	0.688777984	0.124757277	1264	Q3U3Q1-2	337	Q3U3Q1-2	Y	2	67.1532	0.0183967	69.721	Ulk3
Q3UID0_S_302_2	0	0	12379000	0	0	0.057793965	1275	Q3UID0	302	Q3UID0	S	2	20.7432	0.0528414	91.307	Smarcc2
Q3UKG2_S_372_1	6782000	1096000	15736000	0.086078409	0.018012853	0.073466826	17	Q3UKG2	372	Q3UKG2	S	1	44.453	0.00892035	87.895	Prob1
Q3UPY5_S_180_2	13747000	9662400	0	0.174479488	0.158802367	0	1284	Q3UPY5	180	Q3UPY5	S	2	49.7151	0.0426735	49.715	Glb1l2
Q3UPY5_Y_174_2	13747000	9662400	0	0.174479488	0.158802367	0	1284	Q3UPY5	174	Q3UPY5	Y	2	49.7151	0.0426735	49.715	Glb1l2
Q3UTJ2-2_S_1212_1	0	0	74467000	0	0	0.347664851	1288	Q3UTJ2-2	1212	Q3UTJ2-2	S	1	6.57578	0.00010258	144.3	Sorbs2
Q3UTJ2-2_S_345_1	11972000	4846200	90266000	0.151950857	0.07964771	0.421425805	1288	Q3UTJ2-2	345	Q3UTJ2-2	S	1	23.6243	0.00000656	167.89	Sorbs2
Q3UTJ2-2_S_373_1	69948000	0	779450000	0.887793064	0	3.639026256	1288	Q3UTJ2-2	373	Q3UTJ2-2	S	1	8.69187	0.00166869	142.08	Sorbs2
Q3UTJ2-2_S_454_1	0	24544000	314300000	0	0.40338273	1.467375652	1288	Q3UTJ2-2	454	Q3UTJ2-2	S	1	47.5062	0.022467	66.004	Sorbs2
Q3UVT7_S_278_1	8886300	8344700	146400000	0.112786577	0.137145855	0.68349919	115	Q3UVT7	278	Q3UVT7	S	1	94.6917	0.00272769	125.97	Palmd
Q3UZA1-2_S_90_1	0	0	20677000	0	0	0.096534923	1290	Q3UZA1-2	90	Q3UZA1-2	S	1	29.1533	0.00011001	116.48	Rcsd1
Q5DTJ9_S_129_1	28057000	0	31290000	0.356104678	0	0.146083946	1301	Q5DTJ9	129	Q5DTJ9	S	1	41.5218	0.0000163	162.7	Mypn
Q5DTJ9_S_924_1	0	0	34954000	0	0	0.1631901	1301	Q5DTJ9	924	Q5DTJ9	S	1	46.4421	0.000273816	98.74	Mypn
Q5EBG6_S_16_1	16746000	16934000	545400000	0.212543356	0.278311732	2.546314606	1303	Q5EBG6	16	Q5EBG6	S	1	71.2266	0.00394569	121.45	Hspb6
Q5EBP8_S_6_1	28940000	4737800	16207000	0.367311878	0.077866146	0.075665788	1072	Q5EBP8	6	Q5EBP8	S	1	30.0465	0.000695314	108.66	Hnrnpa1
Q5F226_S_2534_4	0	0	58296000	0	0	0.272167137	1304	Q5F226	2534	Q5F226	S	4	40.1078	0.0369756	41.092	Fat2
Q5F226_T_2538_4	0	0	58296000	0	0	0.272167137	1304	Q5F226	2538	Q5F226	T	4	38.1583	0.0369756	41.092	Fat2
Q5F226_T_2539_4	0	0	58296000	0	0	0.272167137	1304	Q5F226	2539	Q5F226	T	4	38.1583	0.0369756	41.092	Fat2
Q5GIG6_S_827_1	7205000	11443000	13560000	0.091447204	0.188066679	0.063307712	1306	Q5GIG6	827	Q5GIG6	S	1	15.6226	0.00413419	128.85	Tnni3k
Q60575-3_S_663_2	0	0	0	0	0	0	380	Q60575-3	663	Q60575-3	S	2	28.5228	0.0313778	53.377	Kif1b
Q60575-3_S_665_2	0	0	0	0	0	0	380	Q60575-3	665	Q60575-3	S	2	19.59	0.0313778	53.377	Kif1b

Q60668-3_S_82_1	40465000	0	77925000	0.513589328	0	0.363809251	1319	Q60668-3	82	Q60668-3	S	1	10.9146	9.06E-83	331.8	Hnrnpd
Q61686_S_93_1	0	0	0	0	0	0	1346	Q61686	93	Q61686	S	1	12.7785	0.00197868	89.507	Cbx5
Q61699-2_S_766_1	0	0	0	0	0	0	1347	Q61699-2	766	Q61699-2	S	1	60.5185	0.00873622	76.228	Hsph1
Q62261_S_2102_1	268730000	119790000	363360000	3.410771287	1.968758851	1.69642258	1356	Q62261	2102	Q62261	S	1	93.1586	0.00414152	128.81	Sptbn1
Q64727_S_290_1	0	0	29830000	0	0	0.139267629	1371	Q64727	290	Q64727	S	1	110.14	0.000507293	110.14	Vcl
Q65CL1_S_637_1	0	0	44467000	0	0	0.207603542	1372	Q65CL1	637	Q65CL1	S	1	163.258	1.79E-53	283.22	Ctnna3
Q65CL1_S_650_1	12004000	8618700	14917000	0.152357007	0.141649068	0.069643152	1372	Q65CL1	650	Q65CL1	S	1	13.8042	0.0141629	102.4	Ctnna3
Q69Z38_S_282_1	8148700	5557000	0	0.10342482	0.091329768	0	1373	Q69Z38	282	Q69Z38	S	1	47.1223	0.0469605	94.309	Peak1
Q6P1H6-3_S_699_1	52776000	36837000	53952000	0.669842836	0.605419232	0.251886259	1387	Q6P1H6-3	699	Q6P1H6-3	S	1	91.6263	0.00981445	132.29	Ankle2
Q6P542_S_194_1	0	0	50035000	0	0	0.233598921	1390	Q6P542	194	Q6P542	S	1	74.5078	0.0000235	155.86	Abcf1
Q6P542_S_225_1	0	0	0	0	0	0	1390	Q6P542	225	Q6P542	S	1	119.458	0.00000108	119.46	Abcf1
Q6ZQ06_T_1108_2	0	0	0	0	0	0	1407	Q6ZQ06	1108	Q6ZQ06	T	2	46.6059	0.0253931	57.785	Cep162
Q6ZQ06_T_1110_2	0	0	0	0	0	0	1407	Q6ZQ06	1110	Q6ZQ06	T	2	46.6059	0.0253931	57.785	Cep162
Q7TMI3-3_T_13_2	25161000	22414000	50095000	0.319348105	0.368375999	0.233879043	1423	Q7TMI3-3	13	Q7TMI3-3	T	2	7.98905	0.0428271	71.279	Uhrf2
Q7TMI3-3_T_15_2	25161000	22414000	50095000	0.319348105	0.368375999	0.233879043	1423	Q7TMI3-3	15	Q7TMI3-3	T	2	5.32353	0.0428271	71.279	Uhrf2
Q7TPM0_S_89_1	0	0	72605000	0	0	0.338971712	1196	Q7TPM0	89	Q7TPM0	S	1	9.24322	0.00240487	118.24	Cbx1
Q7TSG3_S_250_2	0	0	375330000	0	0	1.752307043	1428	Q7TSG3	250	Q7TSG3	S	2	45.5843	0.0411287	52.247	Fbxo5
Q7TSG3_S_253_2	0	0	375330000	0	0	1.752307043	1428	Q7TSG3	253	Q7TSG3	S	2	45.0105	0.0411287	52.247	Fbxo5
Q7TT37_T_936_2	84223000	8235800	262920000	1.068974026	0.135356074	1.227497316	1430	Q7TT37	936	Q7TT37	T	2	8.76445	0.0328943	82.749	Ikbkap
Q7TT37_Y_932_2	84223000	8235800	262920000	1.068974026	0.135356074	1.227497316	1430	Q7TT37	932	Q7TT37	Y	2	7.37118	0.0328943	82.749	Ikbkap
Q80UU9_T_205_1	0	0	70070000	0	0	0.327136532	1433	Q80UU9	205	Q80UU9	T	1	34.1598	0.00000133	119.35	Pgrmc2
Q80ZA0_T_14_1	0	0	422840000	0	0	1.97411747	1444	Q80ZA0	14	Q80ZA0	T	1	48.3941	0.0334436	57.348	Itln1b
Q8BGC0_S_441_1;2	0	0	8093500	0	0	0.037786207	1454	Q8BGC0	441	Q8BGC0	S	1;2	56.8168	0.0272711	94.882	Htatsf1
Q8BGC0_S_446_2	0	0	0	0	0	0	1454	Q8BGC0	446	Q8BGC0	S	2	56.8168	0.0428839	56.817	Htatsf1
Q8BGD9_S_422_1	19412000	6243300	10403000	0.246380725	0.102609167	0.048568593	1456	Q8BGD9	422	Q8BGD9	S	1	24.9064	0.00000205	168.45	Eif4b
Q8BGD9_S_497_1	0	0	9283300	0	0	0.043341038	1456	Q8BGD9	497	Q8BGD9	S	1	14.6301	0.000856579	123.4	Eif4b
Q8BGE5-4_S_1129_1	0	0	0	0	0	0	1457	Q8BGE5-4	1129	Q8BGE5-4	S	1	26.6892	0.0463411	40.268	Fancm
Q8BGU5-2_S_301_1	0	0	41694000	0	0	0.194657208	1459	Q8BGU5-2	301	Q8BGU5-2	S	1	21.2089	0.00041053	150.06	Ccny

Q8BJU0-2_S_306_1	15194000	4406100	30541000	0.192845082	0.072414629	0.142587082	1471	Q8BJU0-2	306	Q8BJU0-2	S	1	11.9453	0.00269062	133.87	Sgta
Q8BJU0-2_T_304_1	7312500	0	0	0.092811614	0	0	1471	Q8BJU0-2	304	Q8BJU0-2	T	1	7.36477	0.0418084	89.466	Sgta
Q8BJU0-2_T_81_1	0	0	0	0	0	0	1471	Q8BJU0-2	81	Q8BJU0-2	T	1	25.4816	0.0113802	74.141	Sgta
Q8BND3-2_S_460_2	33505000	15275000	577180000	0.42525171	0.251045926	2.69468622	1485	Q8BND3-2	460	Q8BND3-2	S	2	87.5248	0.00650955	90.653	Wdr35
Q8BND3-2_T_458_2	33505000	15275000	577180000	0.42525171	0.251045926	2.69468622	1485	Q8BND3-2	458	Q8BND3-2	T	2	87.5248	0.00650955	90.653	Wdr35
Q8BUZ1_S_150_1	0	0	144060000	0	0	0.672574408	1497	Q8BUZ1	150	Q8BUZ1	S	1	14.7303	0.00153312	144.47	Abra
Q8BVD5-3_Y_395_1	39340000	42864000	0	0.499310618	0.70447349	0	1498	Q8BVD5-3	395	Q8BVD5-3	Y	1	14.9548	0.0339446	53.754	
Q8BZN7_S_243_1	24366000	0	45128000	0.309257817	0	0.210689559	1300	Q8BZN7	243	Q8BZN7	S	1	50.5943	0.0179613	105.13	Thrap3
Q8BZN7_S_248_1	0	0	0	0	0	0	1300	Q8BZN7	248	Q8BZN7	S	1	97.4306	0.0121408	97.431	Thrap3
Q8C0E3_S_184_1	0	0	0	0	0	0	1513	Q8C0E3	184	Q8C0E3	S	1	65.2463	0.0360958	65.246	Trim47
Q8C120-4_S_11_2	71576000	18838000	134790000	0.908455943	0.309604134	0.629295463	1515	Q8C120-4	11	Q8C120-4	S	2	58.9807	0.0330623	58.981	Sh3rf3
Q8C120-4_S_6_2	71576000	18838000	134790000	0.908455943	0.309604134	0.629295463	1515	Q8C120-4	6	Q8C120-4	S	2	58.9807	0.0330623	58.981	Sh3rf3
Q8C142_S_198_1	22318000	0	18138000	0.283264219	0	0.084681068	1517	Q8C142	198	Q8C142	S	1	12.592	0.0590354	94.363	Ldlrap1
Q8C419_S_904_3	0	4734300	76684000	0	0.077808624	0.358015382	1519	Q8C419	904	Q8C419	S	3	31.1596	0.0484909	42.118	Gpr158
Q8C419_S_908_3	0	4734300	76684000	0	0.077808624	0.358015382	1519	Q8C419	908	Q8C419	S	3	32.8835	0.0484909	42.118	Gpr158
Q8C4C9_Y_342_1	0	0	0	0	0	0	1520	Q8C4C9	342	Q8C4C9	Y	1	45.7179	0.0588471	45.718	Dnajb12
Q8CG46-2_S_5_1	65396000	0	159200000	0.83001823	0	0.743258682	1532	Q8CG46-2	5	Q8CG46-2	S	1	5.733	0.0326571	56.087	Smc5
Q8JZZ5_S_66_3	41998000	16391000	25536000	0.53304645	0.269387481	0.119220187	1098	Q8JZZ5	66	Q8JZZ5	S	3	35.7438	0.0537325	41.117	Pitpnb
Q8JZZ5_Y_62_3	41998000	16391000	25536000	0.53304645	0.269387481	0.119220187	1098	Q8JZZ5	62	Q8JZZ5	Y	3	23.2207	0.0537325	41.117	Pitpnb
Q8K310_S_598_2	0	0	28761000	0	0	0.134276777	1554	Q8K310	598	Q8K310	S	2	31.0258	0.00300348	122.96	Matr3
Q8K310_S_604_2	0	0	28761000	0	0	0.134276777	1554	Q8K310	604	Q8K310	S	2	24.5496	0.00300348	122.96	Matr3
Q8K440-2_S_1483_2	0	0	70990000	0	0	0.331431745	1560	Q8K440-2	1483	Q8K440-2	S	2	32.5438	0.0413739	41.502	Abca8b
Q8K440-2_Y_1487_2	0	0	70990000	0	0	0.331431745	1560	Q8K440-2	1487	Q8K440-2	Y	2	32.2616	0.0413739	41.502	Abca8b
Q8K4L2_S_960_1	0	0	47970000	0	0	0.223958034	187	Q8K4L2	960	Q8K4L2	S	1	59.6221	0.0000673	121.86	Svil
Q8N7N5-2_S_100_1	14363000	0	55557000	0.182297875	0	0.259379539	1563	Q8N7N5-2	100	Q8N7N5-2	S	1	180.967	4.23E-09	180.97	Dcaf8
Q8R574_S_233_2	12081000	0	120150000	0.153334305	0	0.560945544	1583	Q8R574	233	Q8R574	S	2	66.2625	0.054007	66.262	Prpsap2
Q8R574_S_240_2	12081000	0	120150000	0.153334305	0	0.560945544	1583	Q8R574	240	Q8R574	S	2	66.2625	0.054007	66.262	Prpsap2
Q8VBT0_S_245_1	0	0	42898000	0	0	0.200278335	1587	Q8VBT0	245	Q8VBT0	S	1	65.6903	6.87E-11	154.43	Tmx1

Q8VDD5_S_1943_1	72767000	5359800	65583000	0.923572337	0.088088769	0.306188029	1595	Q8VDD5	1943	Q8VDD5	S	1	58.9628	3.81E-11	190.19	Myh9
Q8VDN2_S_16_1	16335000	37523000	11317000	0.207326867	0.6166937	0.052835795	1597	Q8VDN2	16	Q8VDN2	S	1	78.7354	0.0251219	98.942	Atp1a1
Q8VEK3-2_S_4_1	0	19513000	0	0	0.320697817	0	1599	Q8VEK3-2	4	Q8VEK3-2	S	1	8.86742	0.0362337	46.426	Hnrnpu
Q8VGX2_T_301_2	0	0	68301000	0	0	0.318877583	1600	Q8VGX2	301	Q8VGX2	T	2	7.2869	0.0403087	45.704	Olfr166
Q8VHX6-2_S_2201_1	0	20243000	458750000	0	0.332695429	2.141770857	1602	Q8VHX6-2	2201	Q8VHX6-2	S	1	44.7354	0.0262774	96.171	Flnc
Q91VL0_S_114_1	10659000	0	34607000	0.135286016	0	0.161570058	776	Q91VL0	114	Q91VL0	S	1	43.3132	1.73E-20	217.79	Bnip2
Q920Q2_T_798_2	0	0	70581000	0	0	0.329522243	1629	Q920Q2	798	Q920Q2	T	2	45.161	0.0518725	45.161	Rev1
Q920Q2_T_804_2	0	0	70581000	0	0	0.329522243	1629	Q920Q2	804	Q920Q2	T	2	45.161	0.0518725	45.161	Rev1
Q921W0_T_11_1	18808000	7337600	47075000	0.238714644	0.120594081	0.219779538	1635	Q921W0	11	Q921W0	T	1	18.2168	0.0437898	84.479	Chmp1a
Q924W5_Y_901_1	0	0	0	0	0	0	1642	Q924W5	901	Q924W5	Y	1	67.9926	0.0184195	67.993	Smc6
Q99L43_S_32_1	18627000	6238200	24330000	0.236417359	0.102525348	0.113589722	1663	Q99L43	32	Q99L43	S	1	17.0924	0.0120292	107.65	Cds2
Q99LI7_Y_343_2	66368000	8702600	52076000	0.842355036	0.143027972	0.243127758	1669	Q99LI7	343	Q99LI7	Y	2	40.4886	0.0532816	44.847	Cstf3
Q99LI7_Y_346_2	66368000	8702600	52076000	0.842355036	0.143027972	0.243127758	1669	Q99LI7	346	Q99LI7	Y	2	40.4886	0.0532816	44.847	Cstf3
Q9CQ73_S_135_1	35958000	25923000	106500000	0.456385643	0.426046713	0.497217649	1697	Q9CQ73	135	Q9CQ73	S	1	49.8777	1.02E-27	242.19	Pkp2
Q9CQ73_S_151_2	0	0	15874000	0	0	0.074111108	1697	Q9CQ73	151	Q9CQ73	S	2	49.6804	0.0226434	84.365	Pkp2
Q9CQ73_S_154_1;2	0	0	125880000	0	0	0.587697255	1697	Q9CQ73	154	Q9CQ73	S	1;2	5.55105	0.0226434	89.43	Pkp2
Q9CQ73_S_155_1	0	0	110010000	0	0	0.513604822	1697	Q9CQ73	155	Q9CQ73	S	1	6.82176	0.00853917	113.99	Pkp2
Q9D1T2_S_79_1	0	0	26828000	0	0	0.12525216	1747	Q9D1T2	79	Q9D1T2	S	1	104.206	0.00407233	104.21	Mxra7
Q9D338_S_18_2	193160000	94534000	360030000	2.451622751	1.553674341	1.680875775	1780	Q9D338	18	Q9D338	S	2	54.2655	0.0526462	63.48	Mrpl19
Q9D338_Y_14_2	193160000	94534000	360030000	2.451622751	1.553674341	1.680875775	1780	Q9D338	14	Q9D338	Y	2	53.8918	0.0526462	63.48	Mrpl19
Q9D5W8_S_11_1	0	9576800	15623000	0	0.157395524	0.072939261	1786	Q9D5W8	11	Q9D5W8	S	1	43.1252	0.0499241	45.161	
Q9D7J6_S_121_2	0	0	0	0	0	0	1794	Q9D7J6	121	Q9D7J6	S	2	31.8266	0.0434907	44.863	Dnase1l1
Q9D8U8_Y_310_2	123510000	50414000	328300000	1.567611959	0.828558383	1.532737597	1806	Q9D8U8	310	Q9D8U8	Y	2	34.8656	0.0521665	40.589	Snx5
Q9DBC7_Y_53_1	10013000	13424000	9202000	0.127086864	0.220624583	0.042961472	1814	Q9DBC7	53	Q9DBC7	Y	1	63.5651	0.0479226	63.565	Prkar1a
Q9DC77_S_36_1	14970000	0	132940000	0.190002032	0	0.620658349	1823	Q9DC77	36	Q9DC77	S	1	13.4317	2.73E-17	215.65	Smpx
Q9ESX5_S_508_1	11860000	4596800	0	0.150529332	0.075548799	0	1852	Q9ESX5	508	Q9ESX5	S	1	110.026	0.00327993	110.03	Dkc1
Q9ESX5_T_8_1	0	0	0	0	0	0	1852	Q9ESX5	8	Q9ESX5	T	1	47.6028	0.040662	47.603	Dkc1
Q9ET54-3_S_173_1	0	0	17182000	0	0	0.080217781	1854	Q9ET54-3	173	Q9ET54-3	S	1	71.8161	0.00301737	124.21	Palld

Q9ET54-3_S_401_1	0	0	45686000	0	0	0.213294699	1854	Q9ET54-3	401	Q9ET54-3	S	1	40.7094	4.89E-09	187.78	Palld
Q9JI91_S_147_1	0	0	19157000	0	0	0.089438484	354;1117;1860	A1BN54;P57780;Q9JI91	147	Q9JI91	S	1	29.6424	0.00132511	133.48	Actn1;Actn4;Actn2;Actn3
Q9JI91_T_237_1	0	0	80259000	0	0	0.374706021	1860	Q9JI91	237	Q9JI91	T	1	199.327	3.4E-13	199.33	Actn2
Q9JIF9_S_231_1	0	0	23155000	0	0	0.108103987	1862	Q9JIF9	231	Q9JIF9	S	1	8.37518	0.000143659	152.71	Myot
Q9JJW5_S_116_1	22748000	13343000	113470000	0.288721859	0.219293341	0.52975856	1867	Q9JJW5	116	Q9JJW5	S	1	165.887	1E-10	190.24	Myoz2
Q9JJW5_T_107_1	0	0	152410000	0	0	0.711558139	1867	Q9JJW5	107	Q9JJW5	T	1	10.734	0.00000393	145.54	Myoz2
Q9JKB3-2_S_259_1	0	4443600	18977000	0	0.073030944	0.088598116	1872	Q9JKB3-2	259	Q9JKB3-2	S	1	41.6441	0.0405342	97.767	Ybx3
Q9JKS4-3_S_171_1	58509000	26708000	86322000	0.742607142	0.438948254	0.403012412	1878;1879	Q9JKS4-3;Q9JKS4-5	171	Q9JKS4-3	S	1	32.5078	0.00054323	143.31	Ldb3
Q9JKS4-3_S_179_1	544590000	351500000	439650000	6.912037864	5.776932434	2.05259849	1878;1879	Q9JKS4-3;Q9JKS4-5	179	Q9JKS4-3	S	1	26.8989	0.00057636	142.45	Ldb3
Q9JLV1_S_360_1	22216000	10037000	41345000	0.281969616	0.16495895	0.193027828	1883	Q9JLV1	360	Q9JLV1	S	1	37.8868	0.00000651	171.78	Bag3
Q9QXA6_S_5_1	32468000	43078000	29680000	0.412089912	0.707990599	0.138567322	1887	Q9QXA6	5	Q9QXA6	S	1	17.2942	0.0408325	44.611	Slc7a9
Q9QYG0_S_332_1	0	8026000	34332000	0	0.131907993	0.160286163	1892	Q9QYG0	332	Q9QYG0	S	1	32.2813	0.000254004	147.52	Ndrg2
Q9WTX5_T_131_1	8168200	16897000	0	0.103672318	0.277703634	0	1915	Q9WTX5	131	Q9WTX5	T	1	66.19	0.0200581	66.19	Skp1;Skp1a
Q9Z2I9_S_279_1	0	0	18577000	0	0	0.086730632	1941	Q9Z2I9	279	Q9Z2I9	S	1	15.3133	0.0132387	103.91	Sucla2
REV__A0A087WNL0_S_109_1	505170000	312450000	1203100000	6.411711871	5.135142358	5.616925381	1947	REV__A0A087WNL0	109	REV__A0A087WNL0	S	1	95.0987	0.0603301	95.099	
REV__A0A0R4J1H6_T_499_1	77991000	28356000	0	0.98987632	0.466033275	0	1952	REV__A0A0R4J1H6	499	REV__A0A0R4J1H6	T	1	36.0226	0.0603301	64.374	
REV__F8WIE5_S_2240_1	42851000	8945500	36778000	0.543872885	0.147020054	0.171705828	1965	REV__F8WIE5	2240	REV__F8WIE5	S	1	128.755	0.0513161	128.75	
REV__Q3TAY5_S_15_2	125580000	31167000	31075000	1.593884785	0.512232299	0.145080173	1970	REV__Q3TAY5	15	REV__Q3TAY5	S	2	43.1079	0.0603301	43.897	
S4R1M2_S_366_1	20376000	0	97959000	0.258615993	0	0.457342194	508	S4R1M2	366	S4R1M2	S	1	57.283	5.64E-32	237.81	Safb;Safb2
V9GWU5_S_145_1	0	0	33423000	0	0	0.156042305	408	V9GWU5	145	V9GWU5	S	1	39.2728	0.00715465	120.84	Tpd52l2
Z4YJE4_S_6512_1	0	2090900	219500000	0	0.03436412	1.024781914	370	Z4YJE4	6512	Z4YJE4	S	1	29.326	0.000000295	163.45	Obscn
Z4YJF5_S_938_1	0	0	38578000	0	0	0.180109507	1355	Z4YJF5	938	Z4YJF5	S	1	55.2389	0.0391915	56.488	Myom1
Z4YKA3_T_13_1	128000000	27203000	87037000	1.624599876	0.447083622	0.40635054	1247	Z4YKA3	13	Z4YKA3	T	1	22.6091	0.0081495	107.09	Hp1bp3
Z4YLP6_S_150_4	0	0	42472000	0	0	0.198289465	1309	Z4YLP6	150	Z4YLP6	S	4	50.2923	0.037175	50.292	Ccdc88a
Z4YLP6_S_154_4	0	0	42472000	0	0	0.198289465	1309	Z4YLP6	154	Z4YLP6	S	4	50.2923	0.037175	50.292	Ccdc88a
Z4YLP6_T_155_4	0	0	42472000	0	0	0.198289465	1309	Z4YLP6	155	Z4YLP6	T	4	50.2923	0.037175	50.292	Ccdc88a
Z4YLP6_Y_145_4	0	0	42472000	0	0	0.198289465	1309	Z4YLP6	145	Z4YLP6	Y	4	50.2923	0.037175	50.292	Ccdc88a
