# Supplementary material for: Comparative Proteomic and Phosphoproteomic Analyses Reveal Molecular Signatures of Myocardial Infarction and Transverse Aortic Constriction in Aged Mouse Models
Source: Cardiol Res Pract. 2024 Oct 28;2024:9395213. doi: 10.1155/2024/9395213 (PMC11535427; doi:10.1155/2024/9395213)
Supplement: Supporting Information — Table S6: List of significant differentially expressed phosphoproteins (DPPs) in MI vs sham. [file 9395213.f6.pdf]

Track_id	Intensity B11A	Intensity B15A	Intensity B11A.1	Intensity B15A.1	foldchange	p.value	Protein group IDs	Leading proteins	Position	Protein	Amino acid	Number of Phospho (STY)	Score diff	PEP	Score	Gene names	Intensity B11A___1	Intensity B11A___2	Intensity B11A___3	Intensity B15A___1	Intensity B15A___2	Intensity B15A___3
A0A1B0GSC6_S_143_1	44151000	17289000	0.560372728	0.284146187	1.972128269	1	188	A0A1B0GSC6	143	A0A1B0GSC6	S	1	87.94	0.0127801	93.258		44151000	0	0	17289000	0	0
A0A1Y7VP73_Y_243_1	29977000	14716000	0.380473676	0.241858713	1.57312371	1	256	A0A1Y7VP73	243	A0A1Y7VP73	Y	1	59.2052	0.0569296	59.205		29977000	0	0	14716000	0	0
A0A3B2W8B1_T_92_2	48761000	8575500	0.618883708	0.140939073	4.391143597	1	300	A0A3B2W8B1	92	A0A3B2W8B1	T	2	45.598	0.0454788	45.598	Vmn2r3;Vmn2r2;Vmn2r5;Vmn2r1;Vmn2r4;Vmn2r6;Vmn2r7	0	48761000	0	0	8575500	0
A0A3B2W8B1_T_96_2	48761000	8575500	0.618883708	0.140939073	4.391143597	1	300	A0A3B2W8B1	96	A0A3B2W8B1	T	2	45.598	0.0454788	45.598	Vmn2r3;Vmn2r2;Vmn2r5;Vmn2r1;Vmn2r4;Vmn2r6;Vmn2r7	0	48761000	0	0	8575500	0
A2ASS6_S_264_1;2	96574000	19116000	1.225735222	0.314173088	3.90146473	1	353;400;401	A0A5K1VVQ9;A2ASS6;A2ASS6-3	264	A2ASS6	S	1;2	14.5299	0.00000229	175.01	Ttn	87027000	9546600	0	19116000	0	0
A2ASS6_S_34109_1;2	12639000	6489900	0.160416546	0.106662059	1.503970086	1	353;400	A0A5K1VVQ9;A2ASS6	34109	A2ASS6	S	1;2	42.7422	0.0251378	93.096	Ttn	12639000	0	0	0	6489900	0
A2AUL9_S_353_1	32263000	12398000	0.409488014	0.203762186	2.00963693	1	410	A2AUL9	353	A2AUL9	S	1	40.8364	0.0588548	44.457	Eif2ak4	32263000	0	0	12398000	0	0
D3YTR7_S_199_1	30382000	6688600	0.385614011	0.109927711	3.507887206	1	260	D3YTR7	199	D3YTR7	S	1	16.187	0.0141245	102.46	Cap2	30382000	0	0	6688600	0	0
D3YVV9_S_895_1	25884000	7100500	0.328524556	0.116697322	2.815185048	1	497	D3YVV9	895	D3YVV9	S	1	55.9479	0.000000564	176.08	Synpo2	25884000	0	0	7100500	0	0
D3Z313_S_95_1	127890000	23811000	1.623203736	0.391335813	4.147853792	1	530	D3Z313	95	D3Z313	S	1	31.1731	0.00977603	109.72	Cbx3	127890000	0	0	23811000	0	0
E0CYV9_S_1338_1	21379000	5414800	0.271346256	0.088992699	3.049084465	1	577	E0CYV9	1338	E0CYV9	S	1	45.3656	0.0168153	106.58	1110002E22Rik	21379000	0	0	5414800	0	0
E9PV63_S_10_3	170210000	198210000	2.16033707	3.25759823	0.663168665	1	591	E9PV63	10	E9PV63	S	3	41.2419	0.0393165	51.286	Gstm5	0	0	170210000	0	0	198210000
E9PV63_T_8_3	170210000	198210000	2.16033707	3.25759823	0.663168665	1	591	E9PV63	8	E9PV63	T	3	41.2419	0.0393165	51.286	Gstm5	0	0	170210000	0	0	198210000
E9PV63_Y_16_3	170210000	198210000	2.16033707	3.25759823	0.663168665	1	591	E9PV63	16	E9PV63	Y	3	41.2419	0.0393165	51.286	Gstm5	0	0	170210000	0	0	198210000
E9Q1Q4_S_201_2	99864000	39516000	1.267492516	0.649448825	1.951643405	1	622	E9Q1Q4	201	E9Q1Q4	S	2	28.4192	0.0529053	49.418	Tro	0	99864000	0	0	39516000	0
E9Q1Q4_Y_195_2	99864000	39516000	1.267492516	0.649448825	1.951643405	1	622	E9Q1Q4	195	E9Q1Q4	Y	2	27.6726	0.0529053	49.418	Tro	0	99864000	0	0	39516000	0
E9Q9Q7_S_89_1	18444000	25464000	0.234094688	0.418503009	0.559362019	1	620	E9Q9Q7	89	E9Q9Q7	S	1	30.3516	0.00225526	123.29	Ablim1	18444000	0	0	25464000	0	0
E9Q9T8_S_281_1	3375000000	5965800000	42.83612955	98.04843105	0.436887455	1	670	E9Q9T8	281	E9Q9T8	S	1	17.2372	0.0400393	94.616	Mybpc3	3375000000	0	0	5965800000	0	0
E9QKA4_S_448_1	17538000	3219000	0.222595567	0.052904539	4.207494692	1	368	E9QKA4	448	E9QKA4	S	1	8.04621	0.0056372	79.875	Srrm1	17538000	0	0	3219000	0	0
E9QQ25_S_860_1	6994600	16504000	0.088776768	0.271244645	0.327294085	1	688	E9QQ25	860	E9QQ25	S	1	30.9349	0.00319718	116.78	Speg	6994600	0	0	16504000	0	0
F6TAZ4_S_27_1	3860900	5214700	0.049003263	0.085704039	0.571773088	1	703	F6TAZ4	27	F6TAZ4	S	1	30.7246	0.0449822	90.827	Rbm20	3860900	0	0	5214700	0	0
O35887_Y_47_1	82681000	4449300	1.049402675	0.073124624	14.3508796	1	837;1406	O35887;Q6XLQ8	47	O35887	Y	1	118.156	0.0000056	118.16	Calu	82681000	0	0	4449300	0	0
O54724_S_169_1	95280000	38930000	1.209311533	0.639817865	1.890087163	1	839	O54724	169	O54724	S	1	14.6303	0.0414199	100.82	Ptrf	95280000	0	0	38930000	0	0
O55143-2_S_663_1	16599000	36242000	0.210677604	0.595640356	0.353699345	1	851	O55143-2	663	O55143-2	S	1	38.896	0.00000298	180.24	Atp2a2	16599000	0	0	36242000	0	0
P14602-3_S_52_1	39227000	48426000	0.497876401	0.795885434	0.625562901	1	954	P14602-3	52	P14602-3	S	1	25.9706	0.00943768	110.12	Hspb1	39227000	0	0	48426000	0	0
P20152_S_73_1	424130000	21651000	5.383137074	0.355836029	15.12813947	1	978	P20152	73	P20152	S	1	8.89287	0.041851	100.48	Vim	424130000	0	0	21651000	0	0
P36552_S_233_3	18455000	54174000	0.234234302	0.890354303	0.263079879	1	1036	P36552	233	P36552	S	3	44.3175	0.0498013	44.318	Cpox	0	0	18455000	0	0	54174000
P36552_S_234_3	18455000	54174000	0.234234302	0.890354303	0.263079879	1	1036	P36552	234	P36552	S	3	44.3175	0.0498013	44.318	Cpox	0	0	18455000	0	0	54174000
P36552_T_228_3	18455000	54174000	0.234234302	0.890354303	0.263079879	1	1036	P36552	228	P36552	T	3	44.3175	0.0498013	44.318	Cpox	0	0	18455000	0	0	54174000
P48678_S_390_1	93413000	14272000	1.185615221	0.234561535	5.054602073	1	1066	P48678	390	P48678	S	1	20.0427	0.0317407	101.72	Lmna	93413000	0	0	14272000	0	0
P53986_S_213_1	338970000	437200000	4.302270469	7.185419232	0.598750098	1	1099	P53986	213	P53986	S	1	70.4503	0.000169253	175.48	Slc16a1	338970000	0	0	437200000	0	0
P53986_S_461_1	59241000	27100000	0.751897822	0.445390808	1.688175437	1	1099	P53986	461	P53986	S	1	13.0249	1.01E-74	321.02	Slc16a1	59241000	0	0	27100000	0	0
Q3UKG2_S_372_1	6782000	1096000	0.086078409	0.018012853	4.778721561	1	17	Q3UKG2	372	Q3UKG2	S	1	44.453	0.00892035	87.895	Prob1	6782000	0	0	1096000	0	0
Q3UTJ2-2_S_345_1	11972000	4846200	0.151950857	0.07964771	1.907786891	1	1288	Q3UTJ2-2	345	Q3UTJ2-2	S	1	23.6243	0.00000656	167.89	Sorbs2	11972000	0	0	4846200	0	0
Q5EBP8_S_6_1	28940000	4737800	0.367311878	0.077866146	4.717221756	1	1072	Q5EBP8	6	Q5EBP8	S	1	30.0465	0.000695314	108.66	Hnrnpa1	28940000	0	0	4737800	0	0
Q5GIG6_S_827_1	7205000	11443000	0.091447204	0.188066679	0.486248837	1	1306	Q5GIG6	827	Q5GIG6	S	1	15.6226	0.00413419	128.85	Tnni3k	7205000	0	0	11443000	0	0
Q62261_S_2102_1	268730000	119790000	3.410771287	1.968758851	1.732447468	1	1356	Q62261	2102	Q62261	S	1	93.1586	0.00414152	128.81	Sptbn1	268730000	0	0	119790000	0	0
Q7TT37_T_936_2	84223000	8235800	1.068974026	0.135356074	7.897495801	1	1430	Q7TT37	936	Q7TT37	T	2	8.76445	0.0328943	82.749	Ikbkap	0	84223000	0	0	8235800	0
Q7TT37_Y_932_2	84223000	8235800	1.068974026	0.135356074	7.897495801	1	1430	Q7TT37	932	Q7TT37	Y	2	7.37118	0.0328943	82.749	Ikbkap	0	84223000	0	0	8235800	0
Q8BGD9_S_422_1	19412000	6243300	0.246380725	0.102609167	2.401157053	1	1456	Q8BGD9	422	Q8BGD9	S	1	24.9064	0.00000205	168.45	Eif4b	19412000	0	0	6243300	0	0
Q8BJU0-2_S_306_1	15194000	4406100	0.192845082	0.072414629	2.663068011	1	1471	Q8BJU0-2	306	Q8BJU0-2	S	1	11.9453	0.00269062	133.87	Sgta	15194000	0	0	4406100	0	0
Q8BND3-2_S_460_2	33505000	15275000	0.42525171	0.251045926	1.69391998	1	1485	Q8BND3-2	460	Q8BND3-2	S	2	87.5248	0.00650955	90.653	Wdr35	0	33505000	0	0	15275000	0

Q8BND3-2_T_458_2	33505000	15275000	0.42525171	0.251045926	1.69391998	1	1485	Q8BND3-2	458	Q8BND3-2	T	2	87.5248	0.00650955	90.653	Wdr35	0	33505000	0	0	15275000	0
Q8C120-4_S_11_2	71576000	18838000	0.908455943	0.309604134	2.934250041	1	1515	Q8C120-4	11	Q8C120-4	S	2	58.9807	0.0330623	58.981	Sh3rf3	0	71576000	0	0	18838000	0
Q8C120-4_S_6_2	71576000	18838000	0.908455943	0.309604134	2.934250041	1	1515	Q8C120-4	6	Q8C120-4	S	2	58.9807	0.0330623	58.981	Sh3rf3	0	71576000	0	0	18838000	0
Q8JZZ5_S_66_3	41998000	16391000	0.53304645	0.269387481	1.978735047	1	1098	Q8JZZ5	66	Q8JZZ5	S	3	35.7438	0.0537325	41.117	Pitpnb	0	0	41998000	0	0	16391000
Q8JZZ5_Y_62_3	41998000	16391000	0.53304645	0.269387481	1.978735047	1	1098	Q8JZZ5	62	Q8JZZ5	Y	3	23.2207	0.0537325	41.117	Pitpnb	0	0	41998000	0	0	16391000
Q8VDD5_S_1943_1	72767000	5359800	0.923572337	0.088088769	10.48456401	1	1595	Q8VDD5	1943	Q8VDD5	S	1	58.9628	3.81E-11	190.19	Myh9	72767000	0	0	5359800	0	0
Q8VDN2_S_16_1	16335000	37523000	0.207326867	0.6166937	0.336190992	1	1597	Q8VDN2	16	Q8VDN2	S	1	78.7354	0.0251219	98.942	Atp1a1	16335000	0	0	37523000	0	0
Q921W0_T_11_1	18808000	7337600	0.238714644	0.120594081	1.979488894	1	1635	Q921W0	11	Q921W0	T	1	18.2168	0.0437898	84.479	Chmp1a	18808000	0	0	7337600	0	0
Q99L43_S_32_1	18627000	6238200	0.236417359	0.102525348	2.305940566	1	1663	Q99L43	32	Q99L43	S	1	17.0924	0.0120292	107.65	Cds2	18627000	0	0	6238200	0	0
Q99LI7_Y_343_2	66368000	8702600	0.842355036	0.143027972	5.889442633	1	1669	Q99LI7	343	Q99LI7	Y	2	40.4886	0.0532816	44.847	Cstf3	0	66368000	0	0	8702600	0
Q99LI7_Y_346_2	66368000	8702600	0.842355036	0.143027972	5.889442633	1	1669	Q99LI7	346	Q99LI7	Y	2	40.4886	0.0532816	44.847	Cstf3	0	66368000	0	0	8702600	0
Q9D338_S_18_2	193160000	94534000	2.451622751	1.553674341	1.577951496	1	1780	Q9D338	18	Q9D338	S	2	54.2655	0.0526462	63.48	Mrpl19	0	193160000	0	0	94534000	0
Q9D338_Y_14_2	193160000	94534000	2.451622751	1.553674341	1.577951496	1	1780	Q9D338	14	Q9D338	Y	2	53.8918	0.0526462	63.48	Mrpl19	0	193160000	0	0	94534000	0
Q9D8U8_Y_310_2	123510000	50414000	1.567611959	0.828558383	1.891975256	1	1806	Q9D8U8	310	Q9D8U8	Y	2	34.8656	0.0521665	40.589	Snx5	0	123510000	0	0	50414000	0
Q9DBC7_Y_53_1	10013000	13424000	0.127086864	0.220624583	0.5760322	1	1814	Q9DBC7	53	Q9DBC7	Y	1	63.5651	0.0479226	63.565	Prkar1a	10013000	0	0	13424000	0	0
Q9JKS4-3_S_171_1	58509000	26708000	0.742607142	0.438948254	1.691787438	1	1878;1879	Q9JKS4-3;Q9JKS4-5	171	Q9JKS4-3	S	1	32.5078	0.00054323	143.31	Ldb3	58509000	0	0	26708000	0	0
Q9JLV1_S_360_1	22216000	10037000	0.281969616	0.16495895	1.709332025	1	1883	Q9JLV1	360	Q9JLV1	S	1	37.8868	0.00000651	171.78	Bag3	22216000	0	0	10037000	0	0
Q9QXA6_S_5_1	32468000	43078000	0.412089912	0.707990599	0.582055627	1	1887	Q9QXA6	5	Q9QXA6	S	1	17.2942	0.0408325	44.611	Slc7a9	32468000	0	0	43078000	0	0
REV__F8WIE5_S_2240_1	42851000	8945500	0.543872885	0.147020054	3.699310878	1	1965	REV__F8WIE5	2240	REV__F8WIE5	S	1	128.755	0.0513161	128.75		42851000	0	0	8945500	0	0
REV__Q3TAY5_S_15_2	125580000	31167000	1.593884785	0.512232299	3.11164444	1	1970	REV__Q3TAY5	15	REV__Q3TAY5	S	2	43.1079	0.0603301	43.897		0	125580000	0	0	31167000	0
Z4YKA3_T_13_1	128000000	27203000	1.624599876	0.447083622	3.633771841	1	1247	Z4YKA3	13	Z4YKA3	T	1	22.6091	0.0081495	107.09	Hp1bp3	128000000	0	0	27203000	0	0
