# Supplementary material for: Comparative Proteomic and Phosphoproteomic Analyses Reveal Molecular Signatures of Myocardial Infarction and Transverse Aortic Constriction in Aged Mouse Models
Source: Cardiol Res Pract. 2024 Oct 28;2024:9395213. doi: 10.1155/2024/9395213 (PMC11535427; doi:10.1155/2024/9395213)
Supplement: Supporting Information — Table S7: List of significant differentially expressed phosphoproteins (DPPs) in TAC vs sham. [file 9395213.f7.pdf]

Track_id	Intensity B15A	Intensity B17A	Intensity B15A.1	Intensity B17A.1	foldchange	p.value	Protein group IDs	Leading proteins	Position	Protein	Amino acid	Number of Phospho (STY)	Score diff	PEP	Score	Gene names	Intensity B15A___1	Intensity B15A___2	Intensity B15A___3	Intensity B17A___1	Intensity B17A___2	Intensity B17A___3
A0A1B0GSC6_S_143_1	17289000	107050000	0.284146187	0.499785439	1.758902501	1	188	A0A1B0GSC6	143	A0A1B0GSC6	S	1	87.94	0.0127801	93.258		17289000	0	0	107050000	0	0
A0A1W2P6K9_S_101_1	4918700	38483000	0.080839253	0.179665979	2.222509144	1	229	A0A1W2P6K9	101	A0A1W2P6K9	S	1	20.6522	0.0441692	94.454	Sgpp1	4918700	0	0	38483000	0	0
A0A1Y7VP73_Y_243_1	14716000	24126000	0.241858713	0.112637305	0.465715308	1	256	A0A1Y7VP73	243	A0A1Y7VP73	Y	1	59.2052	0.0569296	59.205		14716000	0	0	24126000	0	0
A0A3B2W8B1_T_92_2	8575500	72958000	0.140939073	0.340619767	2.416787338	1	300	A0A3B2W8B1	92	A0A3B2W8B1	T	2	45.598	0.0454788	45.598	Vmn2r3;Vmn2r2;Vmn2r5;Vmn2r1;Vmn2r4;Vmn2r6;Vmn2r7	0	8575500	0	0	72958000	0
A0A3B2W8B1_T_96_2	8575500	72958000	0.140939073	0.340619767	2.416787338	1	300	A0A3B2W8B1	96	A0A3B2W8B1	T	2	45.598	0.0454788	45.598	Vmn2r3;Vmn2r2;Vmn2r5;Vmn2r1;Vmn2r4;Vmn2r6;Vmn2r7	0	8575500	0	0	72958000	0
A2ASS6_S_2080_1	118040000	1326700000	1.939997452	6.193977976	3.192776346	1	353;400;401	A0A5K1VVQ9;A2ASS6;A2ASS6-3	2080	A2ASS6	S	1	42.9742	0.00793451	124.6	Ttn	118040000	0	0	1326700000	0	0
A2ASS6_S_264_1;2	19116000	502910000	0.314173088	2.347941105	7.473399838	1	353;400;401	A0A5K1VVQ9;A2ASS6;A2ASS6-3	264	A2ASS6	S	1;2	14.5299	0.00000229	175.01	Ttn	19116000	0	0	443510000	59399000	0
A2ASS6_S_322_1	43978000	565210000	0.722782175	2.638801758	3.650894902	1	353;400;401	A0A5K1VVQ9;A2ASS6;A2ASS6-3	322	A2ASS6	S	1	42.5908	0.0154305	101.38	Ttn	43978000	0	0	565210000	0	0
A2ASS6_S_34109_1;2	6489900	56407000	0.106662059	0.263347943	2.468993618	1	353;400	A0A5K1VVQ9;A2ASS6	34109	A2ASS6	S	1;2	42.7422	0.0251378	93.096	Ttn	0	6489900	0	33645000	22762000	0
D3YVV9_S_895_1	7100500	163300000	0.116697322	0.762400395	6.53314388	1	497	D3YVV9	895	D3YVV9	S	1	55.9479	0.000000564	176.08	Synpo2	7100500	0	0	163300000	0	0
D3Z313_S_95_1	23811000	30054000	0.391335813	0.14031342	0.358549909	1	530	D3Z313	95	D3Z313	S	1	31.1731	0.00977603	109.72	Cbx3	23811000	0	0	30054000	0	0
E0CYV9_S_1338_1	5414800	80216000	0.088992699	0.374505267	4.208269568	1	577	E0CYV9	1338	E0CYV9	S	1	45.3656	0.0168153	106.58	1110002E22Rik	5414800	0	0	80216000	0	0
E9Q1Q4_S_201_2	39516000	8492500	0.649448825	0.039649022	0.061050264	1	622	E9Q1Q4	201	E9Q1Q4	S	2	28.4192	0.0529053	49.418	Tro	0	39516000	0	0	8492500	0
E9Q1Q4_Y_195_2	39516000	8492500	0.649448825	0.039649022	0.061050264	1	622	E9Q1Q4	195	E9Q1Q4	Y	2	27.6726	0.0529053	49.418	Tro	0	39516000	0	0	8492500	0
E9Q9Q7_S_110_1	8905000	77705000	0.146354433	0.362782135	2.478791572	1	620	E9Q9Q7	110	E9Q9Q7	S	1	20.6083	0.00000958	162.36	Ablim1	8905000	0	0	77705000	0	0
E9Q9Q7_S_89_1	25464000	274900000	0.418503009	1.283428466	3.066712636	1	620	E9Q9Q7	89	E9Q9Q7	S	1	30.3516	0.00225526	123.29	Ablim1	25464000	0	0	274900000	0	0
E9Q9T8_S_281_1	5965800000	5846300000	98.04843105	27.29468112	0.278379581	1	670	E9Q9T8	281	E9Q9T8	S	1	17.2372	0.0400393	94.616	Mybpc3	5965800000	0	0	5846300000	0	0
E9QKA4_S_448_1	3219000	37058000	0.052904539	0.173013067	3.270287772	1	368	E9QKA4	448	E9QKA4	S	1	8.04621	0.0056372	79.875	Srrm1	3219000	0	0	37058000	0	0
E9QQ25_S_860_1	16504000	9324400	0.271244645	0.043532922	0.160493203	1	688	E9QQ25	860	E9QQ25	S	1	30.9349	0.00319718	116.78	Speg	16504000	0	0	9324400	0	0
F6TAZ4_S_27_1	5214700	7510000	0.085704039	0.035062014	0.409105737	1	703	F6TAZ4	27	F6TAZ4	S	1	30.7246	0.0449822	90.827	Rbm20	5214700	0	0	7510000	0	0
O35887_Y_47_1	4449300	67965000	0.073124624	0.317308897	4.33928928	1	837;1406	O35887;Q6XLQ8	47	O35887	Y	1	118.156	0.0000056	118.16	Calu	4449300	0	0	67965000	0	0
O54724_S_169_1	38930000	210820000	0.639817865	0.984257509	1.538340148	1	839	O54724	169	O54724	S	1	14.6303	0.0414199	100.82	Ptrf	38930000	0	0	210820000	0	0
O55143-2_S_663_1	36242000	597220000	0.595640356	2.788247175	4.681091781	1	851	O55143-2	663	O55143-2	S	1	38.896	0.00000298	180.24	Atp2a2	36242000	0	0	597220000	0	0
O70548_S_161_1	77040000	135170000	1.266158961	0.631069573	0.498412595	1	863	O70548	161	O70548	S	1	24.4793	0.00539791	78.655	Tcap	77040000	0	0	135170000	0	0
P11499_S_255_1	60192000	776420000	0.989260646	3.624880063	3.664231543	1	938	P11499	255	P11499	S	1	85.0408	2.88E-29	238.32	Hsp90ab1	60192000	0	0	776420000	0	0
P20152_S_73_1	21651000	205580000	0.355836029	0.959793467	2.697291417	1	978	P20152	73	P20152	S	1	8.89287	0.041851	100.48	Vim	21651000	0	0	205580000	0	0
P36552_S_233_3	54174000	44334000	0.890354303	0.206982603	0.232472177	1	1036	P36552	233	P36552	S	3	44.3175	0.0498013	44.318	Cpox	0	0	54174000	0	0	44334000
P36552_S_234_3	54174000	44334000	0.890354303	0.206982603	0.232472177	1	1036	P36552	234	P36552	S	3	44.3175	0.0498013	44.318	Cpox	0	0	54174000	0	0	44334000
P36552_T_228_3	54174000	44334000	0.890354303	0.206982603	0.232472177	1	1036	P36552	228	P36552	T	3	44.3175	0.0498013	44.318	Cpox	0	0	54174000	0	0	44334000
P48678_S_390_1	14272000	103190000	0.234561535	0.481764218	2.053892673	1	1066	P48678	390	P48678	S	1	20.0427	0.0317407	101.72	Lmna	14272000	0	0	103190000	0	0
P48962_S_149_1	32196000	54948000	0.529144002	0.256536294	0.484813762	1	1071	P48962	149	P48962	S	1	11.6803	0.0533003	95.854	Slc25a4	32196000	0	0	54948000	0	0
P50462_S_111_1	9908400	6690000	0.162845398	0.031233672	0.191799537	1	1078	P50462	111	P50462	S	1	10.5276	0.0357721	101.32	Csrp3	9908400	0	0	6690000	0	0
P53986_S_213_1	437200000	847760000	7.185419232	3.957945857	0.550830192	1	1099	P53986	213	P53986	S	1	70.4503	0.000169253	175.48	Slc16a1	437200000	0	0	847760000	0	0
P61014_S_16_1;2	61692000	107440000	1.013913274	0.501606236	0.494723019	1	1128	P61014	16	P61014	S	1;2	40.373	0.00105822	101.45	Pln	61692000	0	0	75963000	31476000	0
Q3U3Q1-2_S_339_2	41909000	26722000	0.688777984	0.124757277	0.181128433	1	1264	Q3U3Q1-2	339	Q3U3Q1-2	S	2	67.1532	0.0183967	69.721	Ulk3	0	41909000	0	0	26722000	0
Q3U3Q1-2_Y_337_2	41909000	26722000	0.688777984	0.124757277	0.181128433	1	1264	Q3U3Q1-2	337	Q3U3Q1-2	Y	2	67.1532	0.0183967	69.721	Ulk3	0	41909000	0	0	26722000	0
Q3UKG2_S_372_1	1096000	15736000	0.018012853	0.073466826	4.078578002	1	17	Q3UKG2	372	Q3UKG2	S	1	44.453	0.00892035	87.895	Prob1	1096000	0	0	15736000	0	0
Q3UTJ2-2_S_345_1	4846200	90266000	0.07964771	0.421425805	5.291122683	1	1288	Q3UTJ2-2	345	Q3UTJ2-2	S	1	23.6243	0.00000656	167.89	Sorbs2	4846200	0	0	90266000	0	0
Q3UVT7_S_278_1	8344700	146400000	0.137145855	0.68349919	4.983739319	1	115	Q3UVT7	278	Q3UVT7	S	1	94.6917	0.00272769	125.97	Palmd	8344700	0	0	146400000	0	0
Q5EBG6_S_16_1	16934000	545400000	0.278311732	2.546314606	9.149145772	1	1303	Q5EBG6	16	Q5EBG6	S	1	71.2266	0.00394569	121.45	Hspb6	16934000	0	0	545400000	0	0
Q5GIG6_S_827_1	11443000	13560000	0.188066679	0.063307712	0.336623757	1	1306	Q5GIG6	827	Q5GIG6	S	1	15.6226	0.00413419	128.85	Tnni3k	11443000	0	0	13560000	0	0
Q65CL1_S_650_1	8618700	14917000	0.141649068	0.069643152	0.491659797	1	1372	Q65CL1	650	Q65CL1	S	1	13.8042	0.0141629	102.4	Ctnna3	8618700	0	0	14917000	0	0
Q6P1H6-3_S_699_1	36837000	53952000	0.605419232	0.251886259	0.416052622	1	1387	Q6P1H6-3	699	Q6P1H6-3	S	1	91.6263	0.00981445	132.29	Ankle2	36837000	0	0	53952000	0	0

Q7TMI3-3_T_13_2	22414000	50095000	0.368375999	0.233879043	0.634892185	1	1423	Q7TMI3-3	13	Q7TMI3-3	T	2	7.98905	0.0428271	71.279	Uhrf2	0	22414000	0	0	50095000	0
Q7TMI3-3_T_15_2	22414000	50095000	0.368375999	0.233879043	0.634892185	1	1423	Q7TMI3-3	15	Q7TMI3-3	T	2	5.32353	0.0428271	71.279	Uhrf2	0	22414000	0	0	50095000	0
Q7TT37_T_936_2	8235800	262920000	0.135356074	1.227497316	9.068653365	1	1430	Q7TT37	936	Q7TT37	T	2	8.76445	0.0328943	82.749	Ikbkap	0	8235800	0	0	262920000	0
Q7TT37_Y_932_2	8235800	262920000	0.135356074	1.227497316	9.068653365	1	1430	Q7TT37	932	Q7TT37	Y	2	7.37118	0.0328943	82.749	Ikbkap	0	8235800	0	0	262920000	0
Q8BGD9_S_422_1	6243300	10403000	0.102609167	0.048568593	0.473335808	1	1456	Q8BGD9	422	Q8BGD9	S	1	24.9064	0.00000205	168.45	Eif4b	6243300	0	0	10403000	0	0
Q8BJU0-2_S_306_1	4406100	30541000	0.072414629	0.142587082	1.969036975	1	1471	Q8BJU0-2	306	Q8BJU0-2	S	1	11.9453	0.00269062	133.87	Sgta	4406100	0	0	30541000	0	0
Q8BND3-2_S_460_2	15275000	577180000	0.251045926	2.69468622	10.73383768	1	1485	Q8BND3-2	460	Q8BND3-2	S	2	87.5248	0.00650955	90.653	Wdr35	0	15275000	0	0	577180000	0
Q8BND3-2_T_458_2	15275000	577180000	0.251045926	2.69468622	10.73383768	1	1485	Q8BND3-2	458	Q8BND3-2	T	2	87.5248	0.00650955	90.653	Wdr35	0	15275000	0	0	577180000	0
Q8C120-4_S_11_2	18838000	134790000	0.309604134	0.629295463	2.032580944	1	1515	Q8C120-4	11	Q8C120-4	S	2	58.9807	0.0330623	58.981	Sh3rf3	0	18838000	0	0	134790000	0
Q8C120-4_S_6_2	18838000	134790000	0.309604134	0.629295463	2.032580944	1	1515	Q8C120-4	6	Q8C120-4	S	2	58.9807	0.0330623	58.981	Sh3rf3	0	18838000	0	0	134790000	0
Q8JZZ5_S_66_3	16391000	25536000	0.269387481	0.119220187	0.442560235	1	1098	Q8JZZ5	66	Q8JZZ5	S	3	35.7438	0.0537325	41.117	Pitpnb	0	0	16391000	0	0	25536000
Q8JZZ5_Y_62_3	16391000	25536000	0.269387481	0.119220187	0.442560235	1	1098	Q8JZZ5	62	Q8JZZ5	Y	3	23.2207	0.0537325	41.117	Pitpnb	0	0	16391000	0	0	25536000
Q8VDD5_S_1943_1	5359800	65583000	0.088088769	0.306188029	3.475903143	1	1595	Q8VDD5	1943	Q8VDD5	S	1	58.9628	3.81E-11	190.19	Myh9	5359800	0	0	65583000	0	0
Q8VDN2_S_16_1	37523000	11317000	0.6166937	0.052835795	0.085675912	1	1597	Q8VDN2	16	Q8VDN2	S	1	78.7354	0.0251219	98.942	Atp1a1	37523000	0	0	11317000	0	0
Q921W0_T_11_1	7337600	47075000	0.120594081	0.219779538	1.822473675	1	1635	Q921W0	11	Q921W0	T	1	18.2168	0.0437898	84.479	Chmp1a	7337600	0	0	47075000	0	0
Q99LI7_Y_343_2	8702600	52076000	0.143027972	0.243127758	1.699861605	1	1669	Q99LI7	343	Q99LI7	Y	2	40.4886	0.0532816	44.847	Cstf3	0	8702600	0	0	52076000	0
Q99LI7_Y_346_2	8702600	52076000	0.143027972	0.243127758	1.699861605	1	1669	Q99LI7	346	Q99LI7	Y	2	40.4886	0.0532816	44.847	Cstf3	0	8702600	0	0	52076000	0
Q9D8U8_Y_310_2	50414000	328300000	0.828558383	1.532737597	1.849884846	1	1806	Q9D8U8	310	Q9D8U8	Y	2	34.8656	0.0521665	40.589	Snx5	0	50414000	0	0	328300000	0
Q9DBC7_Y_53_1	13424000	9202000	0.220624583	0.042961472	0.194726587	1	1814	Q9DBC7	53	Q9DBC7	Y	1	63.5651	0.0479226	63.565	Prkar1a	13424000	0	0	9202000	0	0
Q9JJW5_S_116_1	13343000	113470000	0.219293341	0.52975856	2.415753062	1	1867	Q9JJW5	116	Q9JJW5	S	1	165.887	1E-10	190.24	Myoz2	13343000	0	0	113470000	0	0
Q9JKS4-3_S_179_1	351500000	439650000	5.776932434	2.05259849	0.355309416	1	1878;1879	Q9JKS4-3;Q9JKS4-5	179	Q9JKS4-3	S	1	26.8989	0.00057636	142.45	Ldb3	351500000	0	0	439650000	0	0
Q9QXA6_S_5_1	43078000	29680000	0.707990599	0.138567322	0.195719155	1	1887	Q9QXA6	5	Q9QXA6	S	1	17.2942	0.0408325	44.611	Slc7a9	43078000	0	0	29680000	0	0
REV__Q3TAY5_S_15_2	31167000	31075000	0.512232299	0.145080173	0.283231208	1	1970	REV__Q3TAY5	15	REV__Q3TAY5	S	2	43.1079	0.0603301	43.897		0	31167000	0	0	31075000	0
