# Supplementary material for: Comparative Proteomic and Phosphoproteomic Analyses Reveal Molecular Signatures of Myocardial Infarction and Transverse Aortic Constriction in Aged Mouse Models
Source: Cardiol Res Pract. 2024 Oct 28;2024:9395213. doi: 10.1155/2024/9395213 (PMC11535427; doi:10.1155/2024/9395213)
Supplement: Supporting Information — Table S8: List of coexpressed phosphoproteins in MI and TAC. [file 9395213.f8.pdf]

MI phos (Num:49)	TAC phos (Num:50)	MI phos|tac phos(Num:38)
A0A1B0GSC6	A0A1B0GSC6	A0A1B0GSC6
A0A1Y7VP73	A0A1W2P6K9	A0A1Y7VP73
A0A3B2W8B1	A0A1Y7VP73	A0A3B2W8B1
A2ASS6	A0A3B2W8B1	A2ASS6
A2AUL9	A2ASS6	D3YVV9
D3YTR7	D3YVV9	D3Z313
D3YVV9	D3Z313	E0CYV9
D3Z313	E0CYV9	E9Q1Q4
E0CYV9	E9Q1Q4	E9Q9Q7
E9PV63	E9Q9Q7	E9Q9T8
E9Q1Q4	E9Q9T8	E9QKA4
E9Q9Q7	E9QKA4	E9QQ25
E9Q9T8	E9QQ25	F6TAZ4
E9QKA4	F6TAZ4	O35887
E9QQ25	O35887	O54724
F6TAZ4	O54724	O55143-2
O35887	O55143-2	P20152
O54724	O70548	P36552
O55143-2	P11499	P48678
P14602-3	P20152	P53986
P20152	P36552	Q3UKG2
P36552	P48678	Q3UTJ2-2
P48678	P48962	Q5GIG6
P53986	P53986	Q7TT37
Q3UKG2	P61014	Q8BGD9
Q3UTJ2-2	Q3U3Q1-2	Q8BJU0-2
Q5EBP8	Q3UKG2	Q8BND3-2
Q5GIG6	Q3UTJ2-2	Q8C120-4
Q62261	Q3UVT7	Q8JZZ5
Q7TT37	Q5EBG6	Q8VDD5
Q8BGD9	Q5GIG6	Q8VDN2
Q8BJU0-2	Q65CL1	Q921W0
Q8BND3-2	Q6P1H6-3	Q99LI7
Q8C120-4	Q7TMI3-3	Q9D8U8
Q8JZZ5	Q7TT37	Q9DBC7
Q8VDD5	Q8BGD9	Q9JKS4-3
Q8VDN2	Q8BJU0-2	Q9QXA6
Q921W0	Q8BND3-2	REV__Q3TAY5
Q99L43	Q8C120-4	
Q99LI7	Q8JZZ5	
Q9D338	Q8VDD5	
Q9D8U8	Q8VDN2	

Q9DBC7	Q921W0
Q9JKS4-3	Q99LI7
Q9JLV1	Q9D8U8
Q9QXA6	Q9DBC7
REV__F8WIE5	Q9JJW5
REV__Q3TAY5	Q9JKS4-3
Z4YKA3	Q9QXA6
	REV__Q3TAY5

gene names
A0A1B0GSC6
A0A1Y7VP73
Vmn2r3
Ttn
Synpo2
Cbx3
1110002E22Rik
Tro
Ablim1
Mybpc3
Srrm1
Speg
Rbm20
Calu
Ptrf
Atp2a2
Vim
Cpox
Lmna
Slc16a1
Prob1
Sorbs2
Tnni3k
Ikbkap
Eif4b
Sgta
Wdr35
Sh3rf3
Pitpnb
Myh9
Atp1a1
Chmp1a
Cstf3
Snx5
Prkar1a
Ldb3
Slc7a9
REV__Q3TAY5
